# 1-Azinyl-1′-Alkenylferrocenes with Anticholinesterase, Antioxidant, and Antiaggregating Activities as Multifunctional Agents for Potential Treatment of Alzheimer’s Disease

**DOI:** 10.3390/ph18121862

**Published:** 2025-12-05

**Authors:** Galina F. Makhaeva, Irina A. Utepova, Elena V. Rudakova, Nadezhda V. Kovaleva, Natalia P. Boltneva, Elena Yu. Zyryanova, Alexandra A. Musikhina, Vladimir F. Lazarev, Snezhana A. Vladimirova, Irina V. Guzhova, Ilya N. Ganebnykh, Tatiana Y. Astakhova, Elena N. Timokhina, Oleg N. Chupakhin, Valery N. Charushin, Rudy J. Richardson

**Affiliations:** 1 Institute of Physiologically Active Compounds at Federal Research Center of Problems of Chemical Physics and Medicinal Chemistry, Russian Academy of Sciences, Severny proezd 1, Chernogolovka 142432, Russia; gmakh@ipac.ac.ru (G.F.M.); rudakova@ipac.ac.ru (E.V.R.); kovalevanv@ipac.ac.ru (N.V.K.); boltneva@ipac.ac.ru (N.P.B.); astakhova1967.t@yandex.ru (T.Y.A.); 2 Department of Organic and Biomolecular Chemistry, Ural Federal University, Mira Str. 19, Ekaterinburg 620002, Russia; i.a.utepova@urfu.ru (I.A.U.); zyryanovayelena@yandex.ru (E.Y.Z.); alexa5330@yandex.ru (A.A.M.); chupakhin@ios.uran.ru (O.N.C.); charushin@ios.uran.ru (V.N.C.); 3 Institute of Organic Synthesis of the Ural Branch of the Russian Academy of Sciences, S. Kovalevskaya Str. 22, Ekaterinburg 620108, Russia; ing@ios.uran.ru; 4 Institute of Cytology of the Russian Academy of Sciences, Tikhoretsky pr. 4, St. Petersburg 194064, Russia; lazarev@incras.ru (V.F.L.); snezhik1999@mail.ru (S.A.V.); irina.guzhova@incras.ru (I.V.G.); 5 Emanuel Institute of Biochemical Physics Russian Academy of Sciences, Moscow 119334, Russia; arsuna.elena@gmail.com; 6 Department of Environmental Health Sciences, University of Michigan, Ann Arbor, MI 48109, USA; 7 Department of Neurology, University of Michigan, Ann Arbor, MI 48109, USA; 8 Center for Computational Medicine & Bioinformatics, University of Michigan, Ann Arbor, MI 48109, USA; 9 Michigan Institute for Computational Discovery & Engineering, University of Michigan, Ann Arbor, MI 48109, USA; 10 Michigan Institute for Data & AI in Society, University of Michigan, Ann Arbor, MI 48109, USA

**Keywords:** acetylcholinesterase, alkenylferrocene, AlphaFold3, Alzheimer’s disease, β-amyloid, antioxidants, azinylferrocene, butyrylcholinesterase, ligand overlap volume, wittig reaction

## Abstract

**Background/Objectives**: This study focused on synthesizing novel alkenyl derivatives of azinylferrocenes and evaluating their potential as Alzheimer’s disease (AD) therapeutics. **Methods**: 1-Azinyl-1′-acetylferrocenes were obtained by regioselective acetylation of azinylferrocenes, followed by the Wittig reaction or reduction of 1-azinyl-1′-acetylferrocenes and subsequent dehydration of the resulting alcohols. The synthesized compounds underwent the following biological activity testing relevant to AD: inhibition of acetylcholinesterase (AChE), butyrylcholinesterase (BChE), and off-target carboxylesterase (CES); antioxidant capacity (ABTS and FRAP assays); inhibition of Aβ_42_ self-aggregation (thioflavin method); blocking AChE-induced β-amyloid aggregation (propidium displacement); and cytotoxicity in SH-SY5Y and MSC-Neu cells (MTT assay). **Results**: Quinoline and bipyridine derivatives demonstrated effective cholinesterase inhibition, especially quinoline **7b** (AChE IC_50_ 3.32 μM; BChE IC_50_ 3.68 μM), while acridine derivatives were poor inhibitors. Quantum chemical (QC) calculations predicted that acridine derivatives were especially prone to form stable dimers. Molecular docking into protein targets generated by an AlphaFold3 reproduction code showed that these dimers were too bulky to access enzyme active sites, yet they could bind to protein surfaces to inhibit Aβ_42_ self-aggregation and displace propidium from the AChE peripheral anionic site. All compounds showed high antioxidant activity in ABTS and FRAP assays, with quinoline derivatives being 2–4 times more potent than Trolox. QC calculations supported these findings. Quinoline and bipyridine derivatives also exhibited low cytotoxicity and scant CES inhibition. **Conclusions**: Overall, the synthesized ferrocenes, particularly the quinoline and bipyridine derivatives, appear promising for further research as multifunctional therapeutic agents targeting AD due to their anticholinesterase, antiaggregating, and antioxidant activities combined with low toxicity.

## 1. Introduction

A promising scaffold for the synthesis of new biologically active compounds is ferrocene (Fc). Its unique physical and chemical properties, such as inherent stability, resilience in biological environments, strong redox characteristics, and low toxicity, make it widely applicable in medicinal chemistry [1,2,3,4].

Although Fc itself is not biologically active, its presence can significantly influence the properties of organic molecules into which it is integrated [5,6]. Adding an Fc fragment to parent compounds can induce substantial alterations in physicochemical characteristics such as solubility, lipophilicity, and enhanced biological activity. Noteworthy examples include ferrocifen, an Fc-based analog of the anticancer drug tamoxifen, and ferroquine, an Fc derivative of the antimalarial drug chloroquine [6,7].

A functionalized ferrocenyl fragment with different heteroaromatic substituents, such as oxazolines, quinolines, indoles, triazoles, imidazoles, and dihydropyrimidines, has been explored recently for its potential biological applications [2,3,4,7,8,9,10]. Among Fc derivatives, compounds exhibiting antitumor, antiviral, antimalarial, antibacterial, antioxidant, and antifungal activities have been identified.

Despite these advances in many areas of therapeutics, the application of Fc derivatives as agents for neurodegenerative disease treatment, particularly Alzheimer’s disease (AD), has been less thoroughly investigated. Given the critical role of oxidative stress in AD pathogenesis, Fc’s reversible oxidation capability, which can convert the electron-donating Fc group into an electron-deficient ferrocenium cation (Fc^+^), is an attractive property [1,6]. In addition, certain Fc derivatives have shown remarkable antiaggregating effects against β-amyloid [10,11,12]. Moreover, examples of Fc derivatives that inhibit cholinesterases are available, demonstrating moderate to strong inhibitory activity against acetylcholinesterase (AChE) and butyrylcholinesterase (BChE) [13,14], as well as selective BChE inhibition [15,16].

Additionally, Fc moieties are frequently seen as efficient carriers [1], that can cross the blood–brain barrier (BBB), enhancing targeted drug delivery to the brain [5].

Nitrogen-based heterocyclic compounds have been widely adopted in the development of AD drugs [17,18,19,20]. These include derivatives of pyridine [21,22,23,24], pyridazine [25,26], bipyridine [24,27], quinoline [28,29,30,31,32], and acridine [33], among other heterocycles.

On the theme of developing multifunctional agents enhanced by the presence of an Fc moiety, combining an azinyl group, a redox-active Fc scaffold, and a highly conjugated two-dimensional π-electron system of an olefin fragment within one molecule offers a promising route for developing compounds with notable antioxidant properties [34,35,36,37]. Several foundational methods have been developed for synthesizing olefin-containing Fcs. For instance, metal-catalyzed alkenylation via C-H bond activation allows for the creation of planar chiral Fc olefins [38,39,40]. The Wittig reaction, which involves phosphonium salts reacting with aldehydes and ketones, is a conventional approach leading to vinylferrocenes [41,42]. The McMurry cross-coupling reaction, in the presence of Lewis acids, has been successfully employed for synthesizing analogs of the antitumor drug tamoxifen [43,44,45,46,47,48]. Another pathway for vinylferrocenes synthesis involves reducing aldehydes or ketones to the corresponding alcohols followed by dehydration [49,50,51]. Previous work from our group has established synthetic strategies for incorporating azinyl groups into metallocenes and related compounds [52,53,54,55,56,57,58], as well as proposing a regioselective acetylation approach for azinylferrocenes [59].

The main goals for the present study were to synthesize alkenyl derivatives of azinylferrocenes and evaluate their potential as anti-AD agents. The chemical diversity and biological activity of the synthesized compounds was investigated using the following approaches: (1) Calculation of Tanimoto similarity coefficients (Tc), normalized molecular volumes (Vn), and volumes of overlap of docked ligands; (2) Inhibitory potencies against AChE, BChE, and off-target carboxylesterase (CES); (3) Propidium displacement from the AChE peripheral anionic site (PAS); (4) Inhibition of β-amyloid 1–42 (Aβ_42_) self-aggregation; (5) Antioxidant activity; (7) Quantum-chemical (QC) predictions of protonation, dimerization, and structure-antioxidant relationships; (8) Enzyme kinetics; (9) Molecular docking with protein targets generated by an AlphaFold3 reproduction code; and (10) Cytotoxicity of lead compounds in cell cultures.

## 2. Results and Discussion

### 2.1. Synthesis and Characterization of Products

Compounds **1a-e** were synthesized following the procedure described by Chupakhin [52], with compound 1-(pyridazin-4-yl)ferrocene (**1e**) obtained in a yield of 53% (Table 1).

**Scheme 1:**
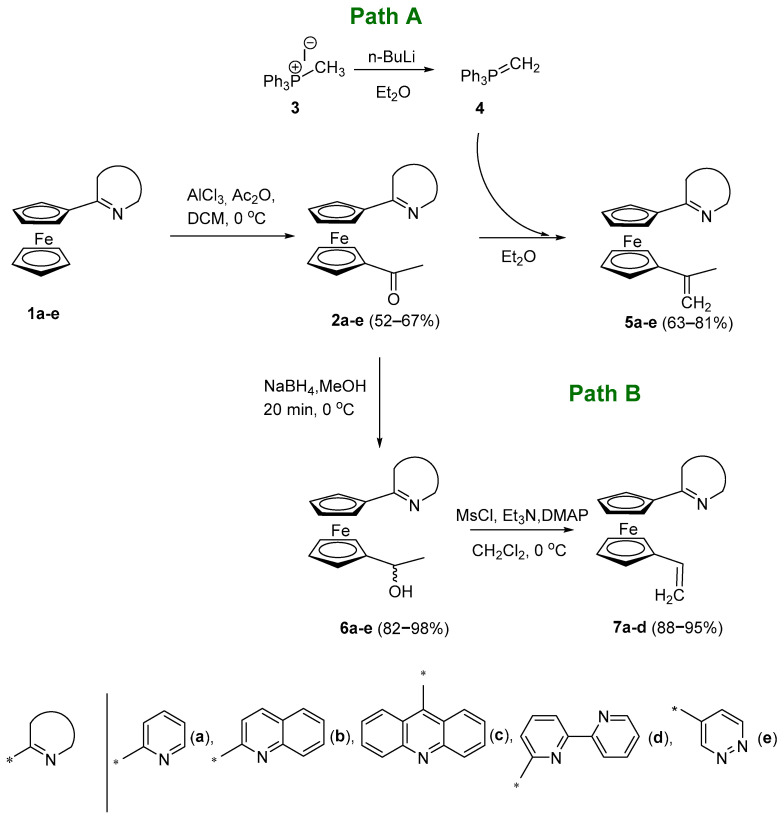
Synthesis of alkenylferrocenes **5a-e** and **7a-e**.

1-Azinyl-1′-acetylferrocenes **2a-e** were produced via the established Friedel-Crafts acetylation protocol using Ac_2_O and AlCl_3_ (Scheme 1), yielding satisfactory results as shown in Table 1 and described in [59]. Next, these 1-azinyl-1′-acetylferrocenes **2a-e** were subjected to a Wittig reaction at room temperature under an argon atmosphere for 4 h using ylide **4**, generated in situ from triphenylmethylphosphonium iodide **3** in presence of *n*-BuLi (Scheme 1, Path A). This reaction resulted in the formation of 1-azinyl-1′-isopropenylferrocenes **5a-e** in good yields [59]. Additionally, reducing acetylferrocenes **2a-e** with NaBH_4_ in methanol (MeOH) at 0 °C yielded alcohols **6a-e** within 20 min and with high efficiency (Scheme 1, Path B). When NaBH_4_ was added, the color of the reaction mass changed from red-brown to orange-yellow. The synthesized compounds **6a-e** were isolated using flash column chromatography on SiO_2_.

It was observed that the synthesized 1-(α-hydroxyethyl)-1′-azinylferrocenes **6a-e** could undergo dehydration in CH_2_Cl_2_ in an inert atmosphere for 5 h at 0 °C in the presence of Et_3_N as a base, catalytic amounts of 4-dimethylaminopyridine, and methanesulfonyl chloride (MsCl). This reaction successfully afforded the desired vinylferrocenes (**7a-d**) with up to 95% yield. The synthesized compounds **7a-d** were isolated using flash column chromatography on Al_2_O_3_. However, it was not possible to isolate derivative **7e** due to the formation of a mixture of unidentifiable compounds.

In the ^1^H NMR spectra for **6a-e**, Fc proton signals appeared between δ 3.90–5.08 ppm. There was an upfield shift for the CH_3_-group proton signal (from δ 2.03–2.30 ppm to δ 1.23–1.36 ppm), along with a one-proton doublet at δ 1.76–5.32 ppm and a one-proton quartet or multiplet at δ 4.30–4.57 ppm, which corresponded to the OH and CH groups, respectively. For compounds **7a-d**, the ^1^H NMR spectra showed characteristic signals for 1,1′-disubstituted ferrocenes at δ 4.11–5.01 ppm, with additional proton signals for heterocyclic fragments between δ 7.03–9.06 ppm. Notably, the ^1^H NMR spectra of **7a-d** displayed a singlet corresponding to the CH group of the double bond at δ 5.35–6.17 ppm, and CH_2_-group signals at δ 4.85–5.38 ppm.

In the ^13^C NMR spectra for **6a-e**, no C=O signal was observed, while a CH group signal appeared at δ 65.10–65.65 ppm. The IR spectra for **6a-e** featured a characteristic absorption band at ν = 3416–3312 cm^−1^, indicating valence vibrations of the alcohol fragment, along with vibrations corresponding to secondary alcohols (ν = 1100–1038 cm^−1^). Mass spectra of derivatives **6a-e** and **7a-d** showed a peak for the molecular ion.

We developed a high-yield method for synthesizing azinyl-containing alkenylferrocenes. Additionally, isopropenylferrocene (**8**) [60] and vinylferrocene (**9**) [51] were synthesized through established methods to facilitate comparative analyses of biological properties.

Quantum chemical (QC) calculations (see Supplemental Information, Section S4; Table S1) predicted that under the conditions used to assess the endpoints summarized in Table 2, the Fc derivatives with pyridine, bipyridine, quinolone and acridine ligands would be fully or partially protonated, while those with pyridazine ligands would be neutral. In addition, QM calculations predicted that the Fc derivatives with pyridine, quinoline, or acridine ligands would display varying degrees of dimer formation stabilized by a HPO_4_^2−^ anion in phosphate buffer for all endpoints in Table 2 except propidium displacement, for which the dimers would be stabilized by two chloride anions in Tris-HCl buffer.

**Figure 1:**
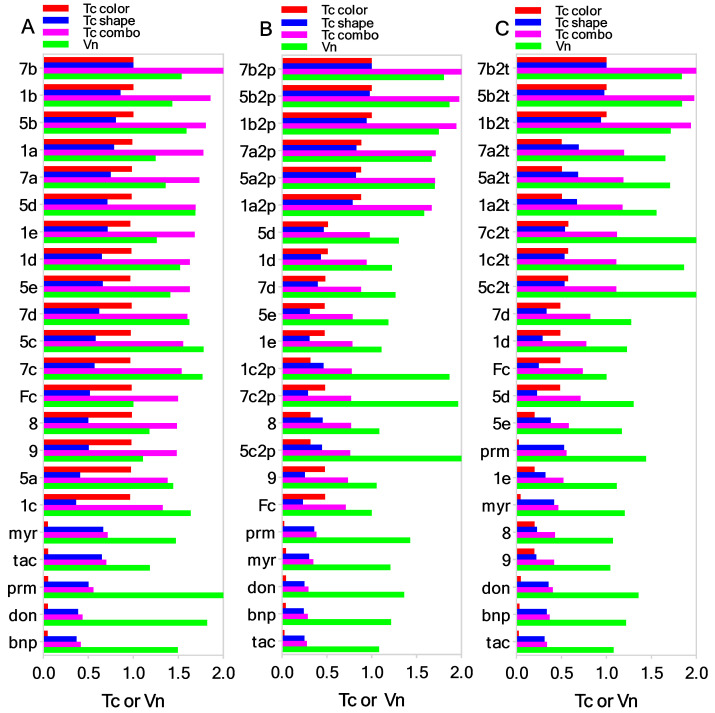
Quantification of chemical diversity of Fc derivatives and reference compounds according to Tanimoto similarity coefficients (Tc) relative to lead compound **7b** and van der Waals volumes (Vn) normalized to the largest-volume compound in each group. (**A**) Monomers, as listed in Table 2. (**B**). Dimers in phosphate buffer (“2p” suffix); otherwise, monomers. (**C**) Dimers in Tris-HCl buffer (“2t” suffix); otherwise, monomers. Tc color = similarity based on optimized overlap volume of pharmacophores (acceptor, donor, anion, cation, ring, and hydrophobe) in 3D-aligned structures; range = (0, 1). Tc shape = similarity based on optimized overlap volume of shape in 3D-aligned structures; range = (0,1). Tc combo = Tc shape + Tc color; range = (0,2). Vn range = (0,2). In each panel, compounds are ranked by Tc combo values. Reference compounds: bnp, bis-4-nitrophenyl phosphate (BNPP); don, donepezil; myr, myricetin; prm, propidium; tac, tacrine).

The chemical diversity of the Fc derivatives and reference compounds was quantified in two ways: (1) Calculating their Tanimoto similarity coefficients (Tc) based on 3D alignments of shape and pharmacophores relative to the lead compound **7b**; and (2) Calculating normalized van der Waals volumes (Vn) relative to the compound with the largest volume in each group. These results are shown in Figure 1.

Note that in Figure 1A for the monomeric forms of the molecules, the compound with the largest volume was propidium. In contrast, when the dimeric forms of the molecules were considered, the acridine Fc derivatives **5c2p**, **7c2p**, and **1c2p** (Figure 1B) and **5c2t**, **7c2t**, and **1c2t** (Figure 1C) displayed the largest volumes.

When ranking the compounds by their Tc combo scores relative to the **7b** monomer (Figure 1A), **7b2p** dimer (Figure 1B), or **7b2t** dimer (Figure 1C), the quinoline derivatives were grouped together either as monomers or dimers, the pyridine derivatives were grouped together as dimers, and the acridine derivatives were grouped together as dimers in Tris-HCl buffer. As expected, the reference compounds (BNPP, donepezil, myricetin, propidium, and tacrine) exhibited extremely low Tc color values, reflecting their lack of pharmacophoric similarity to the Fc-containing molecules.

### 2.2. Biological Investigations

#### 2.2.1. Inhibition Studies of *Hs*AChE, *Ec*BChE, and *Ss*CES. Structure-Activity Relationships

For all synthesized azinylferrocenes (azinyl-Fc) **1a-e**, **5a-e**, **7a-d**, and their basic pharmacophores—Fc (**Fc**), 1-alkenylferrocenes: **8** (Fc–C(CH_3_)=CH_2_, isopropenylferrocene) and **9** (Fc––CH=CH_2_, vinylferrocene)—the esterase profile was evaluated. These determinations and analyses indicate the relative capability of the compounds to inhibit three serine esterases: acetylcholinesterase (AChE, EC 3.1.1.7) and butyrylcholinesterase (BChE, EC 3.1.1.8)—both cholinergic targets—as well as carboxylesterase (CES, EC 3.1.1.1), a structurally related off-target enzyme. This profiling allows for the assessment of primary pharmacological effects based on AChE and BChE inhibition, alongside potential adverse effects due to CES inhibition, as CES is known to hydrolyze many ester-containing drugs [61,62,63].

In these experiments, human (*Homo sapiens*) erythrocyte AChE (*Hs*AChE), equine (*Equus caballus*) serum BChE (*Ec*BChE), and porcine (*Sus scrofa*) liver CES (*Ss*CES) were used because of their ready availability, relatively low cost, and high sequence homology of *Ec*BChE and *Ss*CES to the corresponding human enzymes [62].

Although the dominant carboxylesterase in porcine liver is CES1 [64], the preparation sourced from Sigma-Aldrich is not a purified or recombinant CES1. Therefore, we have designated the preparation used in our experimental studies as *Ss*CES. However, in computational molecular docking studies, it is necessary to choose a single defined protein as the docking target. Thus, the logical choice for our computational studies of CES was the most abundant and best characterized isoform found in porcine liver, which is *Ss*CES1.

The selection of tacrine, an effective inhibitor of both AChE and BChE, and bis-4-nitrophenyl phosphate (BNPP), a selective CES inhibitor, as reference compounds, helped contextualize the activity of the test compounds.

As shown in Table 2, across the three groups of Fc derivatives, compounds with quinoline (**1b**, **5b**, **7b**) and bipyridine (**1d**, **5d**, **7d**) substituents demonstrated effective inhibition of BChE within the micromolar range. Bipyridine derivatives were also found to be potent AChE inhibitors across all groups—compounds **1d**, **5d**, and **7d**. Quinoline derivatives exhibited significant anti-AChE activity in the isopropenyl-Fc (**5b**) and vinyl-Fc (**7b**) groups, with 10 times less activity observed for compound **1b**, which lacked an alkenyl substituent.

All compounds studied displayed minimal inhibition of CES, a structurally similar enzyme to the other esterases in Table 2, but functionally distinct by being responsible for hydrolyzing a wide range of ester-containing drugs. This low CES inhibition suggests a lower risk of adverse drug interactions during potential clinical applications [61].

#### 2.2.2. Kinetic Studies of *Hs*AChE and *Ec*BChE Inhibition

**Figure 2:**
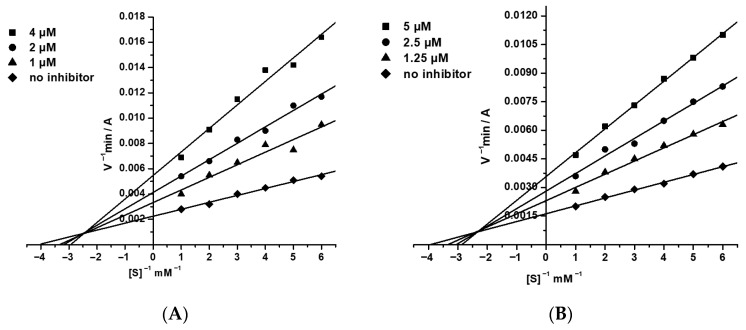
Steady state inhibition of *Hs*AChE (**A**) and *Ec*BChE (**B**) by compound **7b**.

The mechanism of *Hs*AChE and *Ec*BChE inhibition by the synthesized compounds was further examined with the most active compound **7b**. To determine the relationship between enzyme activity and inhibitor concentration at different substrate levels, we conducted kinetic assays. The kinetic data analysis, using Lineweaver–Burk plots, is shown in Figure 2A and Figure 2B for *Hs*AChE and *Ec*BChE, respectively.

It can be seen that when compound **7b** binds to *Hs*AChE and *Ec*BChE, *V*_max_ and *K*_m_ both change, which is typical for a mixed type of inhibition. The competitive (*K*_i_) and noncompetitive (α*K*_i_) components of inhibition constant by compound **7b** were 1.92 ± 0.13 µM and 3.05 ± 0.18 µM for *Hs*AChE and 2.77 ± 0.22 µM and 4.58 ± 0.27 µM for *Ec*BChE.

#### 2.2.3. Molecular Docking of Fc Derivatives and Reference Compounds into *Hs*AChE

As shown in Table 2, the lead compound **7b**, a quinoline derivative, potently inhibited *Hs*AChE, while compound **7c**, an acridine derivative, was a poor inhibitor of this esterase. To gain mechanistic insight into these contrasting inhibitory behaviors, molecular docking into *Hs*AChE was performed with the monomeric and dimeric forms of these compounds in separate virtual experiments.

**Figure 3:**
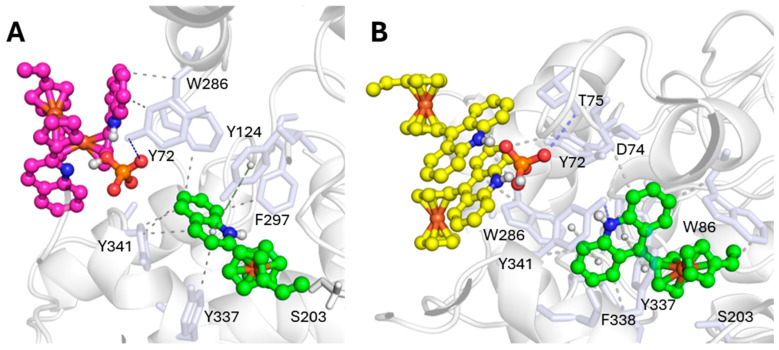
Molecular docking of Fc derivatives to *Hs*AChE. (**A**) Compound **7b** phosphate-stabilized dimer (carbon atoms colored magenta) and monomer (carbon atoms colored green). (**B**) Compound **7c** phosphate-stabilized dimer (carbon atoms colored yellow) and monomer (carbon atoms colored green). Ligands are depicted in ball-and-stick format. Protein secondary structure is shown as a semi-transparent light-gray ribbon. Protein residues forming contacts with ligands are displayed as semi-transparent light-blue sticks and labeled. Nonpolar hydrogen atoms in ligands and protein residues are hidden for clarity. Hydrogen bonds are dark-blue dashed lines. Dashed lines connecting small light-gray dummy atoms within aromatic rings are π-stacking interactions. Hydrophobic interactions are dashed gray lines. Docking of the monomers and dimers was carried out separately. Each panel is an overlay of the results for the monomer and dimer for ease of comparison of the relative positions of the two ligand species.

As can be seen in Figure 3, the dimers of both **7b** and **7c** bind at the mouth of the *Hs*AChE gorge. Residue Y72 forms a hydrogen bond with a phosphate oxygen in dimeric **7b**, and Y72 and W286 have hydrophobic interactions with one of the quinoline rings in dimeric **7b** (Figure 3A). The phosphate group in dimeric **7c** accepts a hydrogen bond from T75 and one of the acridine rings in **7c** has hydrophobic interactions with Y72 and W286 (Figure 3B).

In contrast to the peripheral binding of the dimers, the monomers of **7b** and **7c** bind within the catalytic active site (Figure 3). The quinoline ring in the **7b** monomer has a π-T interaction with residue Y124 and hydrophobic interactions with W286, F297, and Y337. The **7c** monomer forms a hydrogen bond with Y341, π-T stacking interactions with Y337 and Y341, and hydrophobic interactions with D74, W86, F338, and Y341.

Both the **7b** and **7c** monomers had an Fc carbon atom within 4.5 Å of the CB carbon of the active site S203 residue, which is shown as a reference point (Figure 3A,B), but these proximities were not counted as hydrophobic interactions due to the 4.0 Å cutoff distance that was employed.

Experimental enzyme inhibition studies (Table 2) demonstrated that **7b** is a potent inhibitor of *Hs*AChE, while **7c** is not. These relative inhibitory potencies, coupled with the docking poses obtained for the monomeric and dimeric forms of **7b** and **7c** (Figure 3), indicate that the monomer is the dominant species for **7b** and the dimer is the dominant species for **7c**.

It is apparent from visual inspection of the docking poses in Figure 3 that the monomeric forms of each of the ligands do not overlap with their respective dimeric forms. However, this case-by-case qualitative analysis can be extended to all possible pairs of a set of docked ligands and quantified by computing a matrix of their normalized molecular intersection volumes expressed as percent overlap.

**Figure 4:**
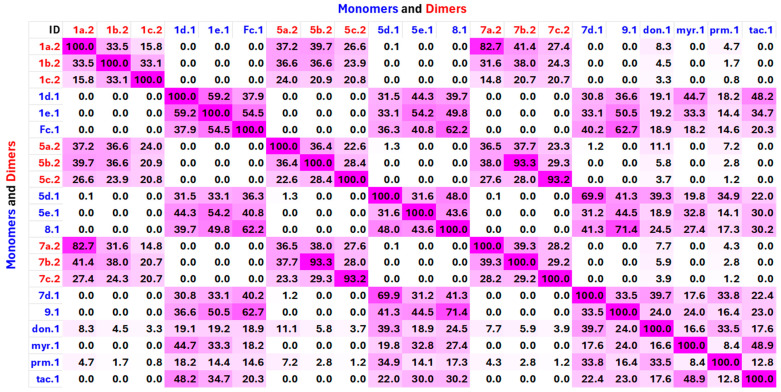
Heatmap of percent overlap for all possible pairs of a set of docked ligands in *Hs*AChE. Ligand names with “.1” extensions indicate monomers and “.2” extensions indicate dimers. Scale: 0 = no shading; 100 = dark magenta. Substantial overlap occurs between dimer-dimer or monomer-monomer pairs, while minimal or no overlap occurs between monomer-dimer pairs. Because this heatmap is a similarity matrix, the diagonal elements are all 100%.

Figure 4 shows such a matrix as a heatmap of percent overlap for a set of 21 ligands docked into *Hs*AChE. The ligands included the following molecules: dimeric forms for the pyridine, quinoline, and acridine Fc derivatives; monomeric forms for the bipyridine and pyridazine Fc derivatives; the three types of parent Fc moieties, and four reference compounds. It can readily be seen from the shaded blocks that the dimers have substantial overlap with dimers, and monomers have substantial overlap with monomers. Likewise, the unshaded or lightly shaded blocks show that dimers tend to have minimal or no overlap with monomers. Because this heatmap is a similarity matrix, the elements on the diagonal are all 100%.

**Figure 5:**
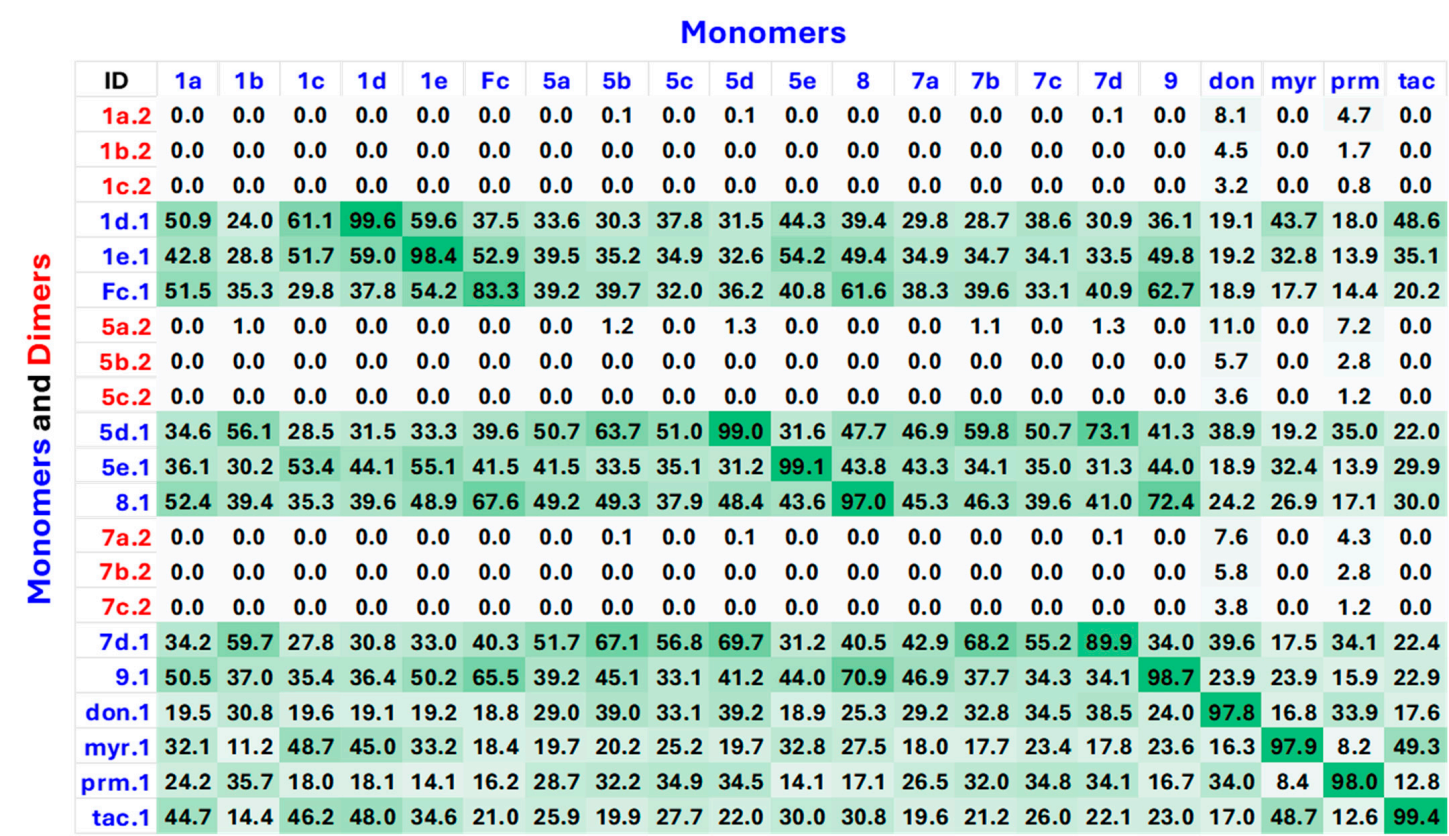
Mixed heatmap of percent overlap for all possible pairs of two sets of docked ligands in *Hs*AChE. The first set consists of monomers; the second set includes monomers (with a name extension of “.1” and dimers (with a name extension of “.2). Scale: 0 = no shading; 100 = dark green. Because this heatmap is not a similarity matrix, the diagonal elements are not 100%. The mixed format allows visualization of overlap between monomeric and dimeric forms of a given ligand.

Figure 5 is a mixed heatmap showing the percent overlap between two sets of ligands docked into *Hs*AChE. One set consists of all monomers; the other set includes monomers and dimers. Thus, unlike Figure 4, which is a similarity matrix, Figure 5 is not a similarity matrix; therefore, the elements on the diagonal are not 100%. The mixed heatmap allows visualization of the degree to which a monomeric form of a ligand overlaps with the corresponding dimeric form of the ligand. For example, the monomers **7b** and **7c** have zero overlap with their corresponding dimers, **7b.2** and **7c.2**, respectively, in agreement with visual inspection of the docking poses in Figure 3. On the other hand, the monomeric form of compound **7b** has 32.8% and 21.2% overlap with donepezil and tacrine, respectively, known inhibitors of *Hs*AChE, which is consistent with the relatively high inhibitory potency of compound **7b** against *Hs*AChE reported in Table 2 and in agreement with the conclusion that the dominant form of this molecule is monomeric.

#### 2.2.4. Molecular Docking of Fc Derivatives and Reference Compounds into *Ec*BChE

**Figure 6:**
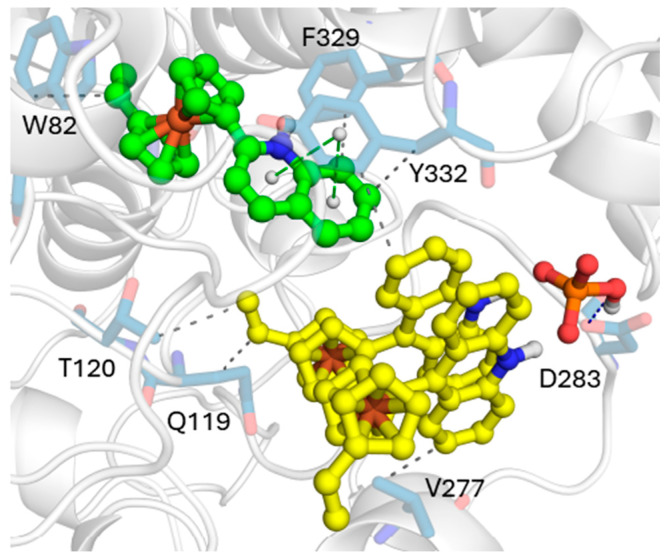
Molecular docking of Fc derivatives into *Ec*BChE. Monomeric **7b** (carbon atoms colored green) binds to the catalytic active site, while dimeric **7c** (carbon atoms colored yellow) binds to the mouth of the gorge. Protein secondary structure is shown as a semi-transparent light-gray ribbon. Residues interacting with ligands are shown as semi-transparent light-blue sticks and labeled. Ligands are in ball-and-stick format. Nonpolar hydrogen atoms in ligands and protein residues are hidden for clarity. Hydrogen bonds are dark-blue dashed lines. Dashed lines connecting small light-gray dummy atoms within aromatic rings are π-stacking interactions. Hydrophobic interactions are dashed lines. Dockings of the monomer and dimer were carried out separately. Results for the monomer and dimer were combined for ease of comparison of the relative positions of the two ligand species.

Figure 6 shows the **7b** monomer and **7c** dimer docked into *Ec*BChE. As with *Hs*AChE, the **7b** monomer binds to the catalytic active site, and the **7c** dimer binds to the peripheral rim of the gorge.

In the catalytic active site of *Ec*BChE, the quinoline ring of monomeric **7b** forms parallel π-stacking interactions with residue Y332 as well as hydrophobic interactions with residues F329 and Y332. In addition, the Fc moiety of **7b** has a hydrophobic interaction with residue W82 (Figure 6).

On the peripheral rim of the *Ec*BChE gorge, the phosphate group in the **7c** dimer forms a hydrogen bond with residue D283, while the 1′-vinyl group on one of the Fc moieties has hydrophobic interactions with Q119. One of the acridine rings also has a hydrophobic interaction with V277 (Figure 6).

The heatmaps of percent overlap of ligands in *Ec*BChE were similar to those for human AChE. However, in the case of *Ec*BChE, the dimeric form of compound **1c** exhibited overlaps ranging from 17.8% to 24.3% with monomeric species **1d**, **5d**, **7d**, **donepezil**, and **propidium** (Figures S7.1 and S7.2). 

#### 2.2.5. Molecular Docking of Fc Derivatives and Reference Compounds into *Ss*CES1

**Figure 7:**
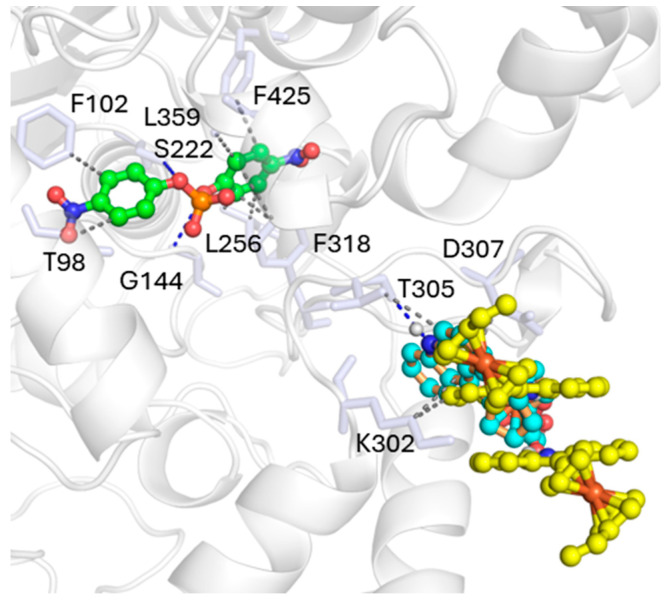
Molecular docking of **BNPP** and **7c** into *Ss*CES1. **BNPP** (carbon atoms colored green) binds to the catalytic active site, whereas both the monomeric form (carbon atoms colored cyan) and dimeric form (carbon atoms colored yellow) of compound **7c** bind near the surface of the enzyme. Protein secondary structure is shown as a semi-transparent light-gray ribbon. Residues interacting with ligands are shown as semi-transparent light-blue sticks and labeled. Ligands are in ball-and-stick format. Hydrogen bonds are dark-blue dashed lines. Hydrophobic interactions are dashed lines. Nonpolar hydrogen atoms in ligands and protein residues are hidden for clarity. Docking of the three ligands was carried out separately. The results were combined for ease of comparison of the relative positions of the ligand species. Note that the docking poses of the monomeric and dimeric forms of compound **7c** overlap.

As shown in Table 2, **BNPP** was capable of potently inhibiting *Ss*CES, but none of the Fc derivatives was an effective inhibitor of this enzyme. Consistent with the experimental results, molecular docking showed that **BNPP** binds to the catalytic active site of *Ss*CES1, but both the monomeric and dimeric forms of Fc derivatives were found to bind near the enzyme surface, as illustrated in Figure 7 for **BNPP** and the monomeric and dimeric forms of compound **7c**.

As can be seen in Figure 7, **BNPP** forms extensive interactions with the active site residues of *Ss*CES1. These contacts include hydrogen bonds from G144 and S222 to phosphoryl oxygens of **BNPP** as well as hydrophobic interactions with T98, F102, L256, F318, L359, and F425.

The monomeric form of compound **7c** entered into interactions with only three *Ss*CES1 residues: hydrophobic interactions with K302 and D307, along with a hydrophobic interaction and a hydrogen bond with T305. The dimeric form of **7c** exhibited only a single *Ss*CES1 contact—a hydrophobic interaction with K302. Note that the docking poses of the monomeric and dimeric forms of compound **7c** overlap with each other (Figure 7).

Heatmaps of percent overlap of docked ligands in *Ss*CES1 differed markedly from those for *Hs*AChE and *Ec*BChE by showing fewer substantial overlaps (Figures S7.7 and S7.8). There were no overlaps between **BNPP** and other ligands except **Fc** (29.6%) and **9** (34.6%)—this might account for the 4.2% and 23.4% inhibition of *Ss*CES that was produced by a 20 μM concentration of these compounds (Table 2).

#### 2.2.6. Displacement of Propidium from the *Ee*AChE PAS

To assess the potential of the synthesized compounds as inhibitors of the proaggregating activity of AChE, a fluorescence-based assay was employed to evaluate their ability to competitively displace propidium [65,66,67]. Propidium, a selective ligand of the AChE peripheral anionic site (PAS), is known to effectively inhibit AChE-induced β-amyloid aggregation [68].

As shown in Table 2, at a concentration of 20 µM, quinoline derivatives (**1b**, **5b**, **7b**), acridine derivatives (**1c**, **5c**, **7c**) and bipyridine derivatives (**1d**, **5d**, **7d**) from all three Fc groups, along with compounds **7a** containing a pyridine substituent, were able to displace propidium. Their level of displacement was comparable to that of the reference compound, donepezil (11.9 ± 0.9%), indicating that these compounds are able to bind to AChE PAS and may act as blockers of AChE-induced β-amyloid aggregation [68,69,70].

Taking the displacement level of donepezil as 100% and expressing the displacement level of the active Fc compounds as a percentage of the donepezil value, the mean ± SEM of the 10 Fc derivatives that had non-zero displacement values was 84.1 ± 3.33%. The Fc compounds that had displacement values of zero were **1a**, **1e**, **Fc**, **5a**, **5e**, **8**, and **9**. From this analysis, it appeared reasonable to hypothesize that the ability of a compound to displace propidium from the *Ee*AChE PAS could be treated as an all-or-nothing phenomenon, suggesting a simple binary classification of compounds into active and inactive categories. Furthermore, it also seemed reasonable that the ability of a compound to displace propidium from the *Ee*AChE PAS would depend upon two principal variables: (1) the compound partially occupying the same volume otherwise filled by the propidium molecule; and (2) the binding energy (−ΔG, where ΔG = free energy of binding) of the compound to the *Ee*AChE PAS. These two variables are readily obtained from the results of molecular docking, described in the next section.

#### 2.2.7. Molecular Docking of Fc Derivatives and Reference Compounds into *Ee*AChE

**Figure 8:**
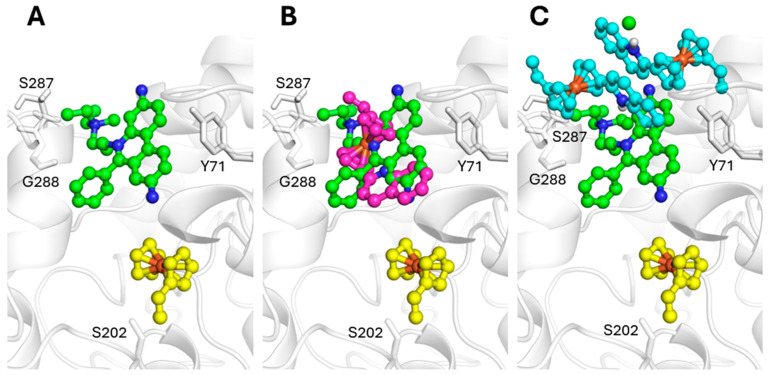
Molecular docking of compounds into *Ee*AChE. (**A**) **Propidium** (carbon atoms colored green) and **9**. (**B**) **Propidium** and **9** with overlay of the monomeric form of **7b** (carbon atoms colored magenta). (**C**) **Propidium** and **9** with overlay of the dimeric form of **7b** (carbon atoms colored cyan); a chloride anion is shown as a noncovalently bound green ball near the protonated nitrogen in one of the quinoline rings of the **7b** dimer. Protein secondary structure is shown as a semi-transparent light-gray ribbon. For clarity and orientation, only a few amino acid residues are shown as light gray sticks and labeled; protein-ligand interactions are not shown but described in detail in the text. Ligands are in ball-and-stick format. Except for the dimeric form of **7b** for which polar hydrogens are shown, hydrogen atoms in ligands and protein residues are hidden for clarity. Docking of the four ligands was carried out separately; results were combined for ease of comparison of the relative positions of the ligand species. Note that the docking poses of both the monomeric and dimeric forms of compound **7b** overlap with **propidium**—these ligands bind to the PAS, while compound **9** binds to the CAS near the active site S202 residue.

Figure 8 shows the results for molecular docking of propidium, compound **9**, monomeric compound **7b** and dimeric compound **7b** into *Ee*AChE. Because the propidium displacement experiments were carried out in Tris-HCl buffer, the dimeric form of compound **7b** stabilized by chloride anions was used for docking rather than the dimeric form stabilized by a phosphate anion as was done for docking into *Hs*AChE, *Ec*BChE, *Ss*CES1, and *Hs*Aβ_42_.

In Figure 8A–C, **propidium** binds to the PAS of *Ee*AChE near landmark residues S287, G288, and Y71. Not shown are extensive contacts including parallel π-stacking interactions with W281 and Y336, a hydrogen bond with D73, and hydrophobic interactions with Y71, W261, L284, F292, F333, and Y336. Compound **9** binds to the CAS of *Ee*AChE where it has only two hydrophobic interactions with a single residue, W85. In Figure 8B, propidium is overlaid with the monomeric form of compound **7b**, which has a π-T stacking interaction with Y123, two parallel π-stacking interactions with Y336, and hydrophobic interactions with Y71, D73, F333, and Y336. Figure 8C shows propidium overlaid with the dimeric form of **7b**, which has two π-T interactions with W281 and hydrophobic interactions with Y71, W281, and L282.

The percent overlap heatmaps for reference compounds as well as the monomeric and dimeric forms of the Fc derivatives docked into *Ee*AChE are shown in Figures S7.5 and S7.6. The data in these matrices are especially important for understanding and making predictions about propidium displacement from the *Ee*AChE PAS by the study compounds. In this regard, it should be noted that significant differences exist between the heatmaps for *Hs*AChE and *Ee*AChE, particularly with respect to the overlap patterns between propidium and monomeric vs. dimeric forms of the Fc derivatives. For example, in *Hs*AChE, propidium overlaps were greater with monomers than with dimers when molecular docking was carried out with phosphate-stabilized dimers of the a, b, and c types. However, when all compounds were docked as monomers into *Hs*AChE, the propidium overlaps were more evenly distributed across the range of compounds (Figure 4 and Figure 5).

In contrast, when molecular docking was conducted against *Ee*AChE with chloride-stabilized dimers of the **a**, **b**, and **c** types of compounds with the other types docked as monomers, the overlaps with propidium were markedly higher for the dimeric Fc compounds. However, when docking was performed with all ligands as monomers, propidium overlaps were low for 12 of the 21 compounds (0–4.1%), and higher for **1b**, **5b**, **7b**; **5c**, **7c**; **5d**, **7d**; and **donepezil** (11.4–35.3%) (Figures S7.3 and S7.4).

**Figure 9:**
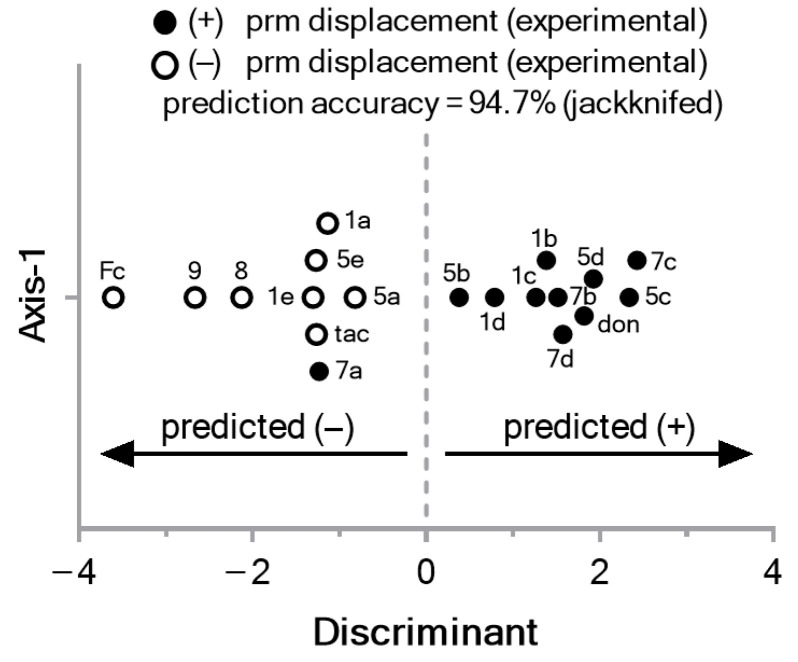
Linear discriminant analysis (LDA) of molecular docking data as a binary classifier to predict active (closed circles) and inactive (open circles) compounds for propidium displacement from the PAS of *Ee*AChE. Prediction accuracy was 94.7% with or without applying an exhaustive leave-one-out (statistical jackknifing) procedure. One compound (**7a**) out of 19 was misclassified as inactive when it was actually active.

To test the hypothesis that information gleaned from molecular docking, namely, propidium overlap volumes and binding energies, could be combined to predict the ability of compounds to displace propidium from the PAS of *Ee*AChE, binary classification models were constructed using linear discriminant analysis (LDA). To do this, the “ground truth” classifications were assigned based on the experimental results, where a displacement based on the reference compound, donepezil, was taken as 100%. On this basis, a compound that had a displacement value > 50% was considered active. The inactive Fc compounds each had a displacement value of zero. The only reference compound other than donepezil for which there was an experimentally determined value was tacrine, which scored 26%; accordingly, this compound was deemed inactive. When running the models, the ground truth classifications and the propidium displacement data were not included in the analysis. The only data used were the propidium overlap volumes and affinities (binding energies) obtained from molecular docking. After the LDA predictions were made, the ground truth experimentally determined classifications were used to assess the prediction accuracy. Figure 9 shows the results of the LDA model.

By using only two variables obtained from molecular docking (percent overlap with propidium and binding energy), LDA was able to distinguish between active and inactive compounds for propidium displacement from the *Ee*AChE PAS with 94.7% accuracy (Figure 9). One compound (**7a**) out of 19 was misclassified. Future work will determine the extent to which an LDA binary classifier is a successful predictor of propidium displacement by other classes of compounds. It is noteworthy that in this case, the best results were obtained by using the binding affinities for monomers rather than dimers. This suggests the possibility that the chloride-stabilized dimers were not as stable as the phosphate-stabilized ones, which is in agreement with the QC predictions (Table S3).

#### 2.2.8. Inhibition of *Hs*Aβ_42_ Self-Aggregation

The potential of compounds from all three groups (**1a-e**, **5a-e**, **7a-d**) to inhibit *Hs*Aβ_42_ self-aggregation was evaluated using a Thioflavin T (ThT) fluorescence assay [45].

The results, presented in Table 2, demonstrate that the studied Fc derivatives inhibited *Hs*Aβ_42_ self-aggregation within a range of 43–90%. Compounds **1** with the azinyl group displayed slightly lower activity compared to 1-azinyl-1′-isopropenyl-Fcs **5** and 1-azinyl-1′-vinyl-Fcs **7**. Maximum inhibition (71–90%) was observed in quinoline derivatives (**1b**, **5b**, **7b**), acridine derivatives (**1c**, **5c**, **7c**), and bipyridine derivatives (**5d**, **7d**), as well as compound **7a** with a pyridine substituent. These compounds inhibited *Hs*Aβ_42_ self-aggregation to a degree comparable to or surpassing that of the reference compound, myricetin (79.4 ± 6.3%). To characterize further the interactions of the study compounds with *Hs*Aβ_42_, molecular docking investigations were conducted, as described in the next section.

#### 2.2.9. Molecular Docking of Fc Derivatives and Reference Compounds to *Hs*Aβ_42_

**Figure 10:**
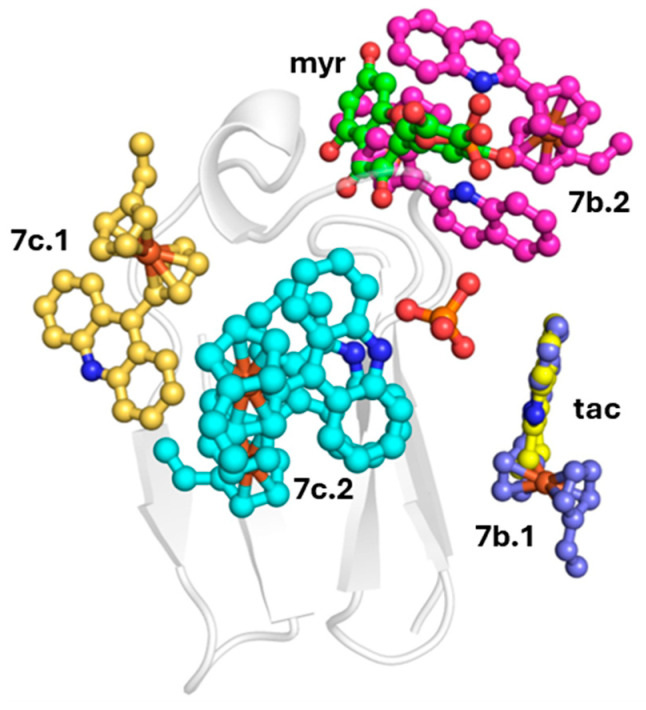
Molecular docking of selected Fc derivatives and reference compounds to *Hs*Aβ_42_. This composite of docking poses includes (clockwise from left) the monomer of **7c** (**7c.1**, carbon atoms colored dark yellow), **myricetin** (**myr**, carbon atoms green), the dimeric form of **7b** (**7b.2**, carbon atoms colored magenta), **tacrine** (**tac**, carbon atoms colored yellow), the monomer of **7b** (**7b.1**, carbon atoms colored dark blue), and the dimeric form of **7c** (**7c.2**, carbon atoms colored cyan; note the presence of the stabilizing phosphate anion). Ligands are depicted in ball-and-stick format; all hydrogens are omitted for clarify). The peptide is shown as a semi-transparent light gray ribbon.

Molecular docking to *Hs*Aβ_42_ was performed with both the monomeric and dimeric forms of the Fc compounds as well as with the reference compounds: **donepezil**, **myricetin**, **propidium**, and **tacrine**; the results are summarized in Figure 10 and the text below.

Figure 10 illustrates the three distinct binding sites on *Hs*Aβ_42_ for monomers characterized by **7c.1**, **myricetin** (**myr**), and **tacrine** (**tac**)/**7b.1**. When molecular docking included the **a**, **b**, and **c** types of compounds as dimers, there were again three distinct binding sites characterized by **7c.2**, **myricetin** (**myr**)/**7b.2**, and **tacrine** (**tac**).

When monomers were employed as ligands, all compounds except **1c**, **5a**, **5c**, **7b**, **7c**, and tacrine docked to the myricetin site of *Hs*Aβ_42_, defined by contacts with residues F4, H6, D7, S8, G9, F20, A21, E22, D23, S26, N27, and K28. When the **a**, **b**, and **c** types of ligands were in dimeric form, all compounds docked to the myricetin site except **1c**, **5a**, **5c**, **7c**, and tacrine. These bindings to the myricetin site can easily be read from the percent overlap heatmaps (Figures S7.5 and S7.6). 

It is noteworthy that the myricetin contacts identified here, using blind molecular docking to the entire *Hs*Aβ_42_ peptide, recapitulated residues found in other studies using molecular docking [71], molecular dynamics (MD) [72,73,74] and NMR [75]. Moreover, the present molecular docking was carried out using a predicted peptide structure using Protenix, an AlphaFold3 reproduction code [76], which generated a structure quite different from those reported from NMR studies [77,78] that were used in our previous investigations [79,80]. Our decision not to use previously published structures solved by solution NMR in the present study was influenced the fact that the NMR structures were determined in solutions that included various concentrations of the highly apolar solvent, hexafluoroisopropanol (HFIP) [77,78], known to promote helix formation. In contrast, our predicted peptide structure in the current investigation contained beta strands and loops but no helices.

However, although the majority of our compounds docked to the myricetin site, binding to this site was apparently not necessary for blocking self-aggregation of *Hs*Aβ_42_. For example, the most potent blocker of self-aggregation was compound **5c** (90.1% inhibition; Table 2). This compound, as well as **1c** and **7c**, which were all predicted to exist mainly in dimeric form, docked congruently to the same site on *Hs*Aβ_42_, defined by residues Y10, L17, V18, F19, A21, E22, D23, and V24. No other compounds docked to this site.

The tacrine binding site on *Hs*Aβ_42_ included residues A2, E3, F4, E11, and F20. Tacrine itself was a poor inhibitor of *Hs*Aβ_42_ self-aggregation (5.9%; Table 2). Among other study compounds, only the monomeric forms of **1c**, **7b**, and **7c** docked to the tacrine site, but in the phosphate buffer used for the self-aggregation assays, the dimeric forms of these compounds were likely favored. The dimeric form of compound **5a** docked to the tacrine site, but this compound also made contacts with residues in the myricetin site, and the potency of **5a** was relatively modest at 62.4% (Table 2).

**Figure 11:**
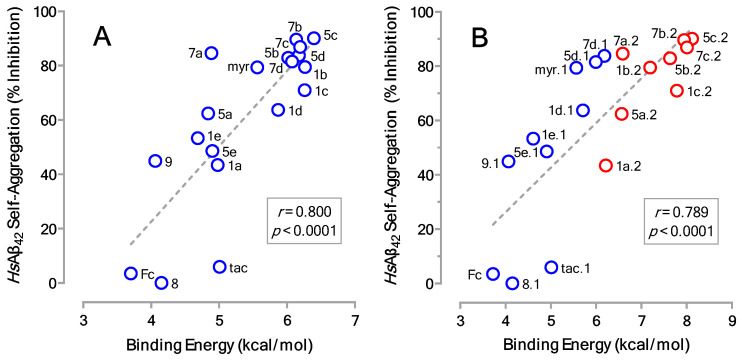
Correlations of *Hs*Aβ_42_ self-aggregation (% inhibition) from experiment vs. binding energy (kcal/mol) from molecular docking. (**A**) All ligands are monomers (open blue circles labeled with compound names). (**B**) Ligands are monomers (open blue circles with compound names and “.1” extensions) or dimers (open red circles with compound names and “.2” extensions). Dashed lines are least-squares best fit lines for orientation. Pearson correlation coefficients (*r*) and associated *p*-values are shown in boxes.

Whereas the binding locus on a peptide or protein is a vital determinant of the biological function of a ligand, the potency of the ligand also depends on its binding energy [81]. As noted above in the case of propidium displacement by ligands from the *Ee*AChE PAS, there was an apparent binary response that prompted prediction from a combination of propidium overlap volume and binding energy using LDA (Table 2; Figure 9). In contrast, inhibition of *Hs*Aβ_42_ self-aggregation exhibited a graded response over a wide range from zero to 90.1% (Table 2), and although correlations with myricetin overlap volumes were not significant (*p* = 0.86 for monomers; *p* = 0.25 for dimers), there were strong correlations with the binding energies obtained from molecular docking of the monomers (*r* = 0.800, *p* < 0.0001) and dimers (*r* = 0.789; *p* < 0.0001), as shown in Figure 11. Although the *r*-value for the monomers was slightly higher than the value for the dimers, the difference between them was not statistically significant. Moreover, based on the QC predictions for the compounds in phosphate buffer, the expectation is that there would be a monomer-dimer equilibrium among the Fc derivatives, with dimers predominating in the following order: acridines > quinolines > pyridines (see Supplemental Information, Section S5).

#### 2.2.10. Antioxidant Activity (AOA)

The primary antioxidant activity of the newly synthesized azinyl-Fcs (**1a-1e**), azinyl- isopropenyl-Fcs (**5a-5e**), azinylvinyl-Fcs (**7a-7d**) and their basic pharmacophores **Fc** and 1-alkenyl-Fcs **8** and **9** was evaluated using two standard assays. The first is the ABTS test, which measures the ability of a compound to scavenge the model radical cation ABTS^•+^. The second is the Ferric Reducing Antioxidant Power (FRAP) test, which assesses the ability of a compound to reduce a particular Fe^3+^ complex to the Fe^2+^ state. The results of these assays, which are displayed in Table 3, provide insight into the antioxidant efficacy of the compounds based on their radical-scavenging and iron-reducing capacities.

Table 3 illustrates that azinyl-Fcs exhibit significantly higher primary antioxidant activity compared to Fc. All the azinyl-Fc derivatives tested displayed high antioxidant activity, matching or surpassing the standard antioxidant Trolox in both the ABTS and FRAP tests.

Among all tested groups, quinoline derivatives demonstrated the highest activity across both assays. In the ABTS test, quinoline derivatives were up to four times more active than Trolox, while in the FRAP test, they showed activity levels 2–3 times greater than Trolox. Conversely, the bas—ic pharmacophores—Fc, isopropenyl-Fc **8**, and vinyl-Fc **9**, —did not exceed Trolox’s antioxidant activity.

#### 2.2.11. QC Analyses of Antioxidant Activity (AOA)

To investigate trends in antioxidant activity (AOA), representatives of all three series of the synthesized Fc derivatives with varied substituents on the second cyclopentadienyl (Cp) rings of Fc were examined: Fc (**Fc**) and compounds **1a**, **1b**, and **1c**, each containing different azinyl substituents of varying sizes (pyridine, quinoline, and acridine) on a single Cp ring. Isopropenyl-Fc **8** and derivatives **5a**, **5b**, **and 5c**, with an iso-propenyl substituent on one Cp ring and the same azinyl substituents on the other. Vinyl-Fc **9** and derivatives **7a**, **7b**, and **7c**, incorporating a vinyl substituent on one Cp ring and the azinyl substituents on the other. In all three series, a significant increase in AOA was observed when a heterocyclic aromatic substituent was added to the second Cp ring.

QC calculations (see Supplementary Information for details) indicated that, under the FRAP test conditions, all compounds containing an aromatic heterocyclic substituent are protonated at the nitrogen atom of this substituent. For the ABTS test, compounds with a pyridine substituent (**1a**, **5a**, **7a**) showed partial protonation at the nitrogen atom, while those with quinoline (**1b**, **5b**, **7b**) and acridine (**1c**, **5c**, **7c**) substituents were fully protonated at the heterocyclic nitrogen atom. Compounds with the protonated nitrogen atom are marked with the subscript “p” for clarity, and all antioxidant properties were calculated based on the protonation states relevant to the experimental conditions.

To evaluate AOA in the FRAP test, both ionization potential (IP) and bond dissociation enthalpy (BDE) values in water were calculated (see Table 4). It was found that compounds lacking azinyl substituents (**Fc**, **8**, **9**) have relatively similar and low IP values, about 74.5 kcal/mol. However, the BDE values for these compounds varied widely, ranging from 88.1 kcal/mol (compound **8**) to 111.6 kcal/mol (**Fc**). Given the similar and moderate AOA of these compounds in the FRAP test (0.8–1.1 TE) a single electron transfer (SET) mechanism is likely responsible for their antioxidant action.

In contrast to non-substituted Fc derivatives, protonated compounds with azinyl substituents (**1a**_p_, **1b**_p_, **1c**_p_, **5a**_p_, **5b**_p_, **5c**_p_, **7a**_p_, **7b**_p_, **7c**_p_) exhibit higher IP values (about 80 kcal/mol) and substantially lower BDE values (67–69 kcal/mol). The observed increase in AOA upon the addition of azinyl substituents indicates an alternative mechanism, where AOA is determined by BDE values. Taking into account that Fe^3+^[TPTZ]_2_ is reduced to Fe^2+^[TPTZ]_2_ during the antioxidant reaction and that AOA correlates with BDE values, a concerted mechanism can be assumed. In this mechanism, the antioxidant molecule loses a hydrogen atom, with the electron being transferred to the cation Fe^3+^[TPTZ]_2_ and the proton being transferred to the solvent molecule. A similar mechanism is realized for phenolic antioxidants in the FRAP test [82].

The lowest AOA of acridine-based compounds likely stems from the greater propensity of acridine compounds to dimerize [83]. QC calculations support this, showing that dimer formation increases with increasing ligand size in the series **1a**_p_, **1b**_p_, **1c**_p_ (with pyridine, quinolone and acridine substituents, respectively) (see Supplementary Information, Section S5, Table S3).

For AOA evaluation in the ABTS test, IP and BDE values were calculated in ethanol (refer to Table 4). QC analyses indicated that changes in BDE and IP values in ethanol followed the same trends observed in water, aligning with similar AOA results in the FRAP and ABTS assays. The mechanism of antioxidant action of each compound is the same in both FRAP and ABTS tests. Compounds without azinyl substituents (**Fc**, **8**, **9**) showed relatively low IP values (about 82 kcal/mol, see Table 4) and reduced the ABTS^•+^ radical through the SET mechanism.

In contrast, protonated compounds with azinyl substituents (**1a**_p_, **1b**_p_, **1c**_p_, **5a**_p_, **5b**_p_, **5c**_p_, **7a**_p_, **7b**_p_, **7c**_p_) have low BDE values (67–69 kcal/mol, Table 4) and operate via the alternative mechanism similar to that in the FRAP test. In this process, an electron transfers to the ABTS^•+^ radical while a proton is simultaneously transferred to the SO_4_**^2−^** anion in the solvent. Similar mechanism was realized in the ABTS test with conjugates of amiridine and salicylic acid derivatives [80]. The change in the AOA mechanism explains the significant increase in AOA with the addition of azinyl substituents.

QC calculations further revealed that, in the ABTS test, compounds with a pyridine substituent are only partially protonated, while compounds with quinoline and acridine substituents are fully protonated. This protonation difference explains the relatively lower TEAC values of pyridine-based compounds compared to quinoline-based ones. The decreased TEAC values observed for acridine-based compounds are attributed to their strong tendency toward dimerization, consistent with results obtained in the FRAP test (see Supplementary Information, Section S5, Table S3).

#### 2.2.12. Cytotoxicity of Fc Derivatives

The cytotoxicity of lead compounds—**1b**, **1d**, **5b**, **5d**, **7b**, **7d** as well as reference compounds **10** and **11** [45]— was assessed using reprogrammed human neuronal cell cultures (MSC-Neu) (see Supplementary Information) and human neuroblastoma cell cultures (SH-SY5Y). Mesenchymal stem cells for reprogramming into a neuronal phenotype and SH-SY5Y cells were obtained from the “Vertebrate Cell Culture Collection”supported by the Ministry of Education and Science of the Russian Federation (Agreement No. 075-15-2021-683). Previously studied compounds **10** and **11** [45] were selected as reference compounds for cytotoxicity assessment as the most structurally similar to ferrocifen type anticancer agents [84,85]. Cytotoxic activity was assessed using the MTT test [86]. The range of compound concentrations was from 1 to 1024 μM and the incubation time was 24 h. For each compound, the half-lethal concentration parameter LC_50_ (μM) was calculated. The results are presented in Table 5.

**Figure 12:**
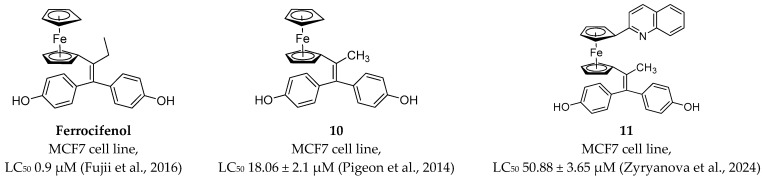
Ferrocifen-type compounds used as references in the MTT assay [45,87,88].

The results showed that the study compounds generally exhibited relatively low cytotoxicity, being substantially less toxic than the control ferrocifen-type compounds (Figure 12), with a more pronounced cytotoxic effect on human neuroblastoma tumor cells (SH-SY5Y) compared to neuronal cells (MSC-Neu).

## 3. Materials and Methods

### 3.1. Chemistry

The dehydration reactions and Wittig reactions were conducted under an argon atmosphere using standard Schlenk techniques [89]. ^1^H-NMR (600 MHz) and ^13^C-NMR (151 MHz) spectra were recorded on a Bruker AVANCE NEO NMR spectrometer, while ^1^H-NMR (400 MHz) and ^13^C-NMR (100 MHz) spectra were recorded on a Bruker AVANCE II NMR spectrometer. Chemical shifts are reported in δ values (ppm) using tetramethylsilane (TMS) as the internal standard, with CDCl_3_ and DMSO-d_6_ as solvents.

High-resolution electrospray ionization mass spectra were obtained for positive ions within the m/z range of 50–1300 Da on a Bruker maXis Impact HD high-resolution Q-TOF LC-MS/MS spectrometer. Samples were prepared in methanol and analyzed after partial chromatographic separation on a ZORBAX SB-C18 column (2.1 × 50 mm, 1.8 μm, 40 °C, methanol-water 9:1, 0.25 mL/min flow rate). The mass scale was calibrated using a calibration solution of HCOONa (10 mmol) in a water-propan-2-ol mixture (1:1).

IR spectra were recorded on a Perkin Elmer Spectrum One B Fourier-transform infrared spectrometer equipped with a diffuse reflection attachment. Melting points were determined using a Boetius heating stage.

Preparative column chromatography was performed on Merck silica gel 60 (particle size 0.063–0.200 mm, Darmstadt, Germany) and Macherey-Nagel aluminum oxide 90 neutral (Düren, Germany). Reaction progress was monitored using thin-layer chromatography (TLC) on 0.2 mm aluminum oxide plates with a fluorescent indicator (Polygram^®^ Alox N/UV254, Macherey-Nagel, Düren, Germany) and 0.20 mm silica gel plates coated with fluorescent indicator F254 (ALUGRAM^®^ Xtra SIL G/UV254, pre-coated TLC sheets, Macherey-Nagel, Düren, Germany).

All solvents were purified according to standard procedures. Pyridazine, methanesulfonyl chloride, 4-(dimethylamino)pyridine, sodium tetrahydroborate, acetic anhydride, aluminum chloride, *n*-BuLi, Fc, acetylferrocene were purchased from Aldrich. Starting methyl(triphenyl)phosphonium iodide [90], bromoferrocene [91] azinylferrocenes **1a-d** [52], isopropenylferrocene **8** [60], vinylferrocene **9** [51], compounds **2a-d** and **5a-d** [59], **10** [88] and **11** [45] were prepared according to the published procedures.

#### 3.1.1. Synthesis of 1-(Pyridazin-4-yl)Ferrocene 1e

To a freshly prepared suspension of lithioferrocene, obtained from the solution bromoferrocene (7.58 mmol, 2 g) in dry diethyl ether (15 mL) и from a 1.6 M *n*BuLi solution in hexane (7.1 mL, 11.3 mmol) in Schlenk tube under an atmosphere of argon at 0 °C within 30 min, added pyridazine solution (15.16 mmol, 1.1 mL) in dry diethyl ether. Then, reaction mixture was heating at 40 °C for 2 h. The product was obtained by column chromatography on Al_2_O_3_ using a mixture of hexane:EtOAc (1:1) as an eluent. The eluate was concentrated to dryness in vacuo and the residue was recrystallized from hexane.

Orange solid. Yield of **1e** 1.06 g (53%). M.p. 147 °C. ^1^H NMR (600 MHz, CDCl_3_): *δ—*4.09 (s, 5H, Cp), 4.52 (s, 2H, C_5_H_4_), 5.08 (s, 2H, C_5_H_4_), 7.35 (s, 1H, 5′-H), 7.51 (s, 1H, 3′-H), 8.99 (s, 1H, 6′-H) ppm. ^13^C NMR (151 MHz, CDCl_3_): *δ—*67.6 (C-Cp); 69.9 (CH-Cp); 70.9 (C-Cp′); 80.2, 123.5, 126.1, 148.9, 162.2 (pyridazine) ppm. IR (*ν*, cm^−1^): 3085, 2961, 2857, 1787, 1668, 1581, 1484, 1415, 1377, 1327, 1288, 1258, 1110, 1006, 887, 793, 746, 707, 514. HRMS (ESI) *m/z* calcd for C_14_H_13_FeN_2_^+^ [M+H]^+^: 265.0422; found 265.0423.

#### 3.1.2. Synthesis of 1-Acetyl-1′-(Pyridazin-4-yl)Ferrocene 2e

Acetic anhydride (3 mmol, 0.37 mL) was added to the solution of 1-(pyridazin-4-yl)ferrocene (1 mmol) **3e** in dry CH_2_Cl_2_ (20 mL) at 0 °C, then for 2 h AlCl_3_ (3 mmol) was added in equal portions approximately every 15 min. The reaction was stirring for 24 h, then the reaction mass was poured into cold water and extracted with CH_2_Cl_2_ (3 ×25 mL). The extract was dried over Na_2_SO_4_, decanted and concentrated at reduced pressure. The residue was purified by flash column chromatography on Al_2_O_3_ (hexane:EtOAc 7:3, *R*_f_ = 0.1). The eluate was concentrated to a dry state in vacuo.

Red oil. Yield of **2e**: 177 mg (58%). M.p. 88 °C. ^1^H NMR (600 MHz, CDCl_3_): *δ—*2.12 (s, 3H, CH_3_); 4.41–4.42 (m, 2H, C_5_H_4_); 4.52 (m, 2H, C_5_H_4_); 4.66–4.67 (m, 2H, C_5_H_4_); 5.08 (m, 2H, C_5_H_4_); 7.41–7.46 (m, 2H, 3′-H, 5′-H); 9.03 (d, 1H, *J* = 3 Hz, 6′-H) ppm. ^13^C NMR (151 MHz, CDCl_3_): *δ—*27.6 (CH_3_); 68.9, 71.1, 72.2, 73.8 (CH-Cp); 80.6, 82.2 (C-Cp); 123.9, 126.3, 149.4, 160.1 (pyridazine); 201.6 (C=O) ppm. IR (*ν*, cm^−1^): 2924, 1660, 1582, 1487, 1455, 1375, 1278, 1116, 889, 818, 750, 620, 538, 522, 501. HRMS (ESI) *m/z* calcd for C_16_H_15_FeN_2_O^+^ [M+H]^+^: 307.0528; found 307.0527.

#### 3.1.3. General Procedure for Formation of 1-Isopropenyl-1′-(Pyridazin-4-yl)Ferrocene 5e

1.6 M *n*-BuLi solution in hexane (0.71 mL, 1.14 mmol) was added to a suspension of methyltriphenylphosphonium iodide **3** (1.25 mmol) in dry diethyl ether in a Schlenk tube under argon at room temperature. After 30 min stirring, a solution of 1-acetyl-1′-(pyridazin-4-yl)ferrocene **2e** (1 mmol) in anhydrous diethyl ether (20 mL) was added. After stirring at room temperature for 24 h, reaction mixture was purified by flash column chromatography on Al_2_O_3_ (hexane:EtOAc 7:3, *R*_f_ = 0.25). The eluate was concentrated to dryness in vacuo.

Red solid. Yield of **5e**: 191 mg (63%). M.p. 90 °C. ^1^H NMR (600 MHz, DMSO-*d_6_*): *δ—*1.72 (s, 3H, CH_3_), 4.15 (t, 2H, *J* = 2.0 Hz, C_5_H_4_), 4.32 (t, 2H, *J* = 2.0 Hz, C_5_H_4_), 4.47 (t, 2H, *J* = 2.0 Hz, C_5_H_4_), 4.62 (s, 1H, =CH_2_), 4.94 (s, 1H, =CH_2_), 5.04 (t, 2H, *J* = 2.0 Hz, C_5_H_4_), 7.57 (br. s, 1H, 5′-H), 7.75 (s, 1H, 3′-H), 8.98 (br.s, 1H, 6′-H) ppm. ^13^C NMR (151 MHz, DMSO-*d_6_*): *δ—*20.9 (CH_3_); 67.0, 68.1, 70.0, 71.3 (CH-Cp); 81.3, 87.2 (C-Cp); 109.5 (CH_2_); 124.1, 126.2, 149.0, 160.1 (pyridazine); 139.5 (C) ppm. IR (*ν*, cm^−1^): 3075, 2918, 1665, 1662, 1579, 1486, 1375, 1288, 1216, 1181, 1112, 1081, 1020, 987, 885, 815, 746, 586, 517. HRMS (ESI) *m/z* calcd for C_17_H_16_FeN_2_^+^ [M+H]^+^: 304.0657; found 304.0656.

#### 3.1.4. Synthesis of 1-Azinyl-1′-(α-Hydroxyethyl)Ferrocenes 6a-e (General Procedure)

To a solution of 1-azinyl-1′-acetylferrocene **2a-e** (1 mmol) in MeOH (15 mL), solid NaBH_4_ (5 mmol) was added in one portion at 0 °C. The reaction mixture was stirred at 0 °C for 20 min. Formed solution was poured into ice-cold water (20 mL), then extracted with ethyl acetate (3 × 30 mL). The collected organic layers were washed with brine (100 mL), dried over Na_2_SO_4_, decanted, solvent was removed under reduced pressure to afford the crude product. Isolated alcohols **6a-e** were purified by flash column chromatography on SiO_2_. The eluate was concentrated to dryness in vacuo.

##### 1-(Pyridin-2-yl)-1′-(α-Hydroxyethyl)Ferrocene (**6a**)

Using 312 mg (1 mmol) 1-(pyridin-2-yl)-1′-acetylferrocene and 190 mg (5 mmol) NaBH_4_, 1-(pyridin-2-yl)-1′-(α-hydroxyethyl)ferrocene was obtained as orange crude oil in 93% yield (285 mg, 0.928 mmol). *R*_f_ = 0.4 (TLC: hexane:EtOAc, 9:1). ^1^H NMR (600 MHz, CDCl_3_): *δ—*1.32 (d, 3H, *J* = 6.5 Hz, CH(OH)CH_3_), 3.90 (m, 1H, C_5_H_4_), 3.96 (m, 1H, C_5_H_4_), 4.10–4.11 (m, 1H, C_5_H_4_), 4.15–4.16 (m, 1H, C_5_H_4_), 4.38–4.39 (m, 1H, C_5_H_4_), 4.44–4.45 (m, 1H, C_5_H_4_), 4.56 (q, 1H, *J* = 6.5 Hz, CH(OH)CH_3_), 4.80–4.83 (m, 2H, C_5_H_4_), 5.29 (s, 1H, CH(OH)CH_3_), 7.10–7.13 (m, 1H, 5′-H), 7.30–7.31 (d, 1H, *J* = 7.0 Hz, 3′-H), 7.57–7.60 (td, 1H, *J* = 7.0 Hz, *J* = 2.0 Hz, 4′-H), 8.53–8.54 (m, 1H, 6′-H) ppm. ^13^C NMR (151 MHz, CDCl_3_): *δ—*25.5 (CH_3_); 65.5 (CH); 67.2, 67.3, 68.2, 68.3, 69.6, 69.9, 70.2 (CH-Cp); 84.8, 96.9 (C-Cp); 120.5 (C3), 121.3 (C5), 136.7 (C4), 149.5 (C6), 159.0 (C2) ppm. IR (ν, cm^−1^): 3086, 2968, 2924, 2857, 1720, 1588, 1564, 1499, 1425, 1368, 1278, 1151, 1100, 1024, 924, 875, 758, 742, 698, 624, 521. HRMS (ESI) *m/z* calcd for C_17_H_18_FeNO^+^ [M+H]^+^: 308.0732; found 308.0731.

##### 1-(Quinolin-2-yl)-1′-(α-Hydroxyethyl)Ferrocene (**6b**)

Using 362 mg (1 mmol) 1-(quinolin-2-yl)-1′-acetylferrocene and 190 mg (5 mmol) NaBH_4_, 1-(quinolin-2-yl)-1′-(α-hydroxyethyl)ferrocene was obtained as orange solid in 98% yield (350 mg, 0.980 mmol). *R*_f_ = 0.5 (TLC: hexane:EtOAc, 9:1). M.p. 79–81 °C. ^1^H NMR (600 MHz, CDCl_3_): *δ—*1.34 (d, 3H, *J* = 6.5 Hz, CH(OH)CH_3_), 3.93 (d, 2H, *J* = 12.0 Hz, C_5_H_4_), 4.14 (d, 2H, *J* = 25.0 Hz, C_5_H_4_), 4.50 (d, 2H, *J* = 30.0 Hz, C_5_H_4_), 4.56 (q, 1H, *J* = 6.4 Hz, CH(OH)CH_3_), 5.02 (d, 2H, *J* = 18.0 Hz, C_5_H_4_), 5.32 (s, 1H, CH(OH)CH_3_), 7.45 (d, 1H, *J* = 8.5 Hz, 4′-H), 7.48 (t, 1H, *J* = 7.6 Hz, 7′-H), 7.70 (t, 1H, *J* = 7.6 Hz, 6′-H), 7.75 (d, 1H, *J* = 7.5 Hz, 5′-H), 8.05 (d, 1H, *J* = 8.5 Hz, 3′-H), 8.10 (d, 1H, *J* = 7.5 Hz, 8′-H) ppm. ^13^C NMR (151 MHz, CDCl_3_): *δ*—25.4 (CH_3_); 65.3 (CH); 67.6, 68.22, 68.24, 68.30, 68.33, 70.0, 70.4, 70.6 (CH-Cp); 84.4, 97.1 (C-Cp); 119.3 (C4); 125.9 (C7); 126.9 (C8a); 127.6 (C5); 128.5 (C8); 130.3 (C6); 136.5 (C3); 148.1 (C4a); 159.8 (C2) ppm. IR (*ν*, cm^−1^): 3082, 2964, 1616, 1594, 1557, 1508, 1442, 1422, 1376, 1245, 1142, 1125, 1092, 1003, 826, 747, 519. HRMS (ESI) *m/z* calcd for C_21_H_20_FeNO^+^ [M+H]^+^: 358.0888; found 358.0891.

##### 1-(Acridin-9-yl)-1′-(α-Hydroxyethyl)Ferrocene (**6c**)

Using 406 mg (1 mmol) 1-(acridin-9-yl)-1′-acetylferrocene and 190 mg (5 mmol) NaBH_4_, 1-(acridin-9-yl)-1′-(α-hydroxyethyl)ferrocene was obtained as red solid in 96% yield (390 mg, 0.958 mmol). *R*_f_ = 0.4 (TLC: hexane:EtOAc, 1:1). M.p. 59–60 °C. ^1^H NMR (600 MHz, CDCl_3_): *δ—*1.36 (d, 3H, *J* = 6.0 Hz, CH(OH)CH_3_), 1,76 (s, 1H, CH(OH)CH_3_), 4,18 (s, 2H, C_5_H_4_), 4.25 (s, 1H, C_5_H_4_), 4.30 (s, 1H, C_5_H_4_) 4.57 (q, 1H, *J* = 6.4 Hz, CH(OH)CH_3_), 4.66 (s, 2H, C_5_H_4_), 4.90 (s, 2H, C_5_H_4_), 7.51–7.53 (m, 2H, 3′-H, 6′-H), 7.73–7.75 (m, 2H, 2′-H, 7′-H), 8.22 (d, 2H, *J* = 9.0 Hz, 4′-H, 5′-H), 9.05 (d, 2H, *J* = 8.8 Hz, 1′-H, 8′-H) ppm. ^13^C NMR (151 MHz, CDCl_3_): *δ*—24.0 (CH_3_); 65.6 (CH); 67.9, 68.0, 69.6 (2C), 71.0, 71.1, 73.9, 74.0 (CH-Cp); 81.8, 95.3 (C-Cp); 124.7 (C3, C6); 125.5 (C4a,C5a); 127.5 (C1, C8); 129.7 (C2, C7); 130.2 (C4,C5); 143.6 (C8a, C9a); 149.0 (C9) ppm. IR (*ν*, cm^−1^): 3091, 2969, 2922, 1723, 1621, 1553, 1447, 1400, 1259, 1038, 912, 871, 827, 760, 672, 638, 484. HRMS (ESI) *m/z* calcd for C_25_H_22_FeNO^+^ [M+H]^+^: 408.1045; found 408.1045.

##### 1-(2,2′-Bipyridin-6-yl)-1′-(α-Hydroxyethyl)Ferrocene (**6d**) 

Using 383 mg (1 mmol) 1-(2,2′-bipyridin-6-yl)-1′-acetylferrocene and 190 mg (5 mmol) NaBH_4_, 1-(2,2′-bipyridin-6-yl)-1′-(α-hydroxyethyl)ferrocene was obtained as orange solid in 98% yield (376 mg, 0.979 mmol). *R*_f_ = 0.45 (TLC: hexane:EtOAc, 6:4). M.p. 60–61 °C. ^1^H NMR (600 MHz, CDCl_3_): *δ—*1.23 (d, 3H, *J* = 6.5 Hz, CH(OH)CH_3_), 3.19 (s, 1H, CH(OH)CH_3_), 3.99–4.00 (m, 1H, C_5_H_4_), 4.05–4.06 (m, 1H, C_5_H_4_), 4.11–4.12 (m, 2H, C_5_H_4_), 4.30 (q, 1H, *J* = 6.4 Hz, CH(OH)CH_3_), 4.46–4.48 (m, 2H, C_5_H_4_), 4.98–4.99 (m, 1H, C_5_H_4_), 5.07–5.08 (m, 1H, C_5_H_4_), 7.31–7.33 (m, 1H, 5″-H), 7.39 (dd, 1H, *J* = 8.0 Hz, *J* = 1.0 Hz, 5′-H), 7.73 (t, 1H, *J* = 8.0 Hz, 4′-H), 7.87 (td, 1H, *J* = 8.0 Hz, *J* = 1.0 Hz, 4″-H), 8.26 (dd, 1H, *J* = 8.0 Hz, *J* = 1.0 Hz, 3′-H), 8.52 (d, 1H, *J* = 8.0 Hz, 6″-H), 8.67–8.69 (m, 1H, 3″-H) ppm. ^13^C NMR (151 MHz, CDCl_3_): *δ*—24.5 (CH_3_); 65.1 (CH); 67.5, 67.6, 67.8, 67.9, 68.6, 68.8, 70.2, 70.3 (CH-Cp); 84.2, 96.9 (C-Cp); 118.6 (C3); 120.1 (C5); 121.3 (C6′); 123.9 (C4′); 137.1 (C4); 137.6 (C4); 149.2 (C3′); 155.9 (C2′); 156.1 (C2); 158.4 (C6) ppm. IR (*ν*, cm^−1^): 3085, 2968, 2924, 2855, 1970, 1733, 1666, 1563, 1491, 1426, 1368, 1311, 1258, 1230, 1155, 1091, 923, 903, 870, 821, 781, 743, 679, 474. HRMS (ESI) *m/z* calcd for C_22_H_21_FeN_2_O^+^ [M+H]^+^: 385.0997; found 385.0998.

##### 1-(Pyridazin-4-yl)-1′-(α-Hydroxyethyl)Ferrocene (**6e**)

Using 307 mg (1 mmol) 1-acetyl-1′-(pyridazin-3-yl)ferrocene and 190 mg (5 mmol) NaBH_4_, 1-(pyridazin-3-yl)-1′-(α-hydroxyethyl)ferrocene was obtained as a red crude oil in 82% yield (253 mg, 0.821 mmol). *R*_f_ = 0.3 (TLC: EtOAc). M.p. 73–74 °C. ^1^H NMR (400 MHz, CDCl_3_): *δ—*1.32 (d, 3H, *J* = 6.3 Hz, CH(OH)CH_3_), 3.60 (s, 1H, CH(OH)CH_3_), 4.01 (s, 2H, C_5_H_4_), 4.10 (d, 2H, *J* = 10.0 Hz, C_5_H_4_), 4.46–4.53 (m, 3H, C_5_H_4_, CH(OH)CH_3_), 4.99 (s, 2H, C_5_H_4_), 7.33–7.36 (m, 1H, 5′-H), 7.47 (br.s, 1H, 4′-H), 8.99 (s, 1H, 6′-H) ppm. ^13^C NMR (100 MHz, CDCl_3_): *δ—*24.9 (CH_3_); 65.3 (CH); 67.6, 67.8, 68.1, 68.9 (2C), 69.0, 70.9, 71.0 (CH-Cp); 80.8, 97.1 (C-Cp); 123.7 (C5), 126.4 (C4), 149.1 (C6), 162.3 (C3) ppm. IR (*ν*, cm^−1^): 3099, 2973, 2925, 1581, 1486, 1401, 1325, 1289, 1227, 1185, 1113, 1089, 983, 883, 869, 752, 711, 590, 522, 477. HRMS (ESI) *m/z* calcd for C_16_H_17_FeN_2_O^+^ [M+H]^+^: 309.0684; found 309.0682.

#### 3.1.5. Synthesis of 1-Azinyl-1′-Vinylferrocenes 7a-e (General Procedure)

Under an inert atmosphere methanesulfonyl chloride (1.05 mmol), then Et_3_N (3 mmol) were added dropwise at 0 °C to a solution of 1-azinyl-1′-(α-hydroxyethyl)ferrocene **6a-e** (1 mmol) and 4-dimethylaminopyridine (0.05 mmol) in anhydrous CH_2_Cl_2_ (10 mL) in tube Shlenk. Then, the reaction mass was heated to room temperature and stirred for 5 h. The reaction was quenched by addition of 10% solution of NaHCO_3_ (10 mL), the mixture was extracted with ethyl acetate (3 × 20 mL). Collected organic layers were washed with brine (30 mL), dried over Na_2_SO_4_, decanted, solvent was removed under reduced pressure. The residue was purified by flash chromatography on Al_2_O_3_, the eluate was concentrated to a dry state in vacuo.

##### 1-(Pyridin-2-yl)-1′-Vinylferrocene (**7a**)

Using 308 mg (1 mmol) 1-(pyridin-2-yl)-1′-(α-hydroxyethyl)ferrocene, 0.08 mL (1.05 mmol) methanesulfonyl chloride, 0.41 mL (3 mmol) Et_3_N, 6.2 mg (0.05 mmol) 4-dimethylaminopyridine, 1-(pyridin-2-yl)-1′-vinylferrocene was obtained as a red crude oil in 90% yield (261 mg, 0.903 mmol). *R*_f_ = 0.6 (TLC: hexane:EtOAc, 9:1). ^1^H NMR (600 MHz, CDCl_3_): *δ—*4.10–4.11 (m, 2H, C_5_H_4_), 4.20–4.21 (m, 2H, C_5_H_4_), 4.34–4.35 (m, 2H, C_5_H_4_), 4.85–4.87 (m, 3H, C_5_H_4_ and -CH=CH_2_), 5.17 (d, 1H, *J* = 17.0 Hz, -CH=CH_2_), 6.15 (dd, 1H, $J_{H}^{trans}$ = 17.0 Hz, $J_{H}^{cis}$= 10.0 Hz -CH=CH_2_), 7.03–7.05 (m, 1H, 5′-H), 7.35 (d, 1H, *J* = 8.0 Hz, 6′-H), 7.56–7.59 (td, 1H, *J* = 8.0 Hz, *J* = 1.8 Hz, 4′-H), 8.52 (d, 1H, *J* = 4.0 Hz, 3′-H) ppm. ^13^C NMR (151 MHz, CDCl_3_): *δ—*68.2, 68.3, 70.3, 71.2 (CH-Cp); 84.5, 84.6 (C-Cp); 120.4 (C6), 120.6 (C5), 135.9 (C4), 149.4 (C3), 158.5 (C2); 112.1 (-CH=CH_2_), 133.4 (-CH=CH_2_) ppm. IR (*ν*, cm^−1^): 3084, 3004, 2923, 2852, 1682, 1628, 1563, 1496, 1453, 1423, 1387, 1288, 1240, 1200, 1149, 1046, 1028, 985, 891, 815, 784, 739, 696, 644, 518. HRMS (ESI) *m/z* calcd for C_17_H_16_FeN^+^ [M+H]^+^: 290.0626; found 290.0628.

##### 1-(Quinolin-2-yl)-1′-Vinylferrocene (**7b**)

Using 357 mg (1 mmol) 1-(quinolin-2-yl)-1′-(α-hydroxyethyl)ferrocene, 0.08 mL (1.05 mmol) methanesulfonyl chloride, 0.41 mL (3 mmol) Et_3_N, 6.2 mg (0.05 mmol) 4-dimethylaminopyridine, 1-(quinolin-2-yl)-1′-vinylferrocene was obtained as red solid in 93% yield (316 mg, 0.932 mmol). *R*_f_ = 0.4 (TLC: hexane:EtOAc, 9:1). M.p. 59–61 °C. ^1^H NMR (600 MHz, CDCl_3_): *δ—*4.12 (s, 2H, C_5_H_4_), 4.22 (s, 2H, C_5_H_4_), 4.42 (s, 2H, C_5_H_4_), 4.84 (d, 1H, *J* = 10.0 Hz, -CH=CH_2_), 5.01 (s, 2H, C_5_H_4_), 5.17 (d, 1H, *J* = 18.0 Hz, -CH=CH_2_), 6.19 (dd, 1H, $J_{H}^{trans}$ = 18.0 Hz, $J_{H}^{cis}$ = 10.0 Hz -CH=CH_2_), 7.47 (t, 1H, *J* = 7.0 Hz, 7′-H), 7.52 (d, 1H, *J* = 9.0 Hz, 3′-H), 7.66 (t, 1H, *J* = 7.0 Hz, 6′-H), 7.74 (d, 1H, *J* = 8.0 Hz, 4′-H), 8.03–8.05 (m, 2H, 5′-H, 8′-H) ppm. ^13^C NMR (151 MHz, CDCl_3_): *δ—*68.3, 69.1, 70.4, 71.1 (CH-Cp); 84.73, 84.75 (C-Cp); 119.8 (C3), 125.5 (C7), 126.8 (C8a), 127.6 (C4), 129.1 (C5), 129.5 (C6), 135.4 (C8), 148.5 (C4a), 158.9 (C2); 112.1 (-CH=CH_2_), 133.5 (-CH=CH_2_) ppm. IR (*ν*, cm^−1^): 3054, 2919, 1614, 1594, 1551, 1506, 1420, 1378, 1324, 1259, 1240, 1125, 1087, 1010, 978, 901, 816, 755, 619, 526. HRMS (ESI) *m/z* calcd for C_21_H_18_FeN^+^ [M+H]^+^: 340.0783; found 340.0785.

##### 1-(Acridin-9-yl)-1′-Vinylferrocene (**7c**)

Using 407 mg (1 mmol) 1-(acridin-9-yl)-1′-(α-hydroxyethyl)ferrocene, 0.08 mL (1.05 mmol) methanesulfonyl chloride, 0.41 mL (3 mmol) Et_3_N, 6.2 mg (0.05 mmol) 4-dimethylaminopyridine, 1-(acridin-9-yl)-1′-vinylferrocene was obtained as a red solid in 88% yield (343 mg, 0.881 mmol). *R*_f_ = 0.5 (TLC: hexane:EtOAc, 9:1). M.p. 109–110 °C. ^1^H NMR (600 MHz, DMSO-*d_6_*): *δ—*4.29 (s, 2H, C_5_H_4_), 4.52 (s, 2H, C_5_H_4_), 4.65 (s, 2H, C_5_H_4_), 4.84 (s, 2H, C_5_H_4_), 5.03 (dd, 1H, *J* = 10.0 Hz, *J* = 1.5 Hz, -CH=CH_2_), 5.36 (dd, 1H, *J* = 18.0 Hz, *J* = 1.5 Hz, -CH=CH_2_), 6.41 (dd, 1H, $J_{H}^{trans}$ = 18.0 Hz, $J_{H}^{cis}$ = 10.0 Hz -CH=CH_2_), 7.64–7.66 (m, 2H, 3′-H, 6′-H), 7.80–7.83 (m, 2H, 2′-H, 7′-H), 8.14 (d, 2H, *J* = 9.0 Hz, 4′-H, 5′-H), 9.05 (d, 2H, *J* = 9.0 Hz, 1′-H, 8′-H) ppm. ^13^C NMR (151 MHz, DMSO-*d_6_*): *δ*—68.4, 71.2, 71.4, 74.1 (CH-Cp); 80.5, 84.4 (C-Cp); 124.6 (C4a, C5a), 124.9 (C3, C6), 127.3 (C1, C8); 129.6 (C4, C5), 129.8 (C2, C7); 143.3 (C9); 148.2 (C8a, C9a); 112.8 (-CH=CH_2_), 133.7 (-CH=CH_2_) ppm. IR (*ν*, cm^−1^): 3069, 2925, 1625, 1563, 1516, 1459, 1404, 1335, 1262, 1101, 1032, 988, 898, 851, 802, 750, 670, 641, 519. HRMS (ESI) *m/z* calcd for C_25_H_20_FeN^+^ [M+H]^+^: 390.0939; found 390.0941.

##### 1-(2,2′-Bipyridin-6-yl)-1′-Vinylferrocene (**7d**)

Using 384 mg (1 mmol) 1-(2,2′-bipyridin-6-yl)-1′-(α-hydroxyethyl)ferrocene, 0.08 mL (1.05 mmol) methanesulfonyl chloride, 0.41 mL (3 mmol) Et_3_N, 6.2 mg (0.05 mmol) 4-dimethylaminopyridine, 1-(2,2′-bipyridin-6-yl)-1′-vinylferrocene was obtained as an orange solid in 95% yield (348 mg, 0.950 mmol). *R*_f_ = 0.4 (TLC: hexane:EtOAc, 9:1). M.p. 48–49 °C. ^1^H NMR (600 MHz, CDCl_3_): *δ—*4.12 (t, 2H, *J* = 2.0 Hz, C_5_H_4_), 4.20 (t, 2H, *J* = 2.0 Hz, C_5_H_4_), 4.37 (t, 2H, *J* = 2.0 Hz, C_5_H_4_), 4.85 (dd, 1H, *J* = 11.0, 1.0 Hz, -CH=CH_2_), 4.96 (t, *J* = 2.0 Hz, 2H, C_5_H_4_), 5.17 (dd, 1H, *J* = 18.0 Hz, *J* = 1.5 Hz, -CH=CH_2_), 6.15 (dd, 1H, $J_{H}^{trans}$ = 28.0 Hz, $J_{H}^{cis}$ = 7.0 Hz -CH=CH_2_), 7.31–7.33 (m, 1H, 5″-H), 7.37 (dd, 1H, *J* = 8.0 Hz, *J* = 1.0 Hz, 5′-H), 7.71–7.73 (t, 1H, *J* = 9.0 Hz, 4′-H), 7.85 (td, 1H, *J* = 7.0, 1.7 Hz, 4″-H), 8.19 (dd, 1H, *J* = 8.0 Hz, *J* = 1.8 Hz, 3′-H), 8.58–8.59 (d, 1H, *J* = 8.0 Hz, 6″-H), 8.68–8.69 (m, 1H, 3″-H) ppm. ^13^C NMR (151 MHz, CDCl_3_): *δ—*68.3, 68.6, 70.2, 71.0 (CH-Cp); 84.7, 85.0 (C-Cp); 117.8 (C3); 120.3 (C5); 121.3 (C6′); 123.7 (C5′); 136.8 (C4), 136.9 (C4′), 149.1 (C3′), 155.4 (C2′), 156.8 (C2), 157.7 (C6); 112.1 (-CH=CH_2_), 133.5 (-CH=CH_2_) ppm. IR (*ν*, cm^−1^): 3086, 3007, 2961, 2924, 2852, 1734, 1662, 1627, 1563, 1489, 1428, 1382, 1310, 1260, 1202, 1155, 1091, 1024, 983, 898, 853, 817, 779, 743, 681, 618, 564, 538, 515, 477. HRMS (ESI) *m/z* calcd for C_22_H_19_FeN_2_^+^ [M+H]^+^: 367.0892; found 367.0892.

#### 3.1.6. Synthesis of Isopropenylferrocene 8 [60]

*n*-BuLi solution in hexane (1.6 M, 0.71 mL, 1.14 mmol) was added to a suspension of methyltriphenylphosphonium iodide **3** (0.505 g, 1.25 mmol) in dry diethyl ether in a Schlenk tube under argon at room temperature. After 30 min stirring, a solution of the acetylferrocene (0.228 g, 1 mmol) in anhydrous diethyl ether (20 mL) was added. The mixture was refluxed at 40 °C for 1 h. Then, reaction mixture was purified by chromatography on Al_2_O_3_ (hexane/EtOAc, 9:1; *R*_f_ = 0.6) as eluent. The eluate was concentrated to dryness in vacuo.

Isopropenylferrocene 8. Dark orange solid. Yield: 147 mg (65%). M.p. 77–78 °C. ^1^H NMR (600 MHz, CDCl_3_): *δ—*2.06 (s, 3H, CH_3_), 4.10 (s, 5H, C_5_H_4_), 4.22 (s, 2H, C_5_H_4_), 4.39 (s, 2H, C_5_H_4_), 4.84 (s, 1H, =CH_2_), 5.13 (s, 1H, =CH_2_) ppm. ^13^C NMR (151 MHz, CDCl_3_): *δ—*21.7 (CH_3_), 65.9, 68.7, 69.3 (CH-Cp), 86.6 (C-Cp), 108.4 (CH_2_), 141.6 (C) ppm. IR (*ν*, cm^−1^): 3093, 3080, 2973, 2941, 2917, 2848, 1705, 1672, 1407, 1386, 1332, 1291, 1260, 1148, 1102, 1029, 998, 935, 871, 837, 819, 736, 703, 686, 673, 663, 623, 593, 583, 566, 524, 514. HRMS (ESI) *m/z* calcd for C_13_H_14_Fe^+^ [M+H]^+^: 226.0439; found 226.0438.

#### 3.1.7. Synthesis of Vinylferrocene 9 [51]

To a solution of acetylferrocene (1.00 g, 4.39 mmol) in MeOH (25 mL), solid NaBH_4_ (0.834 g, 21.95 mmol) was added in five equal portions at r.t during 10 min. The reaction mixture was stirred at r.t. for 20 min. Formed solution was poured into ice-cold water (60 mL) and extracted with CH_2_Cl_2_ (3 × 60 mL). The collected organic layers were washed with brine (60 mL), dried over Na_2_SO_4_, filtrated, and solvent was removed under reduced pressure to afford the α-methylferrocenemethanol. Then, under an inert atmosphere in Schlenk flask, to a solution of α-methylferrocenemethanol. (0.959 g, 4.17 mmol) and 4-dimethylaminopyridine (25.4 mg, 0.21 mmol) in anhydrous CH_2_Cl_2_ (10 mL) was added dropwise Et_3_N (1.8 mL, 12.51 mmol) at 0 °C, followed by addition of methanesulfonyl chloride (0.34 mL, 4.38 mmol). The reaction mixture was then stirred at r.t. for 4 h. The reaction was quenched by addition of 5% solution of NaHCO_3_ (25 mL), and the mixture was extracted with CHCl_3_ (3 × 50 mL). Collected organic layers were washed with brine (50 mL), dried over Na_2_SO_4_, filtrated, and solvent was removed under reduced pressure to afford the crude product. The crude product was purified by chromatography on Al_2_O_3_ (hexane/EtOAc, 9:1; *R*f = 0.8) to afford product **9**.

Vinylferrocene 9. Orange solid. Yield: 530 mg (60%). M.p. 51–52 °C. NMR spectra are in agreement with the literature [51].

### 3.2. Biological Investigations

Experiments were performed in alignment with the standard operating procedures approved by IPAS RAS as described below.

#### 3.2.1. In Vitro Inhibition of *Hs*AChE, *Ec*BChE, and *Ss*CES1 Activities

Enzymes, substrates, and reference compounds were sourced from Sigma-Aldrich (St. Louis, MO, USA). Enzyme activity was measured spectrophotometrically according to [91] using ATCh iodide, BTCh iodide, and 4-NPA as substrates for *Hs*AChE, *Ec*BChE, and *Ss*CES, respectively. Experimental conditions included a K/Na-phosphate buffer (100 mM), at 25 °C, with pH 7.5 for AChE and BChE assays, and pH 8.0 for CES assays. Measurements were conducted on a BioRad Benchmark Plus microplate spectrophotometer (Hercules, CA, USA). Test compounds were dissolved in DMSO with a final solvent concentration of 2% (*v*/*v*) in the incubation mixture. Initial inhibition screening was conducted at 20 μM for each compound, with IC_50_ values determined for compounds showing inhibition ≥35%.

#### 3.2.2. Kinetic Study of *Hs*AChE and *Ec*BChE Inhibition. Determination of Steady-State Inhibition Constants

To investigate the mechanisms of *Hs*AChE and *Ec*BChE inhibition, enzyme kinetics were analyzed (detailed in [92]). After a 5 min equilibration at 25 °C with three different inhibitor concentrations and six substrate concentrations, residual enzyme activity was measured as described. Lineweaver–Burk plots (1/V vs. 1/[S]) were used to determine competitive (*K*_i_) and non-competitive (α*K*_i_) inhibition constants.

#### 3.2.3. Propidium Displacement Studies

A fluorescence-based assay assessed the ability of test compounds to competitively displace propidium iodide from AChE’s PAS [65,93]. Donepezil and tacrine were used as references, with electric eel AChE (*Ee*AChE, type VI-S, lyophilized powder, Sigma-Aldrich) (St. Louis, MO, USA) as the enzyme. The 3D alignment of *Ee*AChE with human AChE confirmed structural similarity [94]. *Ee*AChE (7 μM) was incubated with 20 μM test compounds in Tris-HCl buffer (pH 8.0) for 15 min, followed by addition of propidium iodide (8 μM) as described in detail in [95]. Fluorescence readings (530 nm excitation, 600 nm emission) were obtained using a FLUOStar Optima microplate reader (LabTech, Ortenberg, Germany). Displacement (%) was calculated.

#### 3.2.4. Inhibition of β-Amyloid (1–42) (*Hs*Aβ_42_) Self-Aggregation

Inhibition of *Hs*Aβ_42_ self-aggregation by test compounds was studied using the thioflavin T (ThT) fluorescence method [68,96,97] with minor modifications as described in detail in [79]. Lyophilized HFIP-pretreated *Hs*Aβ_42_ (AnaSpec Inc., Fremont, CA, USA, 0.5 mg) was dissolved in DMSO to obtain a stable 500 μM solution. The samples of 50 μM *Hs*Aβ_42_ in 215 mM Na-phosphate buffer pH 8.0 were incubated for 24 h at 37 °C in the absence or presence of 100 μM test compounds. Myricetin in the same concentration was used as a reference. After incubation, 5 μM ThT in 50 mM glycine-NaOH buffer pH 8.5 was added, and the fluorescence was measured at 440 nm (exc.) and 485 nm (emis.) with a FLUOStar Optima microplate reader. Blanks consisted of 215 mM Na-phosphate buffer, pH 8.0, 20% (*v*/*v*) DMSO or test compounds, respectively. The inhibition (%) of *Hs*Aβ_42_ self-aggregation by the test compounds was calculated.

#### 3.2.5. Antioxidant Activity (AOA)

##### ABTS Radical Cation Scavenging Activity Assay

Radical scavenging activity of the compounds was evaluated using the ABTS radical cation (2,2′-azinobis-(3-ethylbenzothiazoline-6-sulfonic acid), ABTS^•+^) decolorization assay [98], with minor modifications described in detail in [99]. by mixing 10 μL of compound solution (concentration range 1–100 μM) in DMSO with 240 μL of ABTS^•+^ working solution in ethanol (100 μM final concentration). The reduction in ABTS^•+^ absorbance was measured spectrophotometrically at 734 nm using a xMark UV/VIS microplate spectrophotometer (Bio-Rad, Hercules, CA, USA) for 1 h.

Trolox was used as a reference, and results were reported as Trolox Equivalent Antioxidant Capacity (TEAC) as the ratio of the slopes of the concentration−response curves, test compound/Trolox. The IC_50_ values for the test compounds were also determined.

##### FRAP Assay

The ferric reducing antioxidant power (FRAP) assay [100,101] modified to be performed in 96-well microplates as described in detail in [95] was used. 10 μL (0.5 mM) of the test or reference compounds were mixed with 240 μL of FRAP reagent (2.5 mL of 10 mM TPTZ (2,4,6-tris(2-pyridyl)-s-triazine) solution in 40 mM HCl, 2.5 mL of 20 mM FeCl_3_ in distilled water and 25 mL of 0.3 M acetate buffer pH 3.6); the absorbance of the mixture was measured spectrophotometrically at λ = 593 nm after a 1 h incubation at 37 °C against a blank with a SPECTROStar Nano microplate reader (BMG Labtech, Ortenberg, Germany). Trolox was used as a reference compound. The results were expressed as Trolox equivalents (TE)—the ratio of the concentrations of Trolox and the test compound resulting in the same effect on ferric reducing activity.

#### 3.2.6. Cytotoxicity Study

This study employed SH-SY5Y neuroblastoma cells and dental pulp-derived mesenchymal stem cells (MSC-DP). Cells were cultured in DMEM/F12 with 10% (*v*/*v*) FBS under standard conditions. MSC-DP were reprogrammed into neuronal-like cells (MSC-Neu) using Neurobasal medium with addition of Neuromax and 3% (*v*/*v*) FBS for 5 days. Neuronal identity was confirmed via RT-PCR analysis of key markers (i.e., β3-tubulin, NeuN, and MAP2).

RNA isolation, cDNA synthesis, and RT-PCR followed standard protocols using SYBR Green chemistry. Primer sequences are listed (Table S1), and amplicons were validated via melt curves. Fold changes were calculated using BioRad CFX Manager 3.1 software. MSC-Neu verification methods were previously reported [102].

The cytotoxic effect of Fcs was assessed using the Mossman test for cellular dehydrogenase activity (MTT test). Cytotoxicity was measured in the concentration range from 1 to 1024 μM, the incubation time was 24 h. For each compound, the half-lethal concentration parameter (LC_50_) in μM was calculated as described previously [45].

### 3.3. Statistical Analyses

#### 3.3.1. Experimental Data Presentation

All experimental tests were performed at least in triplicate in three independent experiments. Results are presented as mean ± SEM calculated using GraphPad Prism version 6.05 for Windows (San Diego CA, USA).

#### 3.3.2. Experimental Data Analyses

Plots, linear regressions, and IC_50_ values from experimental data were determined using Origin 6.1 for Windows, OriginLab (Northampton, MA, USA). The LC_50_ values in the cytotoxicity assays were calculated with GraphPad Prism 8 for Windows (GraphPad Software, Boston, MA, USA).

### 3.4. Molecular Modeling Studies

#### 3.4.1. Preparation of Ligands for Molecular Docking, Tanimoto Similarity Coefficients (Tc), and Van Der Waals Volumes (Vn)

SDF files of the reference compounds bis(4-nitrophenyl) phosphate (**BNPP**; **bnp**), **donepezil** (**don**), **myricetin** (**myr**), **propidium** (**prm**), and **tacrine** (**tac**) were downloaded from PubChem [103], loaded into YASARA-Structure 25.07.15 for Linux [104] (hereinafter referred to as YASARA), energy-minimized in water at pH 7.4 using the AMBER14 force field [105], and exported as PDB, SDF, and YASARA object (YOB) files, all in 3D format.

The X-ray crystal structure of 1-(pyrimidin-4-yl)Fc was previously determined by members of our group [52] and deposited into the Cambridge Crystallographic Data Centre [106] as code JETXUC and deposition number 282124. The CIF file of this structure was downloaded from the CCDC and imported into YASARA where the pyrimidine substituent was removed and each of the monomeric Fc derivatives shown in Table 2 were created using the 3D molecular builder tools in YASARA. Structures were energy-minimized in water at pH 7.4 using an AMBER14 force field that was parameterized in YASARA for Fe in Fc compounds, including the handling of coordinate-covalent (“dative”) bonds. Protonation states were adjusted in YASARA according to QC predictions described below. Structures were then reminimized in YASARA using the AMBER14 force field, keeping the QC-predicted protonation states intact. Reminimized structures were then exported as PDB, SDF, and YOB files, all in flexible 3D format.

Protonated dimeric forms of Fc compounds **1a**-**c**, each stabilized by an HPO_4_^2−^ anion, were predicted as described in Supplemental Information, Sections S4 and S5, subjected to geometry optimization using QC methods as described below, and saved as PDB files. A similar approach was used to generate the protonated dimeric forms of Fc compounds **1a-c**, each stabilized by two Cl^−^ anions. These dimers were subjected to QC-based geometry optimization and exported as 3D PDB files. The dimeric PDB files were loaded into YASARA for preparation of the **5a-c** and **7a-c** versions of the dimers as described above for preparation of the monomers. However, because the monomeric constituents of the dimers were not held together by covalent bonds, to keep the dimers intact during reminimization in the AMBER14 force field, the positions of all atoms except those in the propenyl and vinyl substituents were fixed. For use in Tc and Vn calculations, the dimer structures were exported as PDB files. For use as ligands in molecular docking, the protonated dimeric structures were exported as semi-rigid 3D YOB files to maintain the core dimeric structures.

#### 3.4.2. Quantum Chemical (QC) Calculations

QC calculations were performed using the DFT method in Gaussian 16 [107] and Priroda 20 [108] software. All energetics characteristics were calculated in the unrestricted B3LYP functional [109] with the 6–31++g(d,p) basis set [110] and the empirical Grimme correction [111]. The continuum SMD model [112] was used to account for solvent (water or ethanol) effects.

#### 3.4.3. Tc and Vn Calculations

Tc values relative to the monomeric and dimeric forms of lead compound **7b** were computed using ROCS 3.8.0.1 for Linux [113,114] using PDB files of the ligands as inputs.

Vn values were calculated using the Schrödinger 2025-2 utility script, phase_volCalc, after uploading the PDB monomeric or dimeric ligand files into Schrödinger Maestro and exporting them in MAE format to use as inputs [115].

#### 3.4.4. Preparation of Protein Targets for Molecular Docking

In the present investigation, the workflow involved generation of the protein targets from their amino acid sequences using Protenix, an AlphaFold3 (AF3) reproduction code [76], followed by molecular docking with the YASARA implementation of Autodock Vina [104,116,117]. This procedure enabled the preparation of protein structures of similar quality to each other that matched the protein species employed in the experimental protocols. In contrast, high-quality X-ray crystal structures were not available for all the species of proteins used in the present study. Moreover, crystal structures of proteins can have variable resolution, missing residues, and biases introduced by structural adaptations to co-crystallized ligands [118]. Whereas co-folding of proteins with ligands might have been used, thereby obviating the docking step, preliminary trials showed that co-folding resulted in dissociation of the Fc dimers into their monomeric constituents.

Sequences for *Ec*BChE, *Ee*AChE, *Hs*Aβ_42_, *Hs*AChE, and *Ss*CES1 were downloaded from UniProt [119] and imported into YASARA. Signal and/or anchor sequences determined by UniProt or NCBI-Protein [120] were deleted, and the remaining trimmed sequences were exported as FASTA files for importing into Protenix to generate the 3D structures of the proteins.

Protenix 0.5.0 [121] was installed in a conda python environment on a Steiger Dynamics Linux workstation equipped with a liquid-cooled AMD 5975WX CPU with 32 cores/64 threads, 256 GB RAM, and 2× Nvidia RTX A6000 GPUs. Each sequence was run in multiple sequence alignment (MSA) mode for 10 recycling steps and 200 sampling steps to generate 25 diffusion samples that were output as CIF files. Using the Protenix scoring function, the top-scoring model from each of the five sequences was chosen for further processing as protein targets for molecular docking.

Each of the top-scoring Protenix models was imported into YASARA, where the proteins were subjected to a docking preparation protocol in the AMBER14 force field using default settings. The protocol included setting of atom types, addition of hydrogens, and optimization of the hydrogen-bonding network [104,122].

For each of the enzyme proteins studied, a simulation cell was positioned to encompass amino acid residues known to comprise the catalytic active site as well as potential binding sites on the protein surface or lining the tunnel or gorge leading to the catalytic active site. These residues have been documented for each species either through direct determinations or by homology to the corresponding proteins in humans or other species as follows: *Ec*BChE [123,124,125,126,127,128], *Ee*AChE [129,130]; *Hs*AChE [131,132,133]; *Ss*CES1 [134]. For the *Hs*Aβ_42_ peptide, the entire structure was enclosed within the simulation cell.

With the center of the local coordinates (x, y, z) of each protein set to (0, 0, 0), the dimensions of the cuboid simulation cell (in Å) for each protein were as follows: *Ec*BChE: (20.0, 26.0, 23.0); *Ee*AChE: (25.0, 35.0, 24.0); *Hs*Aβ_42_: (74.0, 64.0, 52.0); *Hs*AChE: (27.0, 36.0, 23.0); *Ss*CES2: (26.0, 40.0, 25.0). Each protein and its accompanying simulation cell were exported as YASARA scene (SCE) files to serve as protein targets for molecular docking.

#### 3.4.5. Molecular Docking Procedure

Molecular docking was conducted by the YASARA implementation of Autodock Vina 1.2.5 [104,116,117], using as ligands each of the reference compounds along with the monomeric and dimeric Fc derivatives prepared as described above. Likewise, the protein docking targets were prepared as described above. The number of runs per ligand was 500, and the resulting docking poses were sorted by binding energy (Eb = −ΔG, where ΔG = free energy of binding). The top-scoring pose was selected for further processing in images, calculations of overlap volumes and associated heatmaps, prediction of propidium displacement from the PAS of *Ee*AChE, and correlations of binding energies to *Hs*Aβ_42_ with percent inhibition of self-aggregation of *Hs*Aβ_42_. Images of docking poses for all but one of the figures were produced with PyMOL 3.1.6.1 for Linux, Schrödinger, LLC. The docking poses depicted in Figure S8.1 were generated with YASARA 25.07.15 for Linux. Validation of the molecular docking method is described in Supplemental Information, Section S8, and illustrated in Figure S8.1.

#### 3.4.6. Calculation of Overlap Volumes for Docked Ligands

Output from molecular docking included SDF files of the docked ligand coordinates. These files were concatenated into a multi-ligand SDF file, imported into Schrödinger Maestro 2025-2 [115], and exported as a multi-ligand MAE file. The MAE file was used as the input for the utility script, phase_volCalc. To generate a similarity matrix of ligand overlap volumes, the script was configured to compare a given MAE file to itself. To generate a non-similarity matrix of ligand overlap volumes, the script was configured to compare two different MAE files containing docking coordinates. By default, the script calculates molecular volumes based on the van der Waals radii of the constituent atoms.

#### 3.4.7. Linear Discriminant Analysis (LDA) of Propidium Displacement from *Ee*AChE PAS

LDA is a supervised procedure for dimension reduction and classification that finds a linear combination of variables to maximize the distance between the means of different classes and minimizes the within-class variance [135,136]. In the present study, data used as inputs for LDA comprised two outputs from molecular docking: (1) binding energies of the docked monomeric forms of the ligands; and (2) percent volume overlap of each monomeric ligand with propidium. LDA was performed using PAST 5.22 for Windows [137]. An exhaustive leave-one-out procedure (jackknifing) was applied [138], but the classification accuracy remained the same (94.7%) with or without jackknifing. Figure 9 depicting the LDA results was rendered by GraphPad Prism version 10.6.1 for Windows, GraphPad Software, Boston, MA, USA, www.graphpad.com (accessed on 1 October 2025).

#### 3.4.8. Correlation of *Hs*Aβ_42_ Self-Aggregation % Inhibition and Docking Binding Energy

Verification of normal distributions of data by the D’Agostino and Pearson test, calculations of Pearson correlation coefficients and their statistical significance, and correlation plots were carried out using GraphPad Prism version 10.6.1 for Windows, GraphPad Software, Boston, MA, USA, www.graphpad.com (accessed on 1 October 2025).

## 4. Conclusions

A synthetic method for producing azinyl-containing alkenylferrocenes has been developed. It was demonstrated that 1-azinyl-1′-acetylferrocenes can participate in the Wittig reaction to yield 1-azinyl-1′-isopropenylferrocenes. Additionally, sequential reduction and dehydration reactions of 1-azinyl-1′-acetylferrocenes can lead to the formation of 1-azinyl-1′-vinylferrocenes with excellent yields (up to 98%).

Quinoline and bipyridine derivatives of Fc inhibited cholinesterases within the micromolar range and showed minimal inhibition of the off-target enzyme CES. Enzyme kinetics studies demonstrated that the lead compound **7b** exerted a mixed type of inhibition on the cholinesterases. These compounds, along with acridine derivatives, displaced propidium at a level comparable to the reference compound donepezil, indicating their potential to inhibit AChE-induced β-amyloid aggregation. They also exhibited strong inhibitory effects on *Hs*Aβ_42_ self-aggregation, achieving efficacies similar to that of the reference compound myricetin. Among the synthesized compounds, quinoline derivatives displayed the highest antioxidant activity, being 4 times more potent than the standard antioxidant Trolox in the ABTS test and 2–3 times in the FRAP assay, which agrees with the results of QC calculations.

QC calculations have shown (see Table S3) that the advantage of dimer formation growths with increasing size of the aromatic heterocyclic ligand. At the same time, buffer anions increase the advantage of dimer formation for protonated compounds.

Calculations of Tanimoto similarity coefficients and molecular volumes quantified the chemical diversity of the study compounds and assisted with making inferences about the relative access of the monomeric and dimeric forms of the compounds to interior or exterior binding sites of proteins.

This study employed a novel approach to molecular docking by using Protenix, an AlphaFold3 reproduction code, to generate the 3D structures of the protein docking targets. Molecular docking results supported the QC predictions that the acridine derivatives in particular would exist in the conditions of the esterase assays as dimers, which would be too bulky to gain access to the catalytic active sites. However, either as dimers or monomers, the Fc derivatives could bind to the PAS of *Ee*AChE to displace propidium. They could also bind to the surfaces of *Hs*Aβ_42_ to block the self-aggregation of this peptide. Data derived from molecular docking, such as heatmaps of overlap volumes of docked ligands, proved helpful for making predictions about similar or dissimilar actions of compounds on target proteins, especially with respect to displacement of propidium or inhibition of *Hs*Aβ_42_ self-aggregation. When considering propidium displacement as a binary response and classifying the study compounds as active or inactive, it was possible to predict the classifications with 94.7% accuracy using linear discriminant analysis and only two variables from molecular docking of the monomers: binding energy and propidium overlap volumes. Inhibition of *Hs*Aβ_42_ self-aggregation showed a graded response that had strong correlations with the ligand binding energies to the protein.

Quinoline and bipyridine derivatives also exhibited relatively low cytotoxicity on neuronal cells MSC-Neu and human neuroblastoma tumor cells SH-SY5Y being less toxic for MSC-Neu cells.

Overall, these findings suggest that Fc derivatives with quinoline and bipyridine substituents are promising candidates for further investigation as potential multifunctional therapeutic agents for AD. We recommend that future studies focus on optimizing pharmacokinetics parameters with special emphasis on bioavailability and BBB permeability.

## Figures and Tables

**Table 1:** Yields of compounds **1-2a-e** and **5-7a-e**.

Azinyl Substituent	Yield 1a-e (%)	Yield 2a-e (%)	Yield 5a-e (%)	Yield 6a-e (%)	Yield 7a-e (%)
Pyridin-2-yl (**a**)	67 [52]	67	72	93	90
Quinolin-2-yl (**b**)	67 [52]	55	81	98	93
Acridin-9-yl (**c**)	80 [52]	52	75	86	88
2,2′-Bipyridin-6-yl (**d**)	78 [52]	62	68	98	95
Pyridazin-4-yl (**e**)	53	58	63	82	-

**Table 2:** Esterase profiles of compounds **1a-e**, **5a-e**, **7a-d**, **Fc**, **8**, and **9**; their ability to displace propidium from the *Ee*AChE PAS; and their inhibition of *Hs*Aβ_42_ self-aggregation.

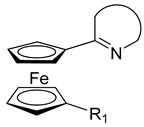						
**No**	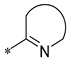	**Inhibition of *Hs* AChE, *Ec*BChE and *Ss*CES** **IC_50_, μM or % Inhibition at 20 μM**			**Propidium Displacement, (%) ^1^**	***Hs*Aβ_42_ self-Aggregation Inhibition, (%) ^2^**
		***Hs*AChE**	***Ec*BChE**	***Ss*CES**		
**1-azinyl-Fcs, R_1_=H**						
**1a**	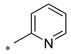	n.a.	20.3 ± 1.4%	17.5 ± 1.3%	n.a.	43.4 ± 3.4
**1b**	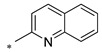	68.1 ± 3.4 μM	2.70 ± 0.19 μM	29.8 ± 2.0%	10.2 ± 0.8	79.5 ± 6.3
**1c**	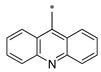	6.5 ± 0.8%	21.2 ± 1.6%	n.a.	11.2 ± 0.9	71.0 ± 4.9
**1d**	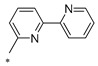	7.89 ± 0.63 μM	13.8 ± 1.2 μM	20.9 ± 1.6%	11.1 ± 0.9	63.7 ± 5.1
**1e**	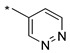	n.a.	13.1 ± 1.4%	n.a.	n.a.	53.3 ± 3.7
**Fc**	-	9.6 ± 1.4%	6.7 ± 1.0%	4.2 ± 0.7%	n.a.	3.5 ± 0.4
**1-azinyl-1′-isopropenyl-Fcs, R_1_ = –C(CH_3_)=CH_2_**						
**5a**	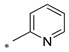	4.4 ± 1.1%	18.3 ± 1.6%	n.a.	n.a.	62.4 ± 4.9
**5b**	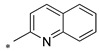	4.01 ± 0.28 μM	9.91 ± 0.69 μM	35.4 ± 2.8%	11.5 ± 0.8	82.9 ± 6.6
**5c**	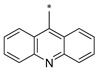	36.7 ± 3.3%	96.7 ± 6.6 μM	n.a.	10.5 ± 0.9	90.1 ± 7.2
**5d**	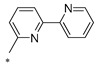	5.12 ± 0.41 μM	4.86 ± 0.39 μM	n.a.	8.2 ± 0.6	83.8 ± 4.2
**5e**	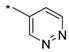	n.a.	n.a.	n.a.	n.a.	48.6 ± 3.5
**8**	-	n.a.	3.9 ± 0.8%	2.9 ± 0.6%	n.a.	n.a.
**1-azinyl-1′-vinyl-Fcs, R_1_ = –CH=CH_2_**						
**7a**	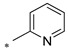	16.5 ± 1.3 μM	20.1 ± 1.6 μM	18.5 ± 1.6%	9.6 ± 0.7	84.6 ± 7.5
**7b**	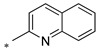	3.32 ± 0.23 μM	3.68 ± 0.29 μM	29.8 ± 2.6%	7.7 ± 0.6	89.6 ± 6.2
**7c**	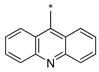	22.8 ± 1.8%	7.6 ± 1.1%	14.1 ± 1.5%	10.4 ± 0.8	86.9 ± 6.1
**7d**	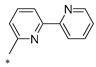	6.34 ± 0.51 μM	7.94 ± 0.55 μM	5.8 ± 1.3%	9.7 ± 0.6	81.5 ± 4.9
**9**	-	4.2 ± 0.9%	n.a.	23.4 ± 2.1%	n.a.	44.9 ± 3.1
BNPP		n.a.	n.a.	99.1 ± 0.9% 1.80 ± 0.11 µM	n.d.	n.d.
Tacrine		0.601 ± 0.047 μM	0.0295 ± 0.0002 μM	n.a.	3.1 ± 0.2	5.9 ± 0.5
Donepezil		0.040 ± 0.004 μM	19.2 ± 3.0 μM	n.a.	11.9 ± 0.9	n.d.
Myricetin		n.d.	n.d.	n.d.	n.d.	79.4 ± 6.3

^1^ Propidium displacement from the peripheral anionic site (PAS) of *Electrophorus electricus* AChE (*Ee*AChE); compound concentration 20 µM; ^2^ Inhibition of human β-amyloid (1–42) (*Hs*Aβ_42_) self-aggregation (50 µM) by the tested compound at 100 µM concentration. n.d.—not determined. n.a.—not active. Data are expressed as mean ± SEM, *n* = 3.

**Table 3:** Antioxidant activity of **Fc**, alkenyl-Fcs **8**, **9** and their 1,1′-disubstituted derivatives **1a-e**, **5a-e**, and **7a-d**.

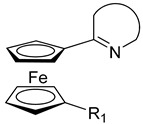				
**No**	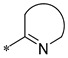	**ABTS^•+^-Scavenging** **Activity**		**Ferric Reducing** **Antioxidant Power**
		**TEAC ^1^**	**IC_50_, µM**	**TE ^2^**
**1-azinyl-Fcs, R_1_=H**				
**1a**	Pyridyl-2	2.1 ± 0.1	10.8 ± 0.6	2.05 ± 0.02
**1b**	Quinolin-2-yl	4.2 ± 0.2	6.6 ± 0.3	2.21 ± 0.01
**1c**	Acridin-9-yl	1.50 ± 0.08	14.6 ± 0.3	1.59 ± 0.17
**1d**	2,2′-Bipyridin-6-yl	1.00 ± 0.04	20.6 ± 1.5	1.77 ± 0.04
**1e**	Pyridazin-4-yl	2.3 ± 0.1	9.2 ± 0.5	1.72 ± 0.02
**Fc**	**-**	0.57 ± 0.03	32.9 ± 1.9	0.79 ± 0.02
**1-azinyl-1′-isopropenyl-Fcs, R_1_ = –C(CH_3_)=CH_2_**				
**5a**	Pyridyl-2	2.2 ± 0.1	9.4 ± 0.5	2.85 ± 0.13
**5b**	Quinolin-2-yl	4.3 ± 0.2	5.6 ± 0.3	3.13 ± 0.17
**5c**	Acridin-9-yl	1.35 ± 0.06	15.8 ± 0.8	2.45 ± 0.14
**5d**	2,2′-Bipyridin-6-yl	0.94 ± 0.05	20.8 ± 0.1	1.47 ± 0.06
**5e**	Pyridazin-4-yl	2.5 ± 0.2	9.3 ± 0.4	2.65 ± 0.02
**8**	**-**	0.73 ± 0.04	26.3 ± 1.5	1.1 ± 0.1
**1-azinyl-1′-vinyl-Fcs, R_1_ = –CH=CH_2_**				
**7a**	Pyridyl-2	2.60 ± 0.08	8.4 ± 0.3	2.13 ± 0.03
**7b**	Quinolin-2-yl	4.4 ± 0.2	5.3 ± 0.2	2.21 ± 0.02
**7c**	Acridin-9-yl	2.9 ± 0.1	6.9 ± 0.3	1.36 ± 0.01
**7d**	2,2′-Bipyridin-6-yl	1.36 ± 0.06	15.6 ± 0.7	1.93 ± 0.06
**9**	**-**	1.00 ± 0.04	19.4 ± 0.1	1.04 ± 0.02
Trolox		1.0	20.1 ± 1.2	1.0

^1^ TEAC (Trolox equivalent antioxidant capacity, ABTS) and ^2^ TE (Trolox equivalent, FRAP)—activity of the compounds relative to Trolox (for calculation, see Experimental). Data are expressed as mean ± SEM, *n* = 3.

**Table 4:** Calculated BDE and IP values in water/ethanol and experimental AOA values in FRAP and ABTS tests.

Compound	BDE, kcal/mol		IP, kcal/mol		Exp. FRAP, TE	Exp. ABTS, TEAC
	Water	Ethanol	Water	Ethanol	Water	Ethanol
**Fc**	111.6 ^1^	111.2 ^1^	74.9	82.6	0.79 ± 0.02	0.57 ± 0.03
**1a**	- not calculated	105.5 ^5^	- not calculated	83.1	-	2.10 ± 0.10
**1a**_p_	67.5 ^4^	67.1 ^4^	81.0	90.6	2.05 ± 0.02	
**1b**_p_	69.1 ^4^	68.7 ^4^	81.6	91.3	2.21 ± 0.01	4.20 ± 0.20
**1c**_p_	68.8 ^4^	69.5 ^4^	78.8	88.4	1.59 ± 0.17	1.50 ± 0.08
**8**	88.1 ^3^	87.0 ^3^	74.0	82.0	1.10 ± 0.10	0.73 ± 0.04
**5a**	- not calculated	86.9 ^3^	- not calculated	82.0	-	2.20 ± 0.10
**5a**_p_	66.8 ^4^	66.6 ^4^	79.9	89.4	2.85 ± 1.3	
**5b**_p_	68.9 ^4^	68.4 ^4^	80.5	90.2	3.13 ± 1.7	4.30 ± 0.20
**5c**_p_	68.5 ^4^	69.1 ^4^	80.3	89.2	2.45 ± 0.14	1.35 ± 0.06
**9**	103.2 ^2^	102.0 ^2^	74.7	82.2	1.04 ± 0.02	1.00 ± 0.04
**7a**	- not calculated	101.5 ^2^	- not calculated	82.4	-	2.60 ± 0.03
**7a_p_**	67.1 ^4^	66.8 ^4^	80.9	90.6	2.13 ± 0.03	
**7b_p_**	68.6 ^4^	68.3 ^4^	81.1	90.8	2.21 ± 0.02	4.40 ± 0.03
**7c_p_**	68.5 ^4^	69.0 ^4^	77.8	87.4	1.36 ± 0.01	2.90 ± 0.03

^1^ For H of Cp, ^2^ For H^1^ in **–CH^1^ = CH^2^_2_** vinyl group, ^3^ For H^1^ in *iso*-propenyl group **–C(CH^1^_3_)=CH^2^_2_**, ^4^ For proton in the heterocyclic aromatic group, ^5^ For H in ortho position of the pyridine group.

**Table 5:** Cytotoxic activity of Fcs (MTT test data, LC_50_, μM).

	1b	1d	5b	5d	7b	7d	10	11
**MSC-Neu**	393.1 ± 13.2	632.3 ± 22.8	564.3 ± 31.8	413.9 ± 18.6	277 ± 14.8	299.2 ± 21.9	103.6 ± 12.3	138.9 ± 15.7
**SH-SY5Y**	242.7 ± 27.4	152.4 ± 21.2	281 ± 30.1	188.8 ± 19.3	287.4 ± 31.7	158.1 ± 19.8	43.4 ± 6.7	57.8 ± 8.2

Data are expressed as mean ± SE, *n* = 3.

## Data Availability

The original contributions presented in this study are included in the article/Supplementary Material. Further inquiries can be directed to the corresponding author.

## Supplementary Materials

The following supporting information can be downloaded at: https://www.mdpi.com/article/10.3390/ph18121862/s1. References [139,140,141,142,143,144,145,146,147,148,149,150,151,152,153,154] are cited in the Supplementary Materials only. Figure S1.1: ^1^H NMR (600 MHz) spectrum for 1-(pyridazin-4-yl)ferrocene (**1e**); Figure S1.2: ^1^H NMR (600 MHz) spectrum for 1-acetyl-1′-(pyridazin-4-yl)ferrocene (**2e**); Figure S1.3: ^1^H NMR (600 MHz) spectrum for 1-isopropenyl-1′-(pyridazin-4-yl)ferrocene (**5e**). Figure S1.4: ^1^H NMR (600 MHz) spectrum for 1-(pyridin-2-yl)-1′-(α-hydroxyethyl)ferrocene (**6a**). Figure S1.5: ^1^H NMR (600 MHz) spectrum for 1-(quinolin-2-yl)-1′-(α-hydroxyethyl)ferrocene (**6b**). Figure S1.6: ^1^H NMR (600 MHz) spectrum for 1-(acridin-9-yl)-1′-(α-hydroxyethyl)ferrocene (**6c**). Figure S1.7: ^1^H NMR (600 MHz) spectrum for 1-(2,2′-bipyridin-6-yl)-1′-(α-hydroxyethyl)ferrocene (**6d**). Figure S1.8: ^1^H NMR (400 MHz) spectrum for 1-(pyridazin-4-yl)-1′-(α-hydroxyethyl)ferrocene (**6e**). Figure S1.9: ^1^H NMR (600 MHz) spectrum for 1-(pyridin-2-yl)-1′-vinylferrocene (**7a**). Figure S1.10: ^1^H NMR (600 MHz) spectrum for 1-(quinolin-2-yl)-1′-vinylferrocene (**7b**). Figure S1.11. ^1^H NMR (600 MHz) spectrum for 1-(acridin-9-yl)-1′-vinylferrocene (**7c**). Figure S1.12: ^1^H NMR (600 MHz) spectrum for 1-(2,2′-bipyridin-6-yl)-1′-vinylferrocene (**7d**). Figure S1.13: ^1^H NMR (600 MHz) spectrum for isopropenylferrocene (**8**) Figure S1.14. ^1^H NMR (600 MHz) spectrum for 1,1-bis(4-hydroxyphenyl)-2-ferrocenylprop-1-ene (**10**). Figure S1.15: ^1^H NMR (600 MHz) spectrum for 1-(quinolin-2-yl)-1’-{1-[bis(4-hydroxyphenyl)methylene]ethyl}ferrocene (**11**). Figure S2.1: ^13^C NMR (151 MHz) spectrum for 1-(pyridazin-4-yl)ferrocene (**1e**). Figure S2.2: ^13^C NMR (151 MHz) spectrum for 1-acetyl-1′-(pyridazin-4-yl)ferrocene (**2e**). Figure S2.3: ^13^C NMR (151 MHz) spectrum for 1-isopropenyl-1′-(pyridazin-4-yl)ferrocene (**5e**). Figure S2.4: ^13^C NMR (151 MHz) spectrum for 1-(pyridin-2-yl)-1′-(α-hydroxyethyl)ferrocene (**6a**). Figure S2.5: ^13^C NMR (151 MHz) spectrum for 1-(quinolin-2-yl)-1′-(α-hydroxyethyl)ferrocene (**6b**). Figure S2.6: ^13^C NMR (151 MHz) spectrum for 1-(acridin-9-yl)-1′-(α-hydroxyethyl)ferrocene (**6c**). Figure S2.7: ^13^C NMR (151 MHz) spectrum for 1-(2,2′-bipyridin-6-yl)-1′-(α-hydroxyethyl)ferrocene (**6d**). Figure S2.8: ^13^C NMR (100 MHz) spectrum for 1-(pyridazin-4-yl)-1′-(α-hydroxyethyl)ferrocene (**6e**). Figure S2.9: ^13^C NMR (151 MHz) spectrum for 1-(pyridin-2-yl)-1′-vinylferrocene (**7a**). Figure S2.10: ^13^C NMR (151 MHz) spectrum for 1-(quinolin-2-yl)-1′-vinylferrocene (**7b**). Figure S2.11: ^13^C NMR (151 MHz) spectrum for 1-(acridin-9-yl)-1′-vinylferrocene (**7c**). Figure S2.12: ^13^C NMR (151 MHz) spectrum for 1-(2,2′-bipyridin-6-yl)-1′-vinylferrocene (**7d**). Figure S2.13: ^13^C NMR (151 MHz) spectrum for isopropenylferrocene (**8**). Figure S2.14: ^13^C NMR (151 MHz) spectrum for 1,1-bis(4-hydroxyphenyl)-2-ferrocenylprop-1-ene (**10**). Figure S2.15: ^13^C NMR (151 MHz) spectrum for 1-(quinolin-2-yl)-1’-{1-[bis(4-hydroxyphenyl)methylene]ethyl}ferrocene (**11**). Figure S3.1: ^1^H-^13^C HSQC spectrum for 1-(pyridazin-4-yl)ferrocene (**1e**). Figure S3.2: ^1^H-^13^C HMBC spectrum for 1-(pyridazin-4-yl)ferrocene (**1e**). Figure S3.3: ^1^H-^13^C HSQC spectrum for 1-acetyl-1′-(pyridazin-4-yl)ferrocene (**2e**). Figure S3.4: ^1^H-^13^C HMBC spectrum for 1-acetyl-1′-(pyridazin-4-yl)ferrocene (**2e**). Figure S3.5: ^1^H-^13^C HSQC spectrum for 1-isopropenyl-1′-(pyridazin-4-yl)ferrocene (**5e**). Figure S3.6: ^1^H-^13^C HMBC spectrum for 1-isopropenyl-1′-(pyridazin-4-yl)ferrocene (**5e**). Figure S3.7: ^1^H-^13^C HSQC spectrum for 1-(pyridin-2-yl)-1′-(α-hydroxyethyl)ferrocene (**6a**). Figure S3.8: ^1^H-^13^C HMBC spectrum for 1-(pyridin-2-yl)-1′-(α-hydroxyethyl)ferrocene (**6a**). Figure S3.9: ^1^H-^13^C HSQC spectrum for 1-(quinolin-2-yl)-1′-(α-hydroxyethyl)ferrocene (**6b**). Figure S3.10: ^1^H-^13^C HMBC spectrum for 1-(quinolin-2-yl)-1′-(α-hydroxyethyl)ferrocene (**6b**). Figure S3.11: ^1^H-^13^C HSQC spectrum for 1-(acridin-9-yl)-1′-(α-hydroxyethyl)ferrocene (**6c**). Figure S3.12: ^1^H-^13^C HMBC spectrum for 1-(acridin-9-yl)-1′-(α-hydroxyethyl)ferrocene (**6c**). Figure S3.13: ^1^H-^13^C HSQC spectrum for 1-(2,2′-bipyridin-6-yl)-1′-(α-hydroxyethyl)ferrocene (**6d**). Figure S3.14: ^1^H-^13^C HMBC spectrum for 1-(2,2′-bipyridin-6-yl)-1′-(α-hydroxyethyl)ferrocene (**6d**). Figure S3.15: ^1^H-^13^C HSQC spectrum for 1-(pyridazin-4-yl)-1′-(α-hydroxyethyl)ferrocene (**6e**). Figure S3.16: ^1^H-^13^C HMBC spectrum 1-(pyridazin-4-yl)-1′-(α-hydroxyethyl)ferrocene (**6e**). Figure S3.17: ^1^H-^13^C HSQC spectrum for 1-(pyridin-2-yl)-1′-vinylferrocene (**7a**). Figure S3.18: ^1^H-^13^C HMBC spectrum for 1-(pyridin-2-yl)-1′-vinylferrocene (**7a**). Figure S3.19: ^1^H-^13^C HSQC spectrum for 1-(quinolin-2-yl)-1′-vinylferrocene (**7b**). Figure S3.20: ^1^H-^13^C HMBC spectrum for 1-(quinolin-2-yl)-1′-vinylferrocene (**7b**). Figure S3.21: ^1^H-^13^C HSQC spectrum for 1-(acridin-9-yl)-1′-vinylferrocene (**7c**). Figure S3.22: ^1^H-^13^C HMBC spectrum for 1-(acridin-9-yl)-1′-vinylferrocene (**7c**). Figure S3.23: ^1^H-^13^C HSQC spectrum for 1-(2,2′-bipyridin-6-yl)-1′-vinylferrocene (**7d**). Figure S3.24: ^1^H-^13^C HMBC spectrum for 1-(2,2′-bipyridin-6-yl)-1′-vinylferrocene (**7d**). Figure S3.25. ^1^H-^13^C HSQC spectrum for isopropenylferrocene (**8**). Figure S3.26. ^1^H-^13^C HMBC spectrum for isopropenylferrocene (**8**). Figure S3.27. ^1^H-^13^C HSQC spectrum for 1,1-bis(4-hydroxyphenyl)-2-ferrocenylprop-1-ene (**10**). Figure S3.28. ^1^H-^13^C HMBC spectrum for 1,1-bis(4-hydroxyphenyl)-2-ferrocenylprop-1-ene (**10**). Figure S3.29: ^1^H-^13^C HSQC spectrum for 1-(quinolin-2-yl)-1’-{1-[bis(4-hydroxyphenyl)methylene]ethyl}ferrocene (**11**). Figure S3.30: ^1^H-^13^C HMBC spectrum for 1-(quinolin-2-yl)-1’-{1-[bis(4-hydroxyphenyl)methylene]ethyl}ferrocene (**11**). Figure S4.1: IR spectrum for 1-(pyridazin-4-yl)ferrocene (**1e**). Figure S4.2: IR spectrum for 1-acetyl-1′-(pyridazin-4-yl)ferrocene (**2e**). Figure S4.3: IR spectrum for 1-isopropenyl-1′-(pyridazin-4-yl)ferrocene (**5e**). Figure S4.4: IR spectrum for 1-(pyridin-2-yl)-1′-(α-hydroxyethyl)ferrocene (**6a**). Figure S4.5: IR spectrum for 1-(quinolin-2-yl)-1′-(α-hydroxyethyl)ferrocene (**6b**). Figure S4.6: IR spectrum for 1-(acridin-9-yl)-1′-(α-hydroxyethyl)ferrocene (**6c**). Figure S4.7: IR spectrum for 1-(2,2′-bipyridin-6-yl)-1′-(α-hydroxyethyl)ferrocene (**6d**). Figure S4.8: IR spectrum for 1-(pyridazin-4-yl)-1′-(α-hydroxyethyl)ferrocene (**6e**). Figure S4.9: IR spectrum for 1-(pyridin-2-yl)-1′-vinylferrocene (**7a**). Figure S4.10: IR spectrum for 1-(quinolin-2-yl)-1′-vinylferrocene (**7b**). Figure S4.11: IR spectrum for 1-(acridin-9-yl)-1′-vinylferrocene (**7c**). Figure S4.12: IR spectrum for 1-(2,2′-bipyridin-6-yl)-1′-vinylferrocene (**7d**). Figure S4.13: IR spectrum for isopropenylferrocene (**8**). Figure S4.14: IR spectrum for 1,1-bis(4-hydroxyphenyl)-2-ferrocenylprop-1-ene (**10**). Figure S4.15: IR spectrum for 1-(quinolin-2-yl)-1’-{1-[bis(4-hydroxyphenyl)methyl-ene]ethyl}ferrocene (**11**). Figure S6.1: Dimers in water: compounds 1ap (a), 1bp (b), 1cp (c) from different points of view. Carbon atoms of one molecule are green; carbon atoms of the other molecule are marsh green. Figure S6.2: Dimers in water stabilized by HPO_4_^2−^ anion: compounds 1ap (a), 1bp (b), 1cp (c) from different points of view. Carbon atoms of one molecule are green; carbon atoms of the other molecule are marsh green. Figure S6.3: Dimers in water stabilized by CH_3_COO^−^ anion: compounds 1ap (a), 1bp (b), 1cp (c) from different points of view. Carbon atoms of one molecule are green; carbon atoms of the other molecule are marsh green. Carbon atoms of CH_3_COO^−^ anion are bright green. Figure S6.4: Dimers in water stabilized by two Cl^−^ anions (Tris-HCl buffer, pH = 8.0): compounds 1ap (a), 1bp (b), 1cp (c) from different points of view. Carbon atoms of one molecule are green; carbon atoms of the other molecule are marsh green. Chloride anions are bright green. Figure S6.5: Dimers in ethanol stabilized by SO_4_^2−^ anion: compounds 1ap (a), 1bp (b), 1cp (c) from different points of view. Carbon atoms of one molecule are green; carbon atoms of the other molecule are marsh green. Figure S7.1. Heatmap of percent overlap of docked monomer ligands in *Ec*BChE. Figure S7.2: Mixed heatmap of percent overlap of docked monomer and dimer ligands in *Ec*BChE. Figure S7.3: Heatmap of percent overlap of docked monomer ligands in *Ee*AChE. Figure S7.4. Mixed heatmap of percent overlap of docked monomer and dimer ligands in *Ee*AChE. Figure S7.5. Heatmap of percent overlap of docked monomer ligands in *Hs*Aβ42. Figure S7.6: Mixed heatmap of percent overlap of docked monomer and dimer ligands in HsAβ42. Figure S7.7: Heatmap of percent overlap of a set of docked ligands in *Ss*CES1. Figure S7.8: Mixed heatmap of percent overlap of docked monomer and dimer ligands in *Ss*CES1. Figure S8.1: Structure of *Hs*AChE generated by the AlphaFold3 reproduction code, Protenix 0.5.0 [76] containing the top-scoring docking pose from the implementation of Autodock Vina 1.2.5 [116,117] in YASARA 25.07.15 for Linux [104] of the reference ligand, donepezil (carbon atoms colored blue), 3D-aligned with an X-ray crystal structure of *Hs*AChE in complex with donepezil (carbon atoms colored yellow) (PDB ID 4EY7; resolution 2.35 A). Ligands shown as sticks; protein secondary structure shown as gray mesh. Hydrogen atoms hidden for clarity. Active site S203 residue shown for orientation. Protein alignment performed with SHEBA [153] in YASARA. Ligand RMSD = 0.541 Å calculated by DockRMSD [154]. Image rendered in YASARA. Table S1. Calculated PA values in water and experimental p*K*a values of pyridine, quinolone and acridine. Table S2: Calculated PA values in ethanol (ABTS test conditions). Table S3: Enthalpy of dimers formation (kcal/mol). Table S4: Sequences of primers used in the study.

## Abbreviations

The following abbreviations are used in this manuscript:

3D	Three-dimensional
AChE	Acetylcholinesterase
AD	Alzheimer’s disease
AF3	AlphaFold3
AMD	American Micro Devices
Azinyl-Fc(s)	Azinylferrocene(s)
BChE	Butyrylcholinesterase
bnp	Bis(4-nitrophenyl) phosphate (BNPP)
BNPP	Bis(4-nitrophenyl) phosphate
CAS	Catalytic active site
CES	Carboxylesterase
ChE	Cholinesterase
CPU	Central processing unit
don	Donepezil
Eb	Binding energy
*Ec*BChE	*Equus caballus* (equine; horse serum) butyrylcholinesterase
*Ee*AChE	*Electrophorus electricus* (electric eel) acetylcholinesterase
FASTA	FAST-All file format for single-letter format sequences
Fc	Ferrocene
GB	Gigabyte
GPU	Graphical processing unit
*Hs*Aβ_42_	*Homo sapiens* (human) amyloid-βpeptide (1–42)
*Hs*AChE	*Homo sapiens* (human) acetylcholinesterase
LDA	Linear discriminant analysis
MSA	Multiple sequence alignment
myr	Myricetin
NCBI	National Center for Biotechnology Information
PAS	Peripheral anionic site
PAST	PAleontological STatistics
PDB	Protein Data Bank
prm	Propidium
QC	Quantum chemical; quantum-chemical
RAM	Random access memory
RTX	Ray tracing texel eXtreme
SCE	YASARA scene file
SDF	Structure-data format
*Ss*CES	*Sus scrofa* (porcine; pig) carboxylesterase
*Ss*CES1	*Sus scrofa* (porcine; pig) carboxylesterase 1
tac	Tacrine
Tc	Tanimoto coefficient
Vn	Normalized volume
YASARA	Yet another scientific artificial reality application
YOB	YASARA object file

## References

[1] Larik F.A., Saeed A., Fattah T.A., Muqadar U., Channar P.A. (2016). Recent advances in the synthesis, biological activities and various applications of ferrocene derivatives. Appl. Organomet. Chem..

[2] Singh A., Lumb I., Mehra V., Kumar V. (2019). Ferrocene-appended pharmacophores: An exciting approach for modulating the biological potential of organic scaffolds. Dalton Trans..

[3] Sharma S. (2023). A Short Review of Past, Present, and Future of Metallocene and Its Derivatives as an Effective Therapeutic Agent. J. Appl. Organomet. Chem..

[4] Sharma A., Rana R., Kumar N., Kaur Gulati H., Jyoti, Khanna A., Sharma S., Pooja, Singh J.V., Bedi P.M.S. (2025). Ferrocene-based compounds: Promising anticancer and antimalarial agents in modern therapeutics. Med. Chem. Res..

[5] Snegur L.V. (2022). Modern Trends in Bio-Organometallic Ferrocene Chemistry. Inorganics.

[6] Jančić A.M., Katanić Stanković J.S., Srećković N., Mihailović V., Komatina D.I., Stevanović D. (2022). Ferrocene-containing tetrahydropyrimidin-2(1H)-ones: Antioxidant and antimicrobial activity. J. Organomet. Chem..

[7] Patra M., Gasser G. (2017). The medicinal chemistry of ferrocene and its derivatives. Nat. Rev. Chem..

[8] Fouda M.F.R., Abd-Elzaher M.M., Abdelsamaia R.A., Labib A.A. (2007). On the medicinal chemistry of ferrocene. Appl. Organomet. Chem..

[9] Yeganeh M.S., Abbasi F., Kazemizadeh A.R. (2019). Recent Advances in the Synthesis of Ferrocene-Based Heterocycles by Multicomponent Reactions: A Review. Curr. Org. Chem..

[10] Halder B., Mitra A., Dewangan S., Gazi R., Sarkar N., Jana M., Chatterjee S. (2023). Solid state synthesis of bispyridyl-ferrocene conjugates with unusual site selective 1,4-Michael addition, as potential inhibitor and electrochemical probe for fibrillation in amyloidogenic protein. J. Mol. Struct..

[11] Goyal D., Shuaib S., Mann S., Goyal B. (2017). Rationally Designed Peptides and Peptidomimetics as Inhibitors of Amyloid-beta (Abeta) Aggregation: Potential Therapeutics of Alzheimer’s Disease. ACS Comb. Sci..

[12] Yao P., Zhang J., You S., Qi W., Su R., He Z. (2020). Ferrocene-modified peptides as inhibitors against insulin amyloid aggregation based on molecular simulation. J. Mater. Chem. B.

[13] Jawaria R., Hussain M., Ahmad H.B., Ashraf M., Hussain S., Naseer M.M., Khalid M., Hussain M.A., al-Rashida M., Tahir M.N. (2020). Probing ferrocene-based thiosemicarbazones and their transition metal complexes as cholinesterase inhibitors. Inorg. Chim. Acta.

[14] Almansour A.I., Ibrahim Al-Shemaimari K., Ibhrahim Alaqeel S. (2024). Cholinesterase inhibitory activity and regioselective synthesis of spiropyrrolidinoindole integrated ferrocene hybrid heterocycles via multicomponent cycloaddition reaction. J. King Saud Univ. Sci..

[15] Altaf A.A., Kausar S., Hamayun M., Lal B., Tahir M.N., Badshah A. (2017). Ferrocenylaniline based amide analogs of methoxybenzoic acids: Synthesis, structural characterization and butyrylcholinesterase (BChE) inhibition studies. J. Mol. Struct..

[16] Altaf A.A., Hamayun M., Lal B., Tahir M.N., Holder A.A., Badshah A., Crans D.C. (2018). Ferrocene-based anilides: Synthesis, structural characterization and inhibition of butyrylcholinesterase. Dalton Trans..

[17] Puranik N., Song M. (2025). Therapeutic Role of Heterocyclic Compounds in Neurodegenerative Diseases: Insights from Alzheimer’s and Parkinson’s Diseases. Neurol. Int..

[18] Waly O.M., Saad K.M., El-Subbagh H.I., Bayomi S.M., Ghaly M.A. (2022). Synthesis, biological evaluation, and molecular modeling simulations of new heterocyclic hybrids as multi-targeted anti-Alzheimer’s agents. Eur. J. Med. Chem..

[19] Kumari S., Maddeboina K., Bachu R.D., Boddu S.H.S., Trippier P.C., Tiwari A.K. (2022). Pivotal role of nitrogen heterocycles in Alzheimer’s disease drug discovery. Drug Discov. Today.

[20] Sanchez J.D., Alcantara A.R., Gonzalez J.F., Sanchez-Montero J.M. (2023). Advances in the discovery of heterocyclic-based drugs against Alzheimer’s disease. Expert Opin. Drug Discov..

[21] Ling Y., Hao Z.-Y., Liang D., Zhang C.-L., Liu Y.-F., Wang Y. (2021). The Expanding Role of Pyridine and Dihydropyridine Scaffolds in Drug Design. Drug Des. Devel. Ther..

[22] De S., Kumar S.K.A., Shah S.K., Kazi S., Sarkar N., Banerjee S., Dey S. (2022). Pyridine: The scaffolds with significant clinical diversity. RSC Adv..

[23] Shaik J.B., Pinjari M.K.M., Gangaiah D.A., Nallagondu C.G.R., Singh P. (2023). Synthetic strategies of functionalized pyridines and their therapeutic potential as multifunctional anti-Alzheimer’s agents. Recent Developments in the Synthesis and Applications of Pyridines.

[24] Rammohan A., Bhaskar B.V., Zyryanov G.V., Singh P. (2023). Chapter 13—Recent developments in the synthesis of pyridine analogues as a potent anti-Alzheimer’s therapeutic leads. Recent Developments in the Synthesis and Applications of Pyridines.

[25] Kalhor H.R., Nazari Khodadadi A. (2018). Synthesis and Structure Activity Relationship of Pyridazine-Based Inhibitors for Elucidating the Mechanism of Amyloid Inhibition. Chem. Res. Toxicol..

[26] Alghamdi S., Asif M. (2021). Role of pyridazine analogs as acetylcholinesterase inhibitor: An approach for management of Alzheimer’s disease. Eurasian Chem. Commun..

[27] Tan R.X., Li W.H., Pang J.M., Zhong S.M., Huang X.Y., Deng J.Z., Zhou L.Y., Wu J.Q., Wang X.Q. (2024). Design, synthesis, and evaluation of 2,2′-bipyridyl derivatives as bifunctional agents against Alzheimer’s disease. Mol. Divers..

[28] Nagani A., Shah M., Patel S., Patel H., Parikh V., Patel A., Patel S., Patel K., Parmar H., Bhimani B. (2024). Unveiling piperazine-quinoline hybrids as potential multi-target directed anti-Alzheimer’s agents: Design, synthesis and biological evaluation. Mol. Divers..

[29] Kostopoulou I., Diassakou A., Kavetsou E., Kritsi E., Zoumpoulakis P., Pontiki E., Hadjipavlou-Litina D., Detsi A. (2021). Novel quinolinone-pyrazoline hybrids: Synthesis and evaluation of antioxidant and lipoxygenase inhibitory activity. Mol. Divers..

[30] Kostopoulou I., Tzani A., Chronaki K., Prousis K.C., Pontiki E., Hadjiplavlou-Litina D., Detsi A. (2023). Novel Multi-Target Agents Based on the Privileged Structure of 4-Hydroxy-2-quinolinone. Molecules.

[31] Yadav V., Reang J., Sharma V., Majeed J., Sharma P.C., Sharma K., Giri N., Kumar A., Tonk R.K. (2022). Quinoline-derivatives as privileged scaffolds for medicinal and pharmaceutical chemists: A comprehensive review. Chem. Biol. Drug. Des..

[32] Chauhan M.S.S., Umar T., Aulakh M.K. (2023). Quinolines: Privileged Scaffolds for Developing New Anti-neurodegenerative Agents. ChemistrySelect.

[33] Sharma A., Piplani P. (2023). Acridine: A Scaffold for the Development of Drugs for Alzheimer’s Disease. Curr. Top. Med. Chem..

[34] Mars A., Argoubi W., Ben Aoun S., Raouafi N. (2016). Induced conformational change on ferrocenyl-terminated alkyls and their application as transducers for label-free immunosensing of Alzheimer’s disease biomarker. RSC Adv..

[35] Kumar N., Kumar V., Anand P., Kumar V., Ranjan Dwivedi A., Kumar V. (2022). Advancements in the development of multi-target directed ligands for the treatment of Alzheimer’s disease. Bioorg. Med. Chem..

[36] Wiles A.A., Zhang X., Fitzpatrick B., Long D.L., Macgregor S.A., Cooke G. (2016). Redox-mediated reactions of vinylferrocene: Toward redox auxiliaries. Dalton Trans..

[37] Chen L., Compton R.G. (2019). Reference Electrodes for Electrochemical Sensors Based on Redox Couples Immobilized within Nafion Films. ACS Sens..

[38] Muller S., Lee W., Song J.Y., Kang E., Joo J.M. (2022). Nondirected Pd-catalyzed aerobic C-H alkenylation of ruthenocene and ferrocene. Chem. Commun..

[39] Pi C., Li Y., Cui X., Zhang H., Han Y., Wu Y. (2013). Redox of ferrocene controlled asymmetric dehydrogenative Heck reaction via palladium-catalyzed dual C–H bond activation. Chem. Sci..

[40] Lou S.J., Zhuo Q., Nishiura M., Luo G., Hou Z. (2021). Enantioselective C-H Alkenylation of Ferrocenes with Alkynes by Half-Sandwich Scandium Catalyst. J. Am. Chem. Soc..

[41] Jia J., Cui Y., Li Y., Sheng W., Han L., Gao J. (2013). Synthesis, third-order nonlinear optical properties and theoretical analysis of vinylferrocene derivatives. Dyes Pigm..

[42] Hildebrandt A., Al Khalyfeh K., Schaarschmidt D., Korb M. (2016). Multi-functionalized ferrocenes: –Synthesis and characterization –. J. Organomet. Chem..

[43] Gormen M., Pigeon P., Top S., Hillard E.A., Huche M., Hartinger C.G., de Montigny F., Plamont M.A., Vessieres A., Jaouen G. (2010). Synthesis, cytotoxicity, and COMPARE analysis of ferrocene and [3]ferrocenophane tetrasubstituted olefin derivatives against human cancer cells. ChemMedChem.

[44] Idlas P., Ladaycia A., Nemati F., Lepeltier E., Pigeon P., Jaouen G., Decaudin D., Passirani C. (2022). Ferrocifen stealth LNCs and conventional chemotherapy: A promising combination against multidrug-resistant ovarian adenocarcinoma. Int. J. Pharm..

[45] Zyryanova E.Y., Utepova I.A., Musikhina A.A., Boltneva N.P., Kovaleva N.V., Rudakova E.V., Serebryakova O.G., Makhaeva G.F., Kiskin M.A., Lazarev V.F. (2024). 1,1′-Disubstituted azinylferrocenes: Synthesis, antiaggregation and antioxidant activity. Russ. Chem. Bull..

[46] Fayolle C., Pigeon P., Fischer-Durand N., Salmain M., Buriez O., Vessieres A., Labbe E. (2022). Synthesis, Electrochemical and Fluorescence Properties of the First Fluorescent Member of the Ferrocifen Family and of Its Oxidized Derivatives. Molecules.

[47] Wang Y., Pigeon P., Li W., Yan J., Dansette P.M., Othman M., McGlinchey M.J., Jaouen G. (2022). Diversity-oriented synthesis and bioactivity evaluation of N-substituted ferrocifen compounds as novel antiproliferative agents against TNBC cancer cells. Eur. J. Med. Chem..

[48] Wang Y., Pigeon P., Top S., Sanz Garcia J., Troufflard C., Ciofini I., McGlinchey M.J., Jaouen G. (2019). Atypical Lone Pair-pi Interaction with Quinone Methides in a Series of Imido-Ferrociphenol Anticancer Drug Candidates. Angew. Chem. Int. Ed. Engl..

[49] Jary W.G., Mahler A.-K., Purkathofer T., Baumgartner J. (2001). A convenient synthesis of E-alkenylferrocenes. J. Organomet. Chem..

[50] Shi R., Wang H., Tang P., Bin Y. (2014). Synthesis of vinylferrocene and the ligand-exchange reaction between its copolymer and carbon nanotubes. Front. Chem. Sci. Eng..

[51] Šebesta R., Plevová K., Mudráková B. (2017). A Practical Three-Step Synthesis of Vinylferrocene. Synthesis.

[52] Chupakhin O.N., Utepova I.A., Kovalev I.S., Rusinov V.L., Starikova Z.A. (2007). Direct C–C Coupling of Ferrocenyllithium and Azaheterocycles by Nucleophilic Substitution of Hydrogen—Synthesis of Mono- and 1,1′-Diazinylferrocenes. Eur. J. Org. Chem..

[53] Musikhina A.A., Serebrennikova P.O., Zabelina O.N., Utepova I.A., Chupakhin O.N. (2022). Advanced Application of Planar Chiral Heterocyclic Ferrocenes. Inorganics.

[54] Chupakhin O.N., Serebrennikova P.O., Utepova I.A., Musikhina A.A., Suvorova A.I., Paznikova Y.A. (2020). Two approaches to the synthesis of planar chiral quinolinyl cymantrene. Russ. Chem. Bull..

[55] Musikhina A.A., Utepova I.A., Chupakhin O.N., Charushin V.N., Slepukhin P.A. (2018). Transition metal-free regioselective cross-coupling of azine N-oxides with cymantrenyl lithium. J. Organomet. Chem..

[56] Utepova I.A., Serebrennikova P.O., Streltsova M.S., Musikhina A.A., Fedorchenko T.G., Chupakhin O.N., Antonchick A.P. (2018). Enantiomerically Enriched 1,2-P,N-Bidentate Ferrocenyl Ligands for 1,3-Dipolar Cycloaddition and Transfer Hydrogenation Reactions. Molecules.

[57] Utepova I.A., Chupakhin O.N., Serebrennikova P.O., Musikhina A.A., Charushin V.N. (2014). Two approaches in the synthesis of planar chiral azinylferrocenes. J. Org. Chem..

[58] Musikhina A.A., Utepova I.A., Serebrennikova P.O., Chupakhin O.N., Charushin V.N. (2013). Synthesis of chiral ferrocenylazines. Negishi cross-coupling or S N H reactions?. Russ. J. Org. Chem..

[59] Musikhina A.A., Utepova I.A., Chupakhin O.N., Suvorova A.I., Zyryanova E.Y. (2020). Regioselective synthesis of 1-azinyl-1′-isopropenylferrocenes. Mend. Commun..

[60] Miller E.J., Weigelt C.A., Serth J.A., Rusyid R., Brenner J., Luck L.A., Godlewski M. (1992). The Wittig reaction in the generation of organometallic compounds containing alkenes as side groups. J. Organomet. Chem..

[61] Makhaeva G.F., Rudakova E.V., Kovaleva N.V., Lushchekina S.V., Boltneva N.P., Proshin A.N., Shchegolkov E.V., Burgart Y.V., Saloutin V.I. (2019). Cholinesterase and carboxylesterase inhibitors as pharmacological agents. Russ. Chem. Bull..

[62] Makhaeva G.F., Radchenko E.V., Palyulin V.A., Rudakova E.V., Aksinenko A.Y., Sokolov V.B., Zefirov N.S., Richardson R.J. (2013). Organophosphorus compound esterase profiles as predictors of therapeutic and toxic effects. Chem. Biol. Interact..

[63] Makhaeva G.F., Rudakova E.V., Serebryakova O.G., Aksinenko A.Y., Lushchekina S.V., Bachurin S.O., Richardson R.J. (2016). Esterase profiles of organophosphorus compounds in vitro predict their behavior in vivo. Chem. Biol. Interact..

[64] Shesterenko Y., Shesterenko Y., Romanovska I., Semenishyn N., Smola S., Dekina S. (2024). Pig Liver Cytosolic Carboxylesterase 1 and Its Inhibition by Remdesivir and Sofosbuvir. ChemistrySelect.

[65] Taylor P., Lappi S. (1975). Interaction of fluorescence probes with acetylcholinesterase. The site and specificity of propidium binding. Biochemistry.

[66] De Ferrari G.V., Canales M.A., Shin I., Weiner L.M., Silman I., Inestrosa N.C. (2001). A structural motif of acetylcholinesterase that promotes amyloid beta-peptide fibril formation. Biochemistry.

[67] Arce M.P., Rodriguez-Franco M.I., Gonzalez-Munoz G.C., Perez C., Lopez B., Villarroya M., Lopez M.G., Garcia A.G., Conde S. (2009). Neuroprotective and cholinergic properties of multifunctional glutamic acid derivatives for the treatment of Alzheimer’s disease. J. Med. Chem..

[68] Bartolini M., Bertucci C., Cavrini V., Andrisano V. (2003). β-Amyloid aggregation induced by human acetylcholinesterase: Inhibition studies. Biochem. Pharmacol..

[69] Marco-Contelles J., Leon R., de los Rios C., Garcia A.G., Lopez M.G., Villarroya M. (2006). New multipotent tetracyclic tacrines with neuroprotective activity. Bioorg. Med. Chem..

[70] Makhaeva G.F., Fisenko V.P., Bachurin S.O. (2025). New functions of cholinesterases in the development of Alzheimer’s disease as a target for neuroprotective drugs. Russ. Chem. Bull..

[71] Keshet B., Gray J.J., Good T.A. (2010). Structurally distinct toxicity inhibitors bind at common loci on beta-amyloid fibril. Protein Sci..

[72] Afzal W., Afshan H., Inam J., Farman S., Khurshid B. (2025). Targeting β-amyloid in Alzheimer’s disease: A molecular dynamics study of myricetin as a natural inhibitor. Pak. J. Med. Cardiol. Rev..

[73] Itoh S.G., Yagi-Utsumi M., Kato K., Okumura H. (2022). Key Residue for Aggregation of Amyloid-beta Peptides. ACS Chem. Neurosci..

[74] Naldi M., Fiori J., Pistolozzi M., Drake A.F., Bertucci C., Wu R., Mlynarczyk K., Filipek S., De Simone A., Andrisano V. (2012). Amyloid beta-peptide 25–35 self-assembly and its inhibition: A model undecapeptide system to gain atomistic and secondary structure details of the Alzheimer’s disease process and treatment. ACS Chem. Neurosci..

[75] Ono K., Li L., Takamura Y., Yoshiike Y., Zhu L., Han F., Mao X., Ikeda T., Takasaki J., Nishijo H. (2012). Phenolic compounds prevent amyloid beta-protein oligomerization and synaptic dysfunction by site-specific binding. J. Biol. Chem..

[76] ByteDance A.M.L.A.I.S.T., Chen X., Zhang Y., Lu C., Ma W., Guan J., Gong C., Yang J., Zhang H., Zhang K. (2025). Protenix—Advancing Structure Prediction Through a Comprehensive AlphaFold3 Reproduction. bioRxiv.

[77] Crescenzi O., Tomaselli S., Guerrini R., Salvadori S., D’Ursi A.M., Temussi P.A., Picone D. (2002). Solution structure of the Alzheimer amyloid beta-peptide (1-42) in an apolar microenvironment. Similarity with a virus fusion domain. Eur. J. Biochem..

[78] Santoro A., Grimaldi M., Buonocore M., Stillitano I., D’Ursi A.M. (2021). Exploring the Early Stages of the Amyloid Abeta(1-42) Peptide Aggregation Process: An NMR Study. Pharmaceuticals.

[79] Makhaeva G.F., Kovaleva N.V., Rudakova E.V., Boltneva N.P., Grishchenko M.V., Lushchekina S.V., Astakhova T.Y., Serebryakova O.G., Timokhina E.N., Zhilina E.F. (2023). Conjugates of Tacrine and Salicylic Acid Derivatives as New Promising Multitarget Agents for Alzheimer’s Disease. Int. J. Mol. Sci..

[80] Makhaeva G.F., Grishchenko M.V., Kovaleva N.V., Boltneva N.P., Rudakova E.V., Astakhova T.Y., Timokhina E.N., Pronkin P.G., Lushchekina S.V., Khudina O.G. (2025). Conjugates of amiridine and salicylic derivatives as promising multifunctional CNS agents for potential treatment of Alzheimer’s disease. Arch. Pharm..

[81] Di Cera E. (2020). Mechanisms of ligand binding. Biophys. Rev..

[82] Gupta D. (2015). Methods for determination of antioxidant capacity: A review. Int. J. Pharm. Sci. Res..

[83] Shenderovich I.G. (2019). The Partner Does Matter: The Structure of Heteroaggregates of Acridine Orange in Water. Molecules.

[84] Jaouen G., Top S. (2013). The Ferrocifen Family as Potent and Selective Antitumor Compounds: Mechanisms of Action. Advances in Organometallic Chemistry and Catalysis.

[85] Jaouen G., Vessieres A., Top S. (2015). Ferrocifen type anti cancer drugs. Chem. Soc. Rev..

[86] Mosmann T. (1983). Rapid colorimetric assay for cellular growth and survival: Application to proliferation and cytotoxicity assays. J Immunol. Methods.

[87] Fujii S. (2016). Expanding the chemical space of hydrophobic pharmacophores: The role of hydrophobic substructures in the development of novel transcription modulators. MedChemComm.

[88] Pigeon P., Gormen M., Kowalski K., Muller-Bunz H., McGlinchey M.J., Top S., Jaouen G. (2014). Atypical McMurry cross-coupling reactions leading to a new series of potent antiproliferative compounds bearing the key [ferrocenyl-ene-phenol] motif. Molecules.

[89] Borys A.M. (2023). An Illustrated Guide to Schlenk Line Techniques. Organometallics.

[90] Gao J., Martin A., Yatvin J., White E., Locklin J. (2016). Permanently grafted icephobic nanocomposites with high abrasion resistance. J. Mater. Chem. A.

[91] Drouin B.J., Lavaty T.G., Cassak P.A., Kukolich S.G. (1997). Measurements of structural and quadrupole coupling parameters for bromoferrocene using microwave spectroscopy. J. Chem. Phys..

[92] Makhaeva G.F., Lushchekina S.V., Boltneva N.P., Serebryakova O.G., Rudakova E.V., Ustyugov A.A., Bachurin S.O., Shchepochkin A.V., Chupakhin O.N., Charushin V.N. (2017). 9-Substituted acridine derivatives as acetylcholinesterase and butyrylcholinesterase inhibitors possessing antioxidant activity for Alzheimer’s disease treatment. Bioorg. Med. Chem..

[93] Taylor P., Lwebuga-Mukasa J., Lappi S., Rademacher J. (1974). Propidium—A fluorescence probe for a peripheral anionic site on acetylcholinesterase. Mol. Pharmacol..

[94] Konagurthu A.S., Whisstock J.C., Stuckey P.J., Lesk A.M. (2006). MUSTANG: A multiple structural alignment algorithm. Proteins.

[95] Makhaeva G.F., Kovaleva N.V., Rudakova E.V., Boltneva N.P., Lushchekina S.V., Faingold I.I., Poletaeva D.A., Soldatova Y.V., Kotelnikova R.A., Serkov I.V. (2020). New Multifunctional Agents Based on Conjugates of 4-Amino-2,3-polymethylenequinoline and Butylated Hydroxytoluene for Alzheimer’s Disease Treatment. Molecules.

[96] LeVine H. (1999). Quantification of beta-sheet amyloid fibril structures with thioflavin T. Meth. Enzymol..

[97] Munoz-Ruiz P., Rubio L., Garcia-Palomero E., Dorronsoro I., del Monte-Millan M., Valenzuela R., Usan P., de Austria C., Bartolini M., Andrisano V. (2005). Design, synthesis, and biological evaluation of dual binding site acetylcholinesterase inhibitors: New disease-modifying agents for Alzheimer’s disease. J. Med. Chem..

[98] Re R., Pellegrini N., Proteggente A., Pannala A., Yang M., Rice-Evans C. (1999). Antioxidant activity applying an improved ABTS radical cation decolorization assay. Free Radic. Biol. Med..

[99] Makhaeva G.F., Elkina N.A., Shchegolkov E.V., Boltneva N.P., Lushchekina S.V., Serebryakova O.G., Rudakova E.V., Kovaleva N.V., Radchenko E.V., Palyulin V.A. (2019). Synthesis, molecular docking, and biological evaluation of 3-oxo-2-tolylhydrazinylidene-4,4,4-trifluorobutanoates bearing higher and natural alcohol moieties as new selective carboxylesterase inhibitors. Bioorg. Chem..

[100] Benzie I.F., Strain J.J. (1996). The ferric reducing ability of plasma (FRAP) as a measure of “antioxidant power”: The FRAP assay. Anal. Biochem..

[101] Benzie I.F., Strain J.J. (1999). Ferric reducing/antioxidant power assay: Direct measure of total antioxidant activity of biological fluids and modified version for simultaneous measurement of total antioxidant power and ascorbic acid concentration. Meth. Enzymol..

[102] Dutysheva E.A., Kuznetcova L.S., Utepova I.A., Margulis B.A., Guzhova I.V., Lazarev V.F. (2025). Induction of Chaperone Synthesis in Human Neuronal Cells Blocks Oxidative Stress-Induced Aging. Acta Naturae.

[103] (2025). PubChem. https://pubchem.ncbi.nlm.nih.gov/.

[104] Ozvoldik K., Stockner T., Krieger E. (2023). YASARA Model-Interactive Molecular Modeling from Two Dimensions to Virtual Realities. J. Chem. Inf. Model..

[105] Maier J.A., Martinez C., Kasavajhala K., Wickstrom L., Hauser K.E., Simmerling C. (2015). ff14SB: Improving the Accuracy of Protein Side Chain and Backbone Parameters from ff99SB. J. Chem. Theory Comput..

[106] (2025). CCDC. https://www.ccdc.cam.ac.uk/.

[107] Frisch M.J., Trucks G.W., Schlegel H.B., Scuseria G.E., Robb M.A., Cheeseman J.R., Scalmani G., Barone V., Petersson G.A., Nakatsuji H. (2016). Gaussian 16 Revision C. 01. 2016.

[108] Laikov D.N., Ustynyuk Y.A. (2005). PRIRODA-04: A quantum-chemical program suite. New possibilities in the study of molecular systems with the application of parallel computing. Russ. Chem. Bull..

[109] Becke A.D. (1993). Density-functional thermochemistry. III. The role of exact exchange. J. Chem. Phys..

[110] Petersson G.A., Al-Laham M.A. (1991). A complete basis set model chemistry. II. Open-shell systems and the total energies of the first-row atoms. J. Chem. Phys..

[111] Grimme S., Antony J., Ehrlich S., Krieg H. (2010). A consistent and accurate ab initio parametrization of density functional dispersion correction (DFT-D) for the 94 elements H-Pu. J. Chem. Phys..

[112] Marenich A.V., Cramer C.J., Truhlar D.G. (2009). Universal Solvation Model Based on Solute Electron Density and on a Continuum Model of the Solvent Defined by the Bulk Dielectric Constant and Atomic Surface Tensions. J. Phys. Chem. B.

[113] Hawkins P.C., Skillman A.G., Nicholls A. (2007). Comparison of shape-matching and docking as virtual screening tools. J. Med. Chem..

[114] OpenEye (2025). ROCS 3.8.0.1.

[115] Schrödinger, LLC (2025). The Schrödinger Suite, Including Phase (Used for Volume Calculations) (Release 2025-2).

[116] Eberhardt J., Santos-Martins D., Tillack A.F., Forli S. (2021). AutoDock Vina 1.2.0: New Docking Methods, Expanded Force Field, and Python Bindings. J. Chem. Inf. Model..

[117] Trott O., Olson A.J. (2010). AutoDock Vina: Improving the speed and accuracy of docking with a new scoring function, efficient optimization, and multithreading. J. Comput. Chem..

[118] Lexa K.W., Carlson H.A. (2012). Protein flexibility in docking and surface mapping. Q. Rev. Biophys..

[119] (2025). UniProt. https://www.uniprot.org/.

[120] (2025). NCBI-Protein. https://www.ncbi.nlm.nih.gov/protein/.

[121] (2025). Protenix. https://github.com/bytedance/Protenix.

[122] Krieger E., Dunbrack R.L., Hooft R.W., Krieger B. (2012). Assignment of protonation states in proteins and ligands: Combining pKa prediction with hydrogen bonding network optimization. Methods Mol. Biol..

[123] Chen X., Fang L., Liu J., Zhan C.G. (2012). Reaction pathway and free energy profiles for butyrylcholinesterase-catalyzed hydrolysis of acetylthiocholine. Biochemistry.

[124] Golicnik M., Sinko G., Simeon-Rudolf V., Grubic Z., Stojan J. (2002). Kinetic model of ethopropazine interaction with horse serum butyrylcholinesterase and its docking into the active site. Arch. Biochem. Biophys..

[125] Kovarik Z., Radic Z., Grgas B., Skrinjaric-Spoljar M., Reiner E., Simeon-Rudolf V. (1999). Amino acid residues involved in the interaction of acetylcholinesterase and butyrylcholinesterase with the carbamates Ro 02-0683 and bambuterol, and with terbutaline. Biochim. Biophys. Acta.

[126] Masson P., Froment M.T., Fortier P.L., Visicchio J.E., Bartels C.F., Lockridge O. (1998). Butyrylcholinesterase-catalysed hydrolysis of aspirin, a negatively charged ester, and aspirin-related neutral esters. Biochim. Biophys. Acta.

[127] Moorad D., Chunyuan L., Ashima S., Bhupendra D., Gregory G. (2008). Purification and Determination of the Amino Acid Sequence of Equine Serum Butyrylcholinesterase. Toxicol. Meth..

[128] Rosenberry T.L., Brazzolotto X., Macdonald I.R., Wandhammer M., Trovaslet-Leroy M., Darvesh S., Nachon F. (2017). Comparison of the Binding of Reversible Inhibitors to Human Butyrylcholinesterase and Acetylcholinesterase: A Crystallographic, Kinetic and Calorimetric Study. Molecules.

[129] Bourne Y., Grassi J., Bougis P.E., Marchot P. (1999). Conformational flexibility of the acetylcholinesterase tetramer suggested by x-ray crystallography. J. Biol. Chem..

[130] Salih E., Chishti S.B., Vicedomine P., Cohen S.G., Chiara D.C., Cohen J.B. (1994). Active-site peptides of acetylcholinesterase of Electrophorus electricus: Labelling of His-440 by 1-bromo-[2-14C] pinacolone and Ser-200 by tritiated diisopropyl fluorophosphate. Biochim. Biophys. Acta.

[131] Johnson G., Moore S.W. (2006). The peripheral anionic site of acetylcholinesterase: Structure, functions and potential role in rational drug design. Curr. Pharm. Des..

[132] Shafferman A., Velan B., Ordentlich A., Kronman C., Grosfeld H., Leitner M., Flashner Y., Cohen S., Barak D., Ariel N. (1992). Substrate inhibition of acetylcholinesterase: Residues affecting signal transduction from the surface to the catalytic center. EMBO J..

[133] Wiesner J., Kriz Z., Kuca K., Jun D., Koca J. (2007). Acetylcholinesterases--the structural similarities and differences. J. Enzyme Inhib. Med. Chem..

[134] Wang D., Zou L., Jin Q., Hou J., Ge G., Yang L. (2018). Human carboxylesterases: A comprehensive review. Acta Pharm. Sin. B.

[135] Ireland D., Rabeler C., Rao S., Richardson R.J., Collins E.S. (2025). Distinguishing classes of neuroactive drugs based on computational physicochemical properties and experimental phenotypic profiling in planarians. PLoS ONE.

[136] Zaki M.J., Meira W. (2020). Linear discriminant analysis. Data Mining and Machine Learning: Fundamental Concepts and Algorithms.

[137] (2025). PAST. https://www.nhm.uio.no/english/research/resources/past/.

[138] Lachenbruch P.A., Mickey M.R. (1968). Estimation of Error Rates in Discriminant Analysis. Technometrics.

[139] Haynes W.M. (2016). CRC Handbook of Chemistry and Physics.

[140] Brown H.C., Braude E.A., Nachod F.C. (1955). Determination of Organic Structures by Physical Methods.

[141] Lide D.R. (2009). CRC Handbook of Chemistry and Physics.

[142] https://www.chemicalbook.com/ChemicalProductProperty_EN_CB5195697.htm.

[143] Portenkirchner E., Enengl C., Enengl S., Hinterberger G., Schlager S., Apaydin D., Neugebauer H., Knör G., Sariciftci N.S. (2014). A Comparison of Pyridazine and Pyridine as Electrocatalysts for the Reduction of Carbon Dioxide to Methanol. ChemElectroChem.

[144] Fujiki R., Matsui T., Shigeta Y., Nakano H., Yoshida N. (2021). Recent Developments of Computational Methods for pKa Prediction Based on Electronic Structure Theory with Solvation Models. J.

[145] Wang S., Shen Y., Zhang X., Liu H., Zhang S.-T., Li W., Yang B. (2022). Noncovalent π–π dimerization based on acridine and acid-responsive luminescence switching. Dyes Pigm..

[146] Choudhury R.R., Chitra R. (2010). Stacking interaction between homostacks of simple aromatics and the factors influencing these interactions. CrystEngComm.

[147] Paik D., Lee H., Kim H., Choi J.M. (2022). Thermodynamics of pi-pi Interactions of Benzene and Phenol in Water. Int. J. Mol. Sci..

[148] Srivastava A., Garg A., Das D., Debnath A. (2020). Molecular dynamics simulations of a stacked *π*-conjugated soft material: Binding energy and preferential geometry for self-assembly. Bull. Mater. Sci..

[149] Lessa M.D., Stoyanov S.R., de Carneiro J.W.M., da Costa L.M. (2024). Density functional theory investigation of the contributions of pi-pi stacking and hydrogen bonding with water to the supramolecular aggregation interactions of model asphaltene heterocyclic compounds. J. Mol. Model..

[150] Tachikawa H., Iura R., Kawabata H. (2019). Water-accelerated pi-Stacking Reaction in Benzene Cluster Cation. Sci. Rep..

[151] Pawledzio S., Ziemniak M., Trzybinski D., Arhangelskis M., Makal A., Wozniak K. (2024). Influence of N-protonation on electronic properties of acridine derivatives by quantum crystallography. RSC Adv..

[152] Ilyasov I.R., Beloborodov V.L., Selivanova I.A., Terekhov R.P. (2020). ABTS/PP Decolorization Assay of Antioxidant Capacity Reaction Pathways. Int. J. Mol. Sci..

[153] Bell E.W., Zhang Y. (2019). DockRMSD: An open-source tool for atom mapping and RMSD calculation of symmetric molecules through graph isomorphism. J. Cheminform..

[154] Jung J., Lee B. (2000). Protein structure alignment using environmental profiles. Protein Eng..

