# Supplementary material for: 1-Azinyl-1′-Alkenylferrocenes with Anticholinesterase, Antioxidant, and Antiaggregating Activities as Multifunctional Agents for Potential Treatment of Alzheimer’s Disease
Source: Pharmaceuticals (Basel). 2025 Dec 5;18(12):1862. doi: 10.3390/ph18121862 (PMC12736194; doi:10.3390/ph18121862)
Supplement: Supplementary file 1 [file pharmaceuticals-18-01862-s001.zip › pharmaceuticals-3984004-supplementary/Makhaeva_3984004_Supplemental.docx.pdf]

Supplementary Materials

1-Azinyl-1′-Alkenylferrocenes with Anticholinesterase, Antioxidant, and Antiaggregating Activities as Multifunctional Agents for Potential Treatment of Alzheimer's Disease

Galina F. Makhaeva, Irina A. Utepova, Elena V. Rudakova, Nadezhda V. Kovaleva, Natalia P. Boltneva, Elena Yu. Zyryanova, Alexandra A. Musikhina, Vladimir F. Lazarev, Snezhana A. Vladimirova, Irina V. Guzhova, Ilya N. Ganebnykh, Tatiana Y. Astakhova, Elena N. Timokhina, Oleg N. Chupakhin, Valery N. Charushin and Rudy J. Richardson *

***Correspondence: rjrich@umich.edu (R.J.R.)**

Table of contents

1. NMR ^{1}H Spectra 2
2. NMR ^{13}C Spectra 17
3. HSQC and HMBC Spectra 32
4. IR Spectra 62
5. Protonation state of the studied compounds under experimental conditions 64
6. Dimerization of compounds **1a, 1b**, and **1c** 72
7. Heatmaps of percent overlap of docked ligands in *Ec*BChE, *Ee*AChE, *Hs*Aβ_{42}, and *Ss*CES1 77
8. Validation of molecular docking method 82
9. Reprogramming of mesenchymal stem cells obtained from human dental pulp (MSC-DP) 81

Section S1. NMR ^{1}H Spectra

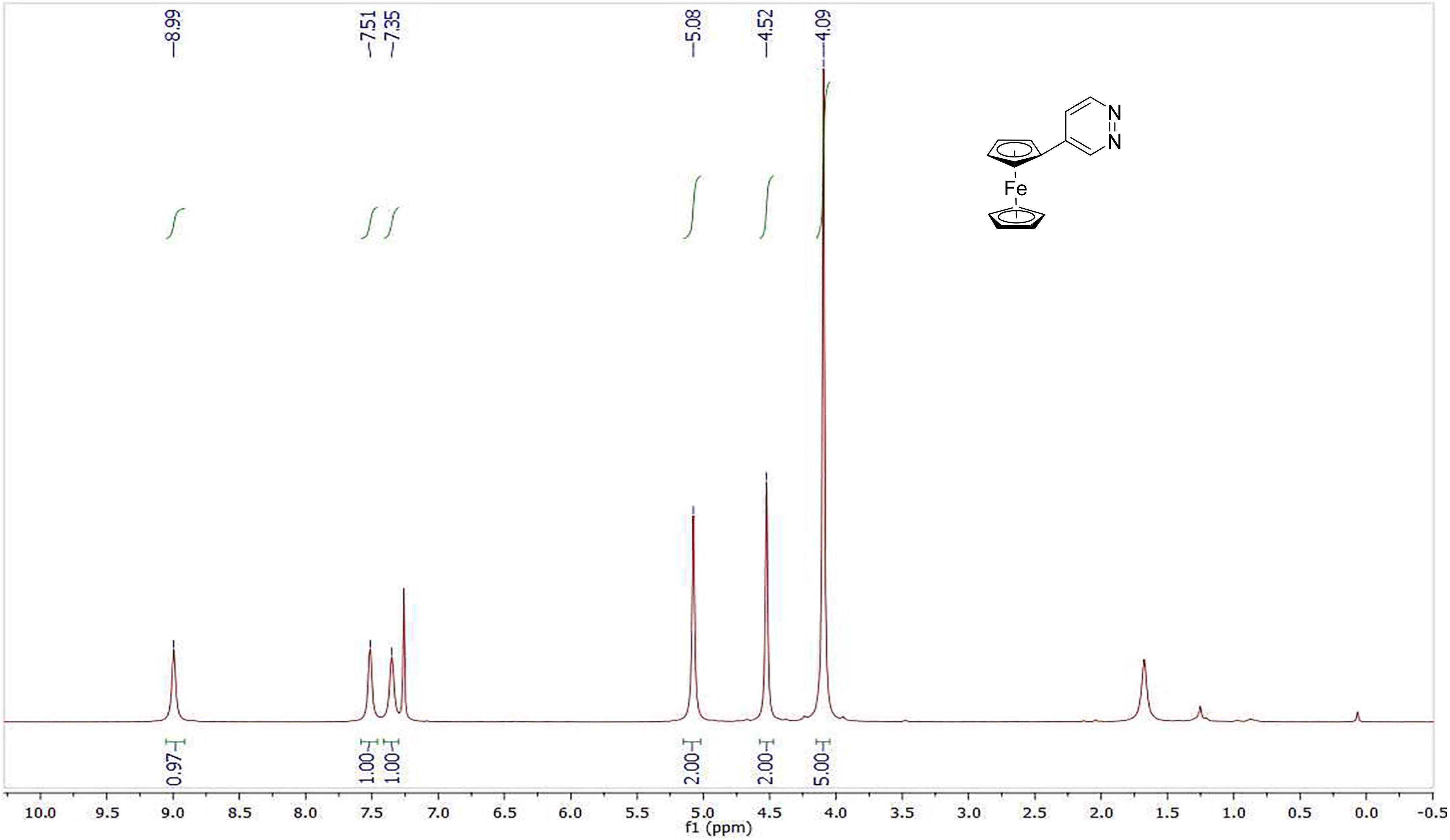

Figure S1.1. ^{1}H NMR (600 MHz) spectrum for 1-(pyridazin-4-yl)ferrocene **(1e)**.

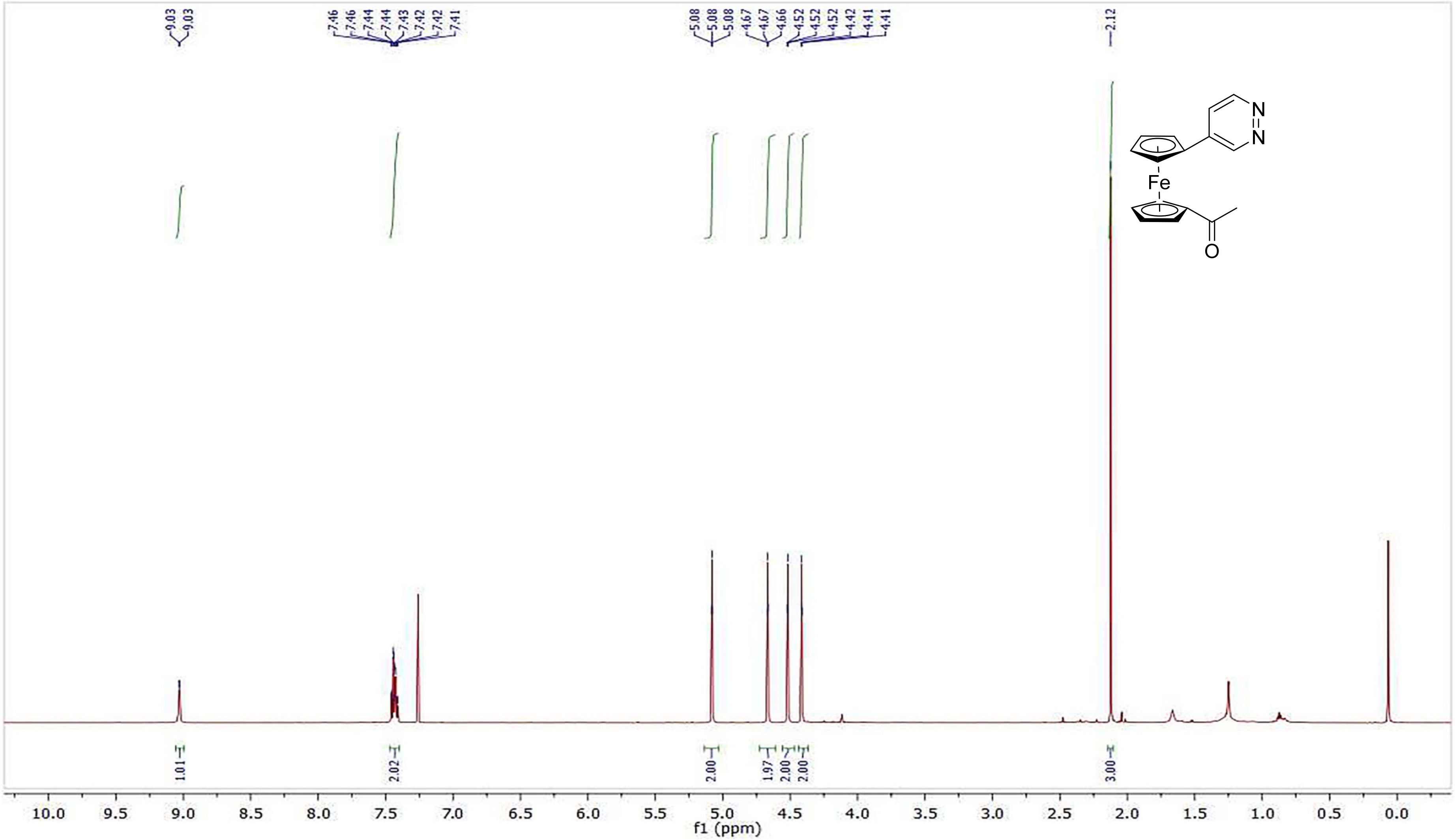

Figure S1.2. ^{1}H NMR (600 MHz) spectrum for 1-acetyl-1'-(pyridazin-4-yl)ferrocene **(2e)**.

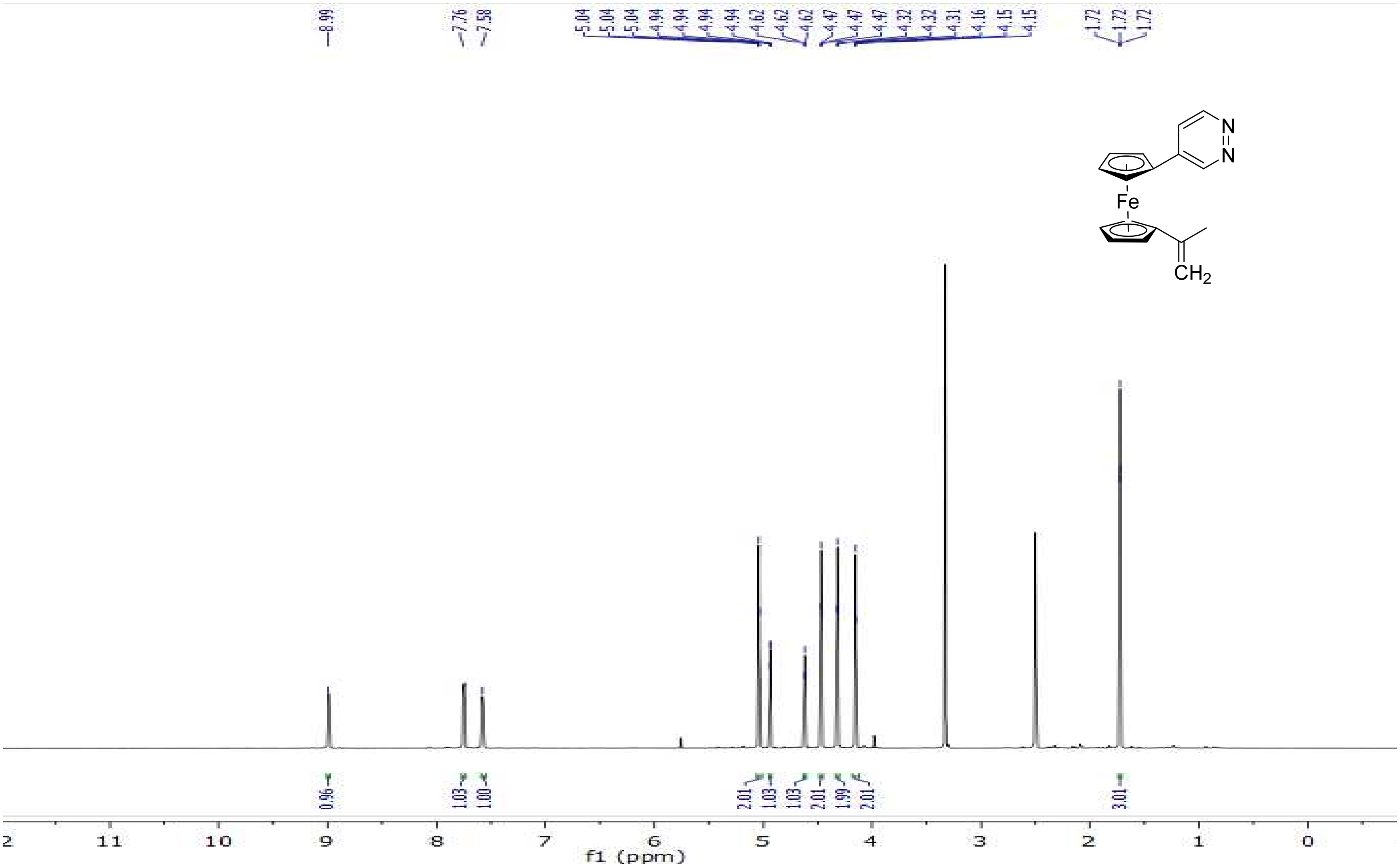

Figure S1.3. ^{1}H NMR (600 MHz) spectrum for 1-isopropenyl-1'-(pyridazin-4-yl)ferrocene **(5e)**.

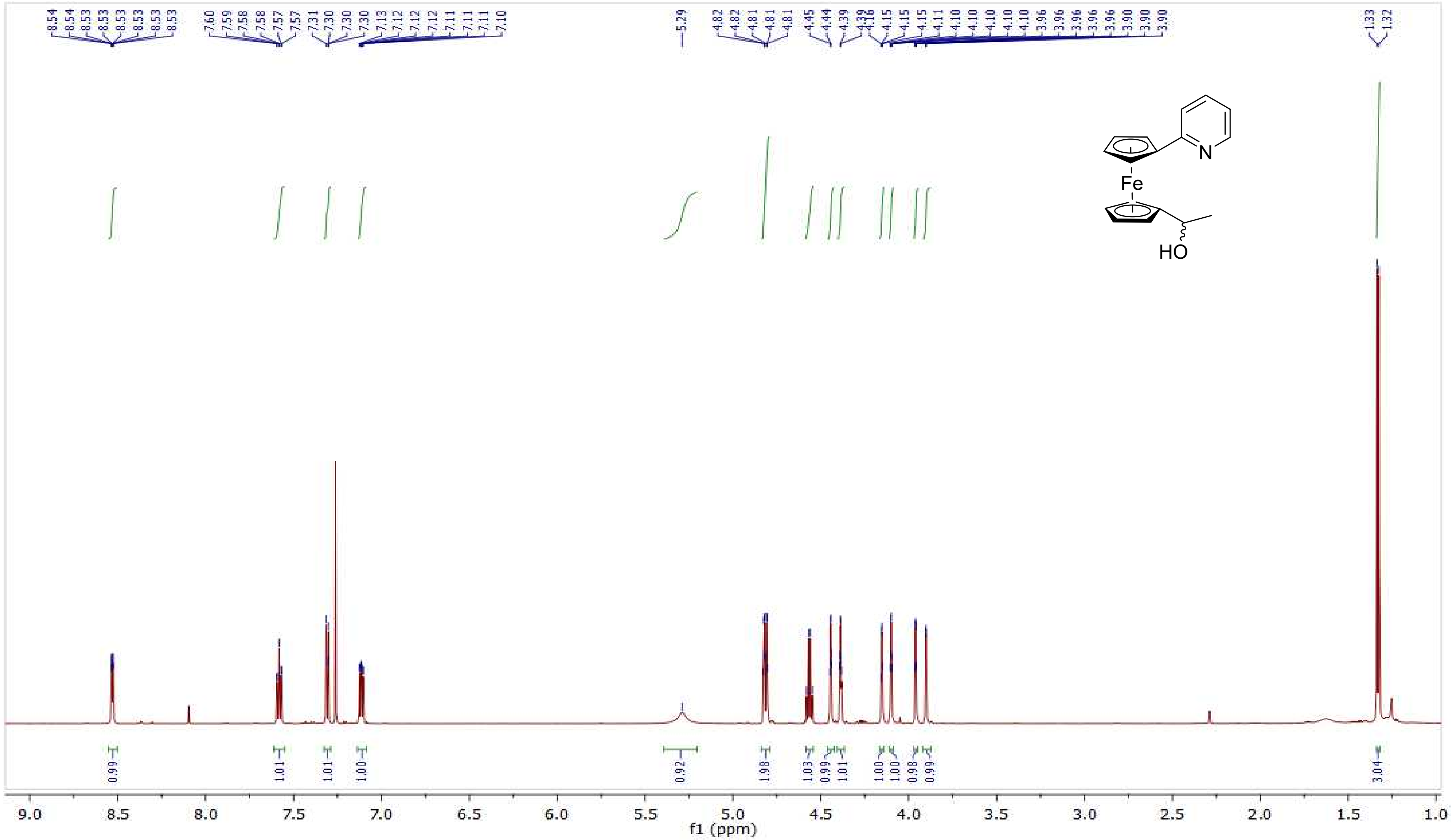

Figure S1.4. ^{1}H NMR (600 MHz) spectrum for 1-(pyridin-2-yl)-1'-(α-hydroxyethyl)ferrocene **(6a)**.

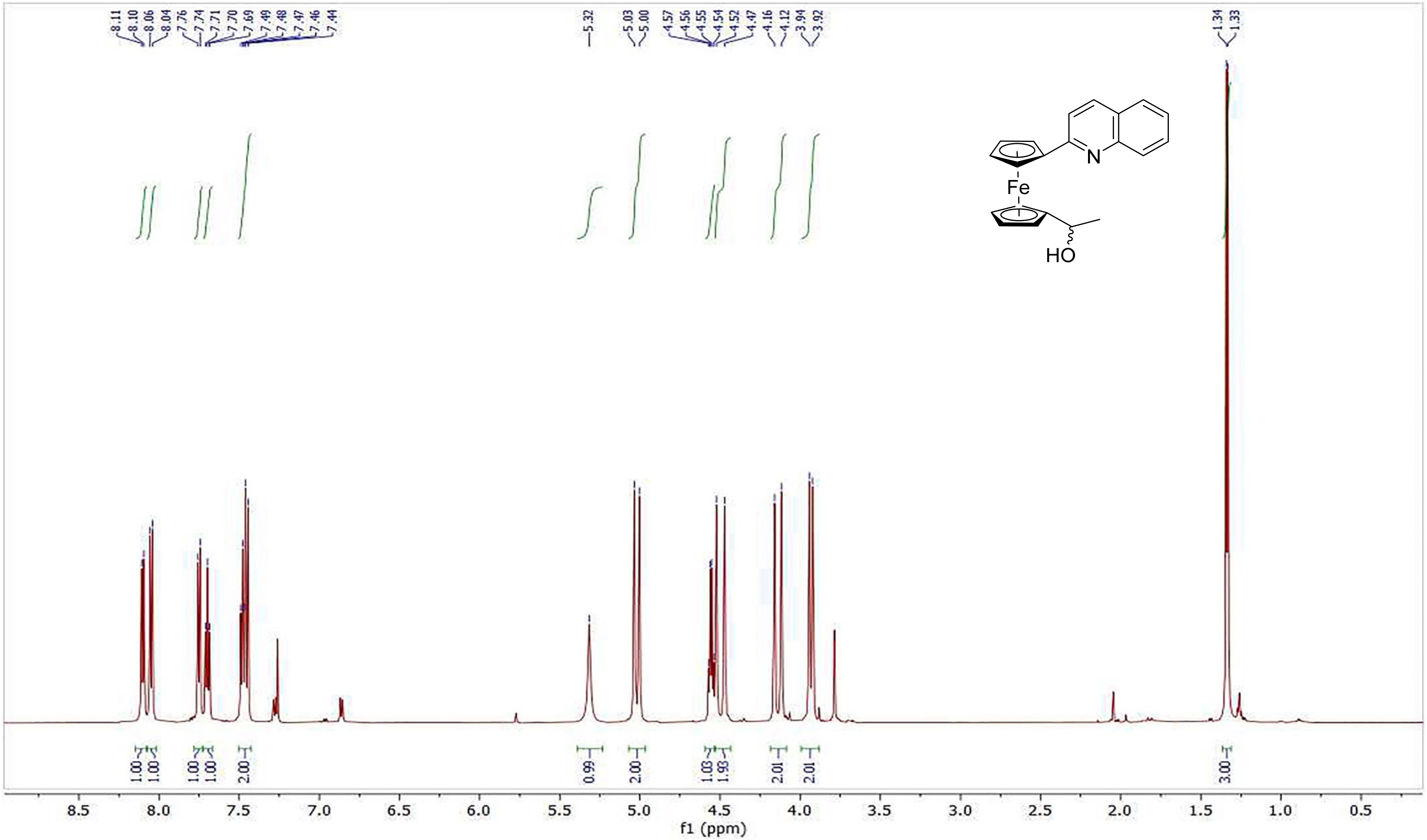

Figure S1.5. ^{1}H NMR (600 MHz) spectrum for 1-(quinolin-2-yl)-1'-(α-hydroxyethyl)ferrocene **(6b)**.

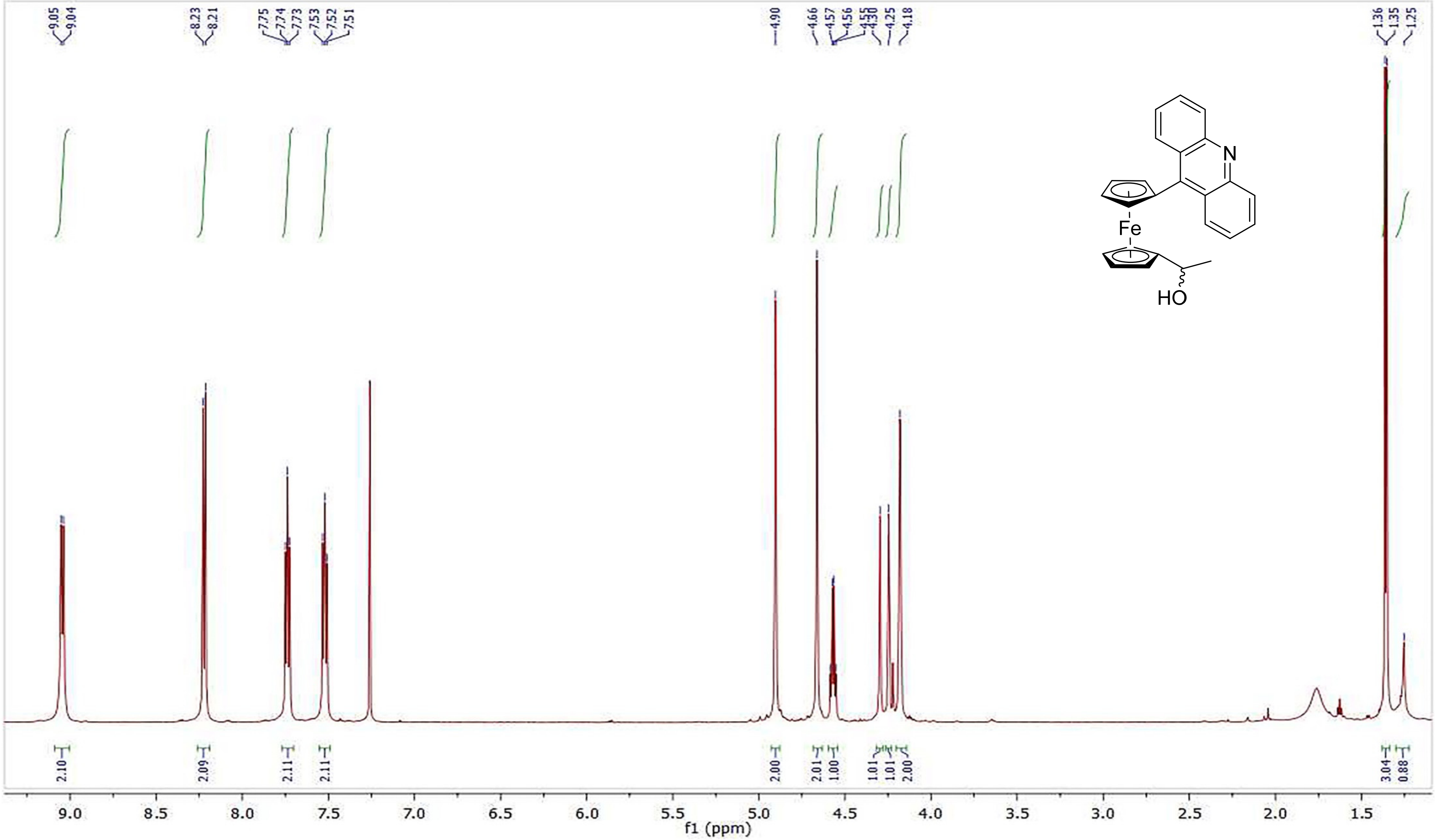

Figure S1.6. ^{1}H NMR (600 MHz) spectrum for 1-(acridin-9-yl)-1'-(α-hydroxyethyl)ferrocene **(6c)**.

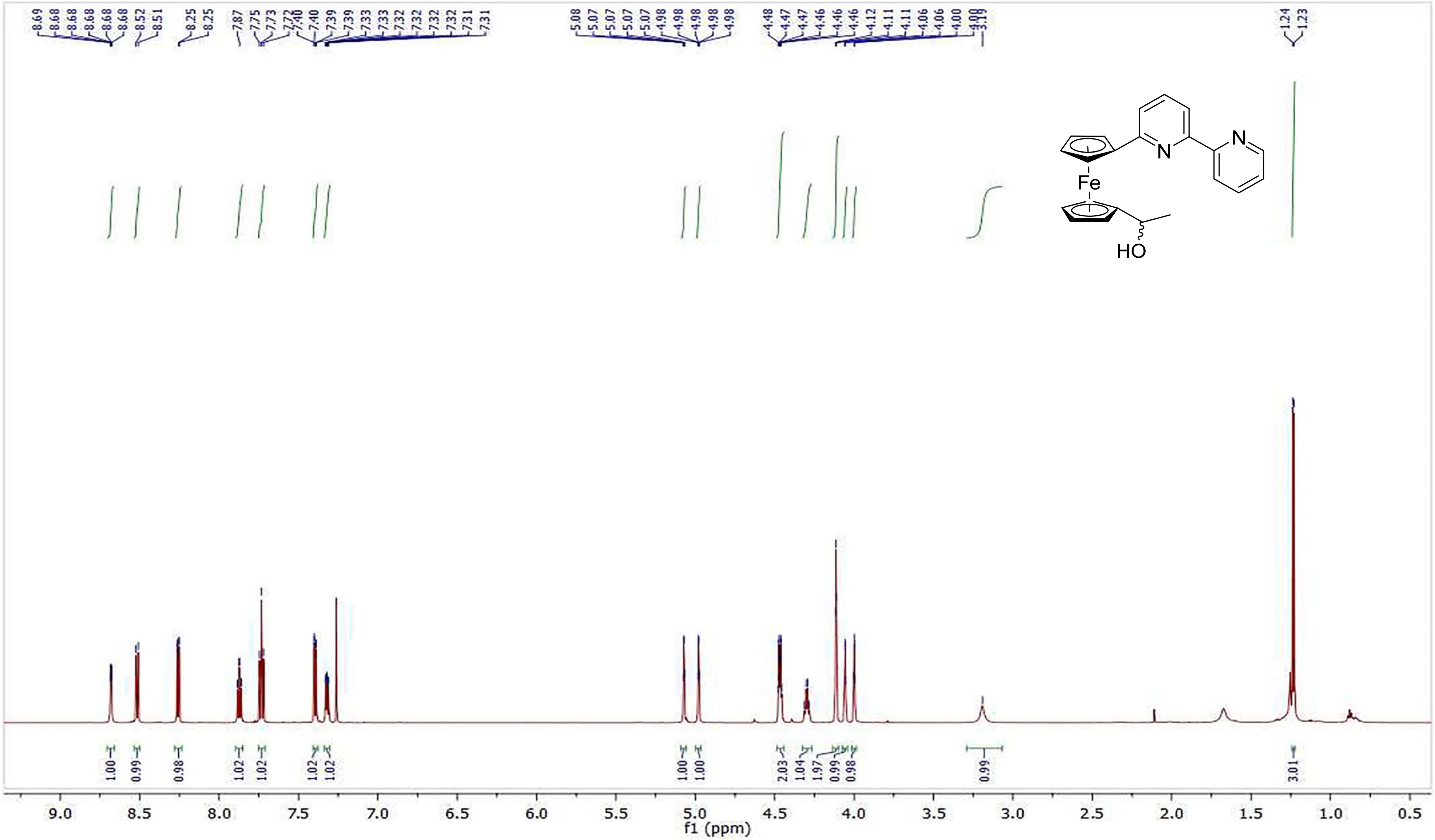

Figure S1.7. ^{1}H NMR (600 MHz) spectrum for 1-(2,2'-bipyridin-6-yl)-1'-(α-hydroxyethyl)ferrocene **(6d)**.

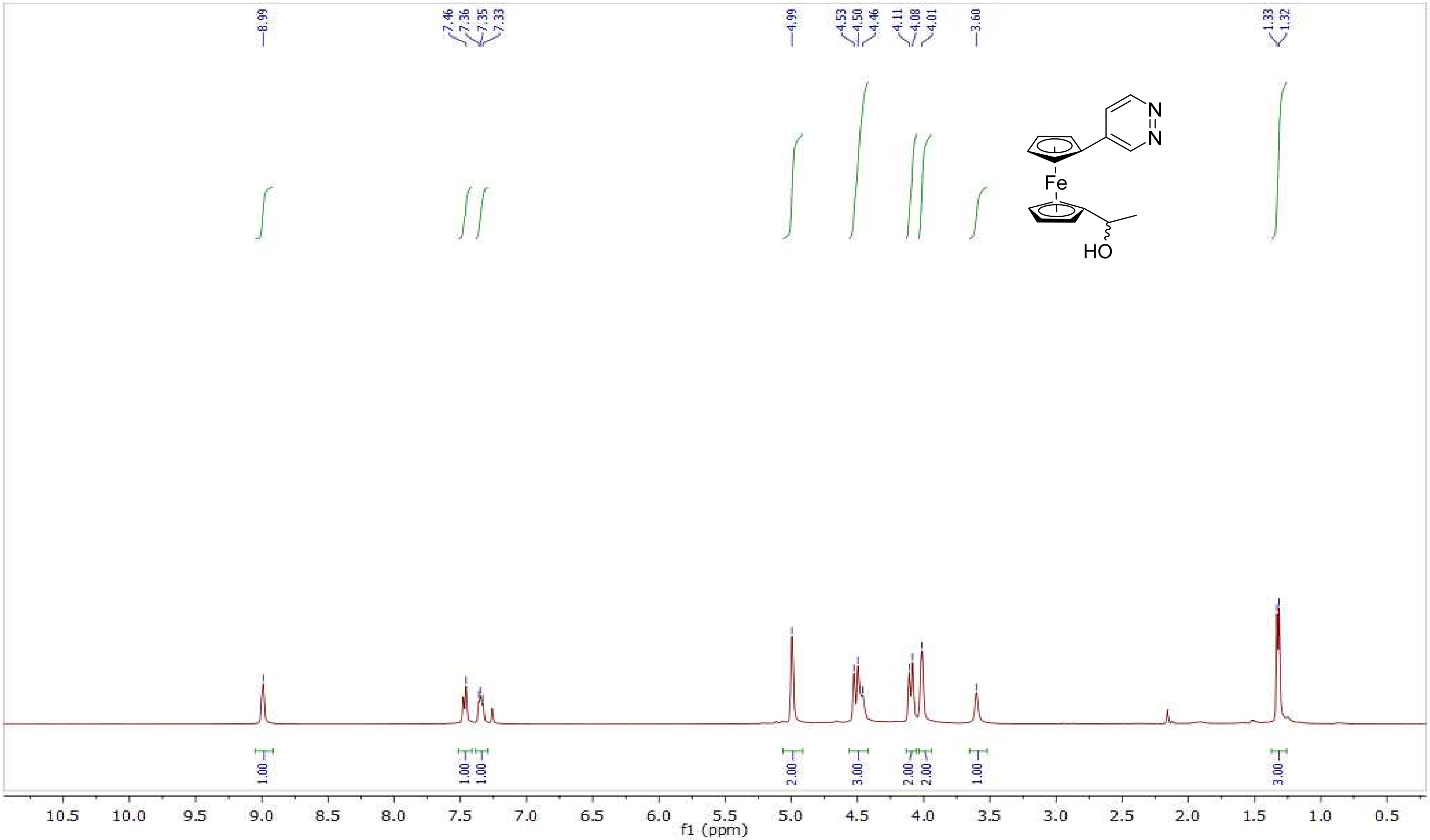

Figure S1.8. ^{1}H NMR (400 MHz) spectrum for 1-(pyridazin-4-yl)-1'-(α-hydroxyethyl)ferrocene **(6e)**.

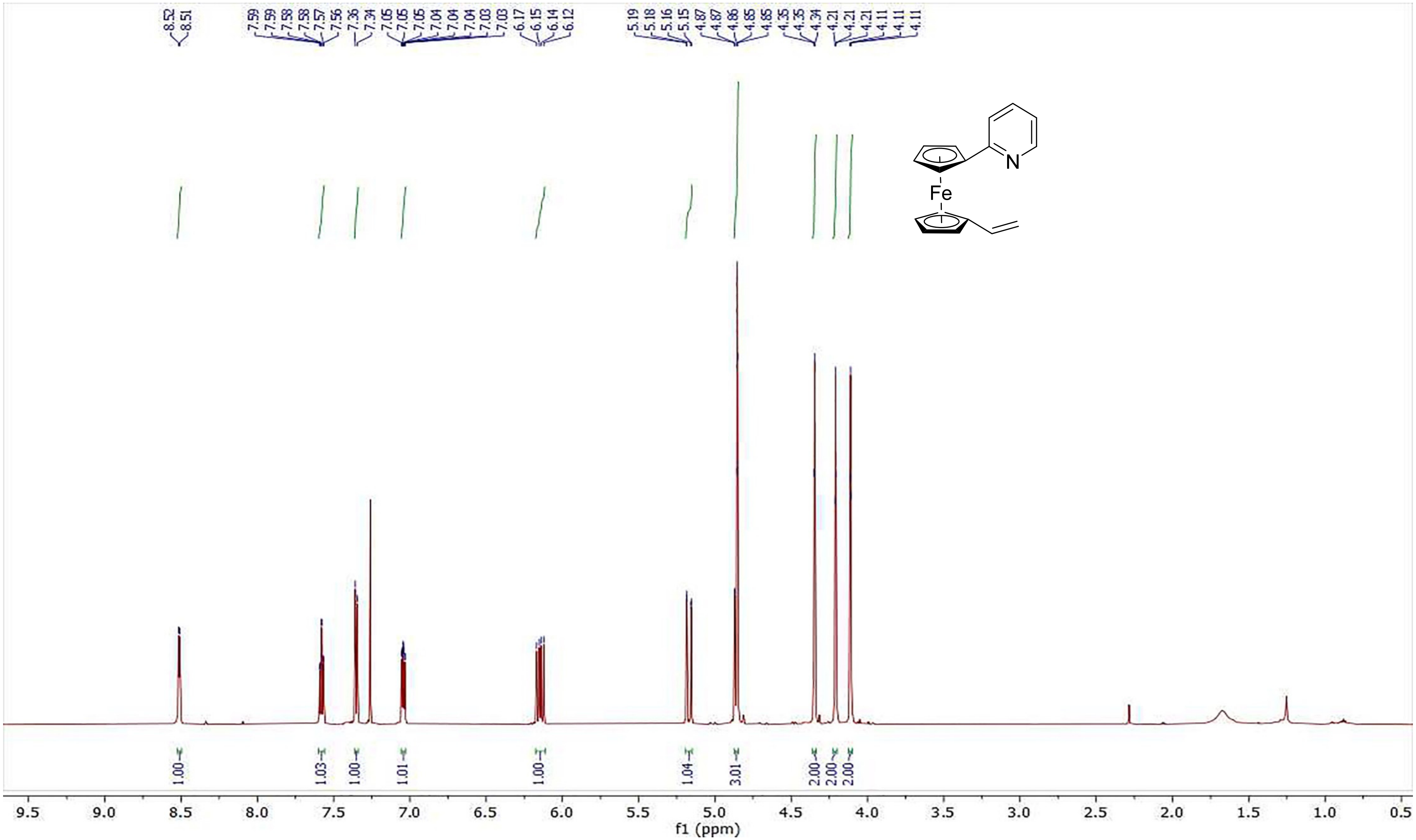

Figure S1.9. ^{1}H NMR (600 MHz) spectrum for 1-(pyridin-2-yl)-1'-vinylferrocene **(7a)**.

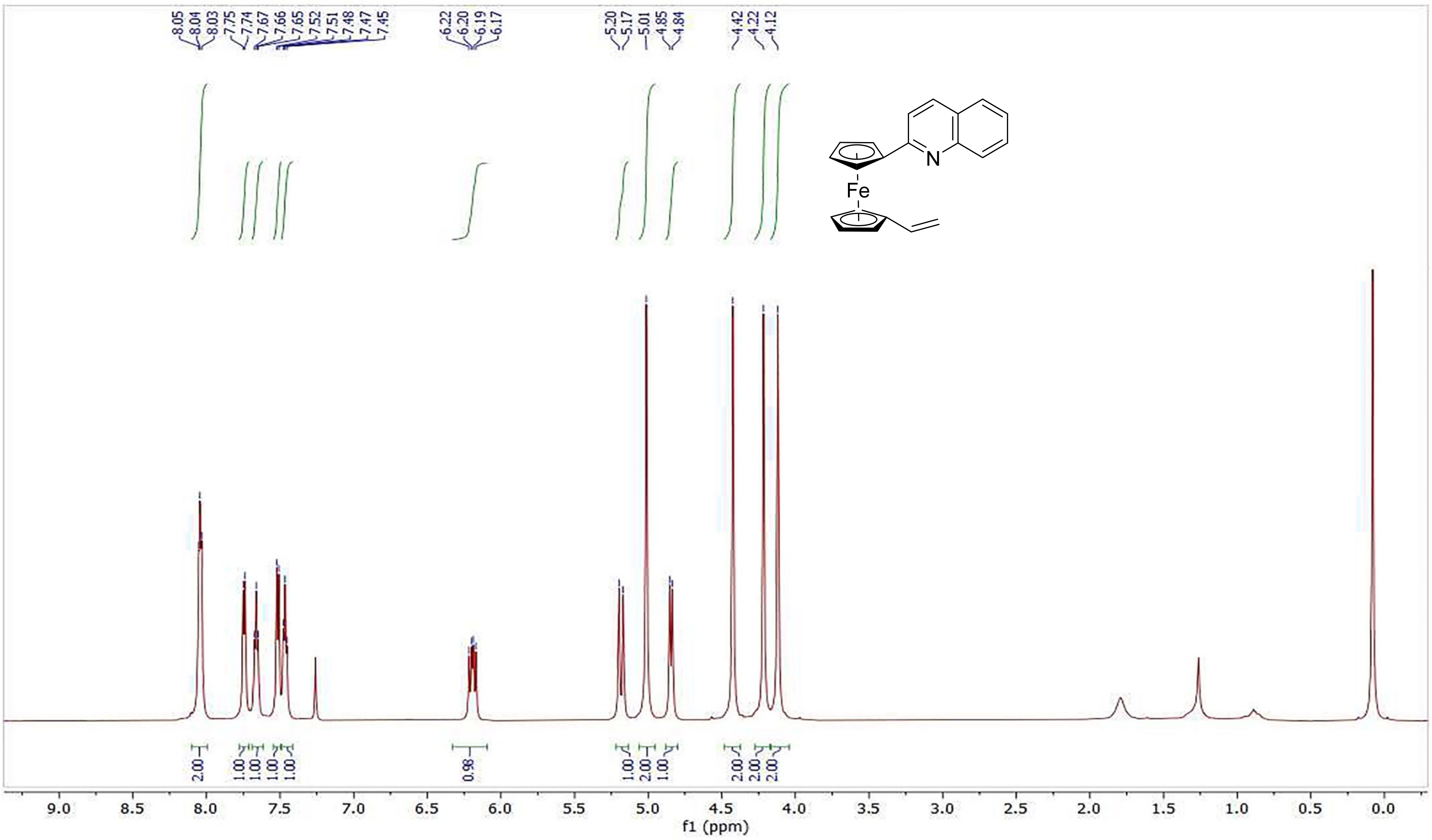

Figure S1.10. ^{1}H NMR (600 MHz) spectrum for 1-(quinolin-2-yl)-1'-vinylferrocene **(7b)**.

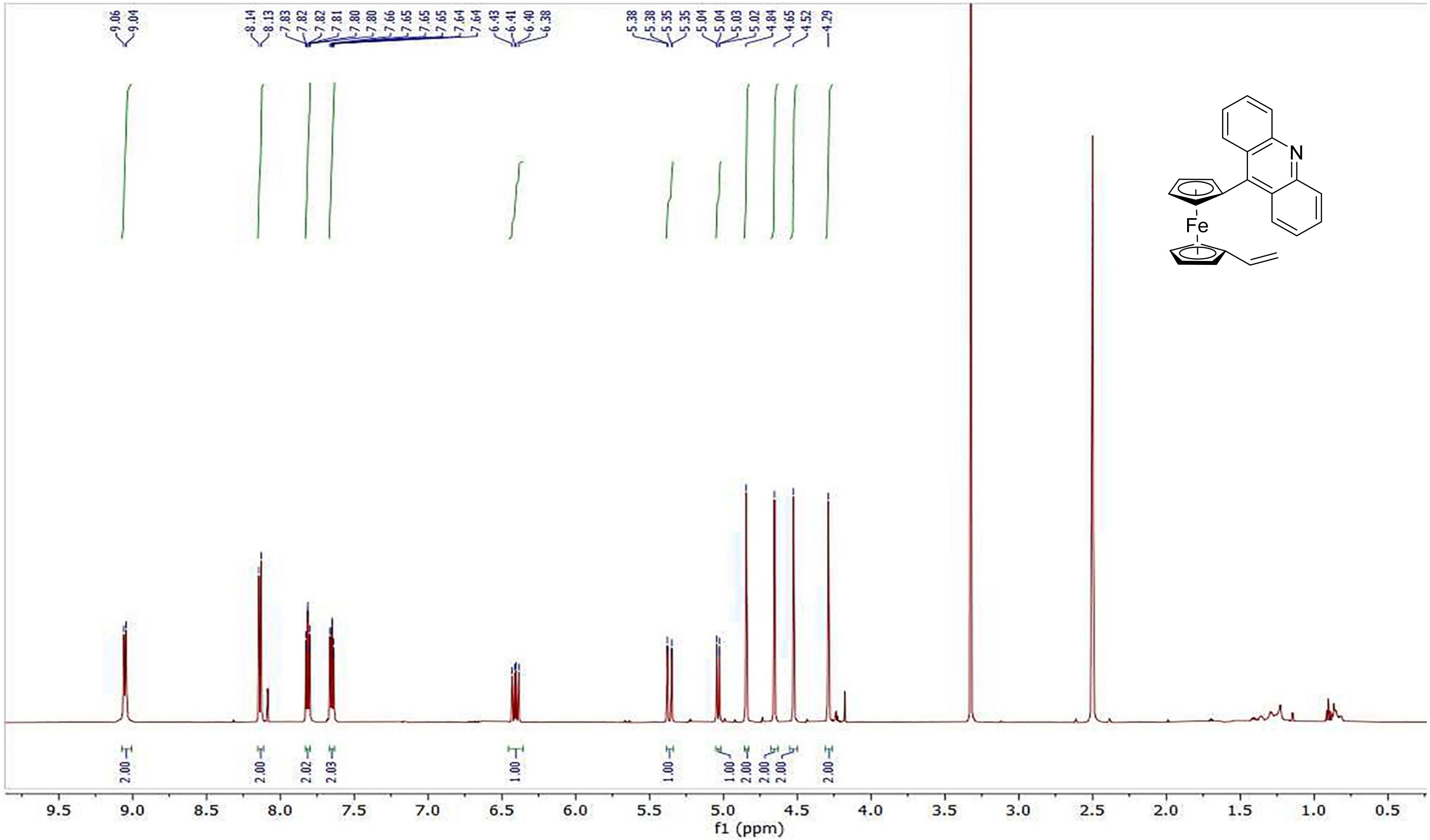

Figure S1.11. ^{1}H NMR (600 MHz) spectrum for 1-(acridin-9-yl)-1'-vinylferrocene **(7c)**.

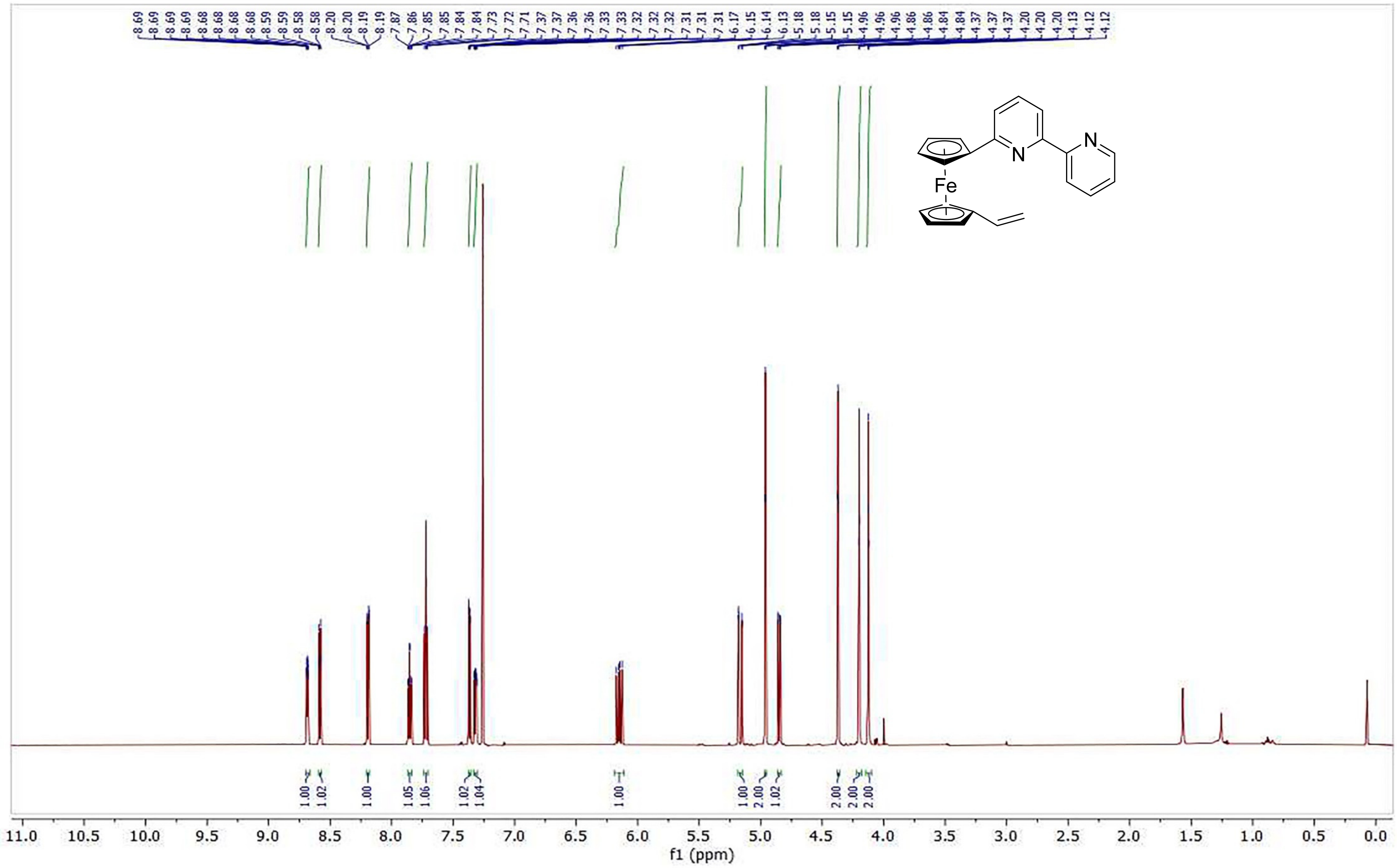

Figure S1.12. ^{1}H NMR (600 MHz) spectrum for 1-(2,2'-bipyridin-6-yl)-1'-vinylferrocene **(7d)**.

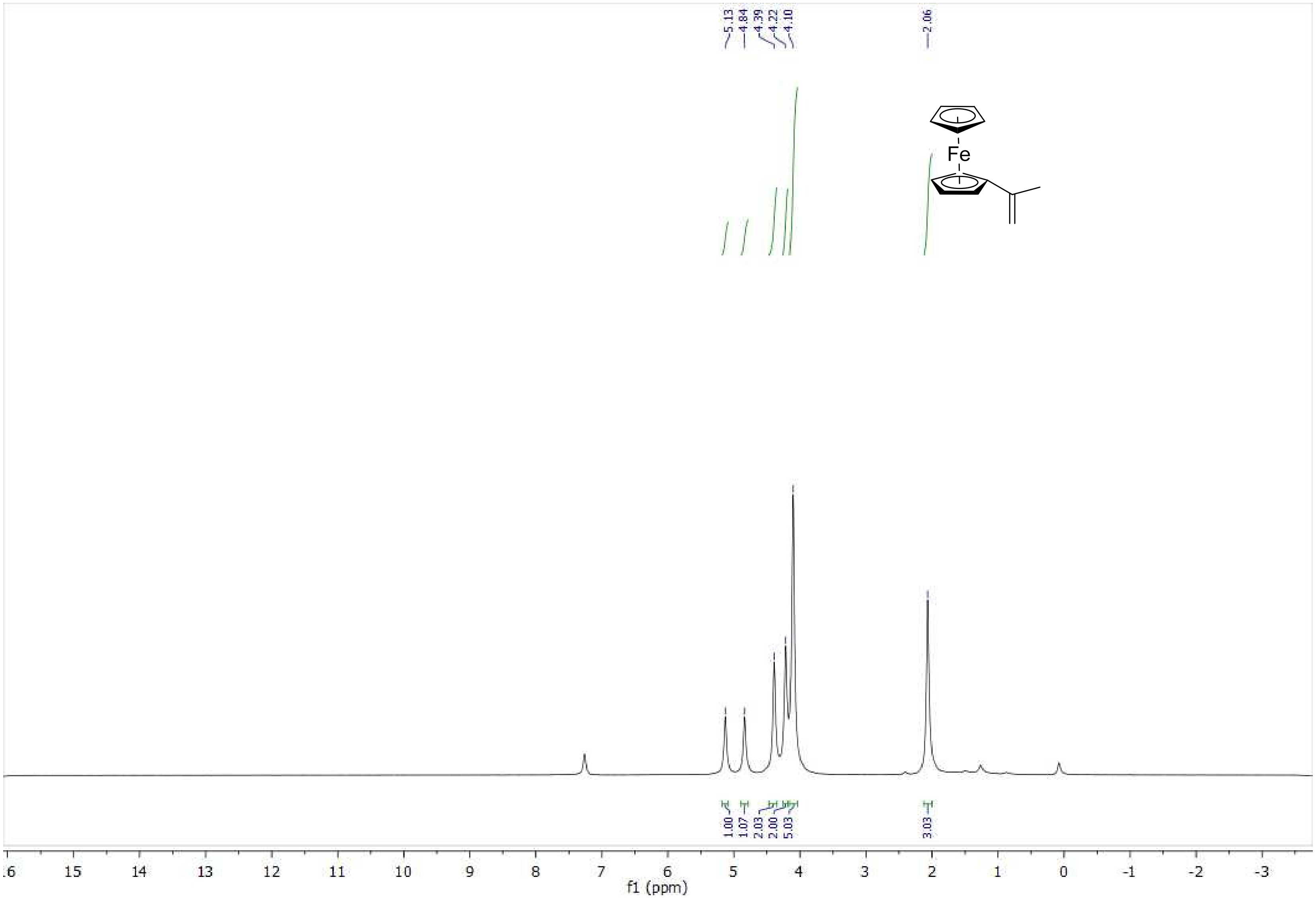

Figure S1.13. ^{1}H NMR (600 MHz) spectrum for isopropenylferrocene **(8)**.

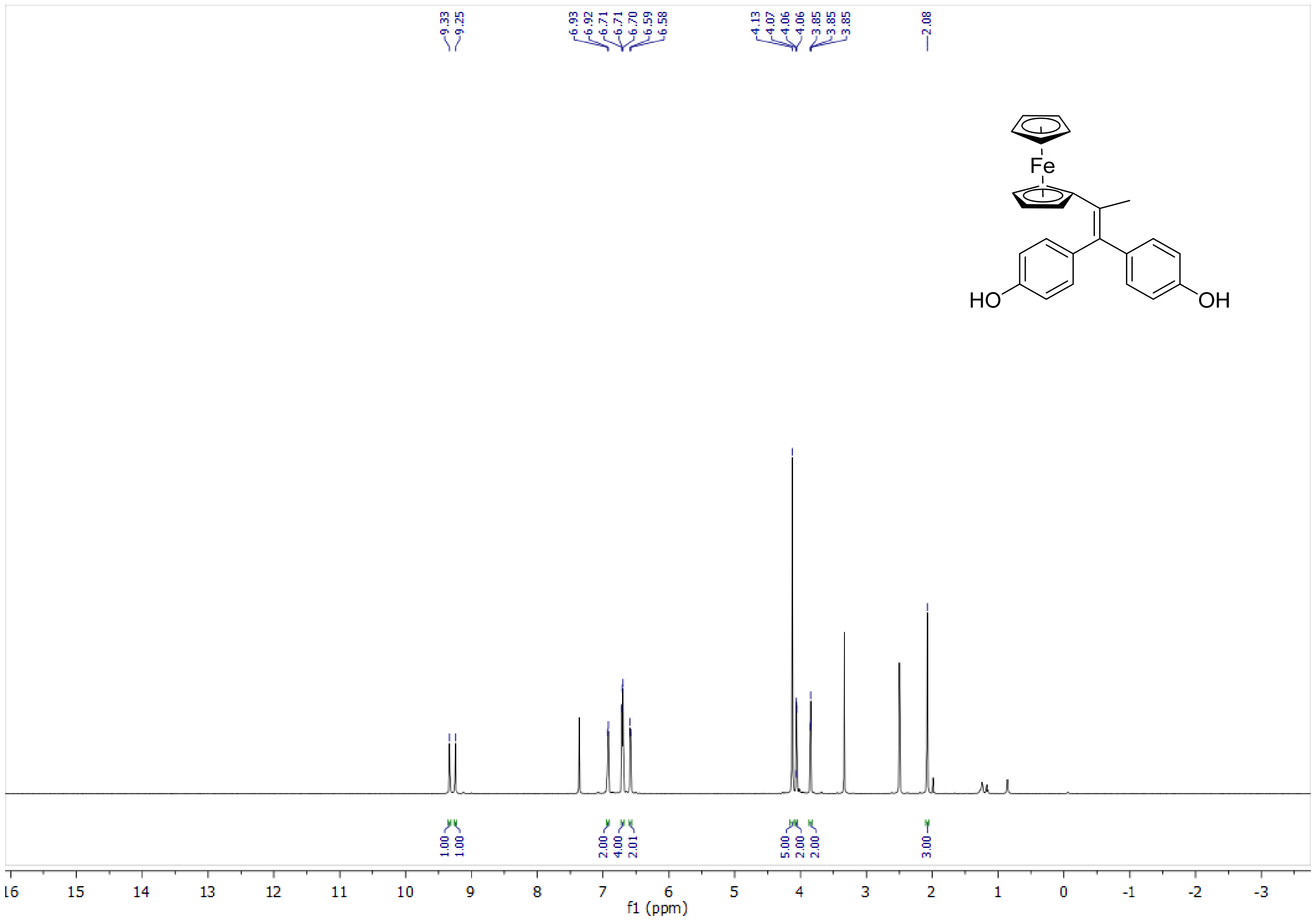

Figure S1.14. ^{1}H NMR (600 MHz) spectrum for 1,1-bis(4-hydroxyphenyl)-2-ferrocenylprop-1-ene **(10)**.

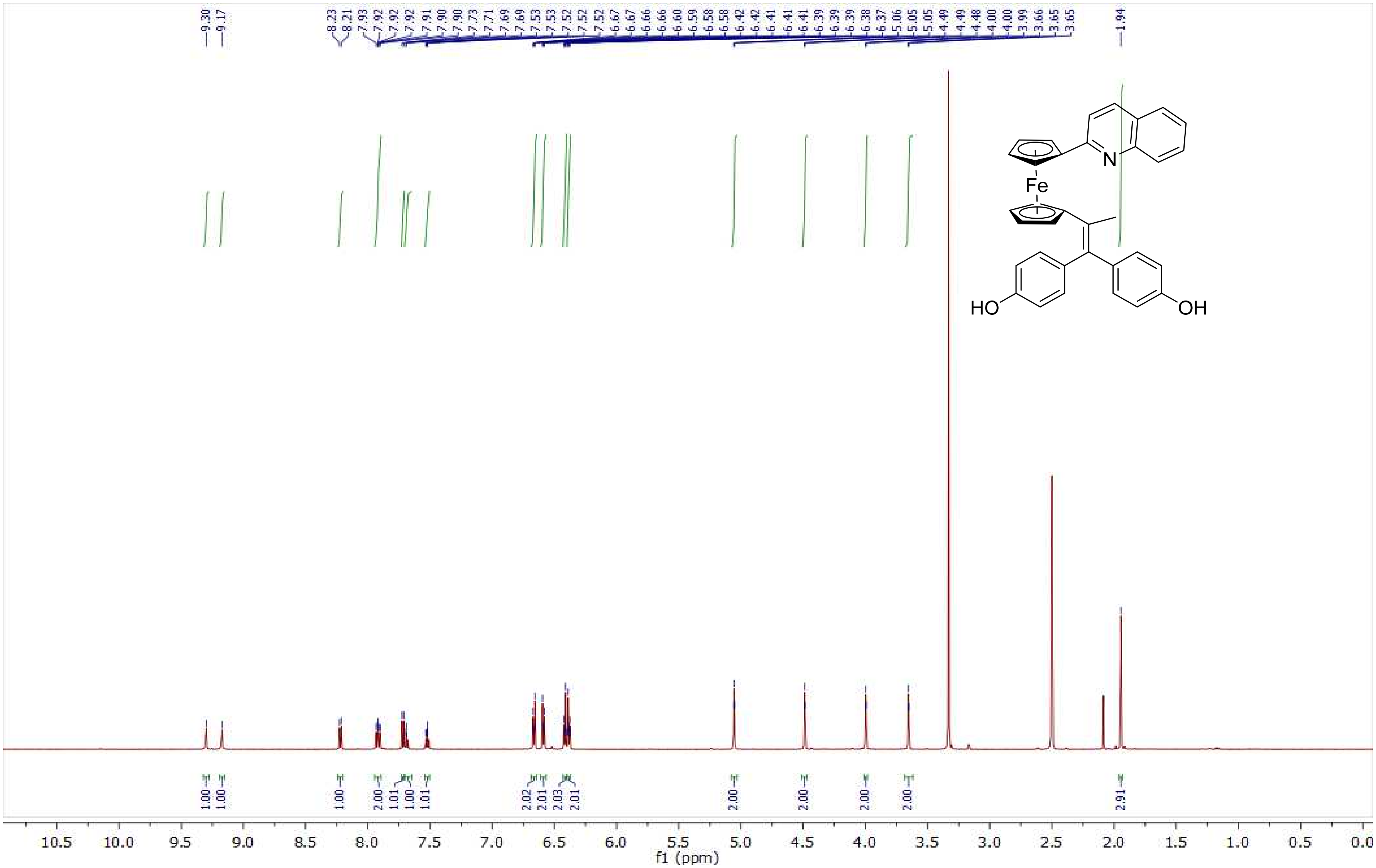

Figure S1.15. ^{1}H NMR (600 MHz) spectrum for 1-(quinolin-2-yl)-1´-{1-[bis(4-hydroxyphenyl)methylene]ethyl}ferrocene **(11)**.

Section S2. NMR ^{13}C Spectra

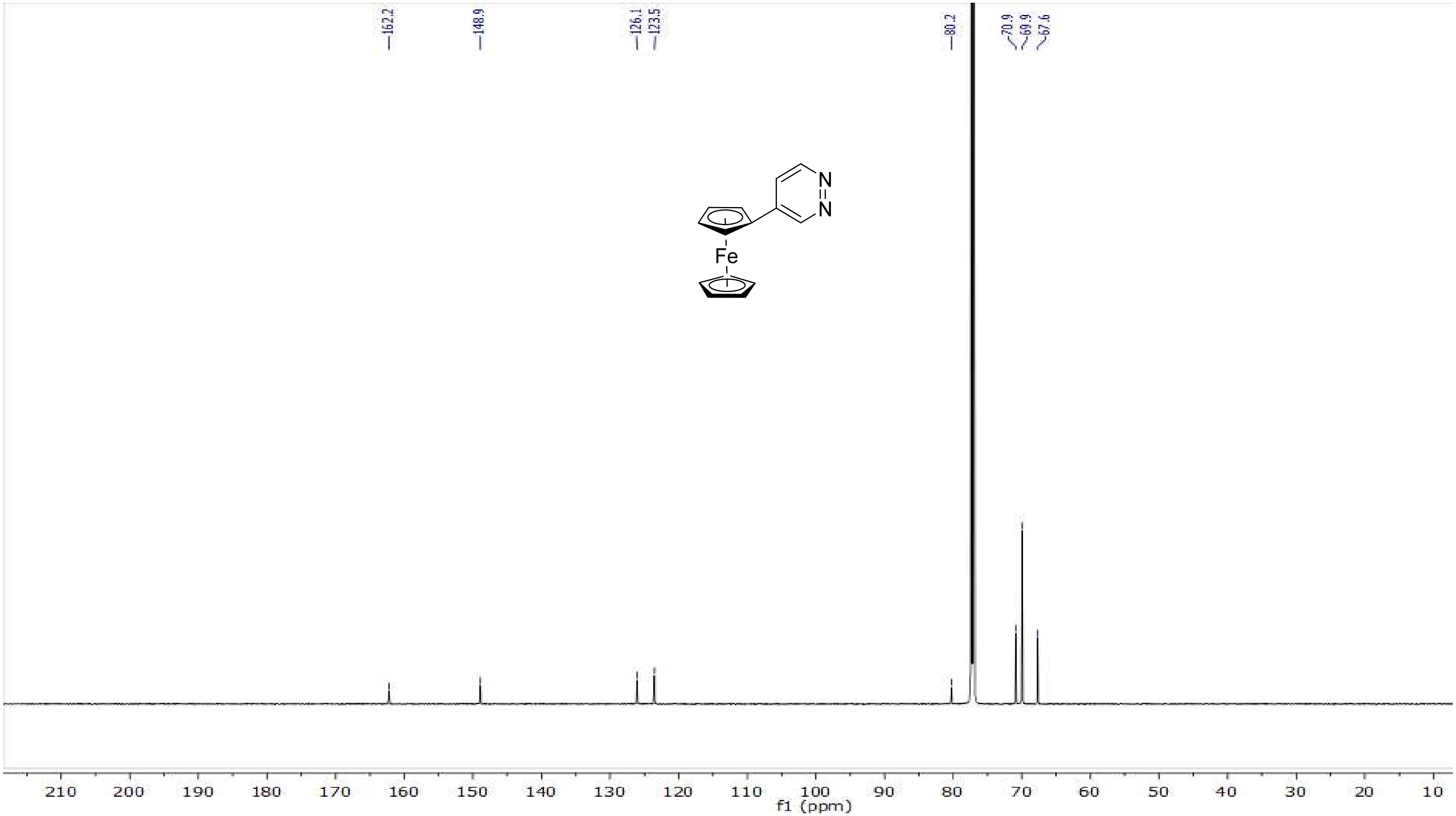

Figure S2.1. ^{13}C NMR (151 MHz) spectrum for 1-(pyridazin-4-yl)ferrocene **(1e)**.

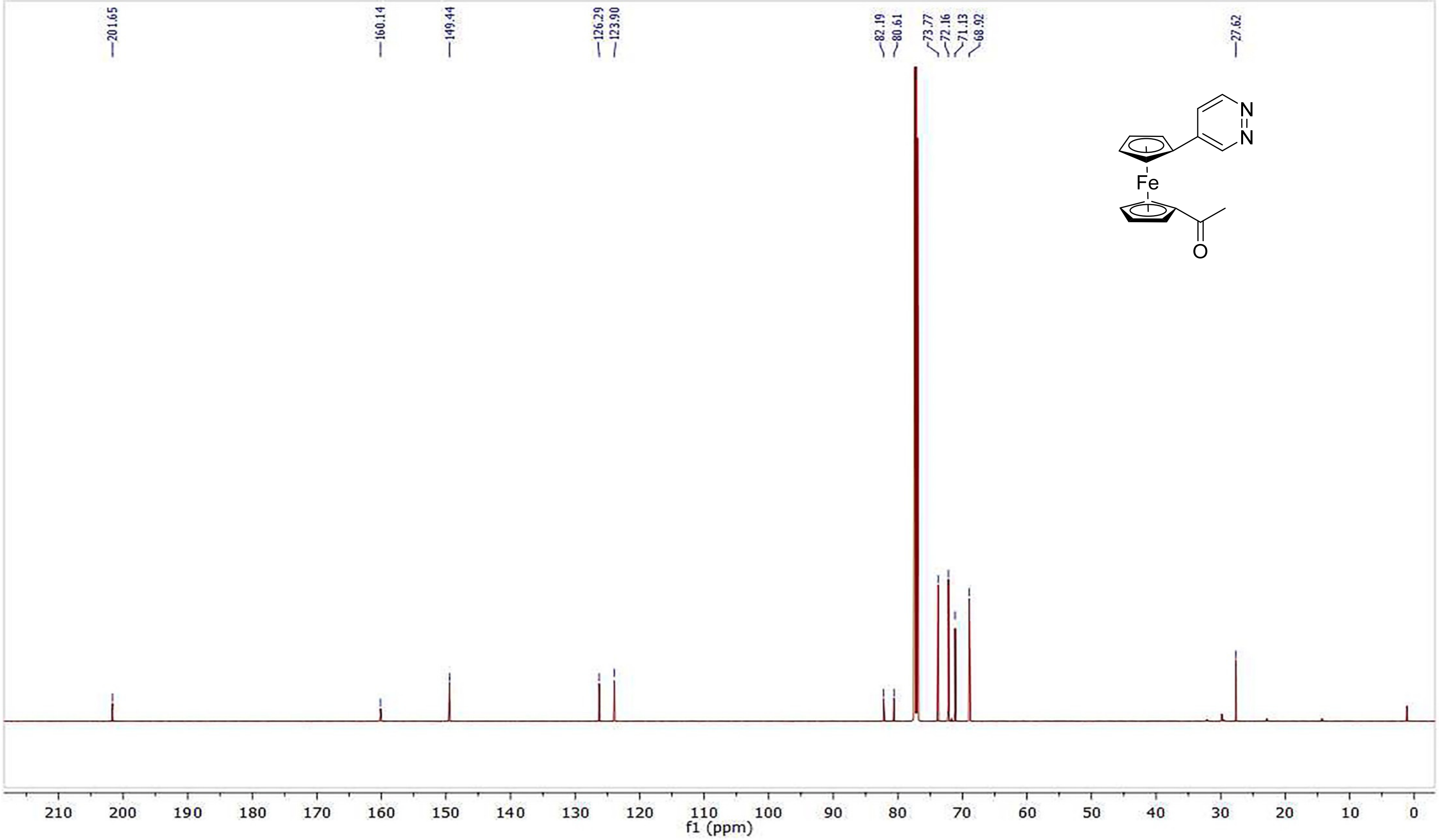

Figure S2.2. ^{13}C NMR (151 MHz) spectrum for 1-acetyl-1'-(pyridazin-4-yl)ferrocene **(2e)**.

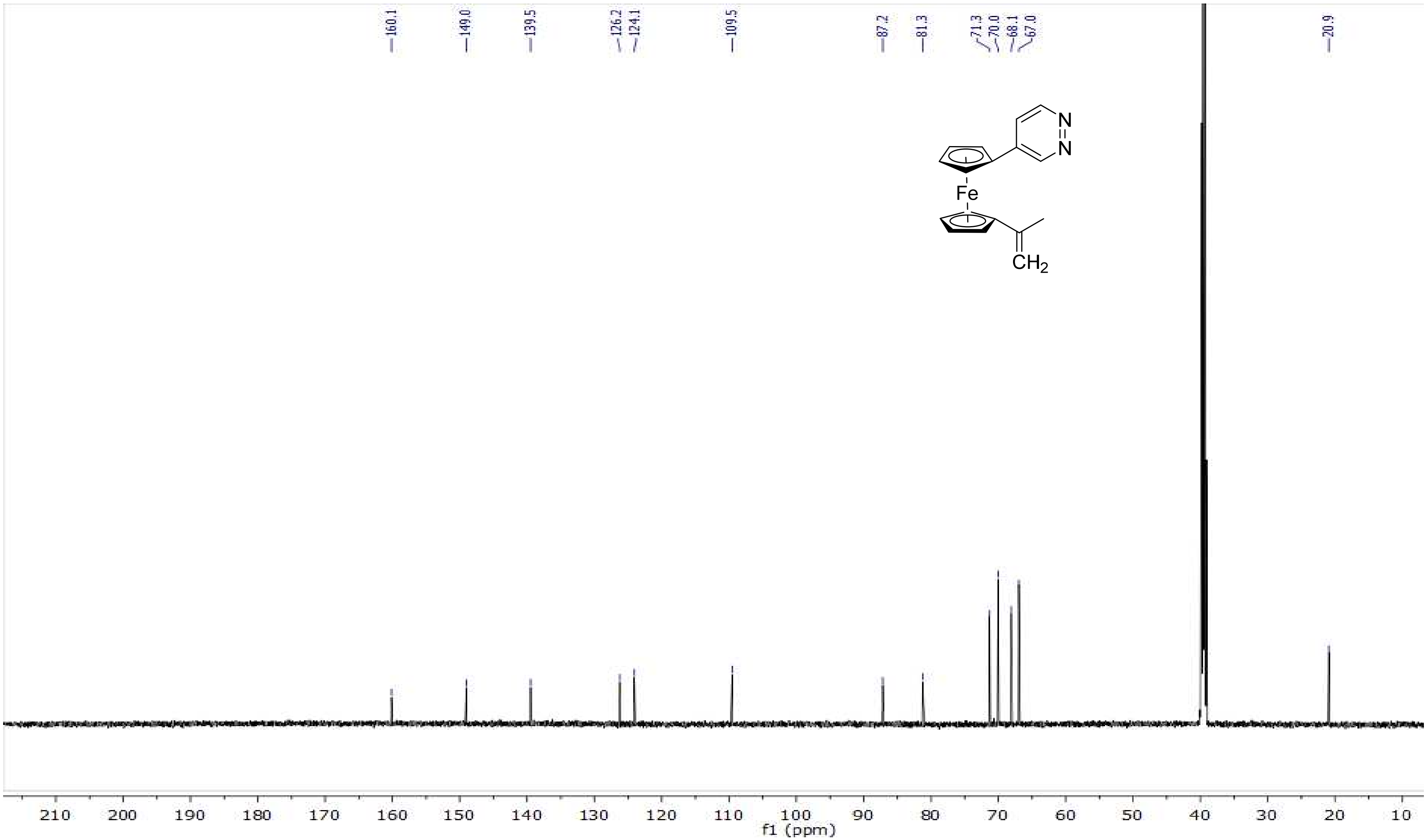

Figure S2.3. ^{13}C NMR (151 MHz) spectrum for 1-isopropenyl-1'-(pyridazin-4-yl)ferrocene **(5e)**.

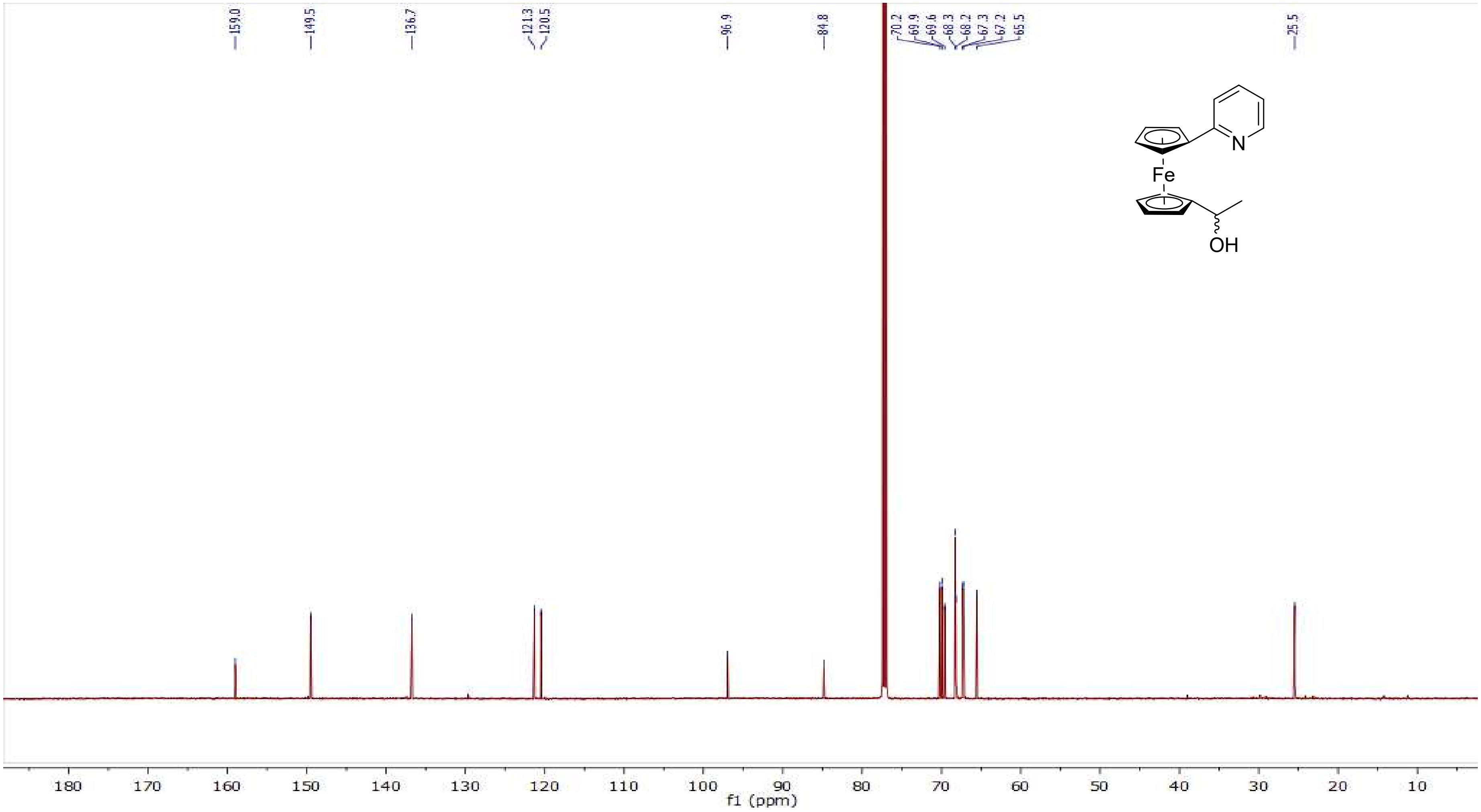

Figure S2.4. ^{13}C NMR (151 MHz) spectrum for 1-(pyridin-2-yl)-1'-(α-hydroxyethyl)ferrocene **(6a)**.

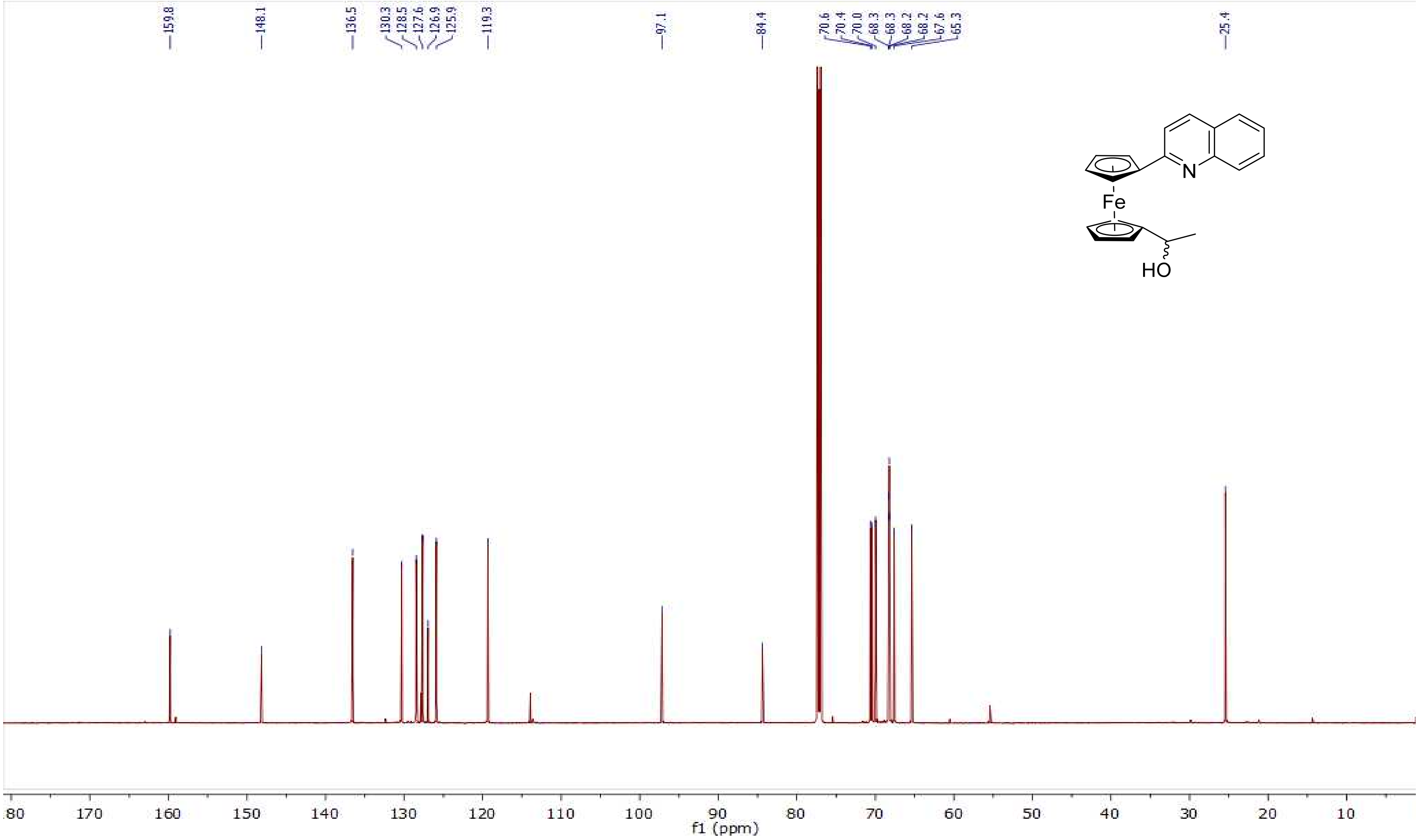

Figure S2.5. ^{13}C NMR (151 MHz) spectrum for 1-(quinolin-2-yl)-1'-(α-hydroxyethyl)ferrocene **(6b)**.

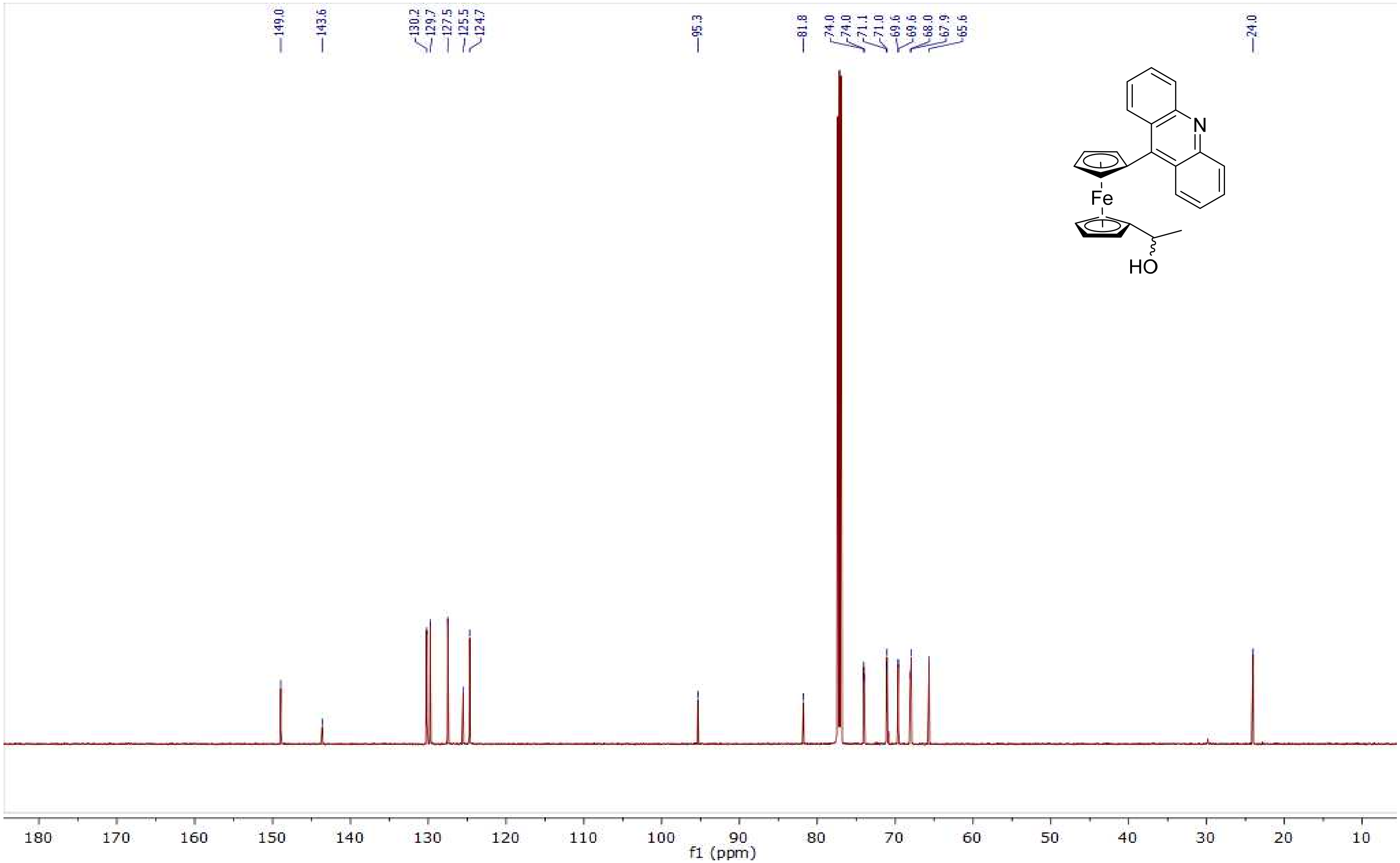

Figure S2.6. ^{13}C NMR (151 MHz) spectrum for 1-(acridin-9-yl)-1'-(α-hydroxyethyl)ferrocene **(6c)**.

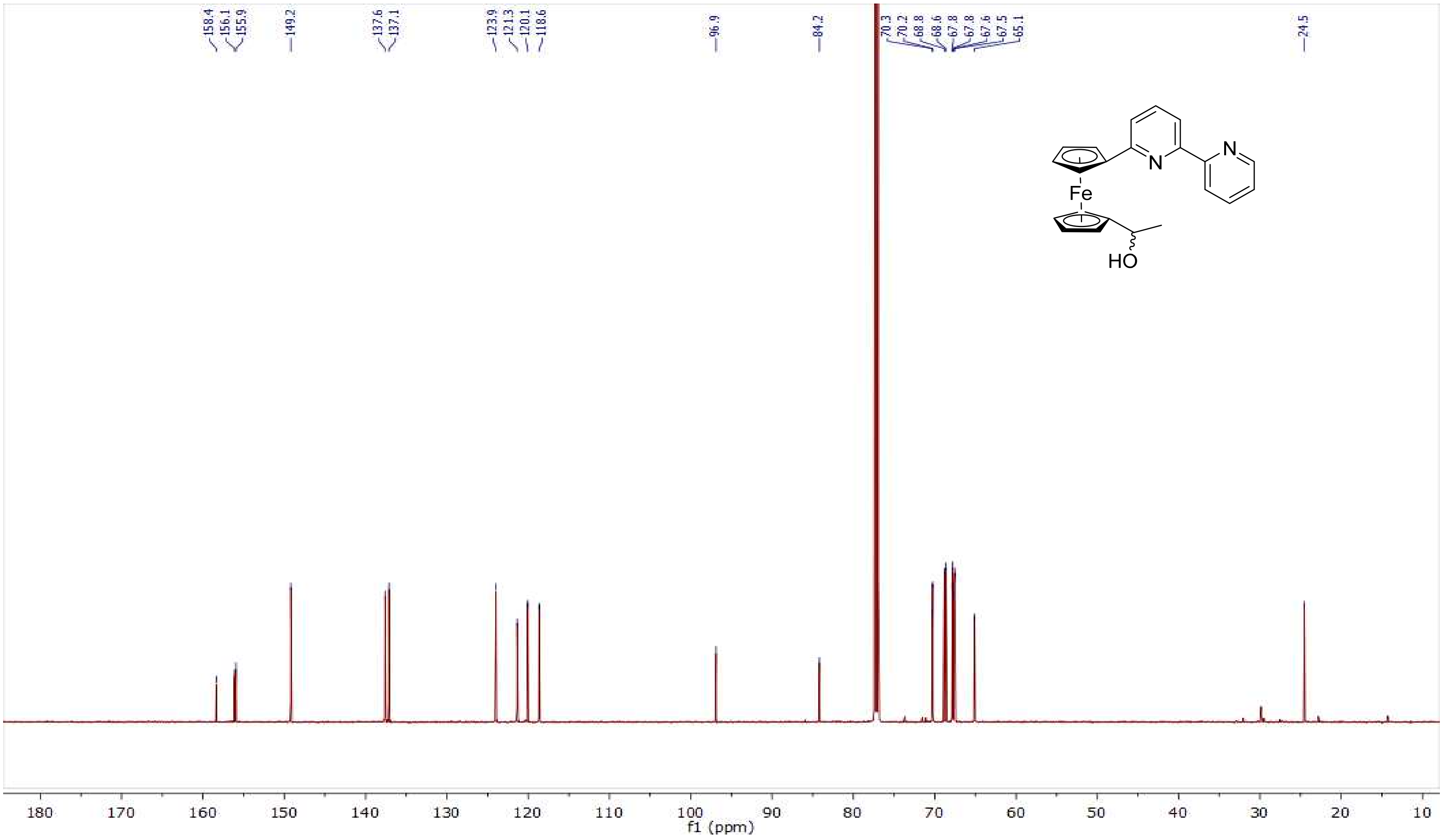

Figure S2.7. ^{13}C NMR (151 MHz) spectrum for 1-(2,2'-bipyridin-6-yl)-1'-(α-hydroxyethyl)ferrocene **(6d)**.

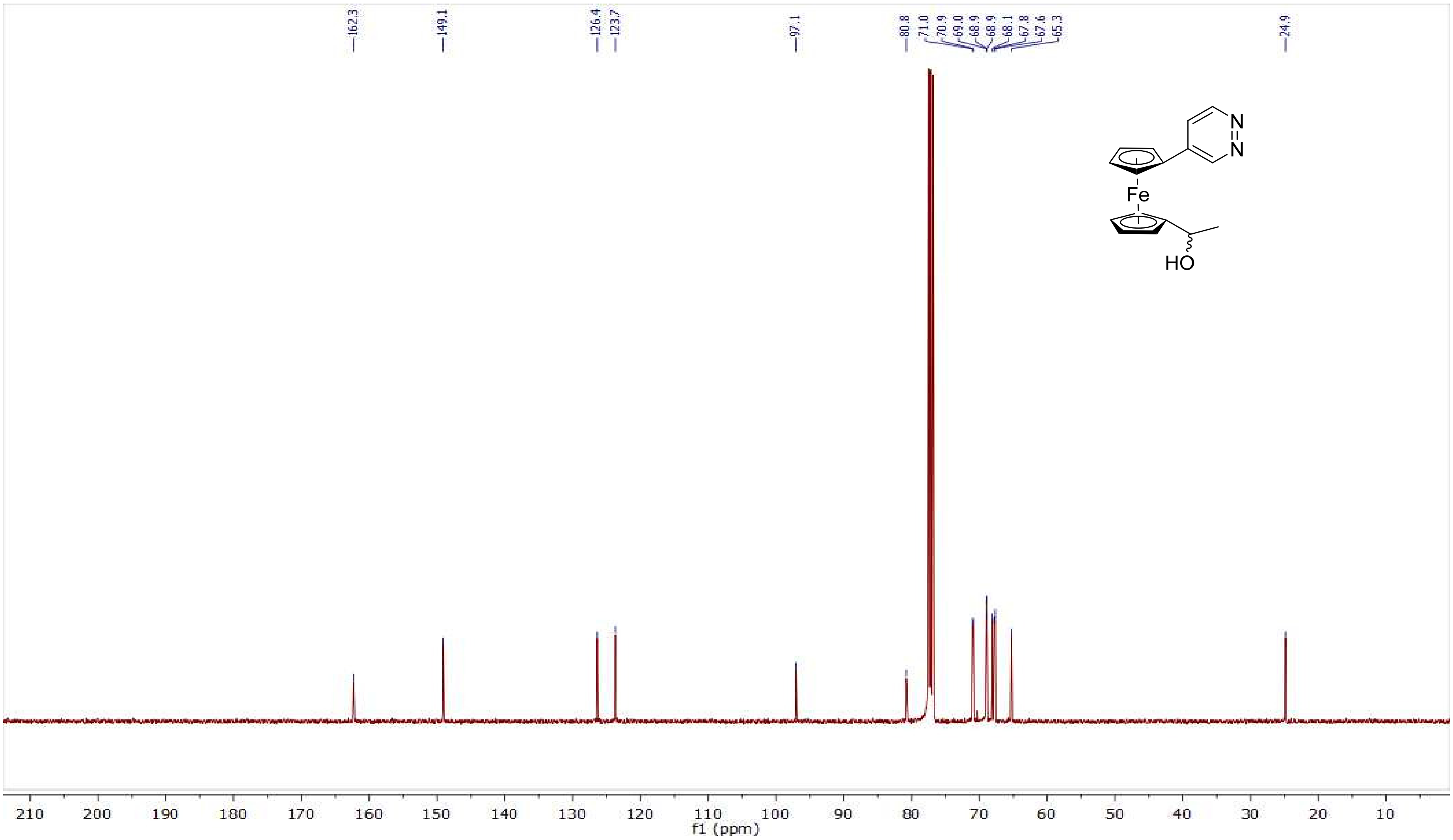

Figure S2.8. ^{13}C NMR (100 MHz) spectrum for 1-(pyridazin-4-yl)-1'-(α-hydroxyethyl)ferrocene **(6e)**.

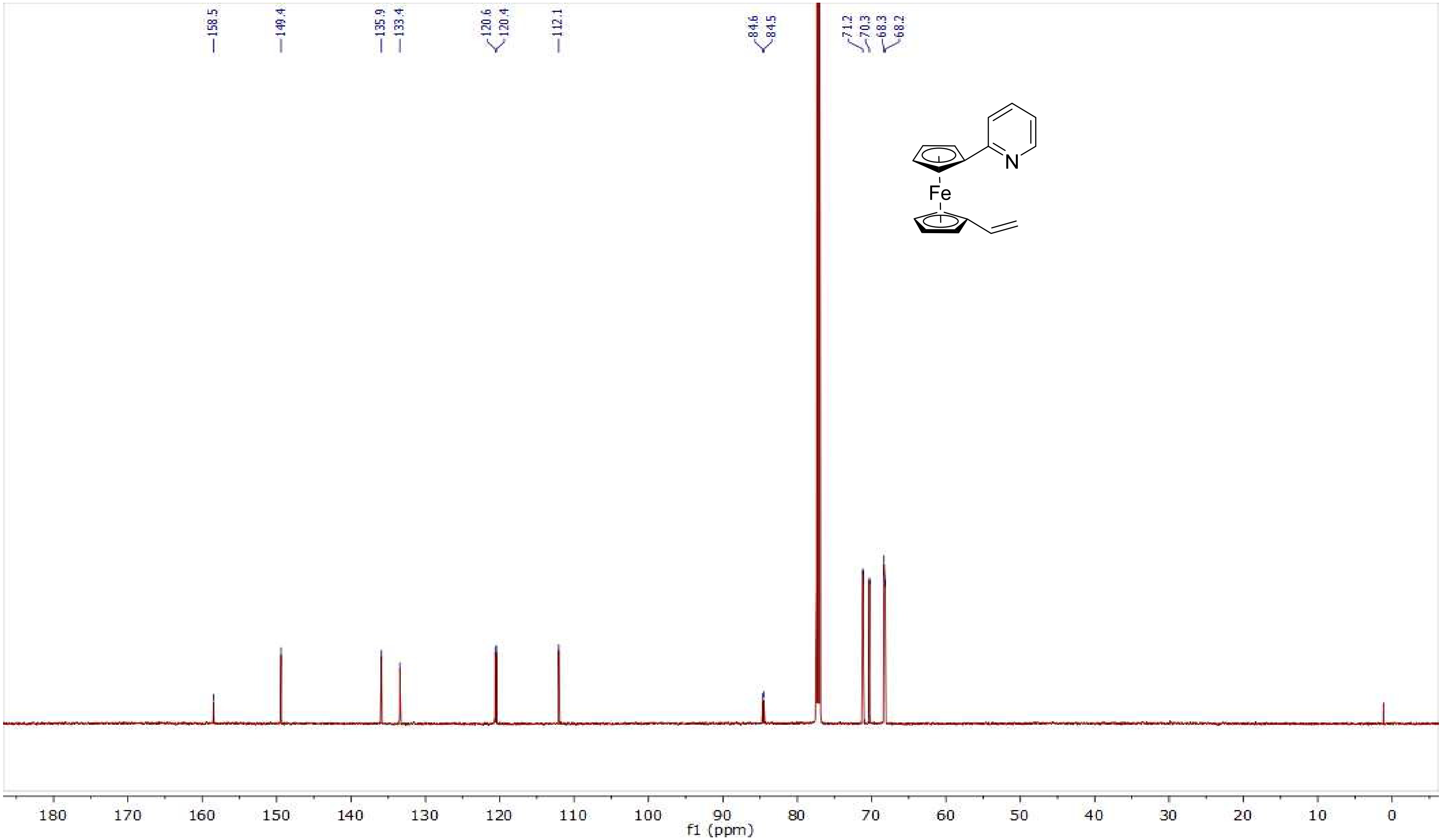

Figure S2.9. ^{13}C NMR (151 MHz) spectrum for 1-(pyridin-2-yl)-1'-vinylferrocene **(7a)**.

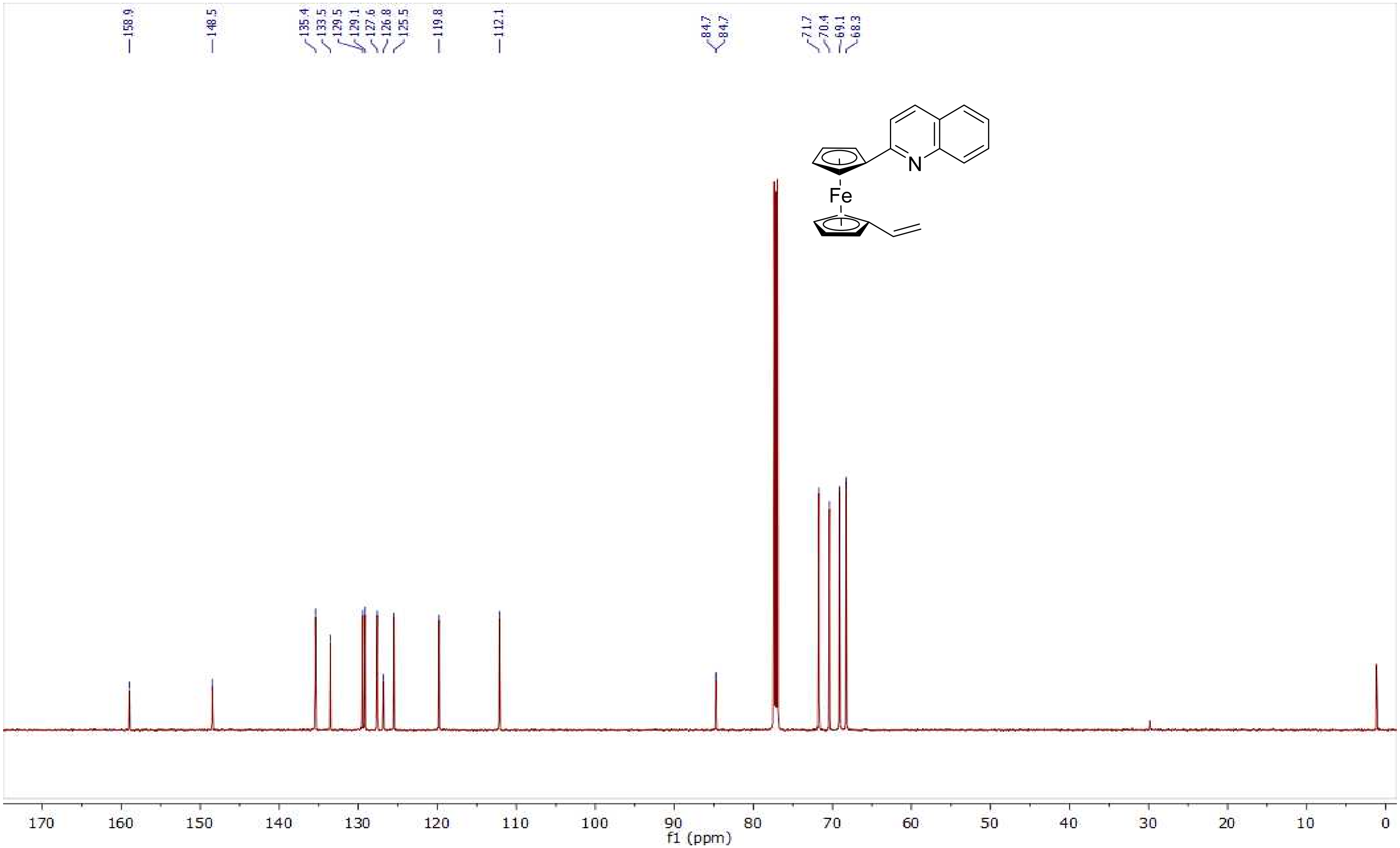

Figure S2.10. ^{13}C NMR (151 MHz) spectrum for 1-(quinolin-2-yl)-1'-vinylferrocene **(7b)**.

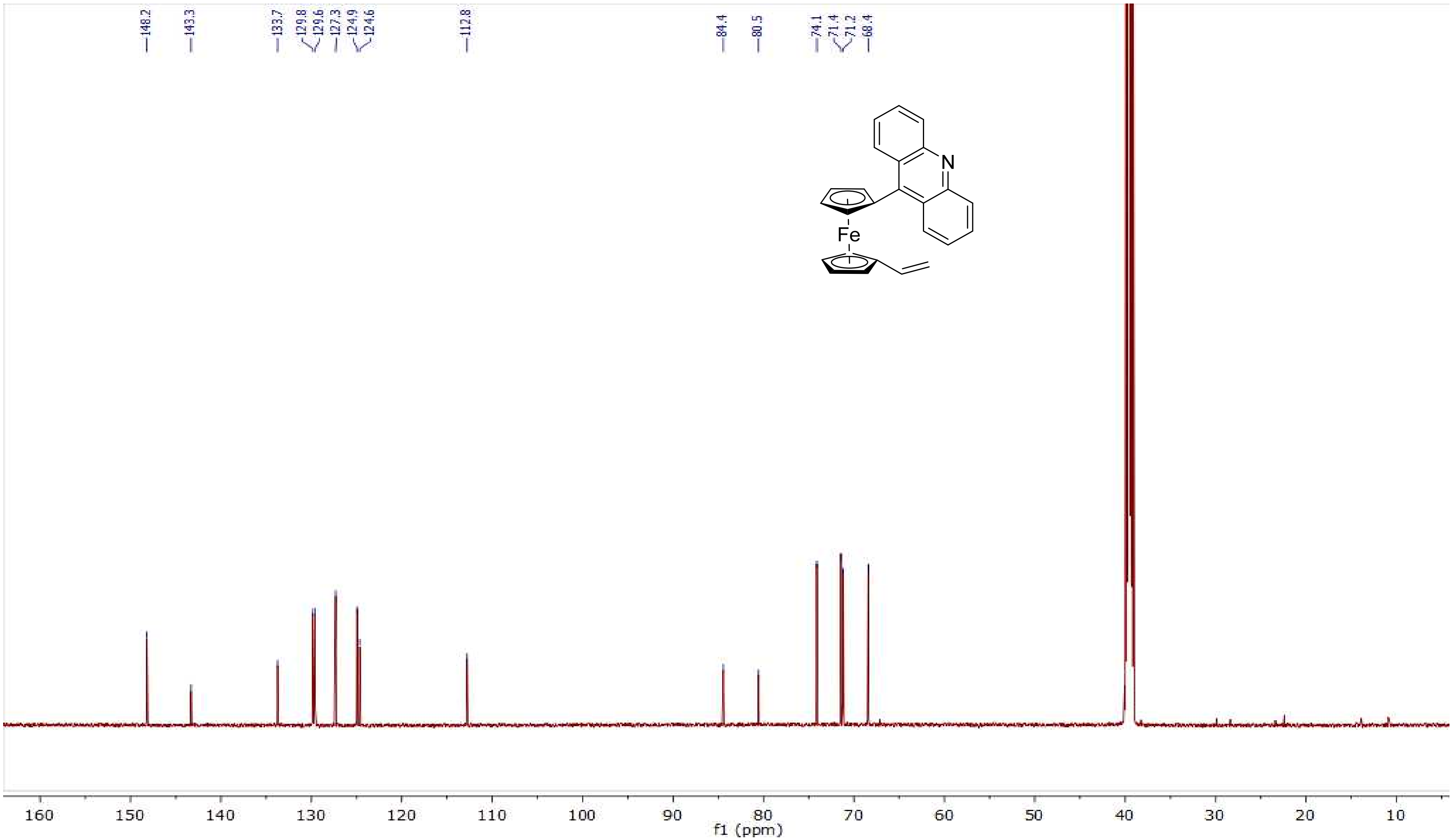

Figure S2.11. ^{13}C NMR (151 MHz) spectrum for 1-(acridin-9-yl)-1'-vinylferrocene **(7c)**.

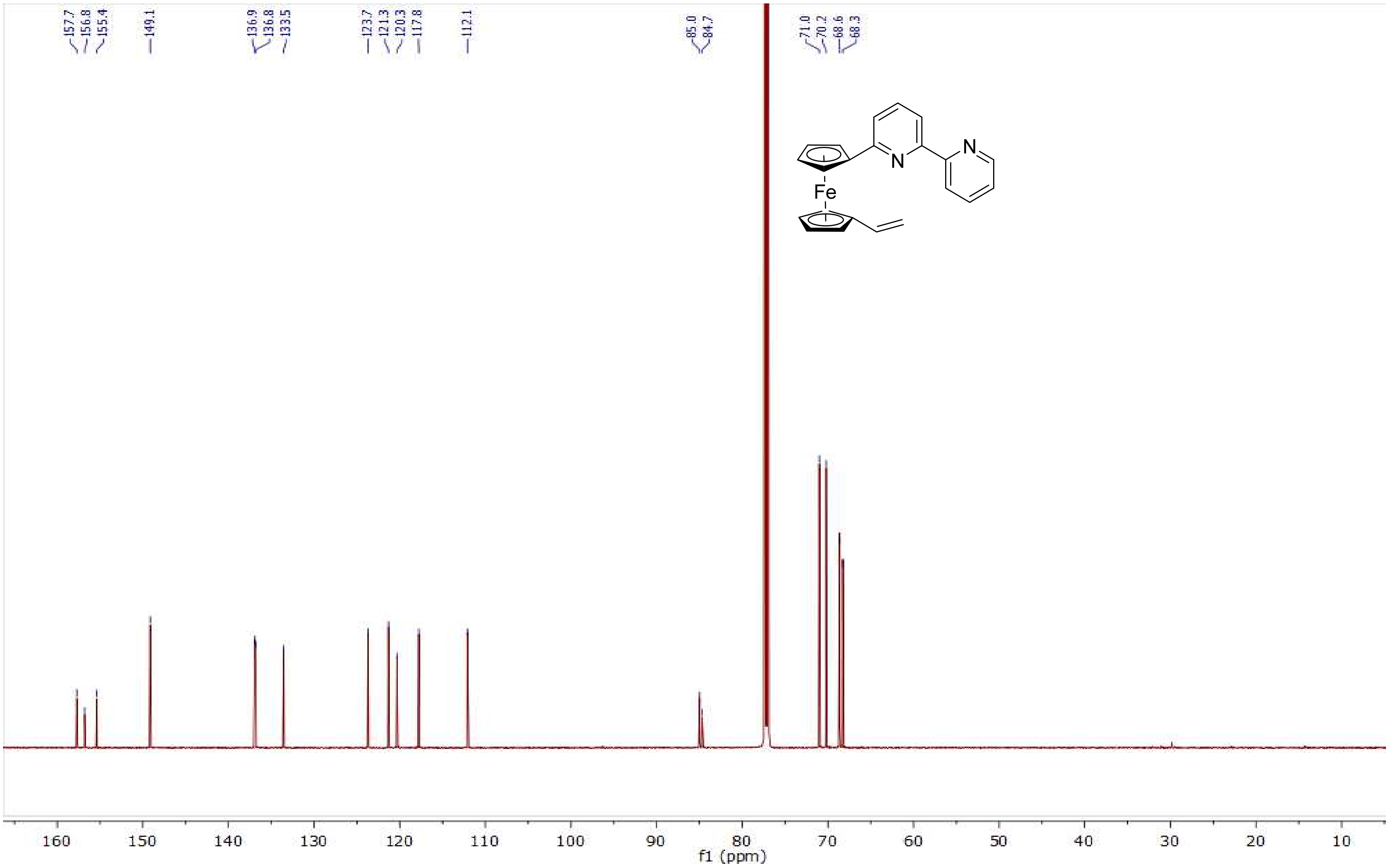

Figure S2.12. ^{13}C NMR (151 MHz) spectrum for 1-(2,2'-bipyridin-6-yl)-1'-vinylferrocene **(7d)**.

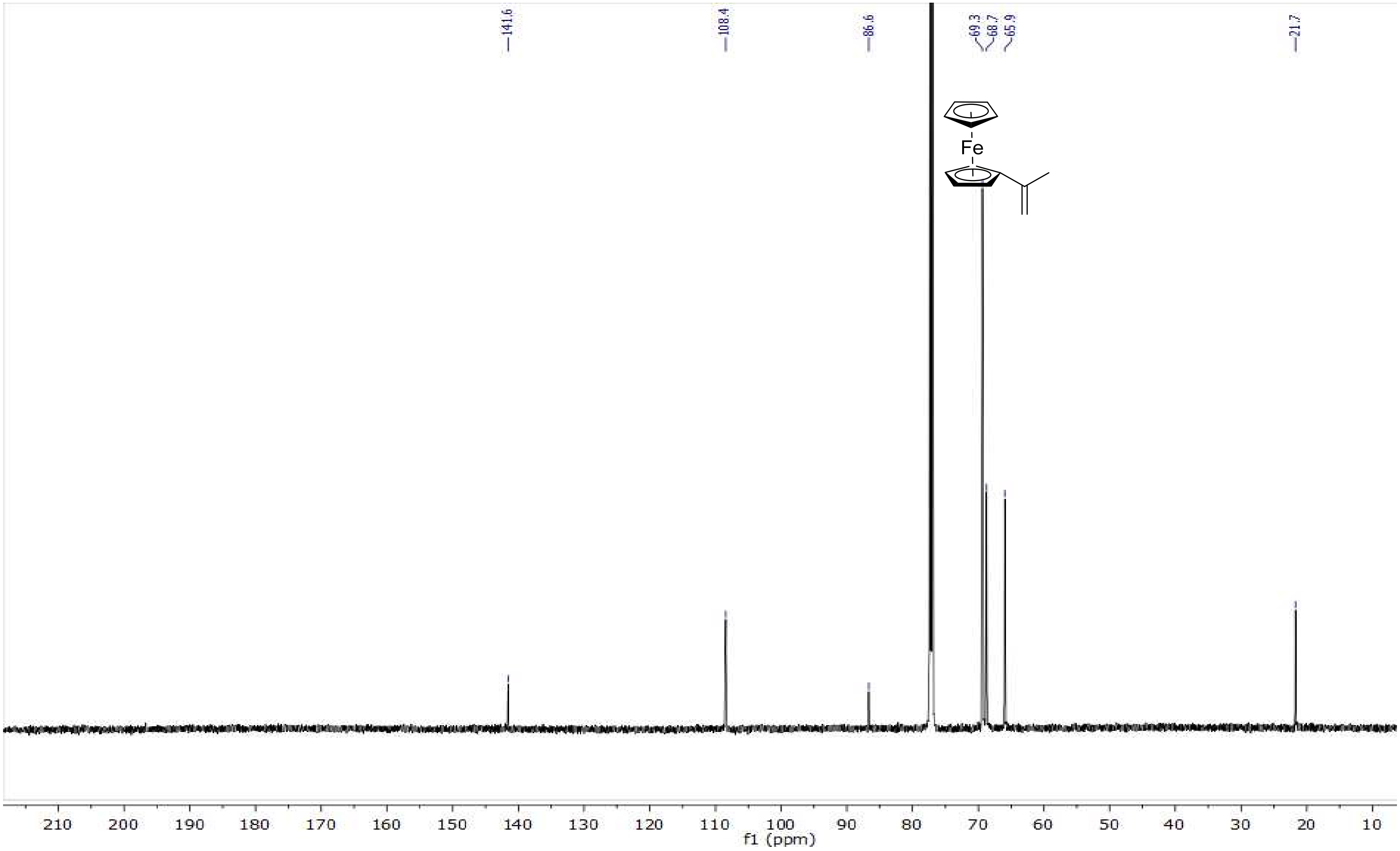

Figure S2.13. ^{13}C NMR (151 MHz) spectrum for isopropenylferrocene **(8)**.

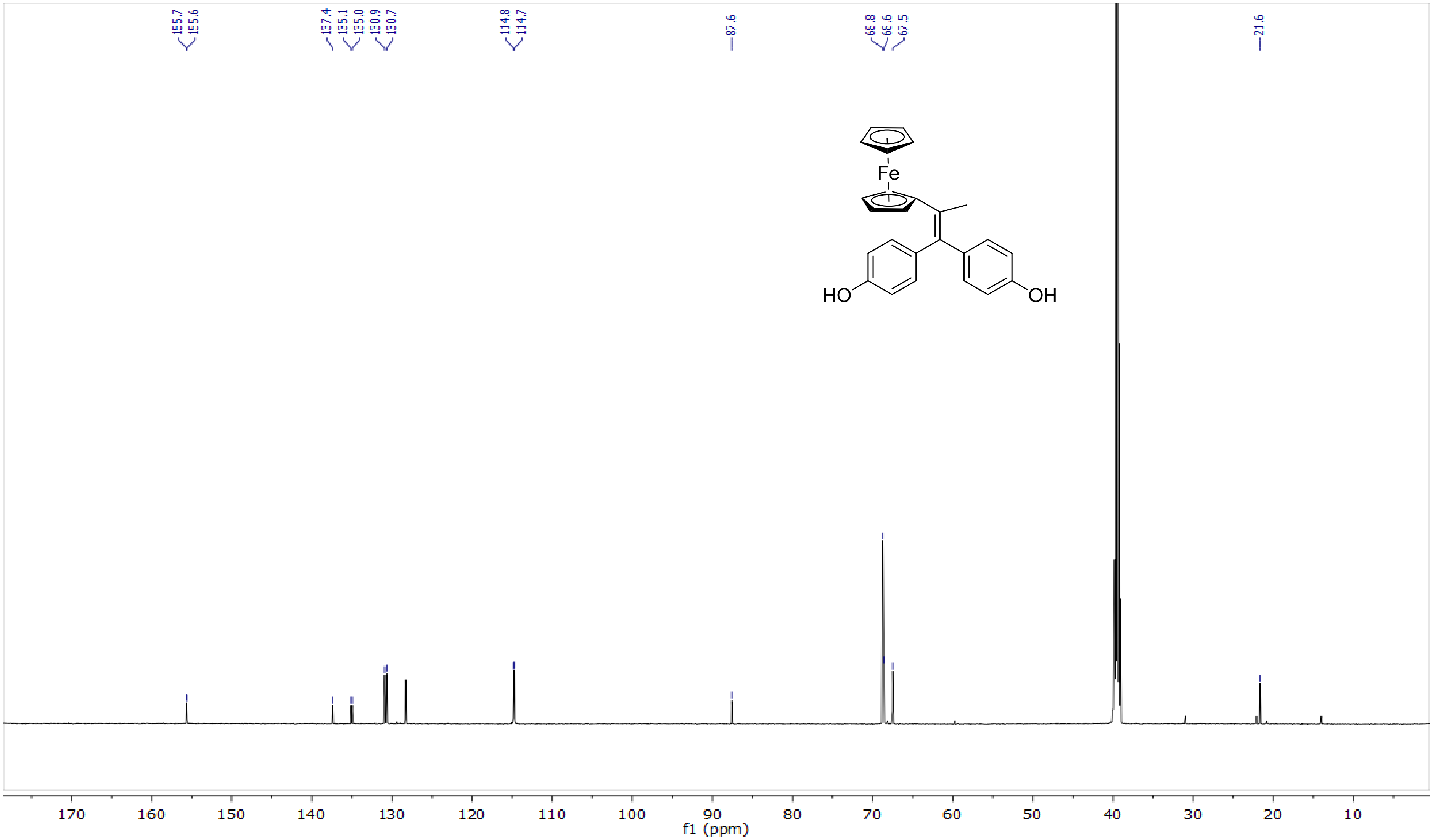

Figure S2.14. ^{13}C NMR (151 MHz) spectrum for 1,1-bis(4-hydroxyphenyl)-2-ferrocenylprop-1-ene **(10)**.

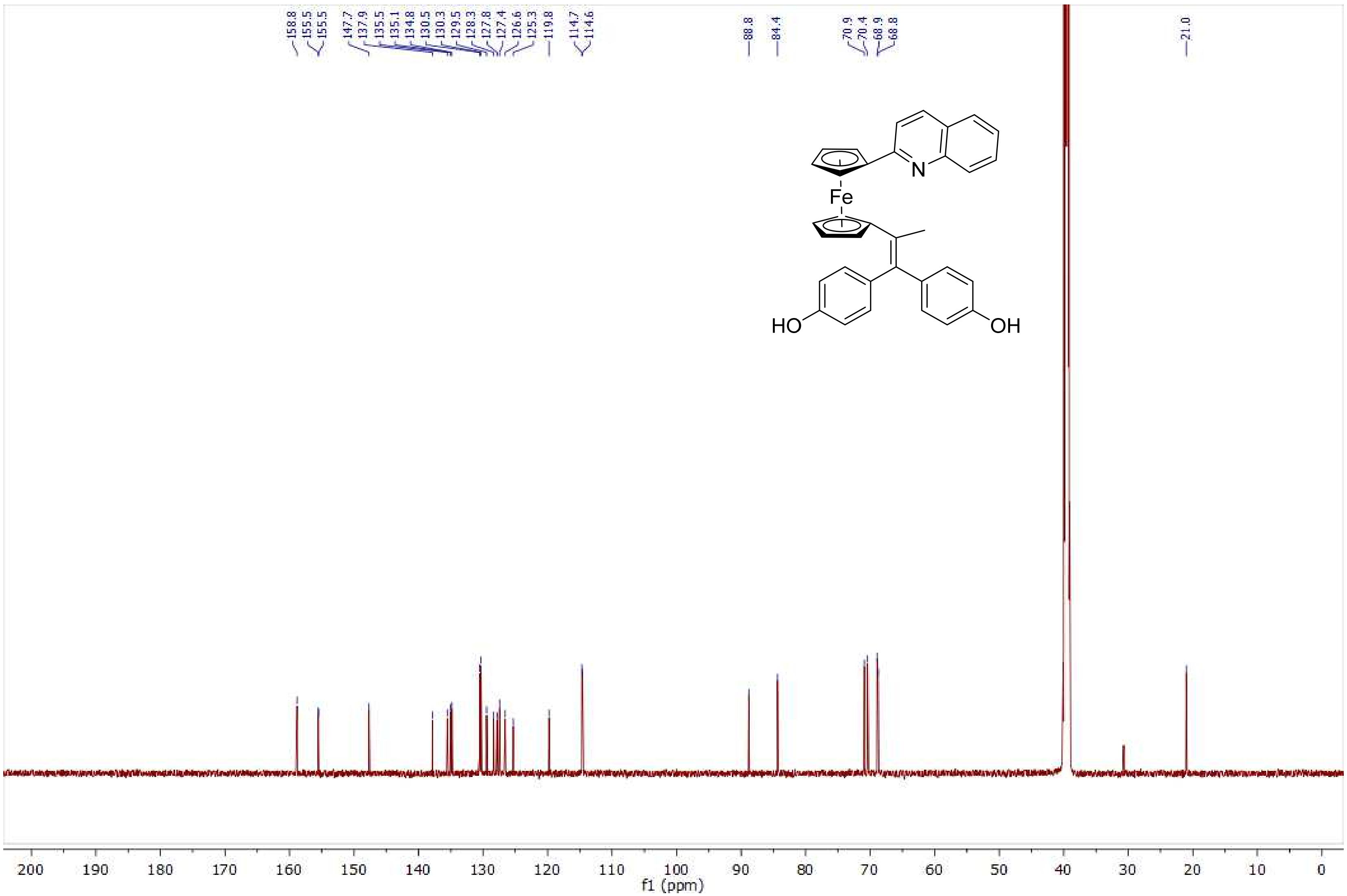

Figure S2.15. ^{13}C NMR (151 MHz) spectrum for 1-(quinolin-2-yl)-1´-{1-[bis(4-hydroxyphenyl)methylene]ethyl}ferrocene **(11)**.

Section S3. HSQC and HMBC Spectra

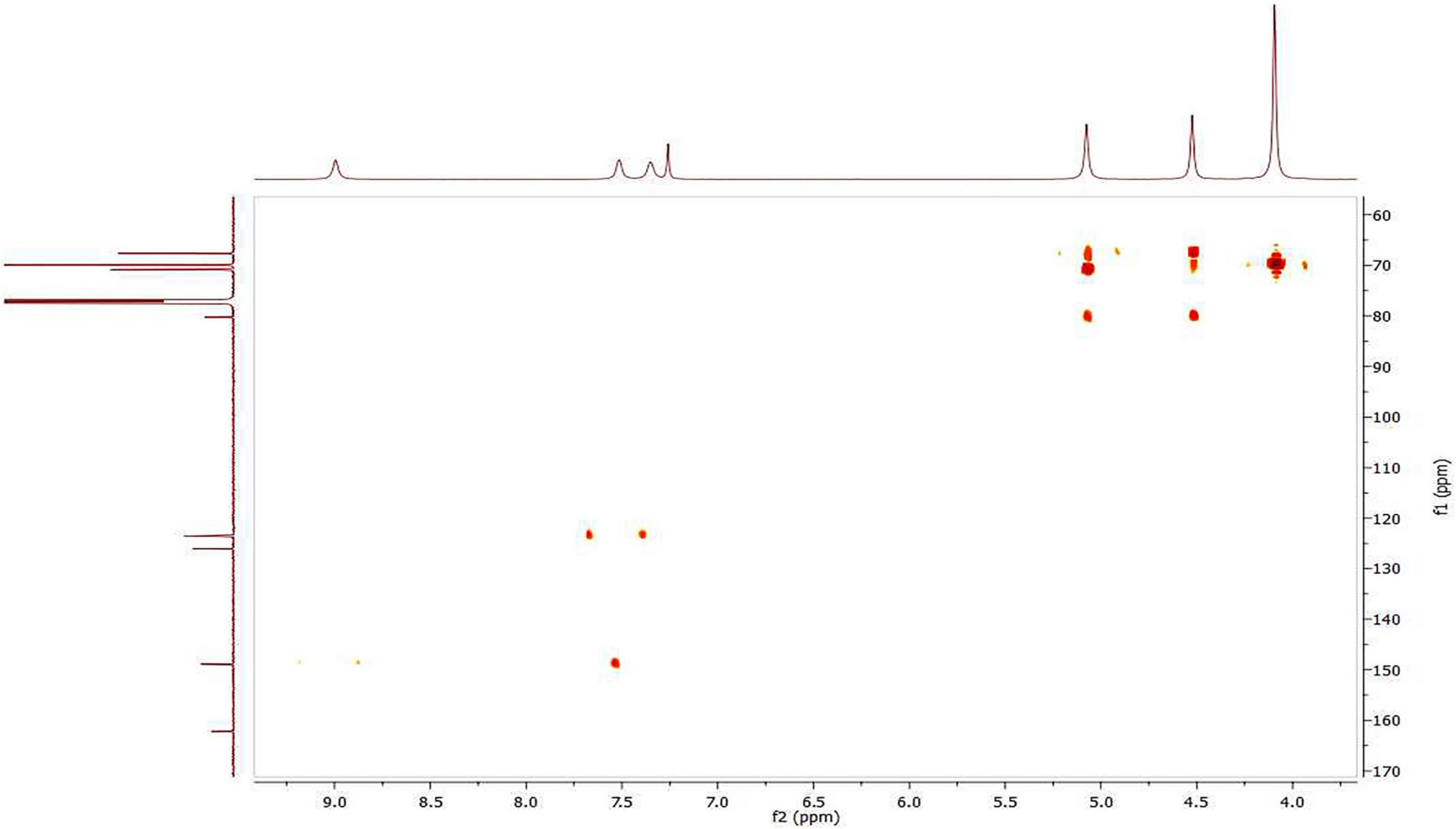

Figure S3.1. ^{1}H-^{13}C HSQC spectrum for 1-(pyridazin-4-yl)ferrocene **(1e)**.

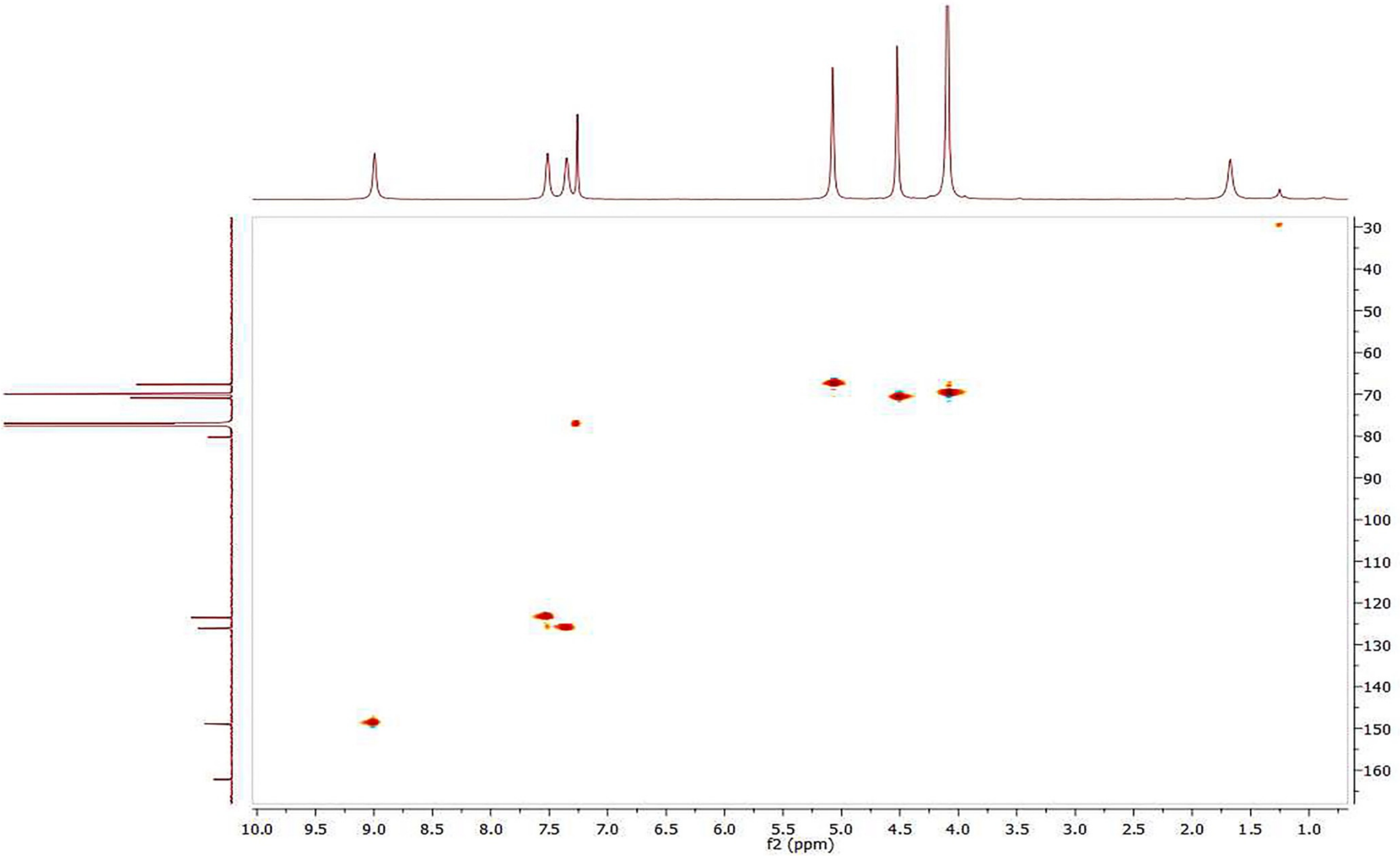

Figure S3.2. ^{1}H-^{13}C HMBC spectrum for 1-(pyridazin-4-yl)ferrocene **(1e)**.

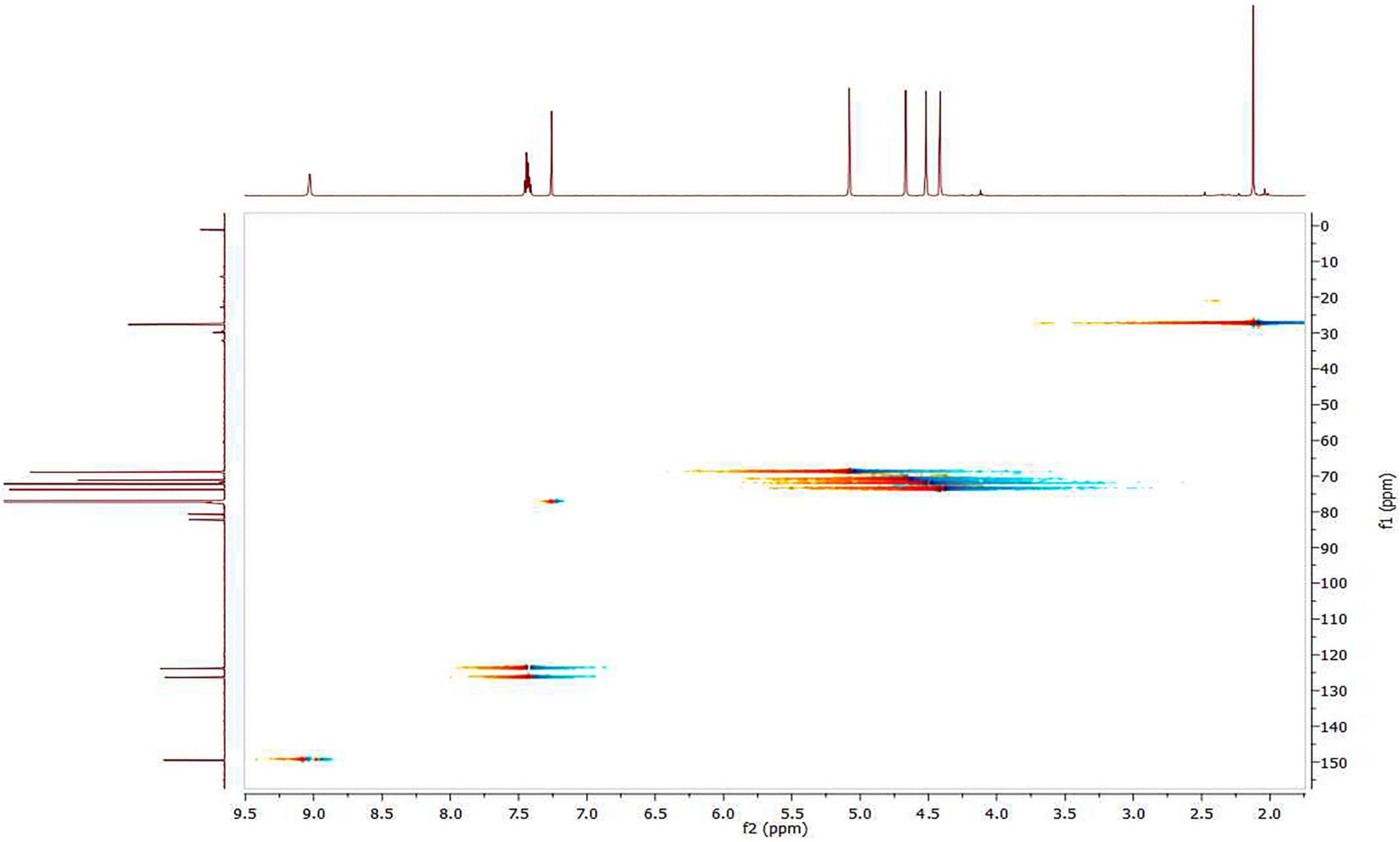

Figure S3.3. ^{1}H-^{13}C HSQC spectrum for 1-acetyl-1'-(pyridazin-4-yl)ferrocene **(2e)**.

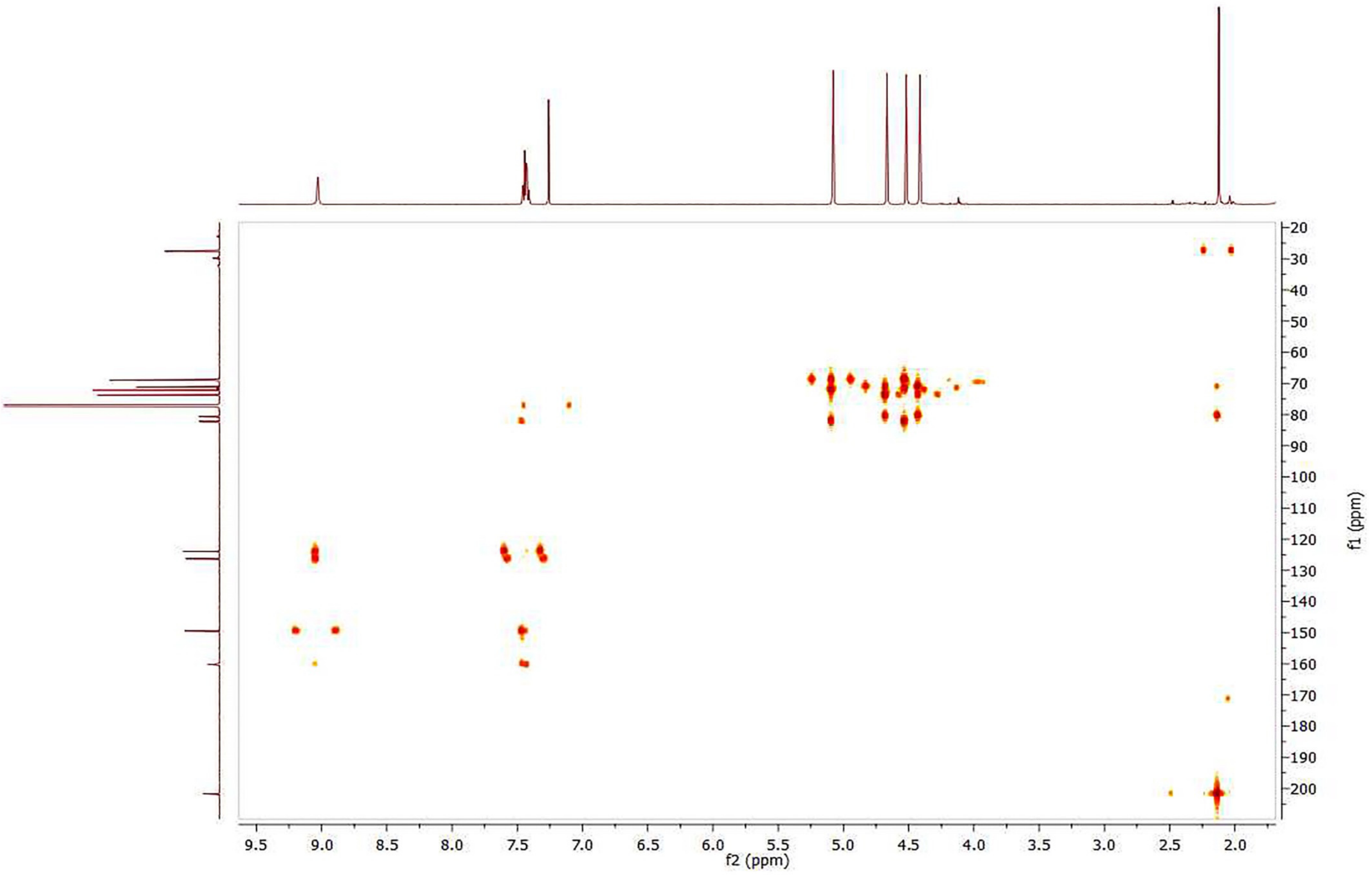

Figure S3.4. ^{1}H-^{13}C HMBC spectrum for 1-acetyl-1'-(pyridazin-4-yl)ferrocene **(2e)**.

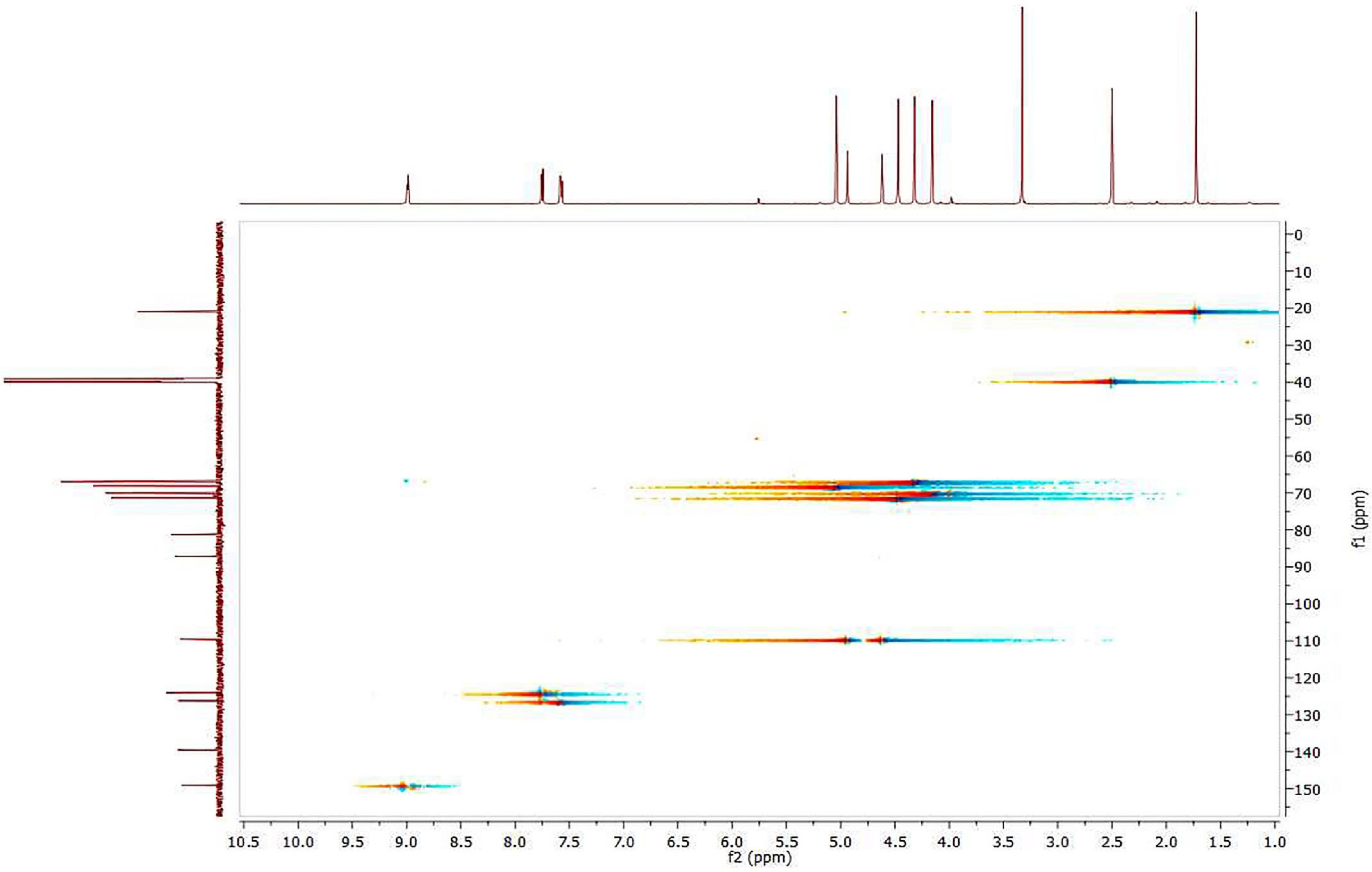

Figure S3.5. ^{1}H-^{13}C HSQC spectrum for 1-isopropenyl-1'-(pyridazin-4-yl)ferrocene **(5e)**.

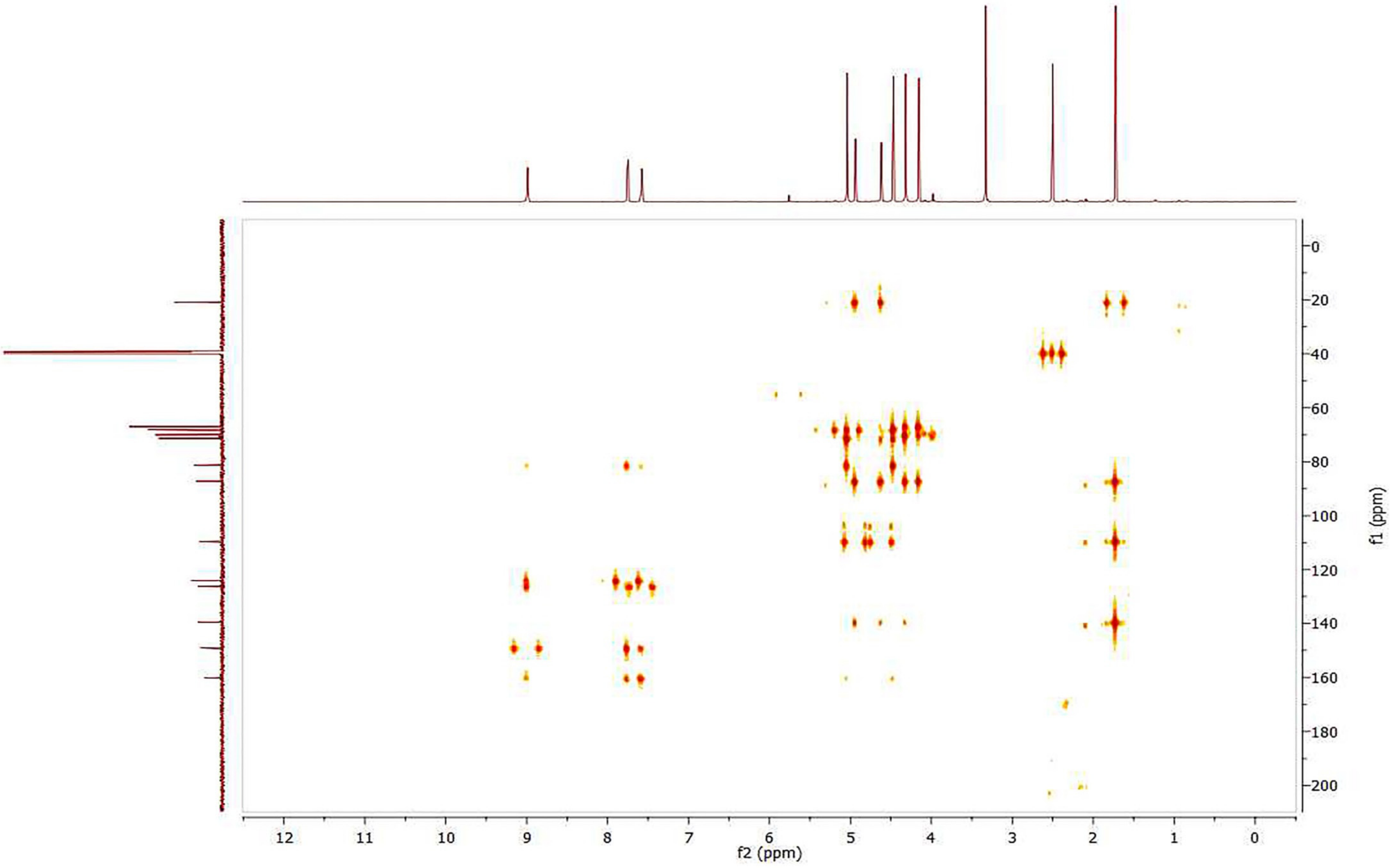

Figure S3.6. ^{1}H-^{13}C HMBC spectrum for 1-isopropenyl-1'-(pyridazin-4-yl)ferrocene **(5e)**.

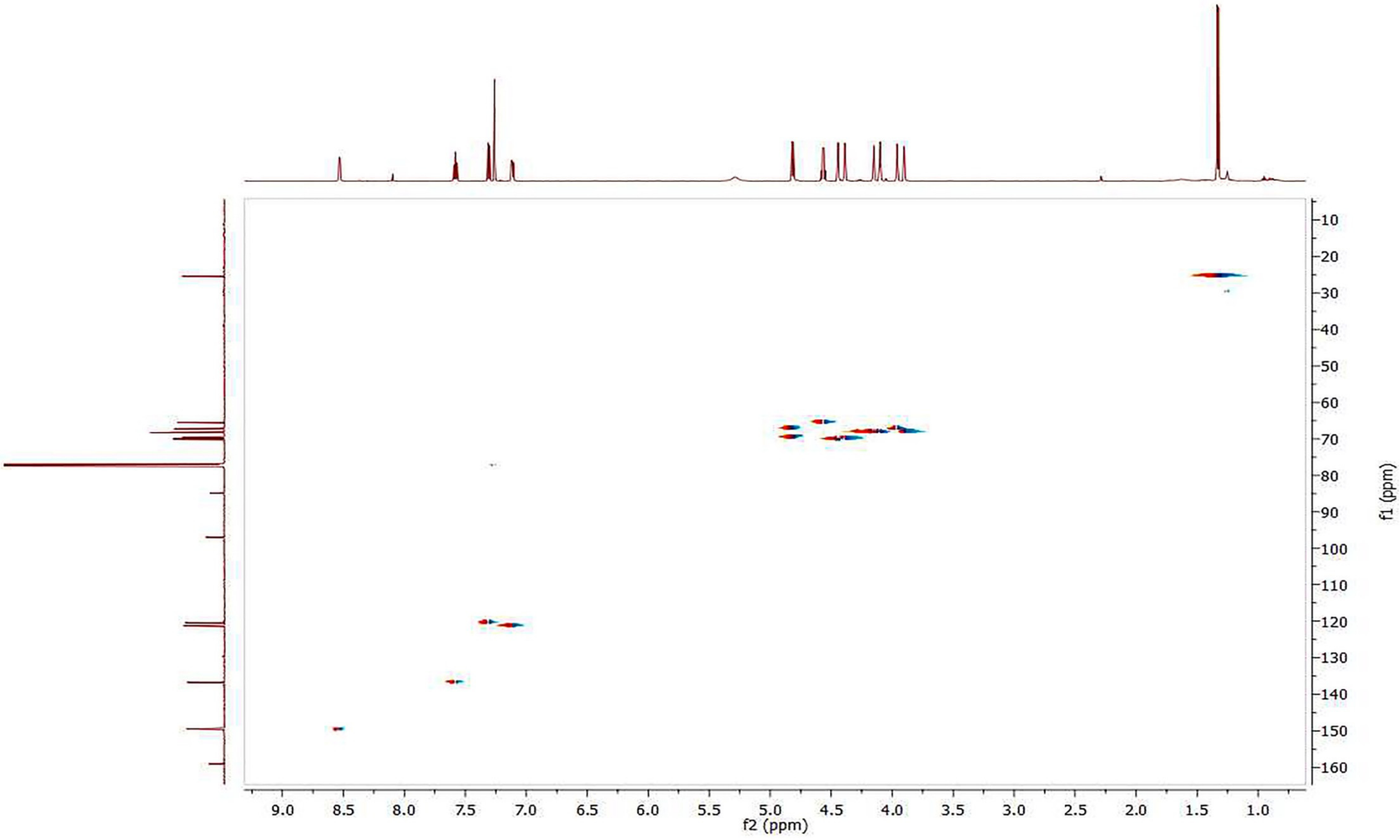

Figure S3.7. ^{1}H-^{13}C HSQC spectrum for 1-(pyridin-2-yl)-1'-(α-hydroxyethyl)ferrocene **(6a)**.

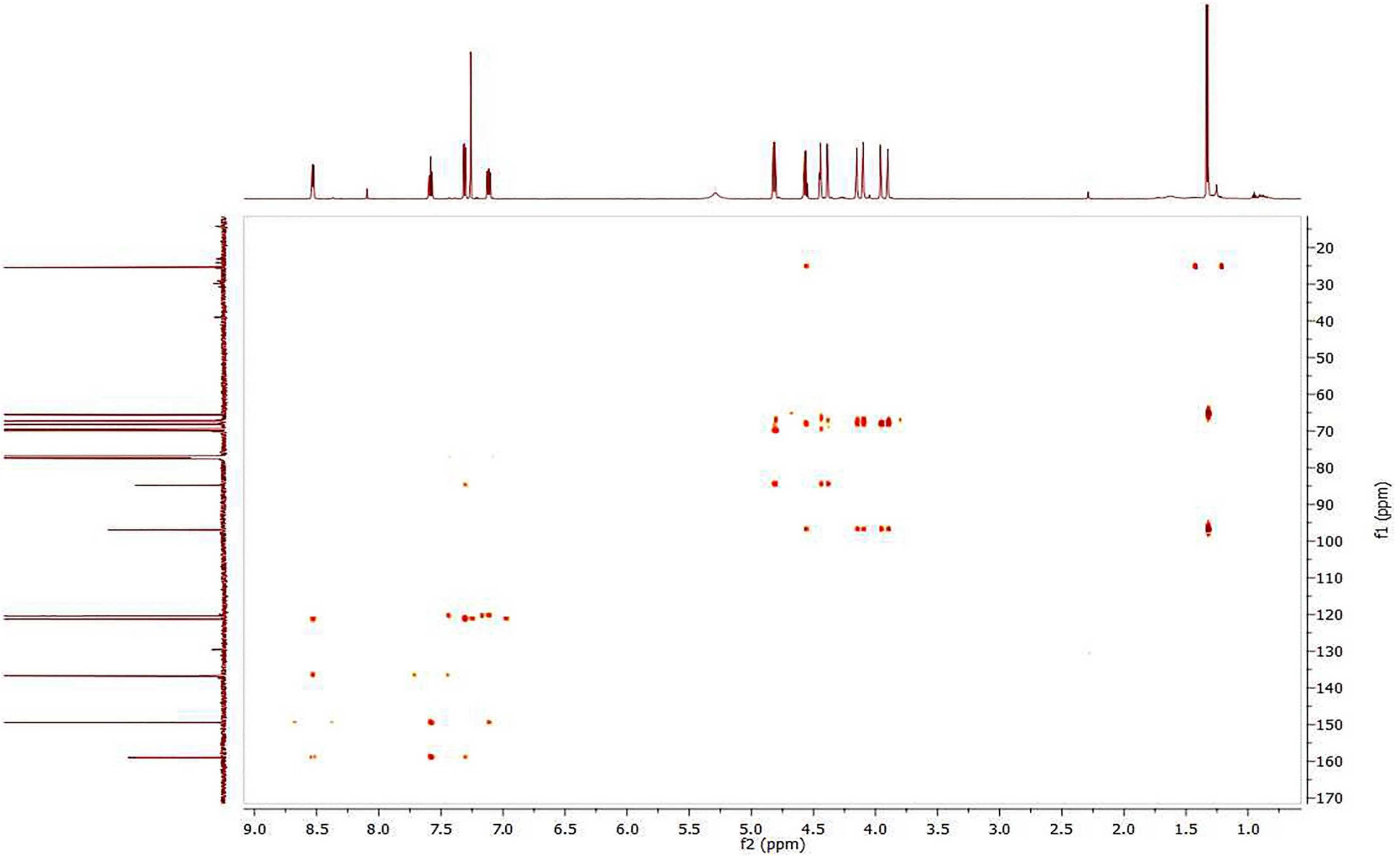

Figure S3.8. ^{1}H-^{13}C HMBC spectrum for 1-(pyridin-2-yl)-1'-(α-hydroxyethyl)ferrocene **(6a)**.

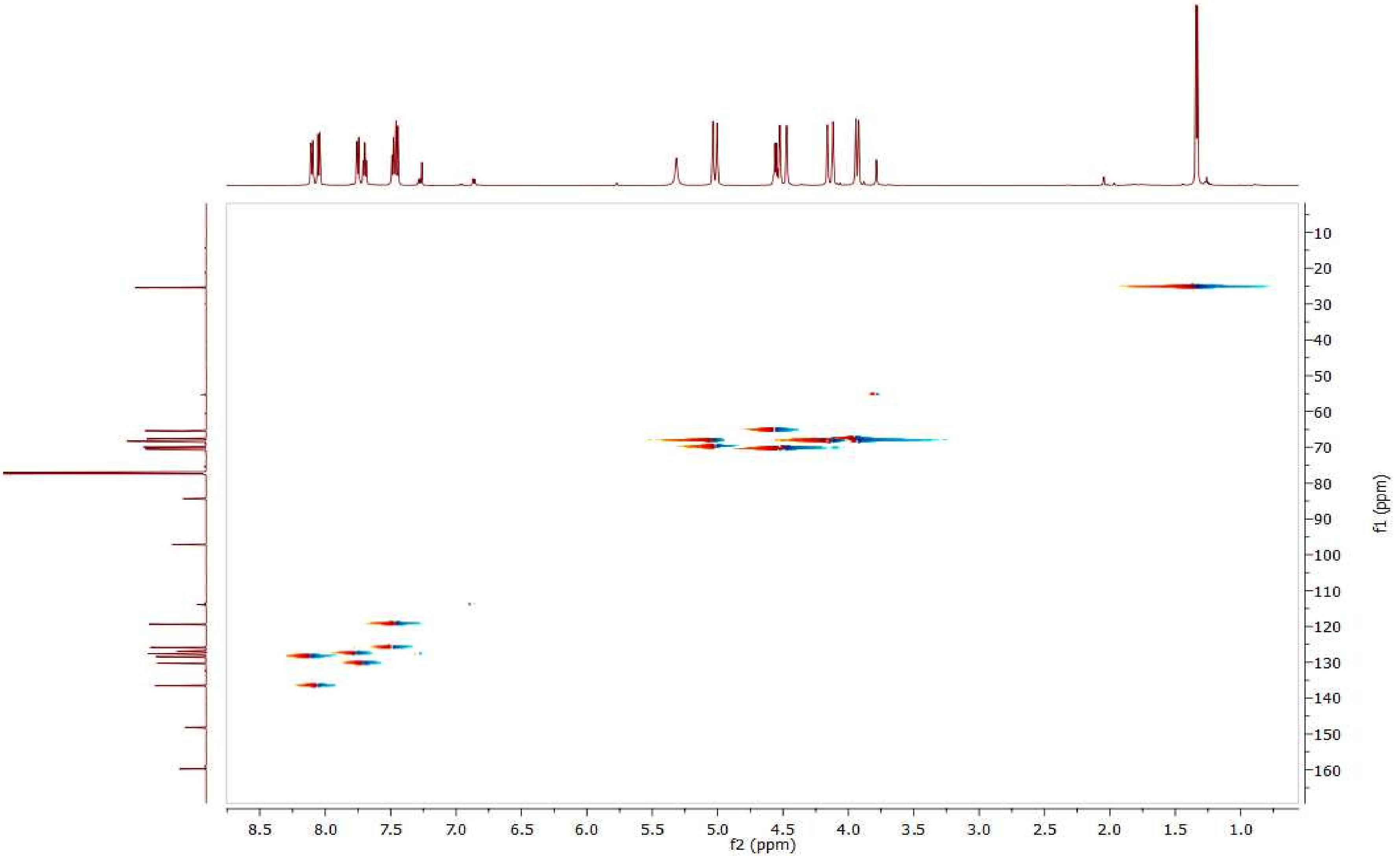

Figure S3.9. ^{1}H-^{13}C HSQC spectrum for 1-(quinolin-2-yl)-1'-(α-hydroxyethyl)ferrocene **(6b)**.

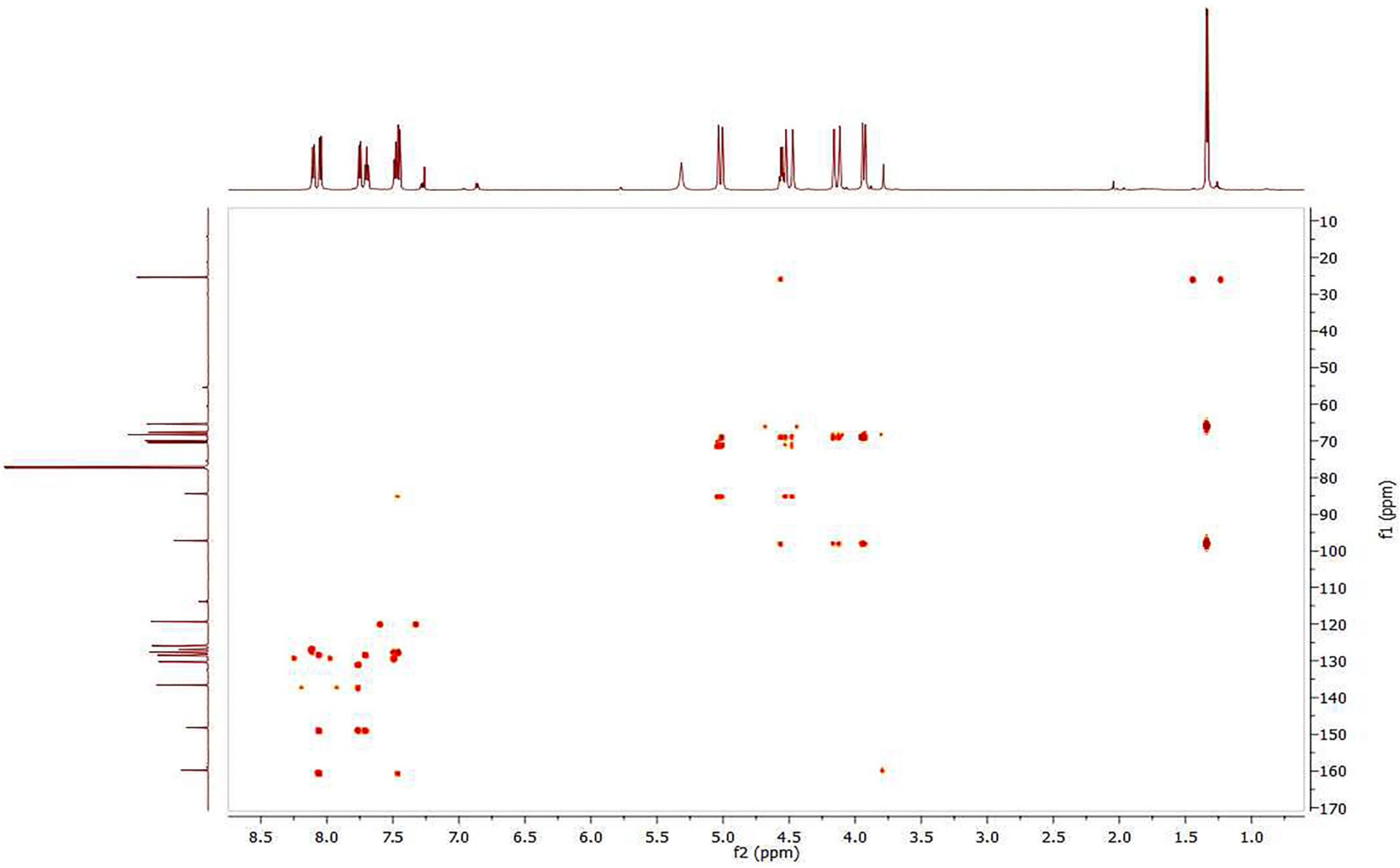

Figure S3.10. ^1H-^{13}C HMBC spectrum for 1-(quinolin-2-yl)-1'-(α-hydroxyethyl)ferrocene **(6b)**.

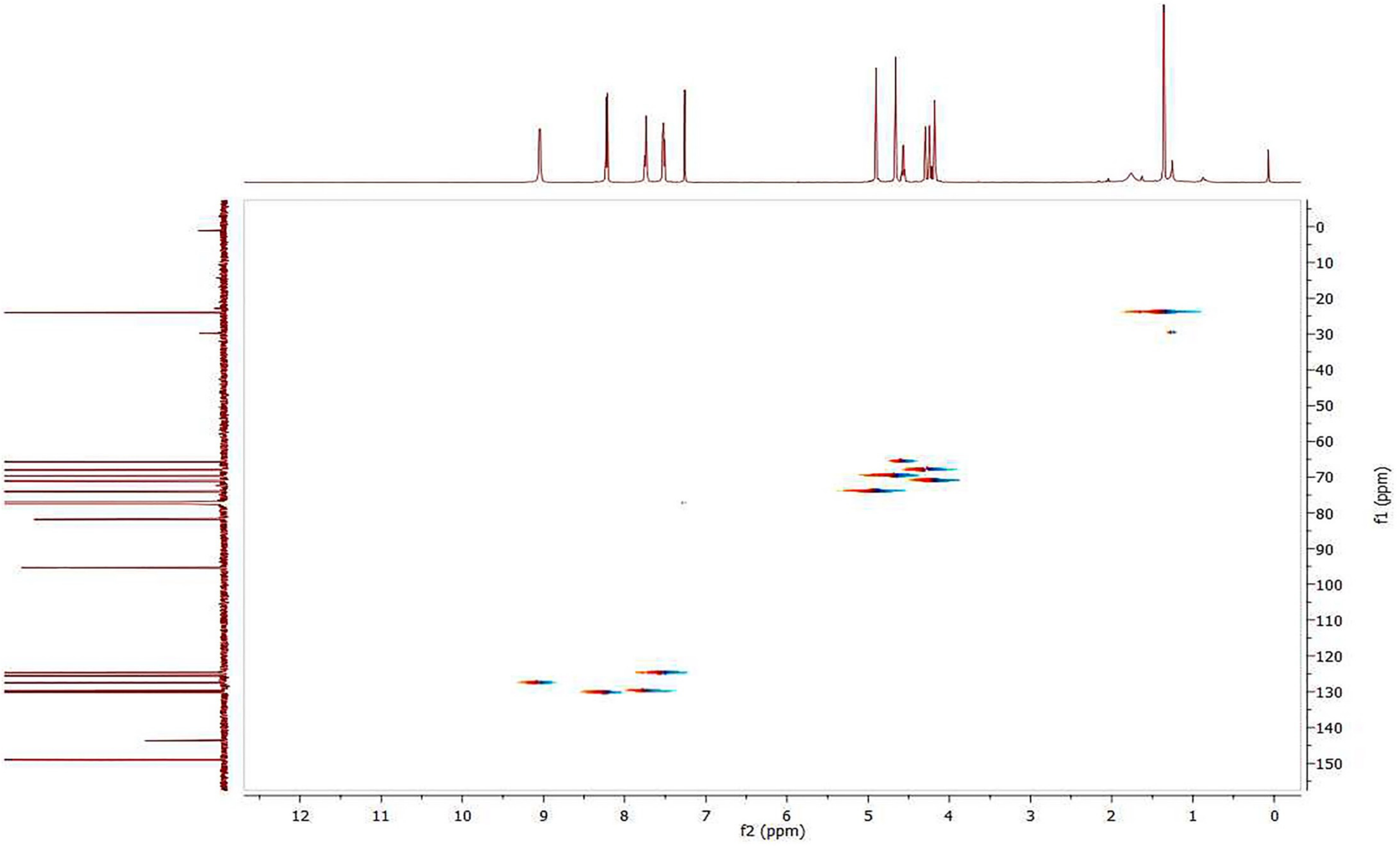

Figure S3.11. ^{1}H-^{13}C HSQC spectrum for 1-(acridin-9-yl)-1'-(α-hydroxyethyl)ferrocene **(6c)**.

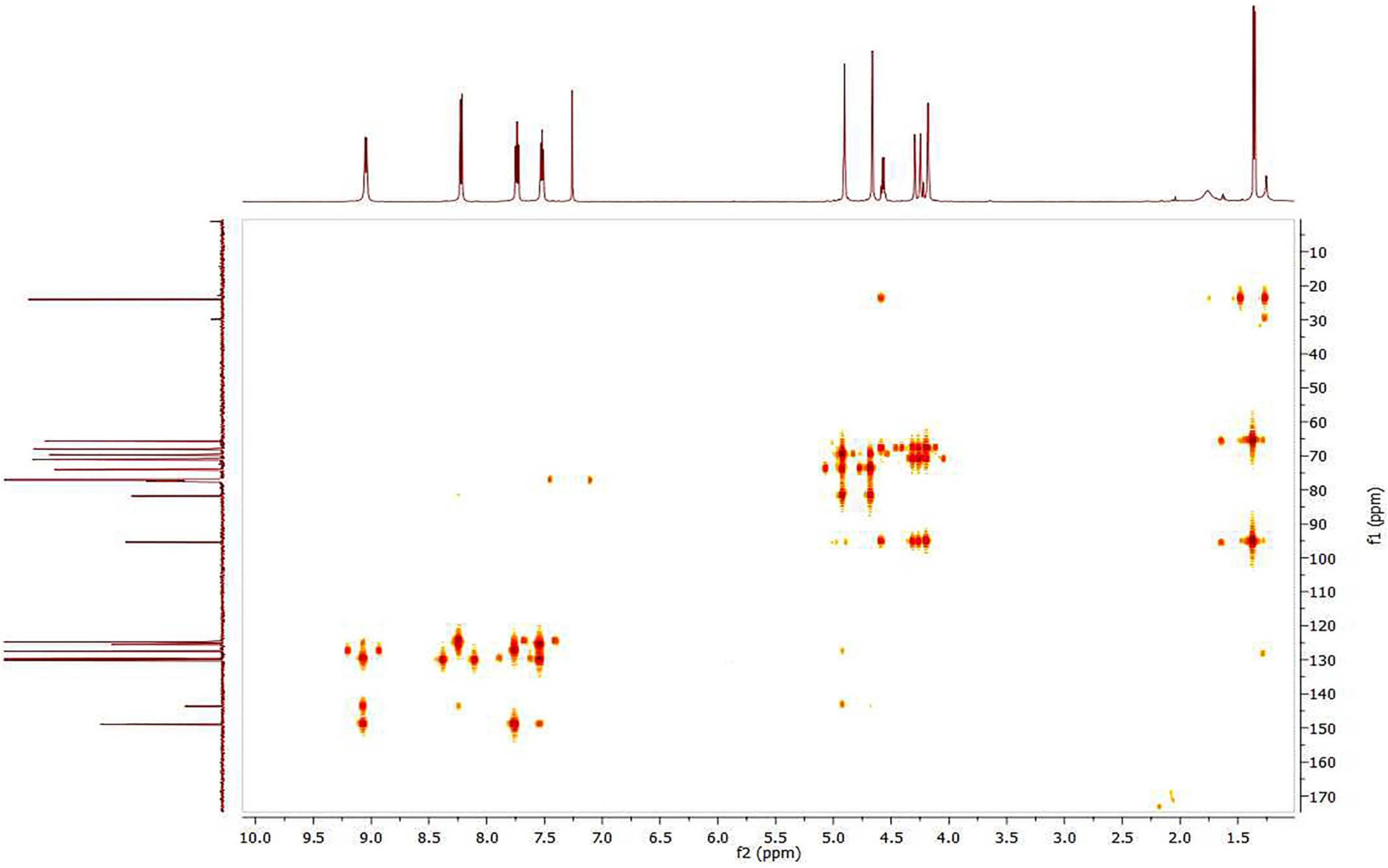

Figure S3.12. ^1H-^{13}C HMBC spectrum for 1-(acridin-9-yl)-1'-(α-hydroxyethyl)ferrocene **(6c)**.

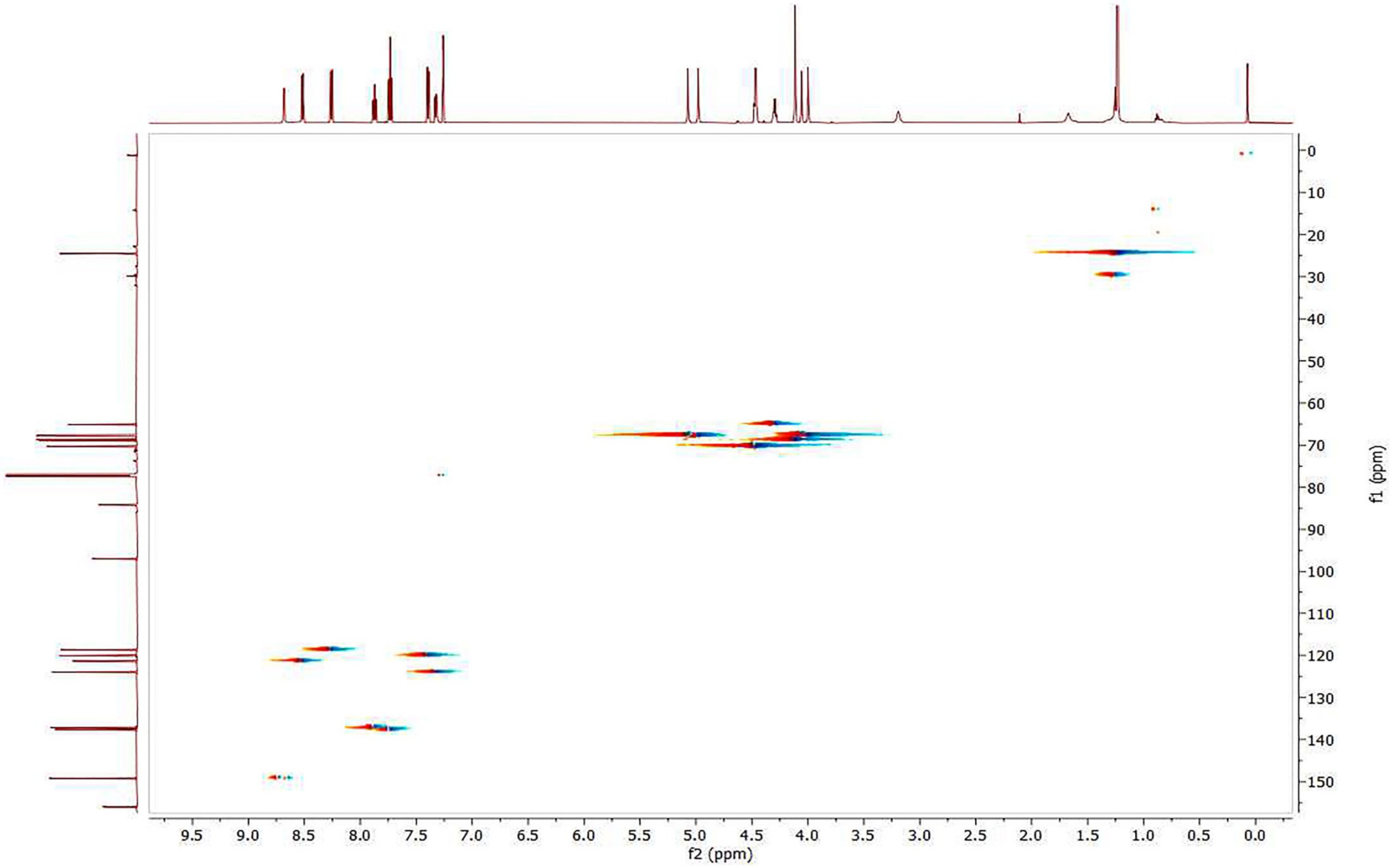

Figure S3.13. ^{1}H-^{13}C HSQC spectrum for 1-(2,2'-bipyridin-6-yl)-1'-(α-hydroxyethyl)ferrocene **(6d)**.

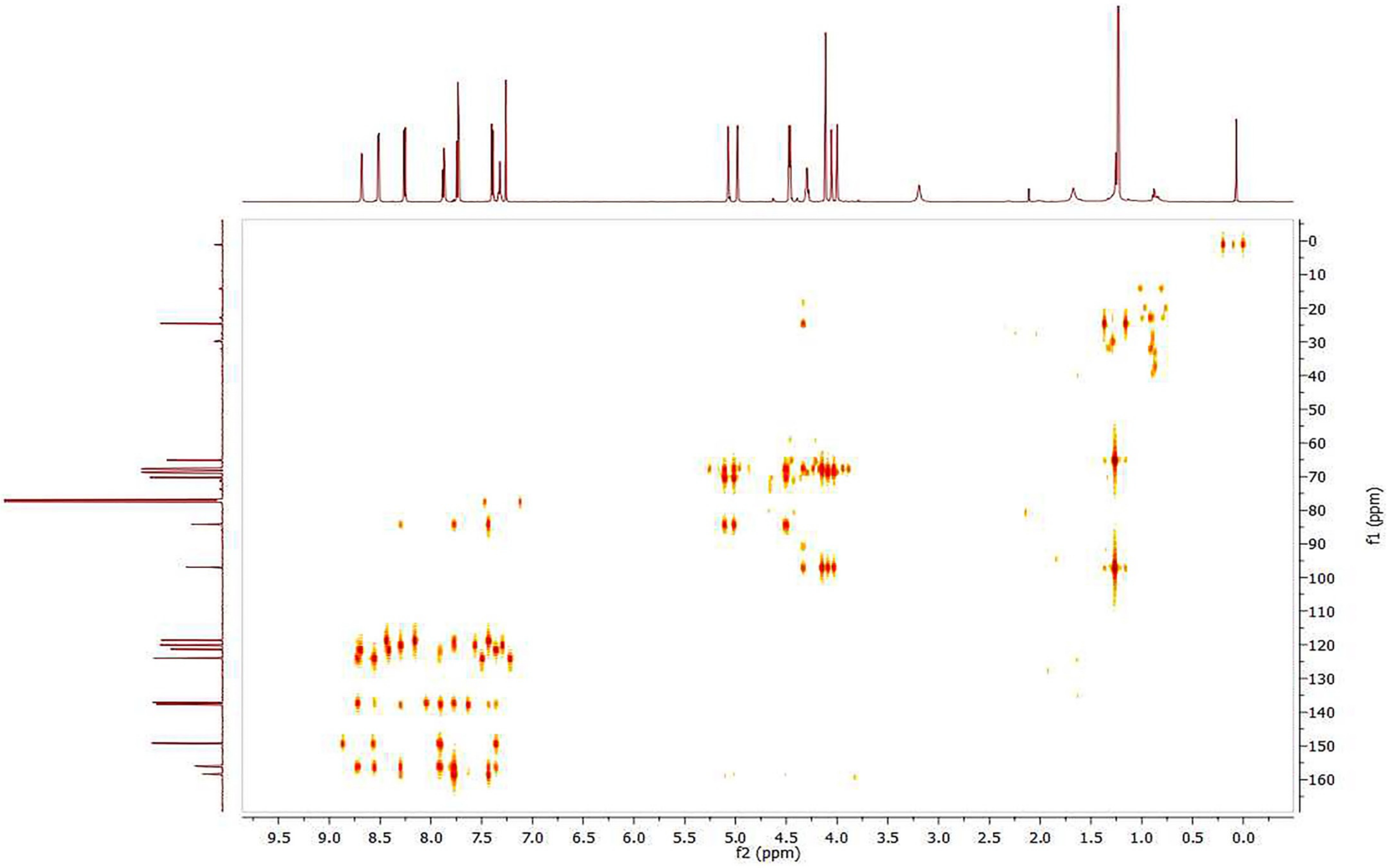

Figure S3.14. ^{1}H-^{13}C HMBC spectrum for 1-(2,2'-bipyridin-6-yl)-1'-(α-hydroxyethyl)ferrocene **(6d)**.

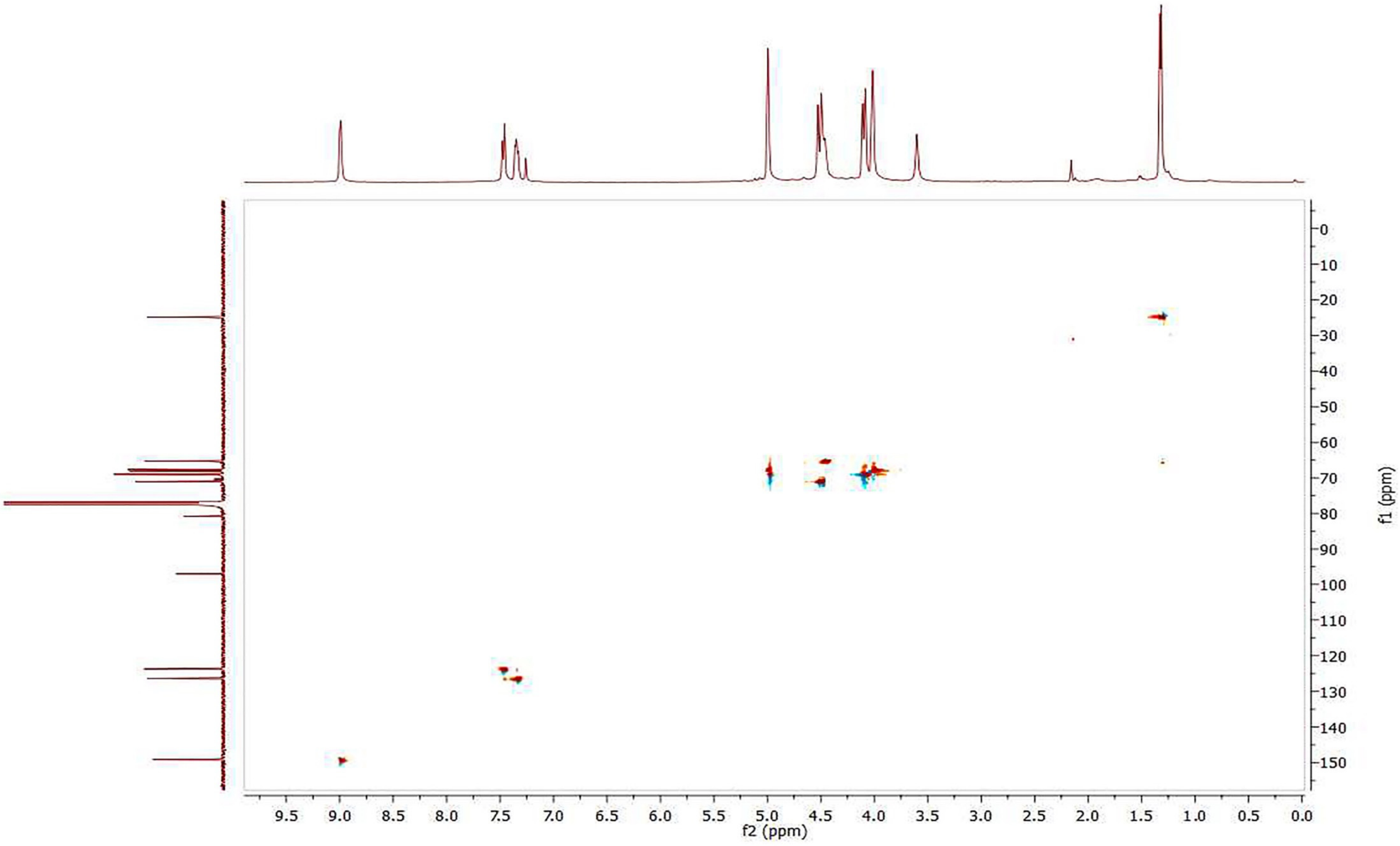

Figure S3.15. ^{1}H-^{13}C HSQC spectrum for 1-(pyridazin-4-yl)-1'-(α-hydroxyethyl)ferrocene **(6e)**.

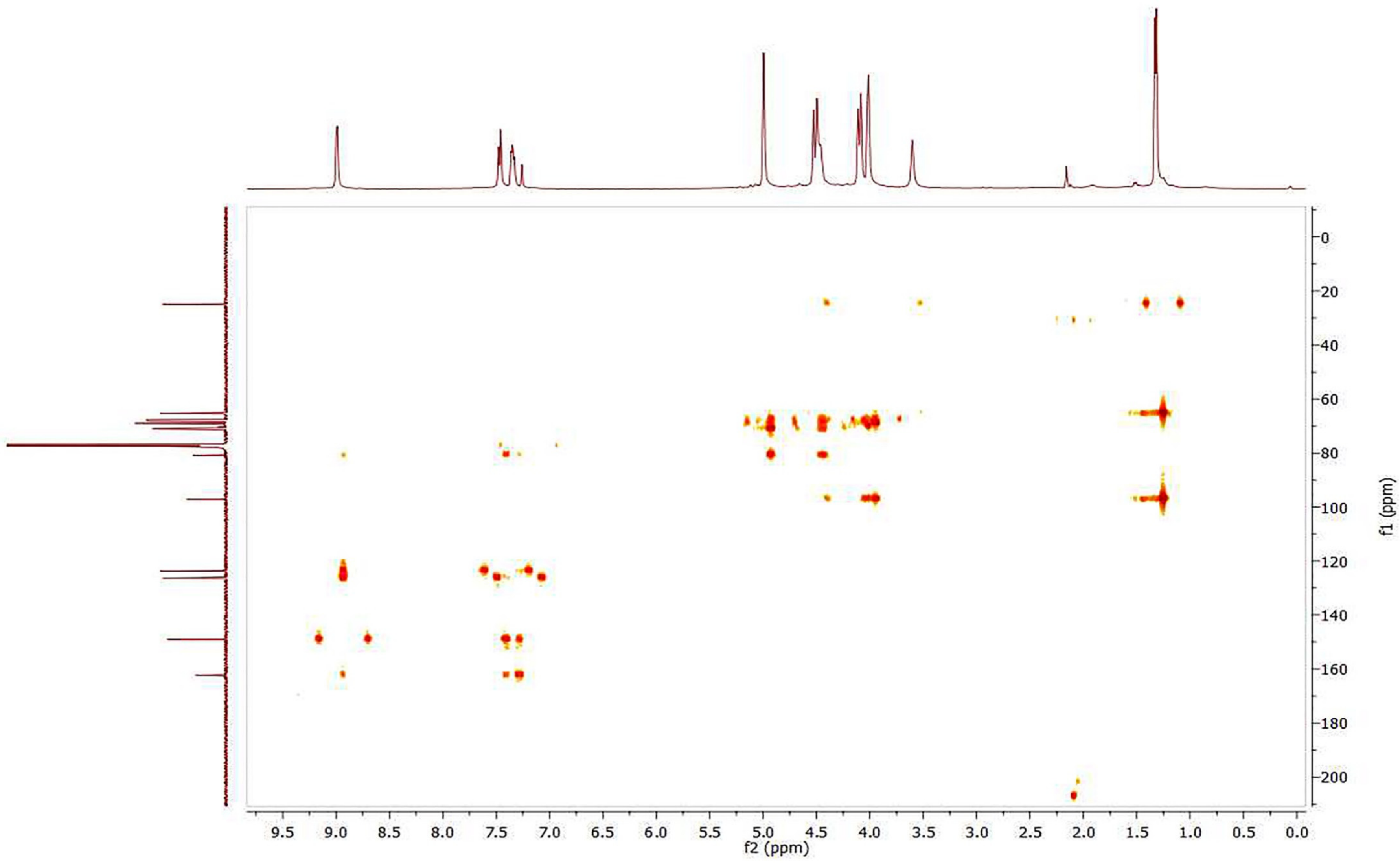

Figure S3.16. ^{1}H-^{13}C HMBC spectrum 1-(pyridazin-4-yl)-1'-(α-hydroxyethyl)ferrocene **(6e)**.

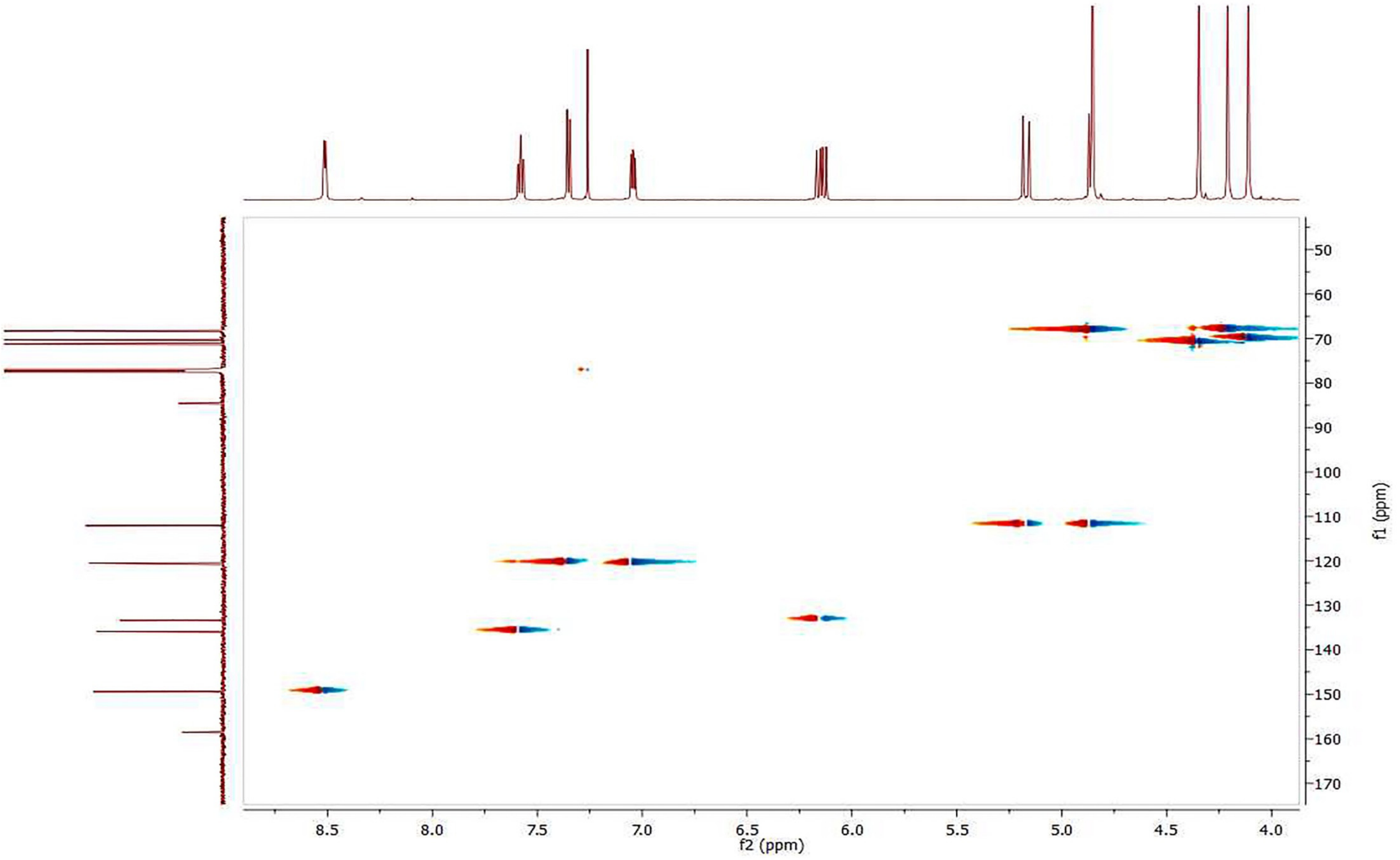

Figure S3.17. ^{1}H-^{13}C HSQC spectrum for 1-(pyridin-2-yl)-1'-vinylferrocene **(7a)**.

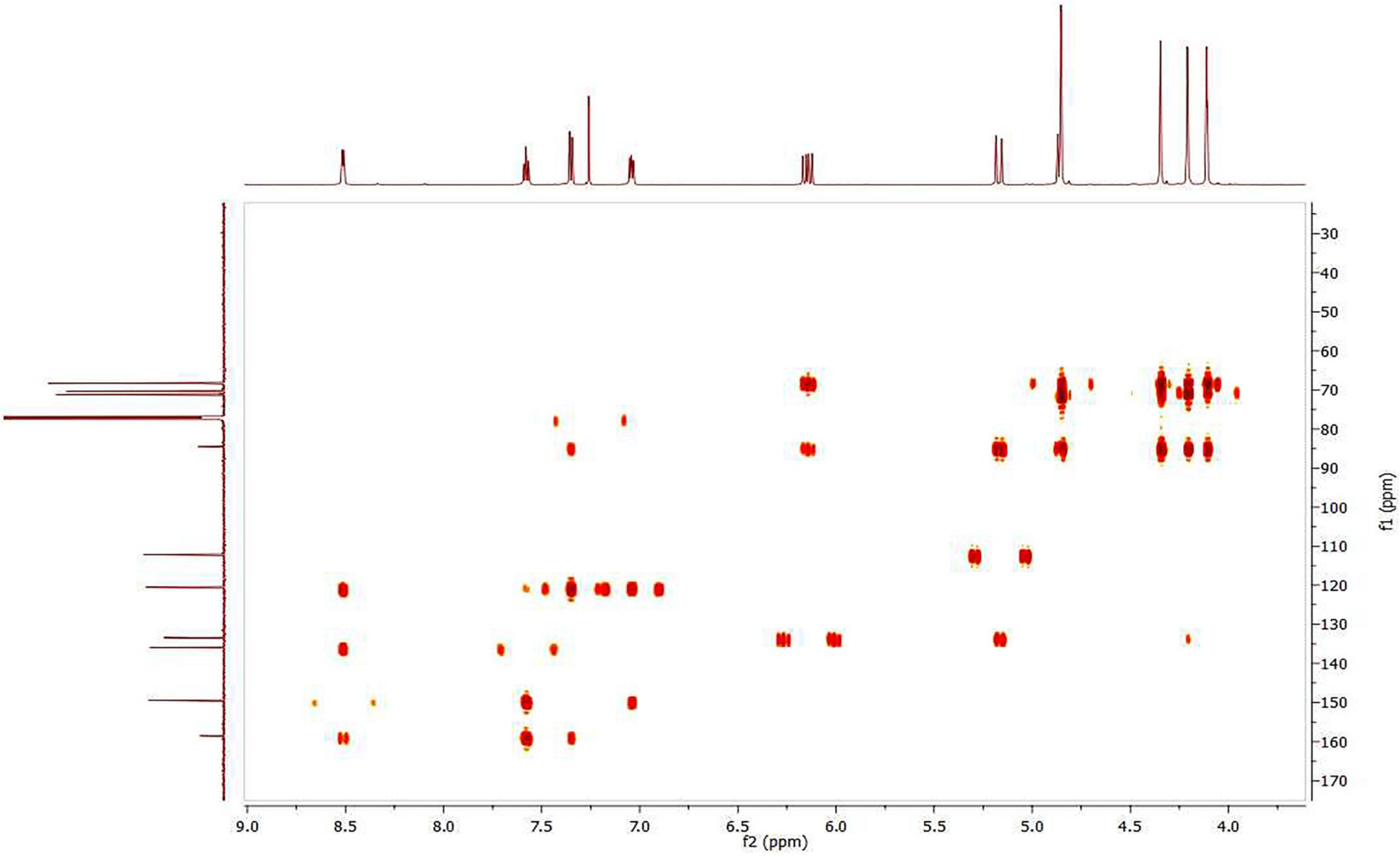

Figure S3.18. ^{1}H-^{13}C HMBC spectrum for 1-(pyridin-2-yl)-1'-vinylferrocene **(7a)**.

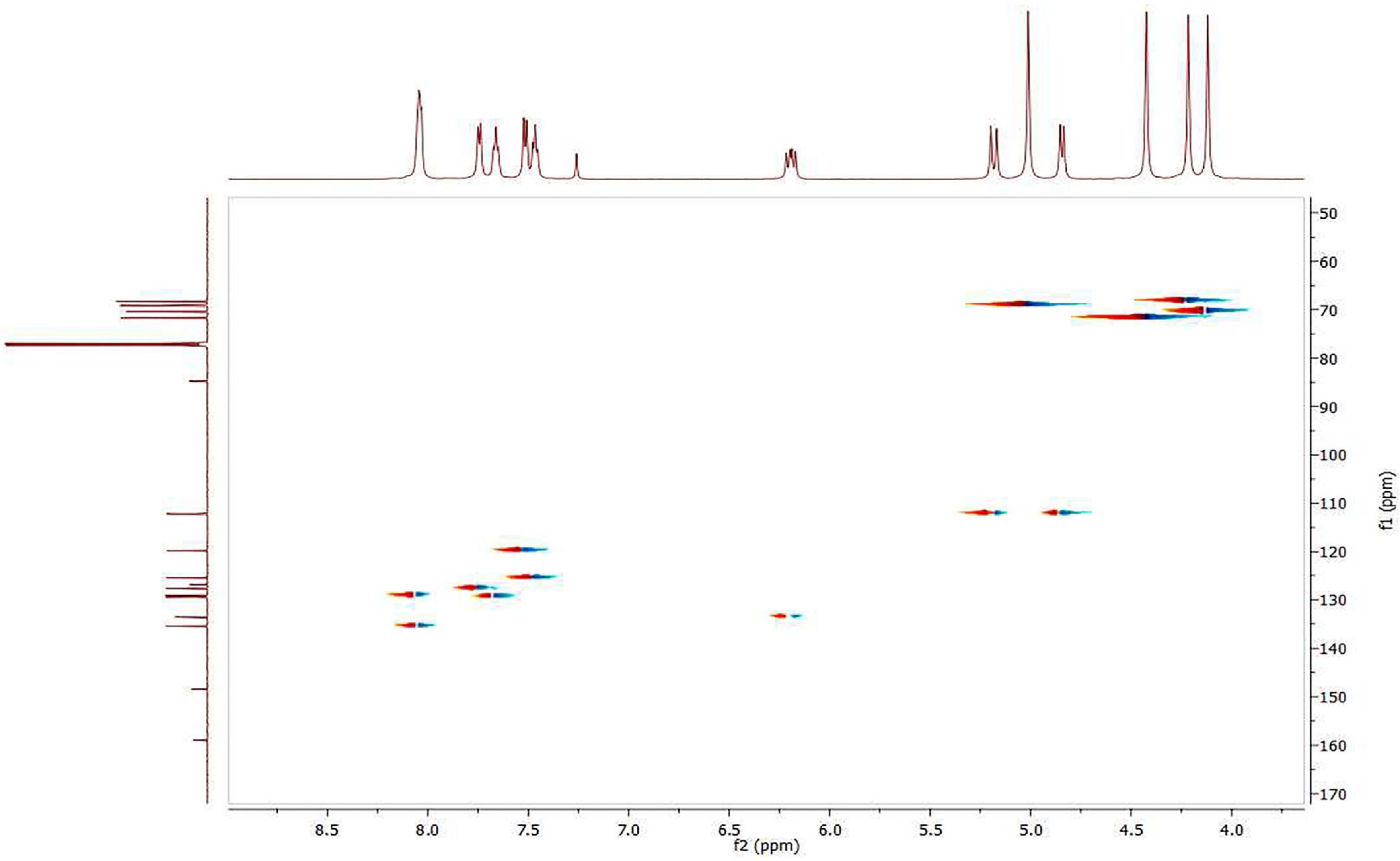

Figure S3.19. ^{1}H-^{13}C HSQC spectrum for 1-(quinolin-2-yl)-1'-vinylferrocene **(7b)**.

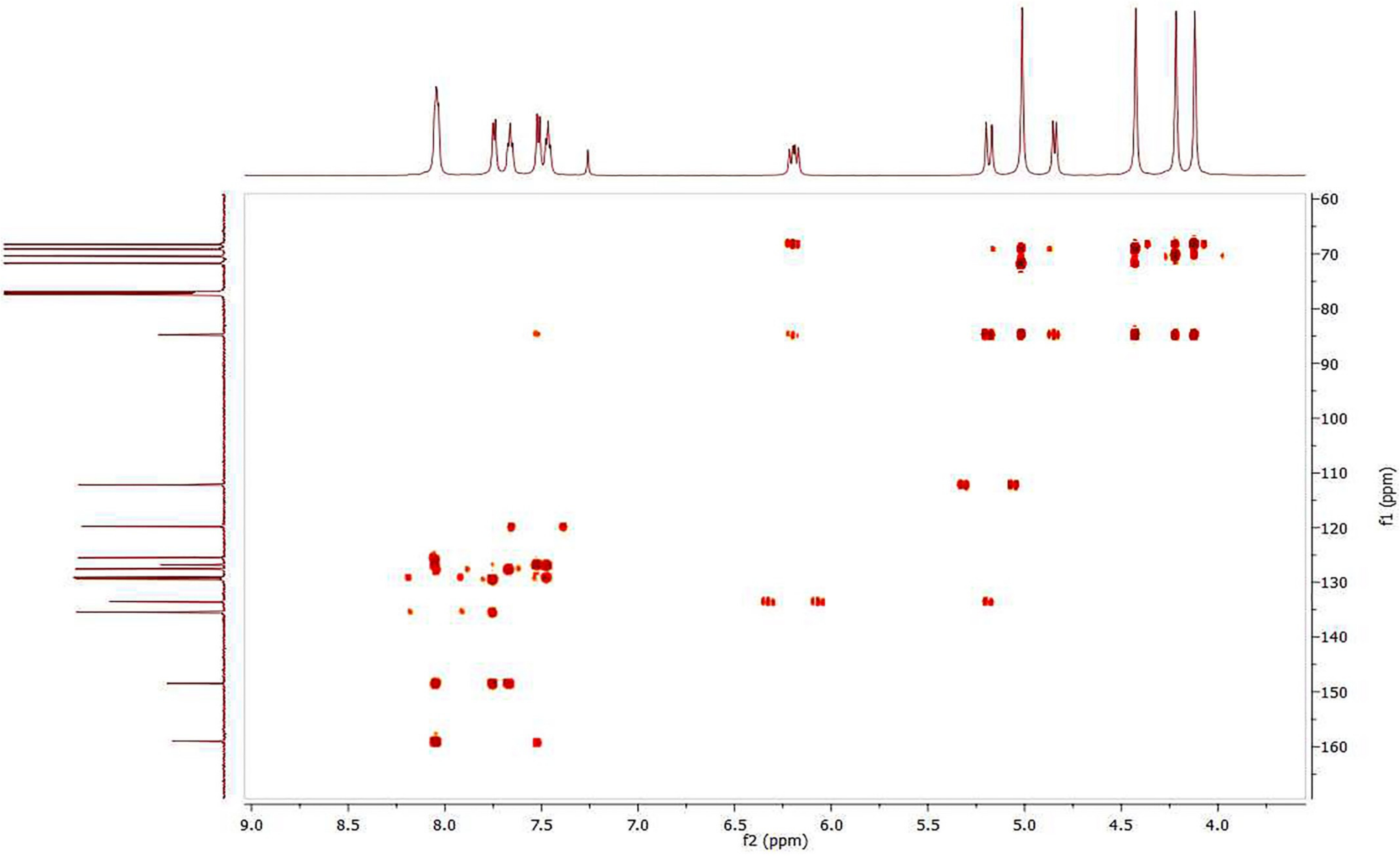

Figure S3.20. ^{1}H-^{13}C HMBC spectrum for 1-(quinolin-2-yl)-1'-vinylferrocene **(7b)**.

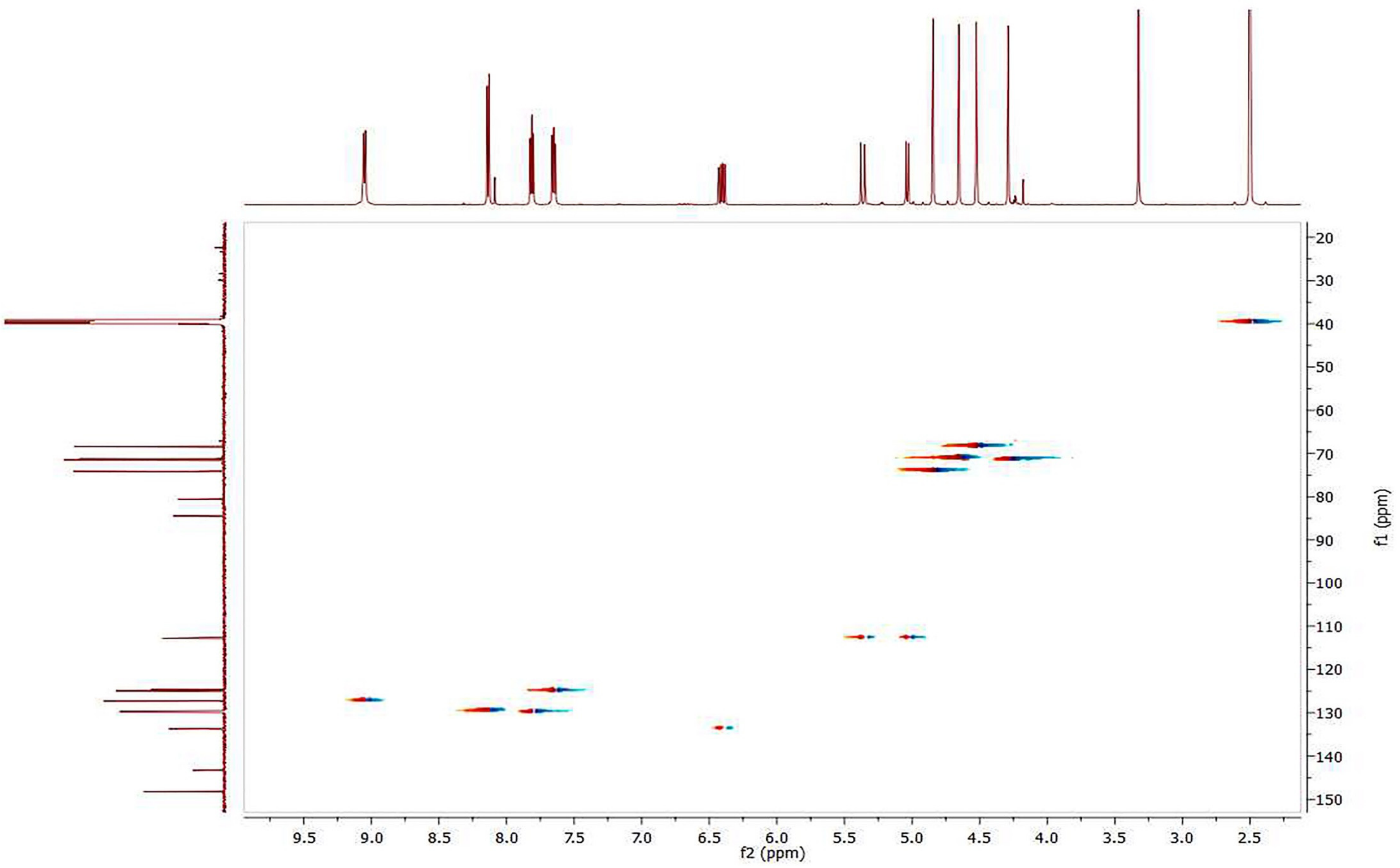

Figure S3.21. ^{1}H-^{13}C HSQC spectrum for 1-(acridin-9-yl)-1'-vinylferrocene **(7c)**.

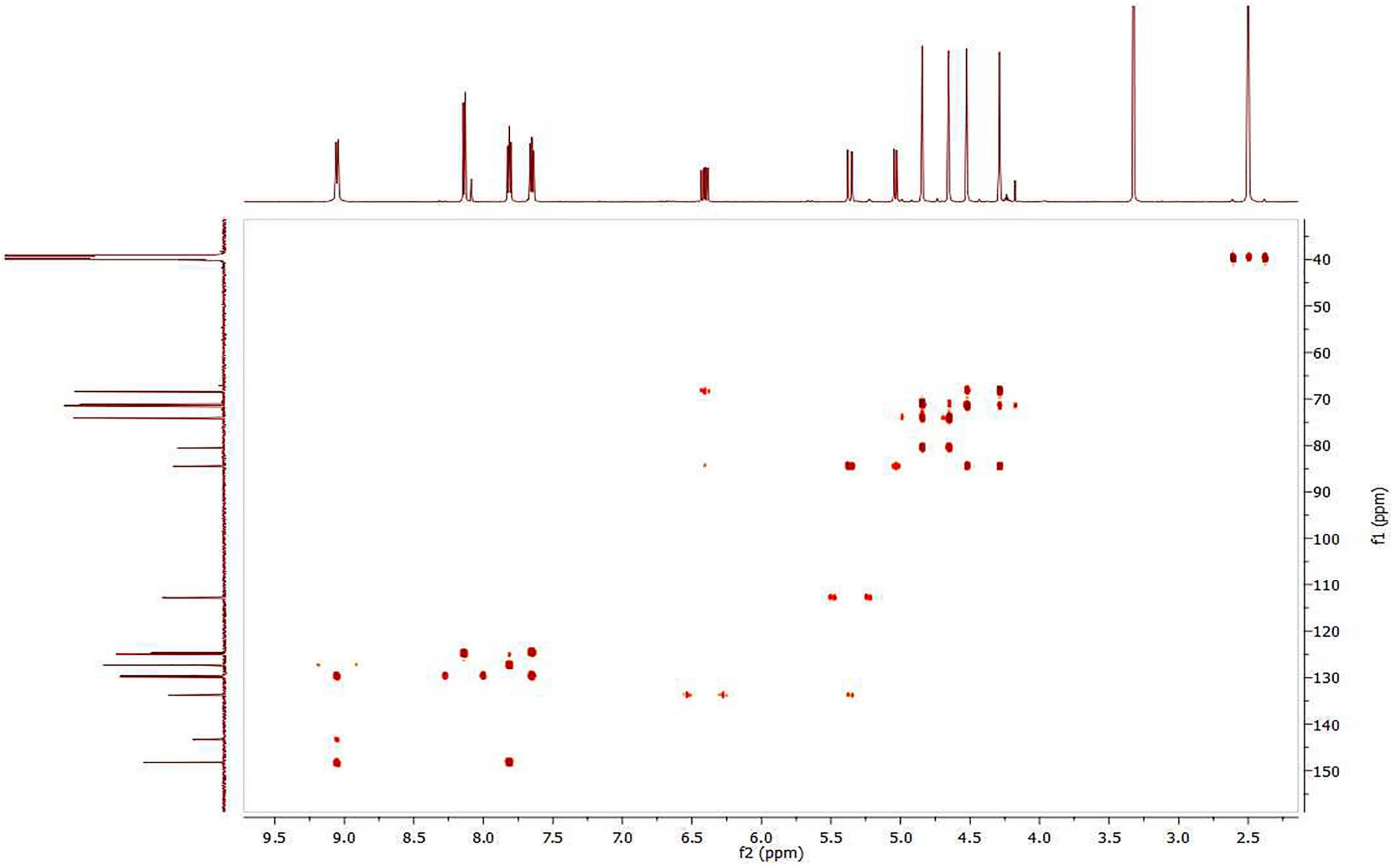

Figure S3.22. ^{1}H-^{13}C HMBC spectrum for 1-(acridin-9-yl)-1'-vinylferrocene **(7c)**.

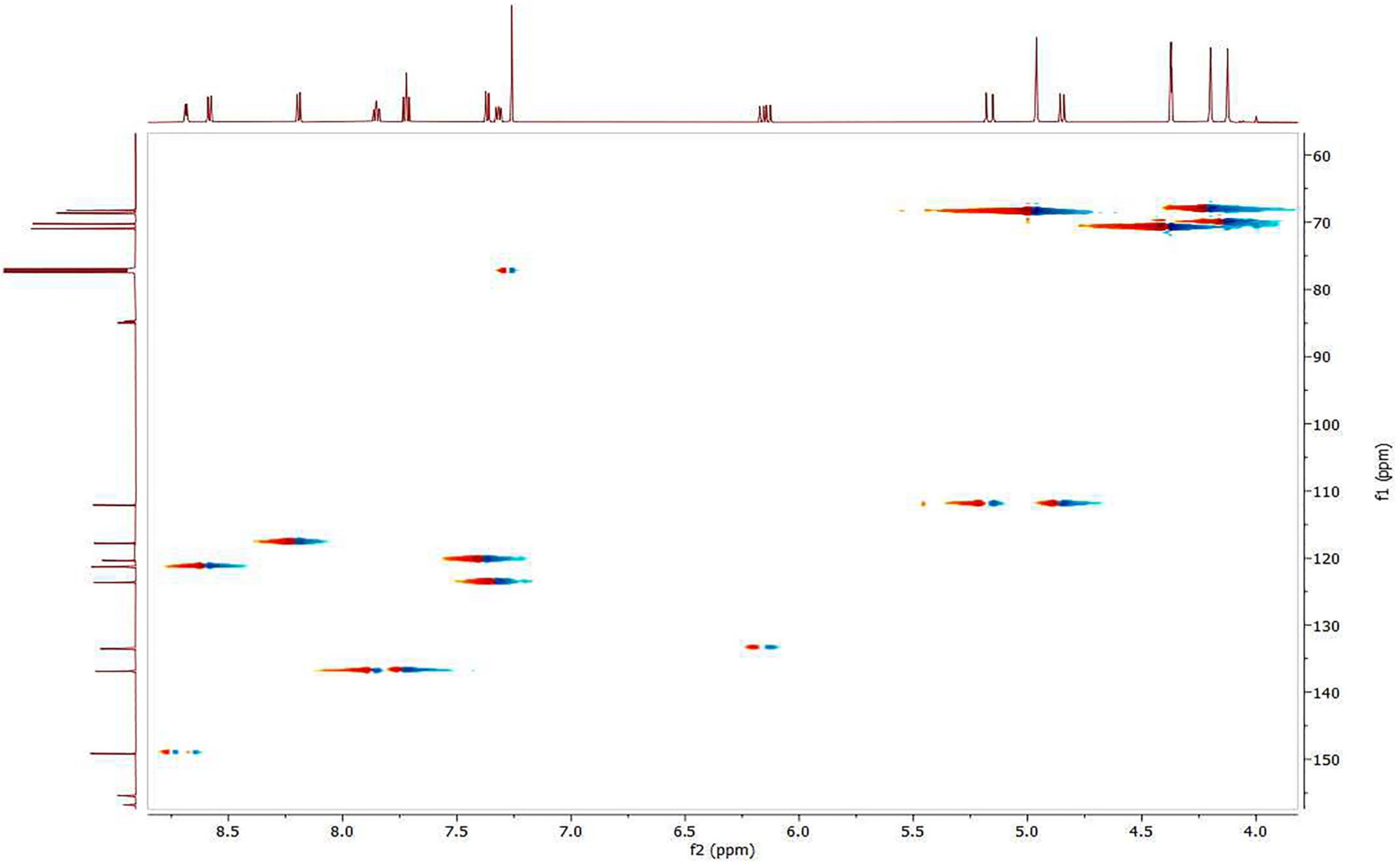

Figure S3.23. ^{1}H-^{13}C HSQC spectrum for 1-(2,2'-bipyridin-6-yl)-1'-vinylferrocene **(7d)**.

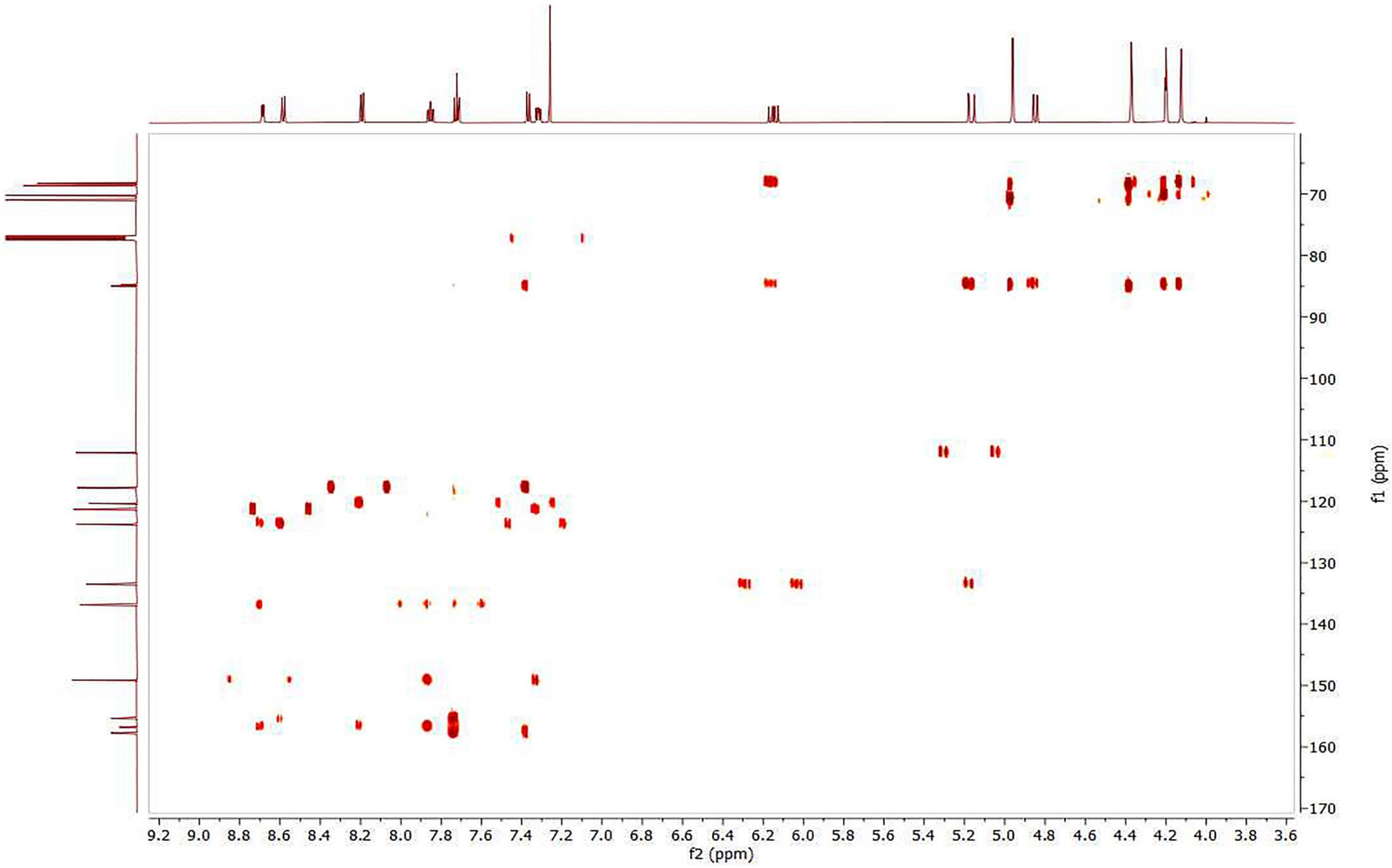

Figure S3.24. ^{1}H-^{13}C HMBC spectrum for 1-(2,2'-bipyridin-6-yl)-1'-vinylferrocene **(7d)**.

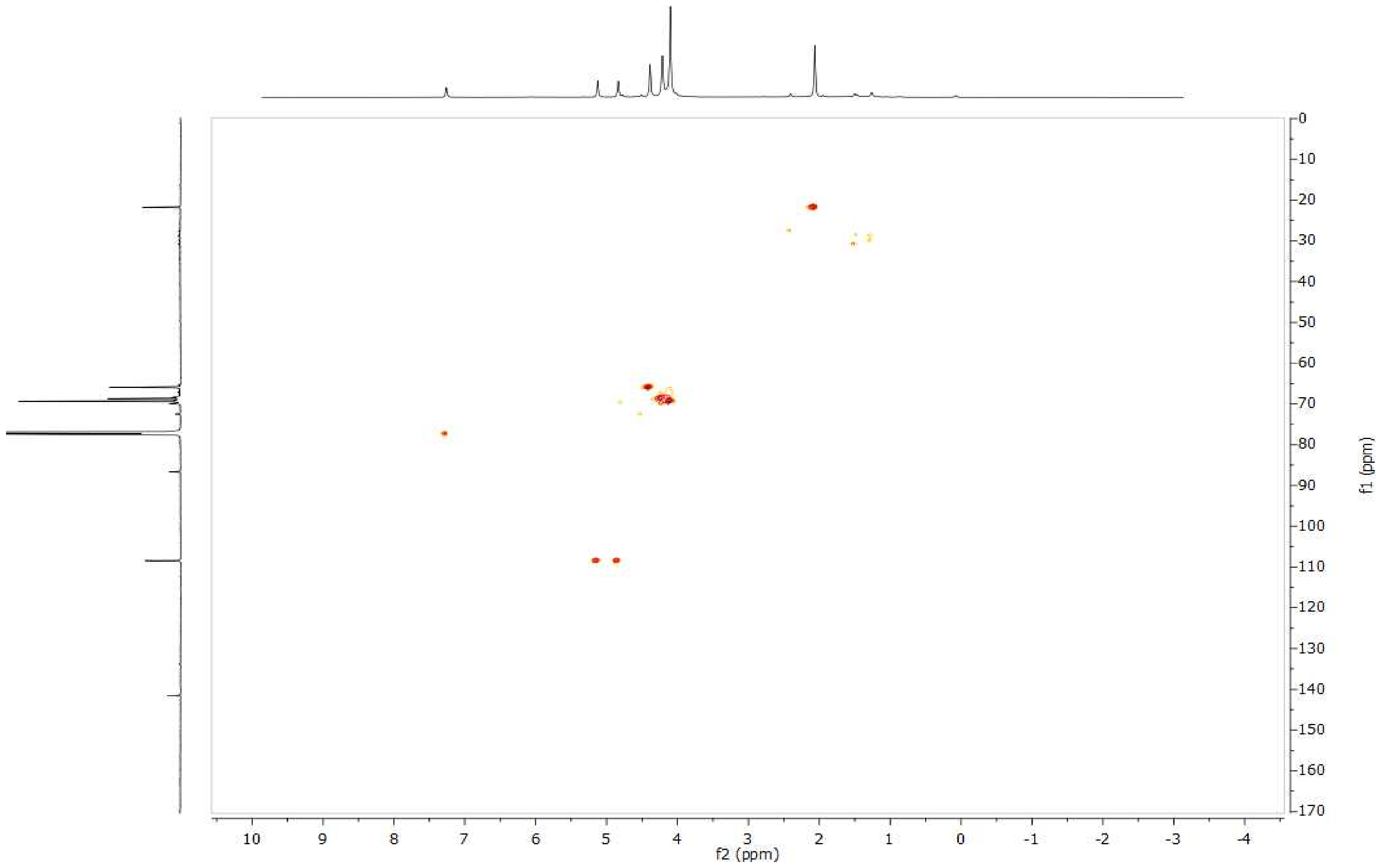

Figure S3.25. ^{1}H-^{13}C HSQC spectrum for Isopropenylferrocene **(8)**.

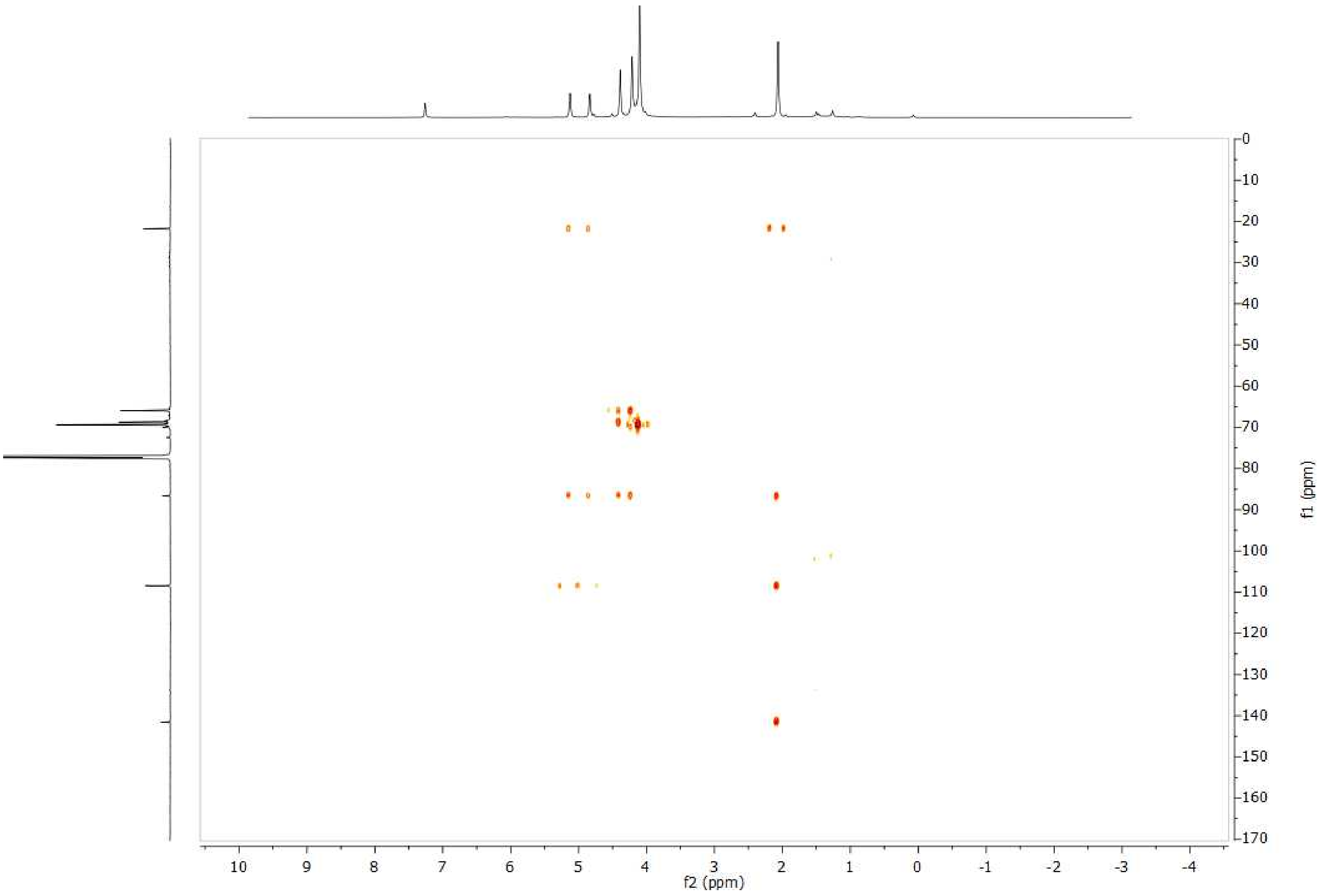

Figure S3.26. ^{1}H-^{13}C HMBC spectrum for Isopropenylferrocene **(8)**.

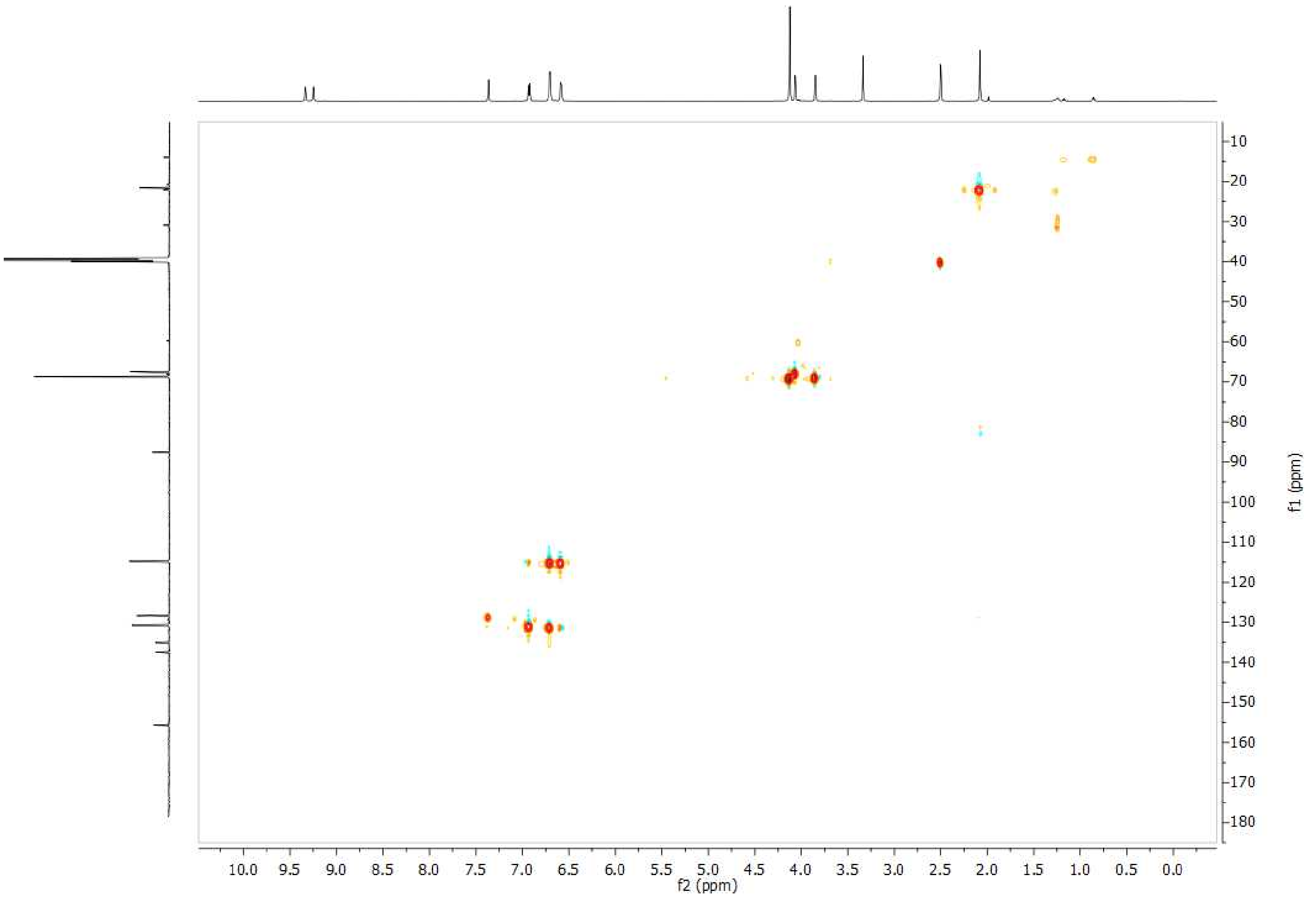

Figure S3.27. ^1H-^{13}C HSQC spectrum for 1,1-bis(4-hydroxyphenyl)-2-ferrocenylprop-1-ene **(10)**.

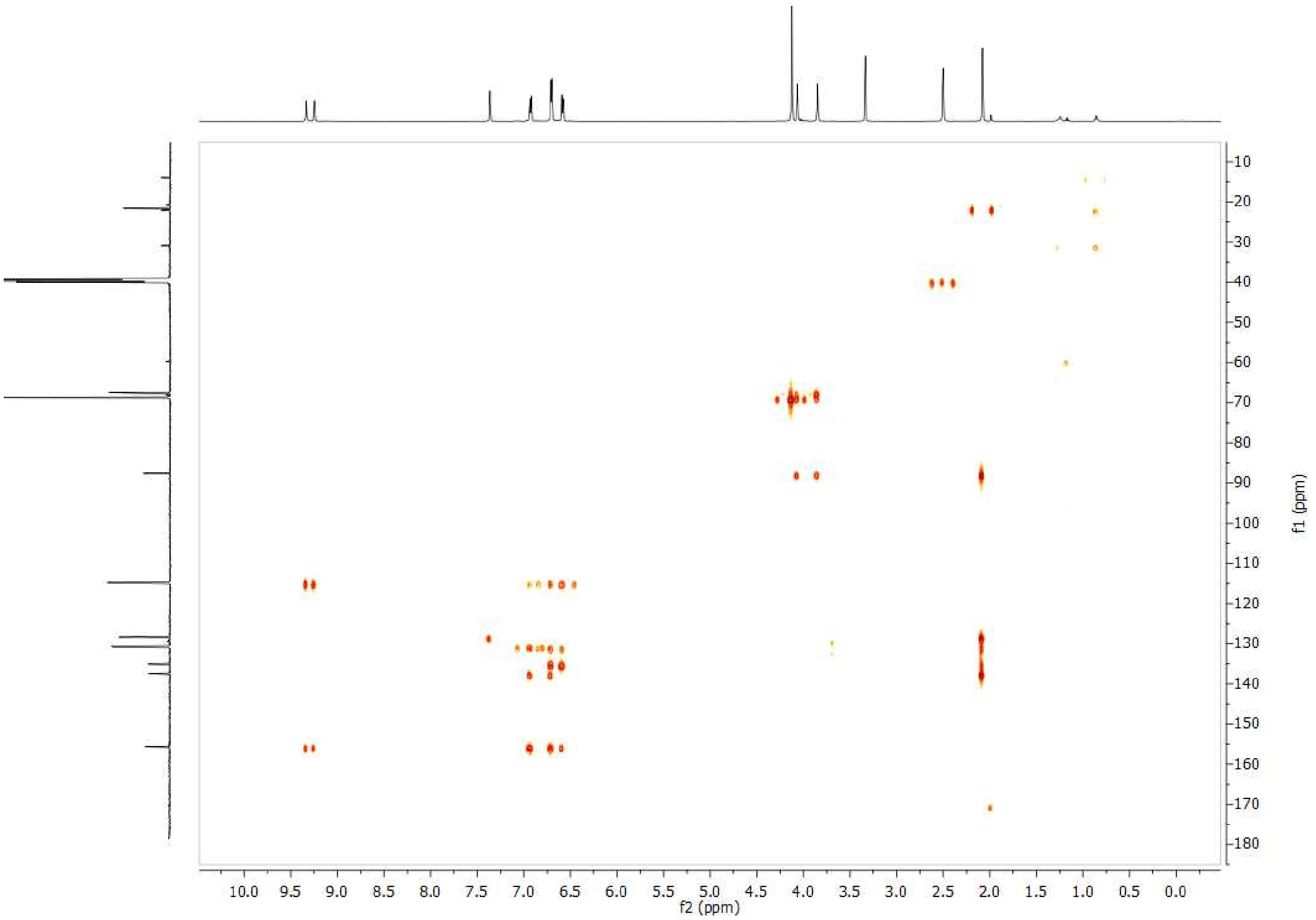

Figure S3.28. ^{1}H-^{13}C HMBC spectrum for 1,1-bis(4-hydroxyphenyl)-2-ferrocenylprop-1-ene **(10)**.

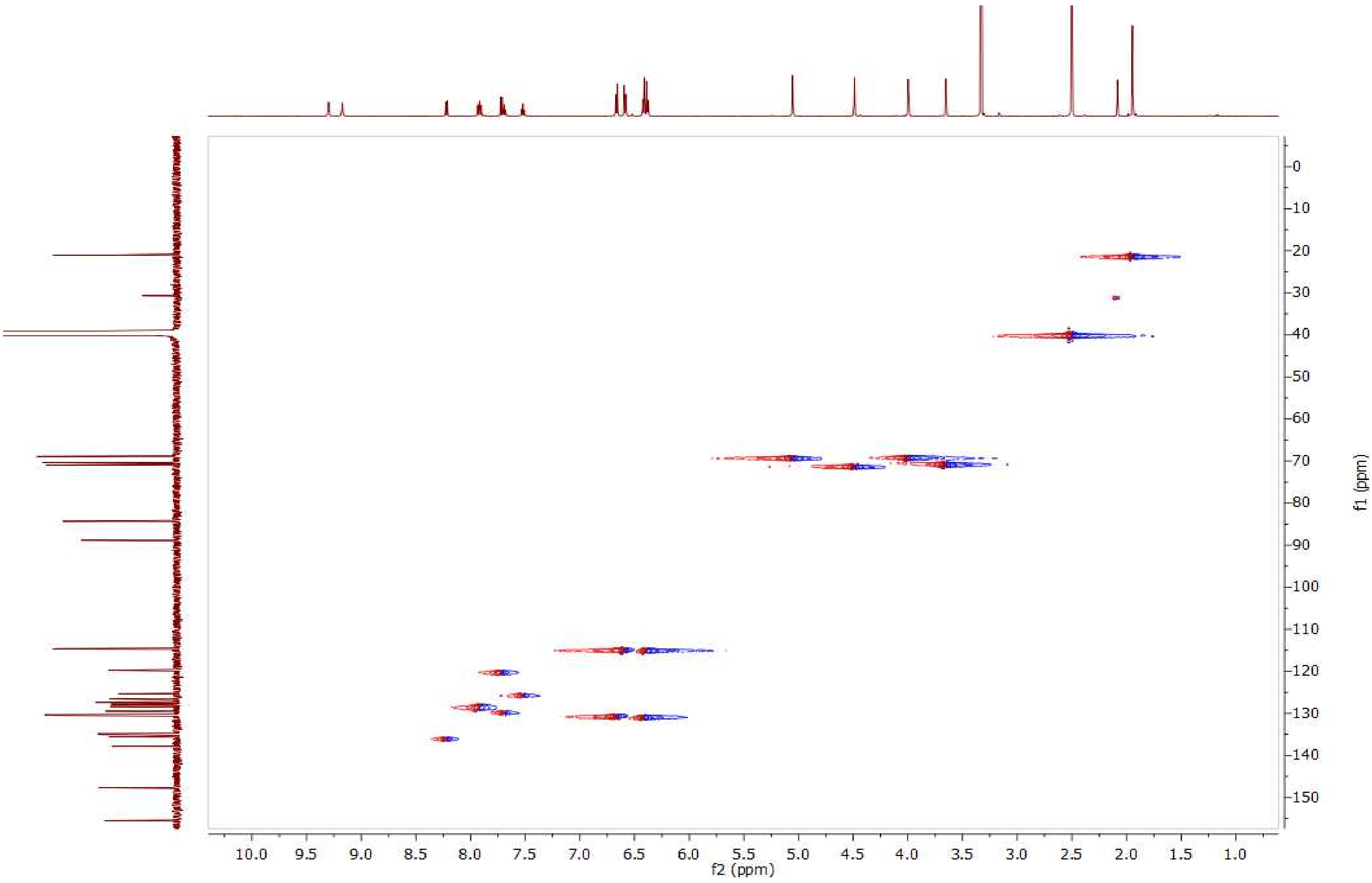

Figure S3.29. ^{1}H-^{13}C HSQC spectrum for 1-(quinolin-2-yl)-1´-{1-[bis(4-hydroxyphenyl)methylene]ethyl}ferrocene **(11)**.

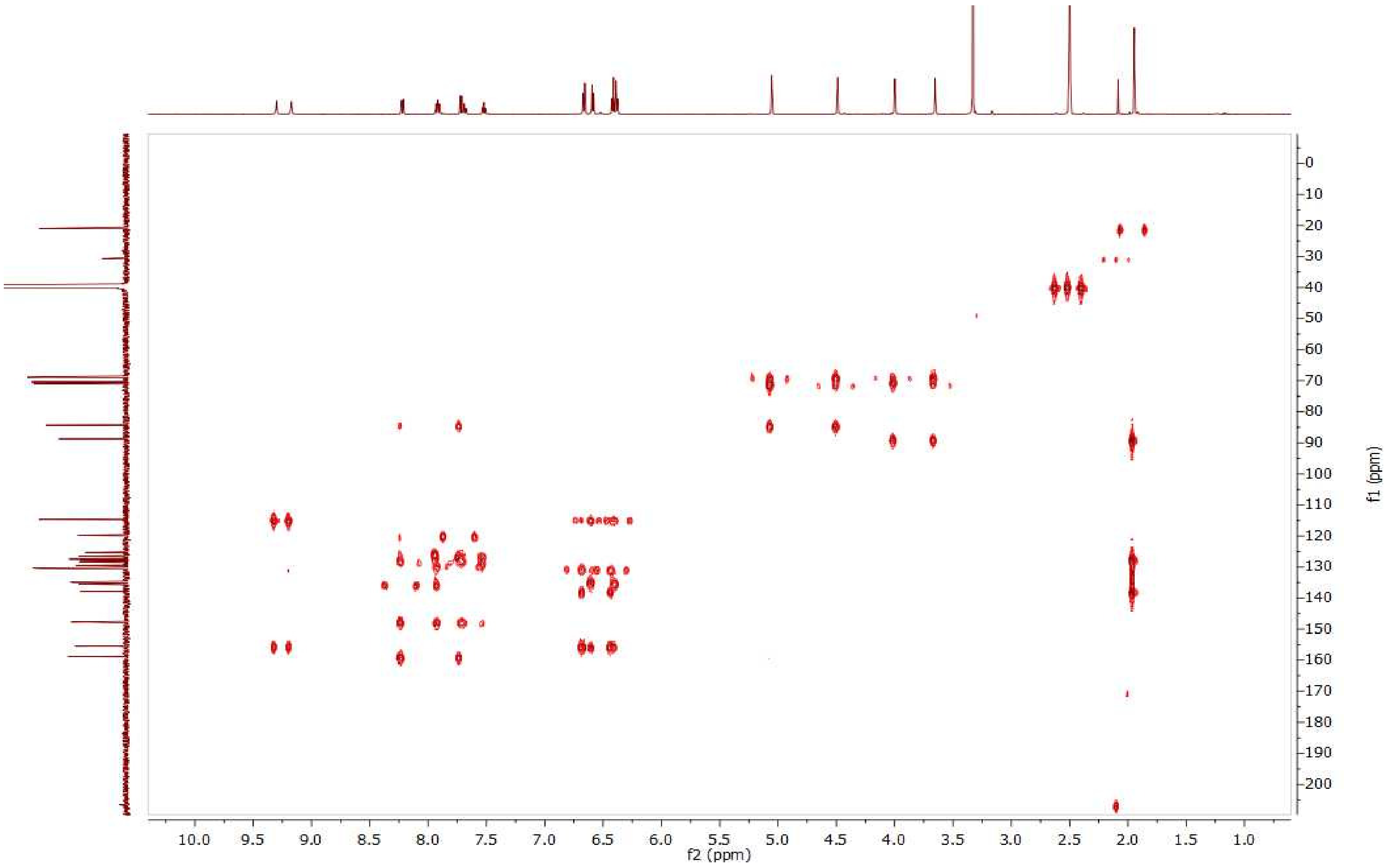

Figure S3.30. ^{1}H-^{13}C HMBC spectrum for 1-(quinolin-2-yl)-1´-{1-[bis(4-hydroxyphenyl)methylene]ethyl}ferrocene **(11)**.

Section S4. IR spectra

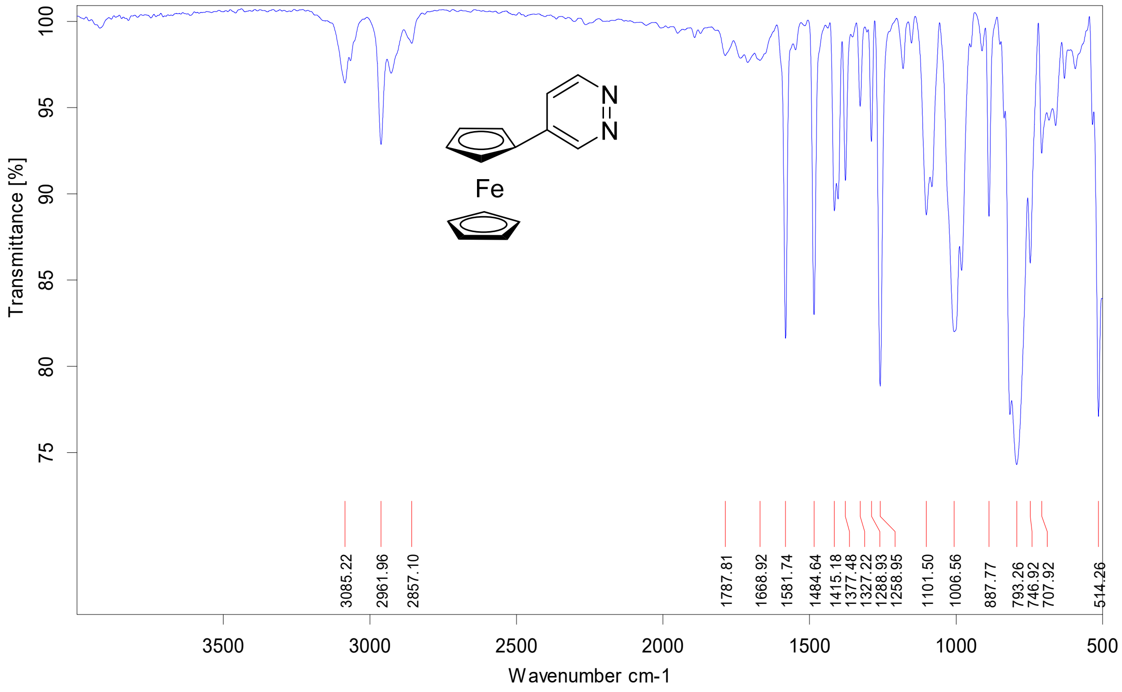

Figure S4.1. IR spectrum for 1-(pyridazin-4-yl)ferrocene **(1e)**.

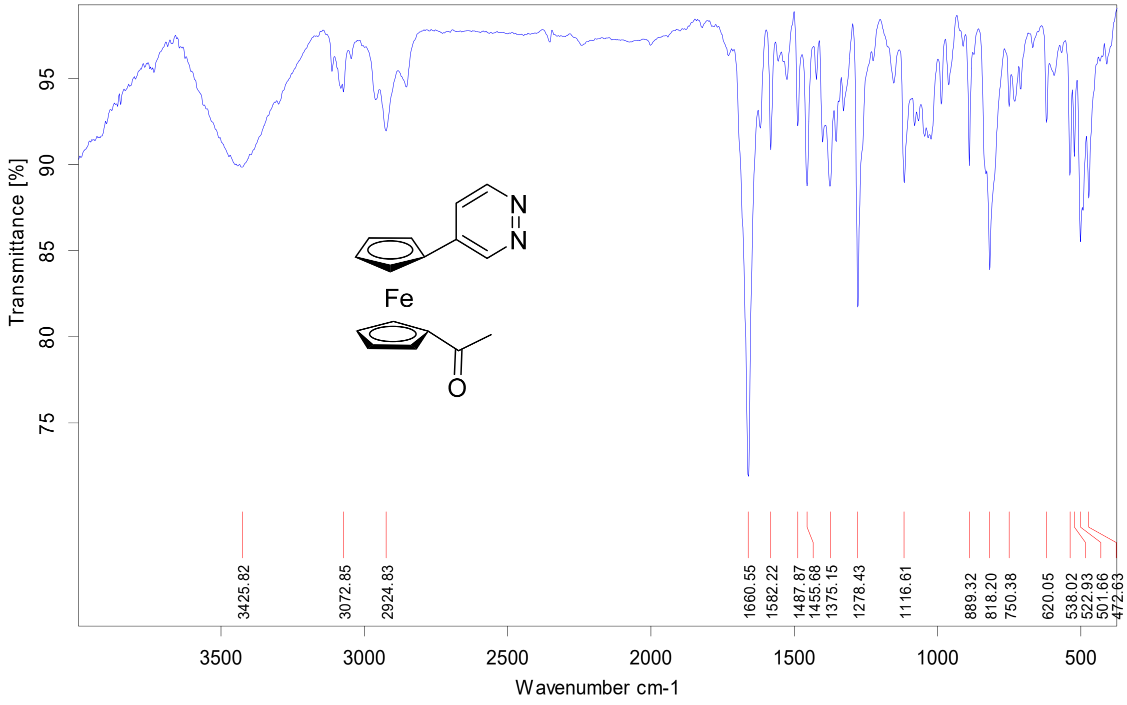

Figure S4.2. IR spectrum for 1-acetyl-1'-(pyridazin-4-yl)ferrocene **(2e)**.

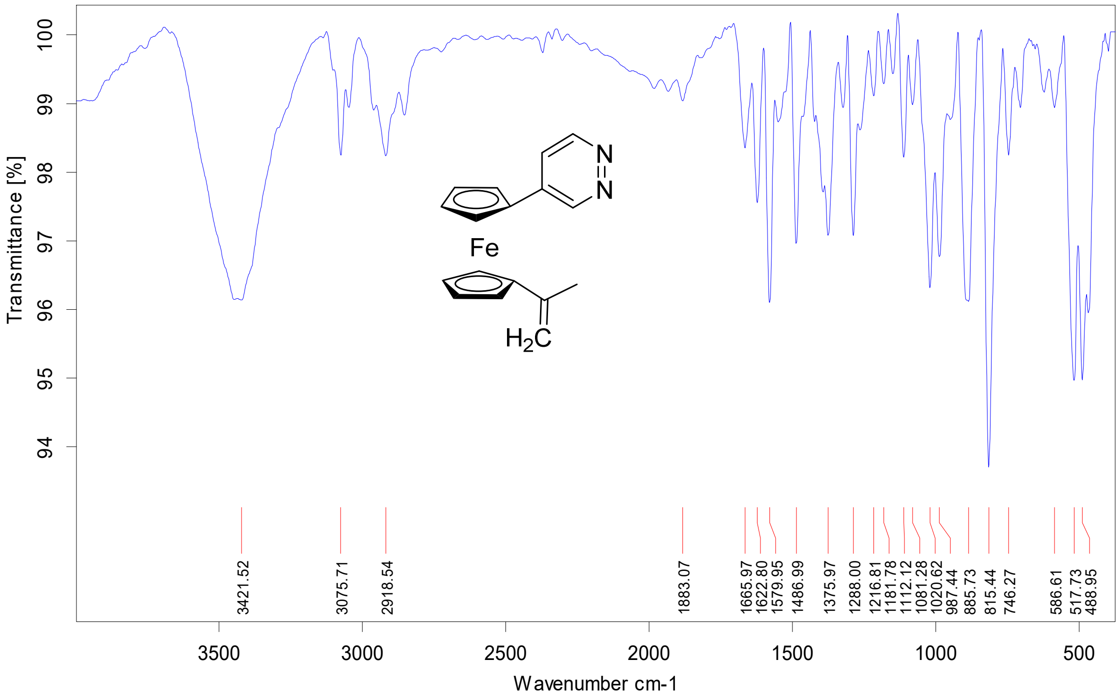

Figure S4.3. IR spectrum for 1-isopropenyl-1'-(pyridazin-4-yl)ferrocene **(5e)**.

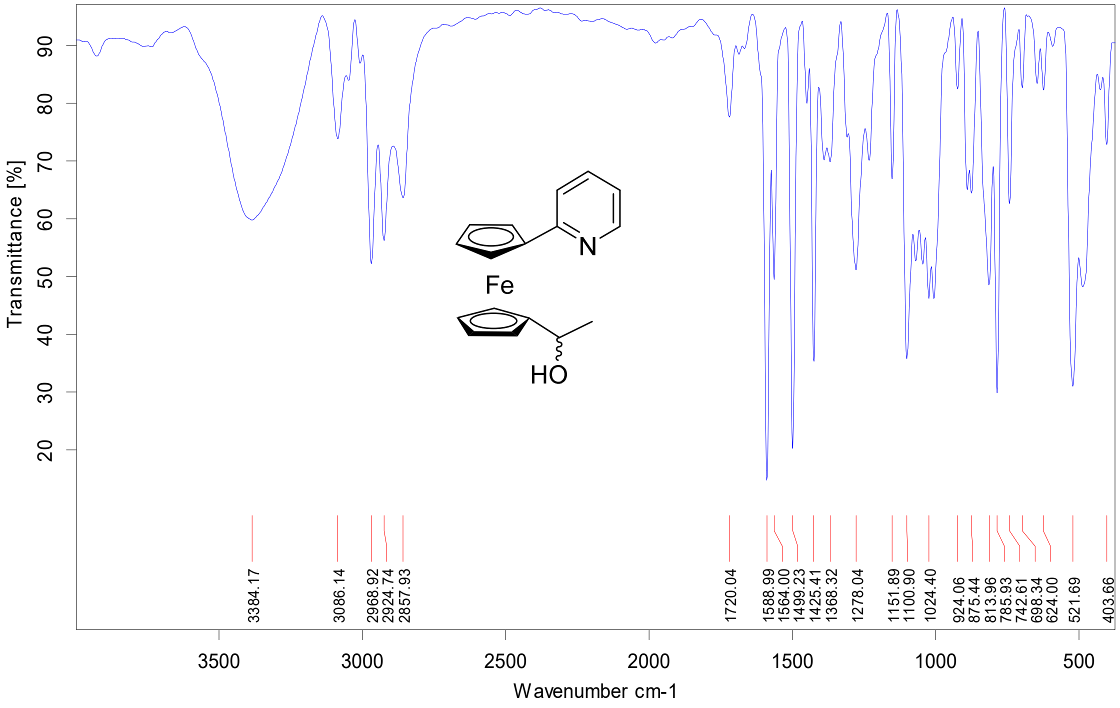

Figure S4.4. IR spectrum for 1-(pyridin-2-yl)-1'-(α-hydroxyethyl)ferrocene **(6a)**.

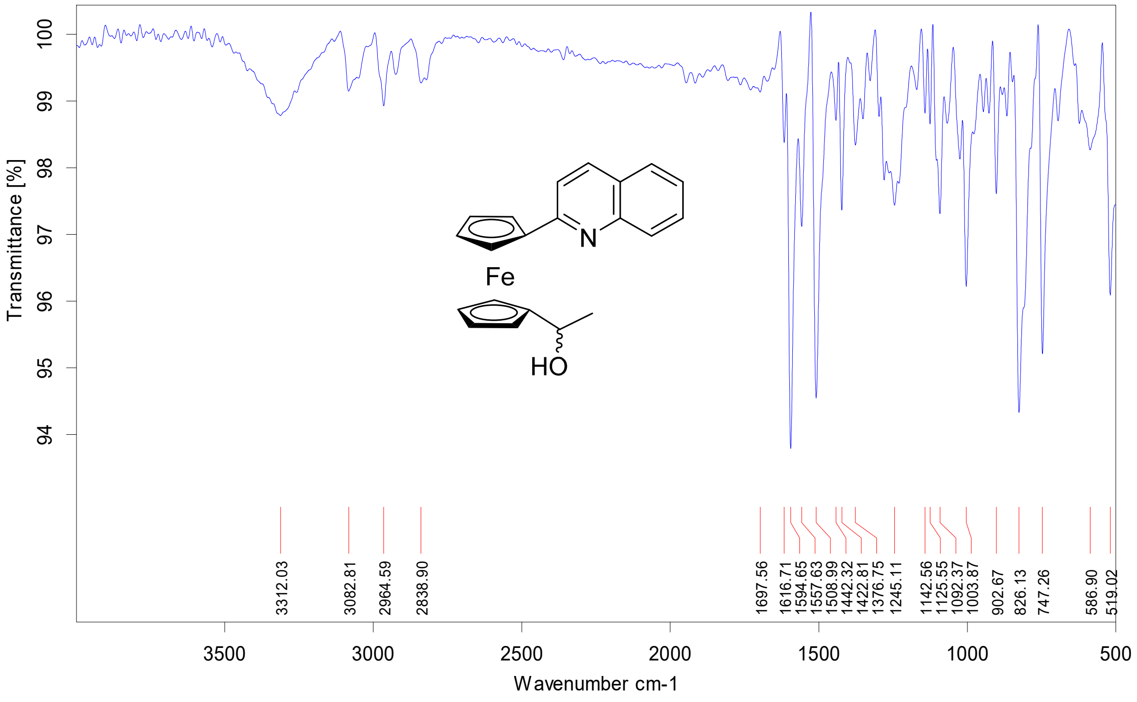

Figure S4.5. IR spectrum for 1-(quinolin-2-yl)-1'-(α-hydroxyethyl)ferrocene **(6b)**.

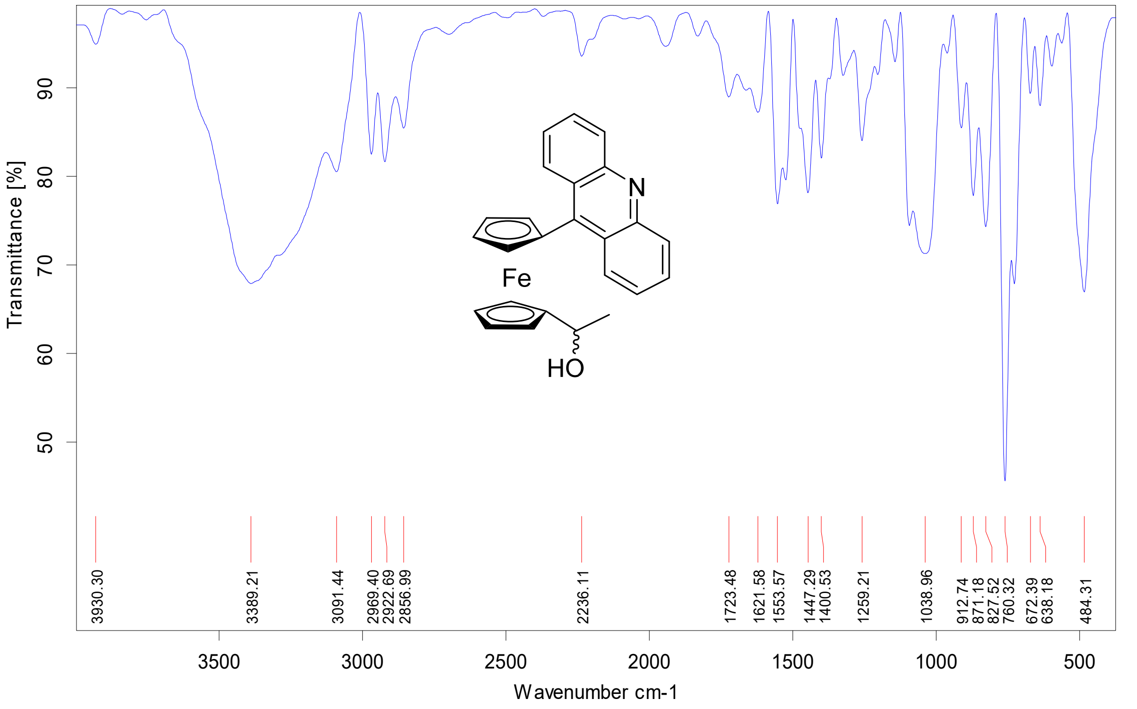

Figure S4.6. IR spectrum for 1-(acridin-9-yl)-1'-(α-hydroxyethyl)ferrocene **(6c)**.

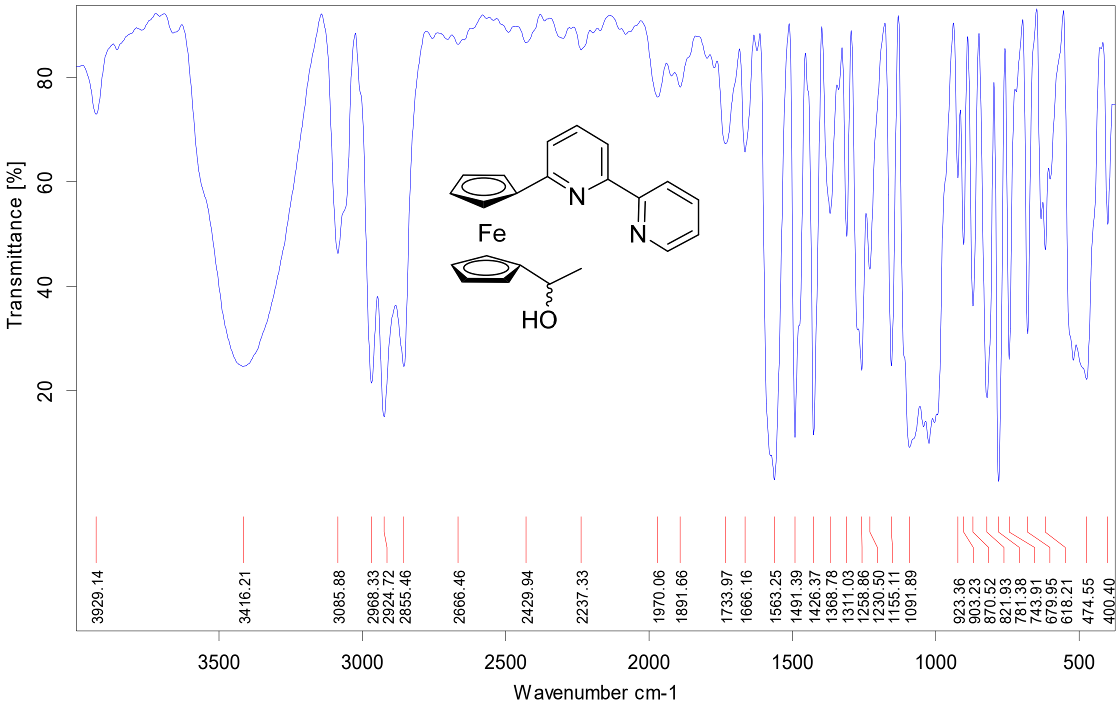

Figure S4.7. IR spectrum for 1-(2,2'-bipyridin-6-yl)-1'-(α-hydroxyethyl)ferrocene **(6d)**.

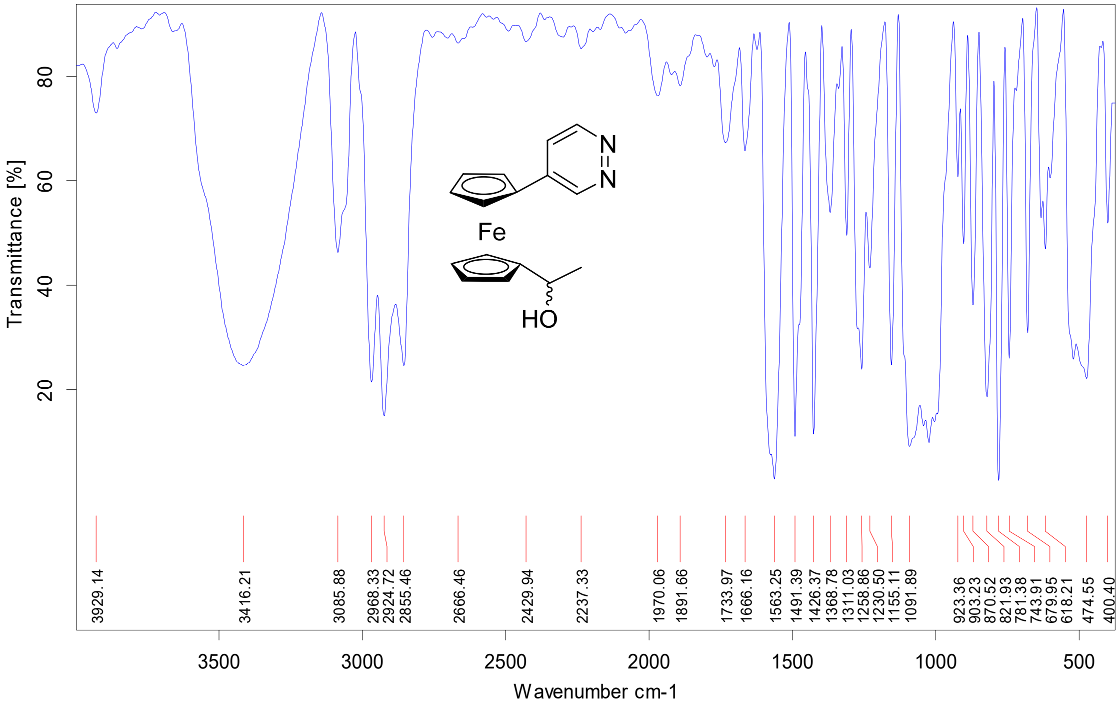

Figure S4.8. IR spectrum for 1-(pyridazin-4-yl)-1'-(α-hydroxyethyl)ferrocene **(6e)**.

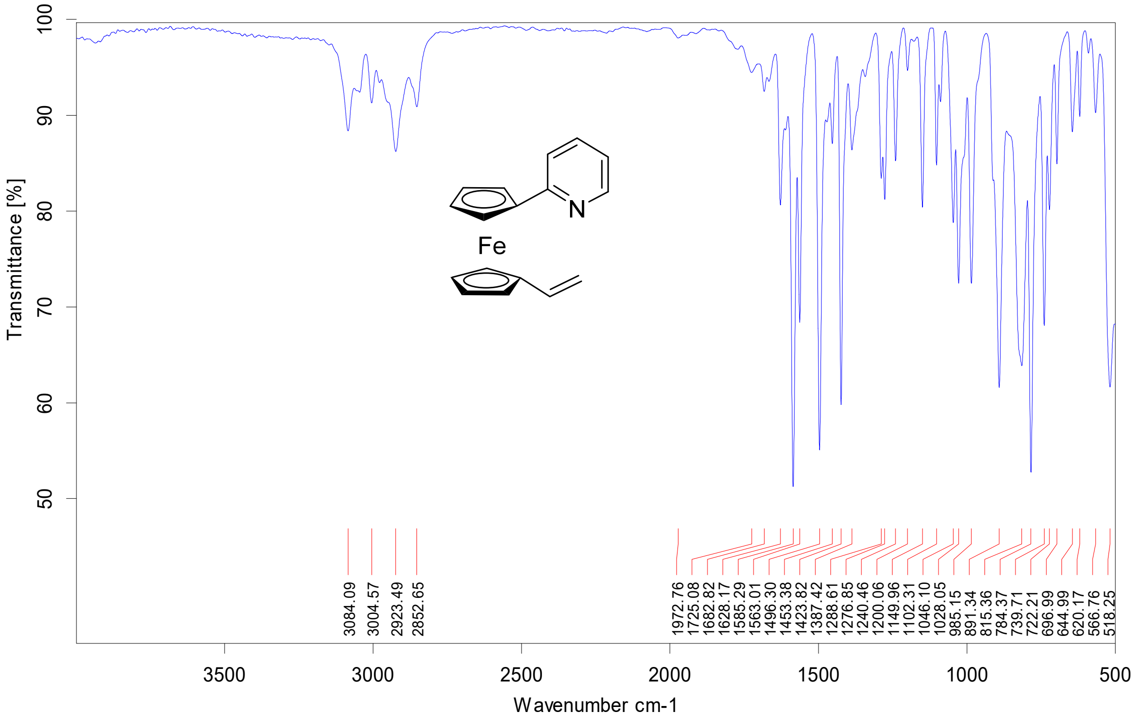

Figure S4.9. IR spectrum for 1-(pyridin-2-yl)-1'-vinylferrocene **(7a)**.

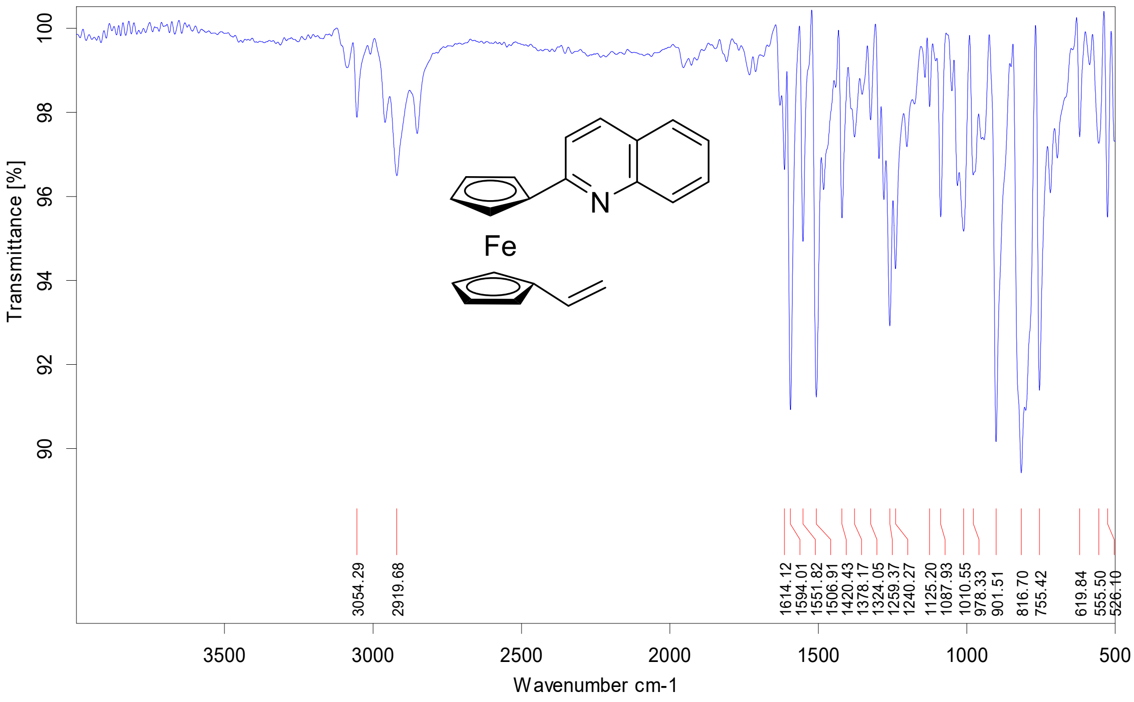

Figure S4.10. IR spectrum for 1-(quinolin-2-yl)-1'-vinylferrocene **(7b)**.

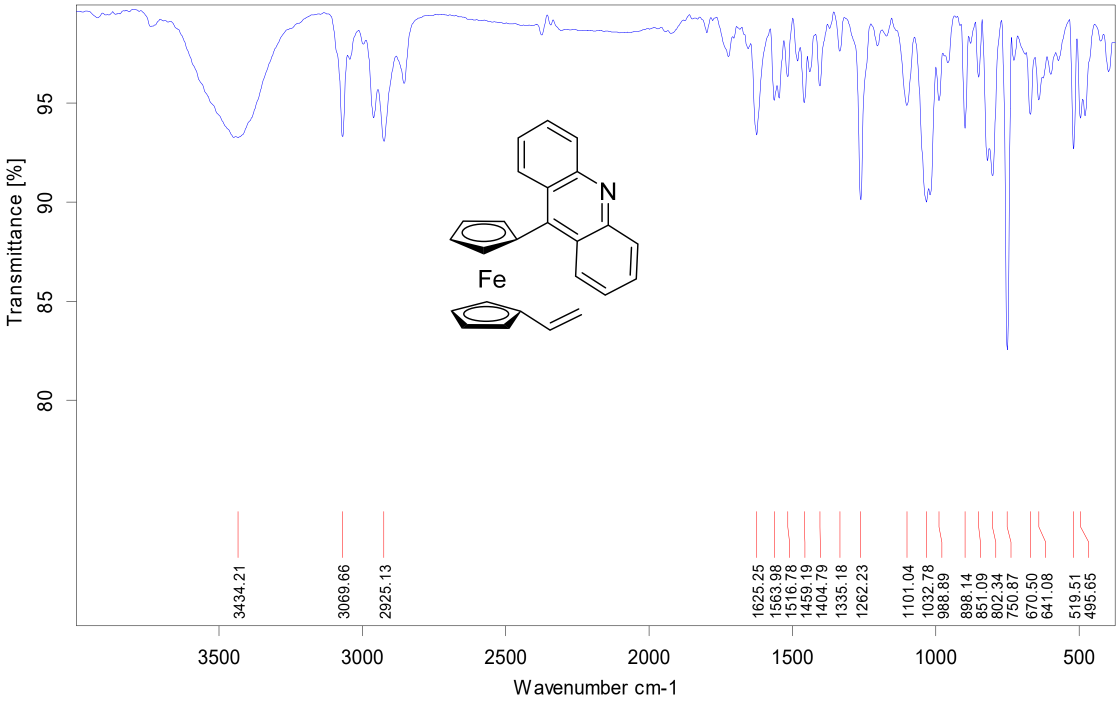

Figure S4.11. IR spectrum for 1-(acridin-9-yl)-1'-vinylferrocene **(7c)**.

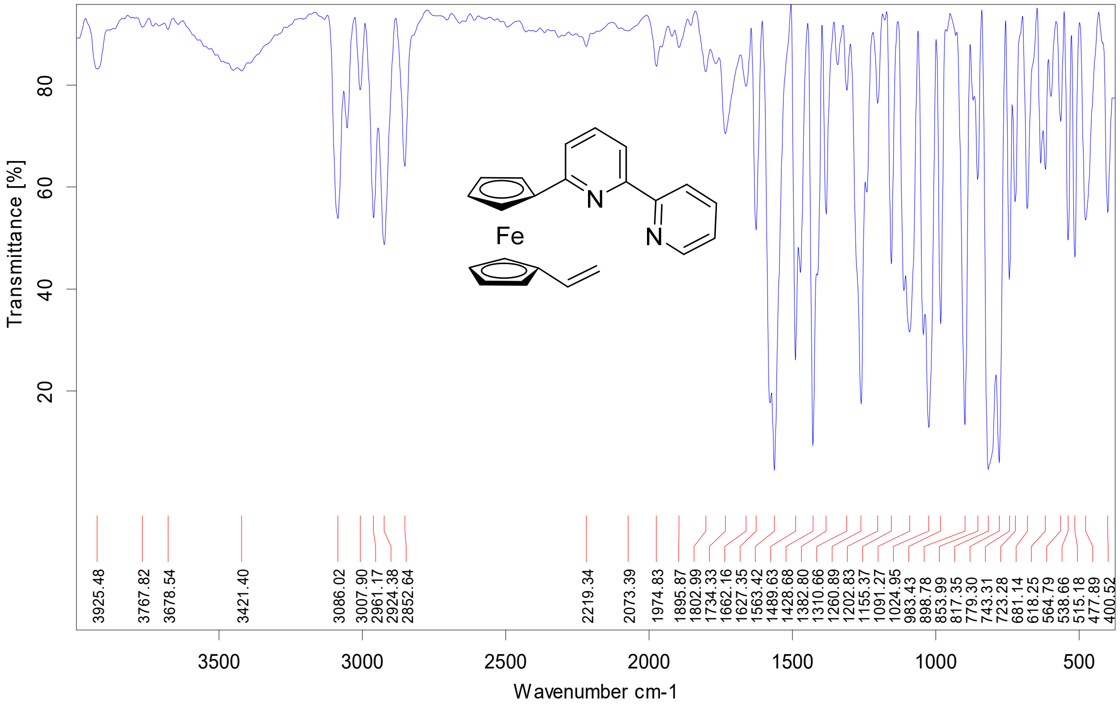

Figure S4.12. IR spectrum for 1-(2,2'-bipyridin-6-yl)-1'-vinylferrocene **(7d)**.

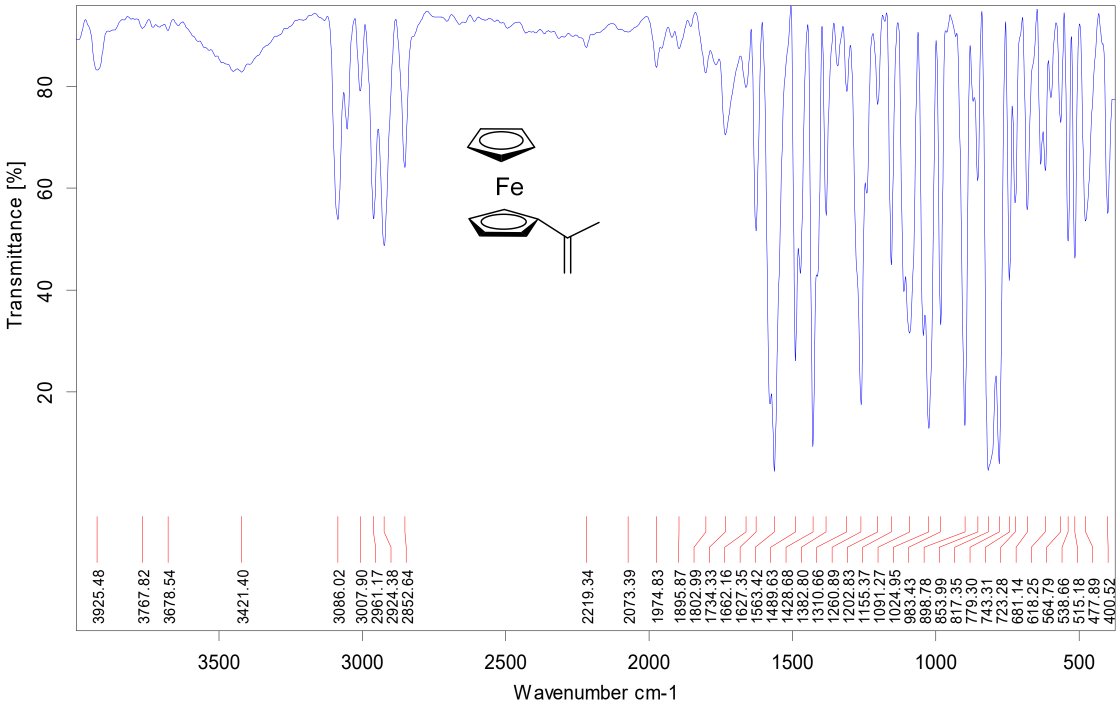

Figure S4.13. IR spectrum for isopropenylferrocene **(8)**.

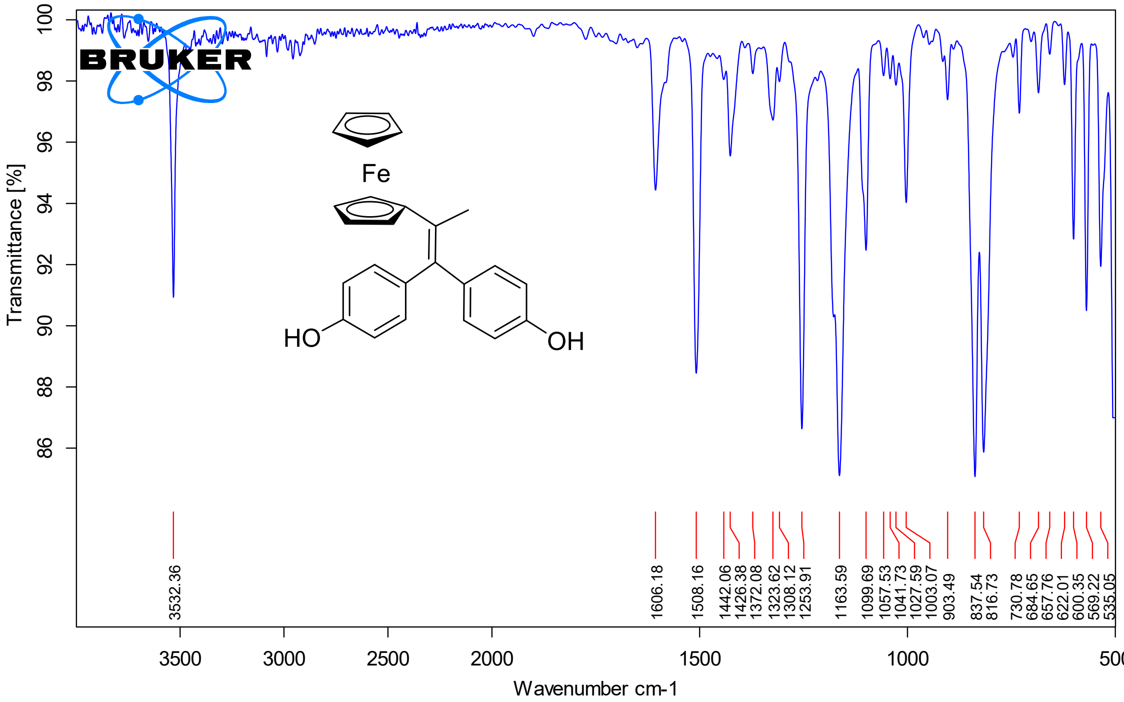

Figure S4.14. IR spectrum for 1,1-bis(4-hydroxyphenyl)-2-ferrocenylprop-1-ene **(10)**.

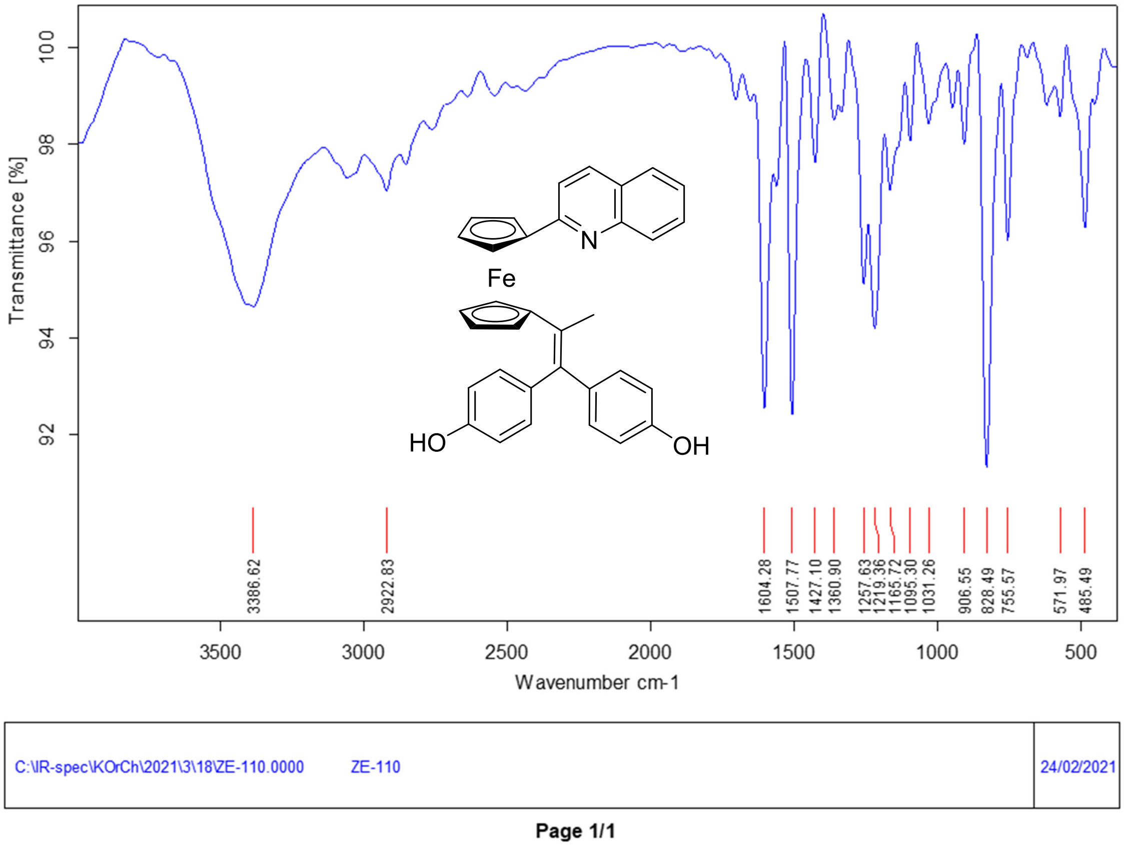

Figure S4.15. IR spectrum for 1-(quinolin-2-yl)-1´-{1-[bis(4-hydroxyphenyl)methyl-ene]ethyl}ferrocene **(11)**.

Section S5. Protonation state of the studied compounds under experimental conditions

Given that ferrocenes are metal complexes, conventional empirical programs such as Marvin or MolGpKa cannot be used to estimate pKa. To understand the protonation state under experimental conditions, the proton affinity (PA) values of the studied Fc compounds were calculated and compared with those of the ligands (pyridine, quinolone and acridine), the experimental pKa values of which are known.

Table S1. Calculated PA values in water and experimental pKa values of pyridine, quinolone and acridine

compound	PA, kcal/mol	Experimental pKa
Pyridine	25.1	5.23 [139]
Quinoline	25.7	4.85 [140]
Acridine	27.9	5.58 [141]
Bipyridine	27.4	4.35 [142]
Pyridazine	20.9	2.10 [143]
**1a**	27.4	n/a
**1b**	28.3	n/a
**1c**	29.2	n/a
**1d**	29.9	n/a
**1e**	24.4	n/a
**5a**	27.9	n/a
**5b**	29.5	n/a
**5c**	30.5	n/a
**7a**	27.7	n/a
**7b**	28.9	n/a
**7c**	29.5	n/a

Although there is no direct correlation between the PA and pKa values (as seen in the series pyridazine, pyridine, bipyridine, quinoline, acridine), the increase in the PA value of ferrocene derivatives with heterocyclic aromatic groups compared to the PA values of the heterocyclic aromatic groups themselves by an average of 2 kcal/mol indicates more basic properties of ferrocene derivatives than those of the heterocyclic aromatic groups themselves (see Table S1).

Calculations of pKa values require high accuracy time consuming quantum-chemical calculations of Gibbs free energy change in the thermodynamic cycle of the acid dissociation reaction [144], which are beyond the scope of this work. However, it can be assumed that the pKa values of ferrocene derivatives are higher than those of the corresponding heterocyclic aromatic compounds.

The pKa value is proportional to the Gibbs energy change, ΔG, in the protonation reaction (or the acid dissociation reaction):

$$pKa = \frac{\Delta \mathrm{G}}{(\ln 10)RT},$$

where *R* and *T* denote the gas constant and absolute temperature, respectively.

If ΔG values of ferrocene derivatives are also approximately 2 kcal/mol higher than those of the corresponding heterocyclic aromatic compounds, then pKa values of their ferrocene derivatives with pyridine, bipyridine, quinolone and acridine ligands seems to be about 7 or higher. This means that in AChE/BChE inhibition experiments, these ferrocene derivatives are partially or fully protonated.

As for the pyridazine ligand compounds, they appear to be neutral in AChE/BChE inhibition experiments, despite their PA being approximately 3.5 kcal/mol higher than that of pyridazine itself. We

assume this because pyridazine with pKa=2.1 has PA = 20.9 kcal/mol, and compound **1e** with pyridazine ligand has PA = 24.4 kcal/mol, which is lower than the PA values of pyridine, bipyridine, quinolone and acridine ligands with pKa values below 6.

All of the above allows us to assume that in experiments on the inhibition of AChE/BChE, ferrocene derivatives with pyridine, bipyridine, quinolone and acridine ligands are fully or partially protonated, while ferrocene derivatives with pyridazine ligand are neutral.

These protonation states of the studied compounds are consistent with the experimental anti-AChE/BChE activity, where protonated compounds demonstrate high activity and neutral compounds demonstrate low activity.

Taking into account all the above, compounds **1a**, **1b**, **1c**, **5a**, **5b**, **5c**, **7a**, **7b**, and **7c** are clearly protonated under the FRAP test conditions (water, acetate buffer, pH=3.6).

In the ABTS test, the protonation state is controlled by the favorability of proton transfer from NH_4^+ cation to the compound under study. The point is that in the ABTS test the acidity of the medium (pH ≈ 5) is the result of dissolving 2,2'-Azinobis(3-ethylbenzothiazoline-6-sulfonic acid) diammonium salt in the solvent (90% ethanol, 8% water, 2% DMSO), and is not maintained by any buffer. To estimate the protonation state, the PA values of ammonia and the studied compounds were calculated in ethanol (see Table S2).

Table S2. Calculated PA values in ethanol (ABTS test conditions).

compound	PA, kcal/mol
NH_3	24.3
**1a**	24.1
**1b**	25.0
**1c**	26.3
**5a**	24.5
**5b**	25.7
**5c**	26.3
**7a**	24.7
**7b**	26.1
**7c**	27.0

The calculated PA values of compounds with pyridine substituents are close the PA value of NH_3. This means that some of the protons are transferred from NH_4^+ cations to the compounds under study, i.e. the compounds with pyridine ligands are partially protonated. The calculated PA values of compounds with quinoline and acridine substituents are higher than that of NH_3. This means that compounds with quinoline and acridine substituents are protonated under the ABTS test conditions.

Section S6. Dimerization of compounds $1a_p$, $1b_p$, and $1c_p$

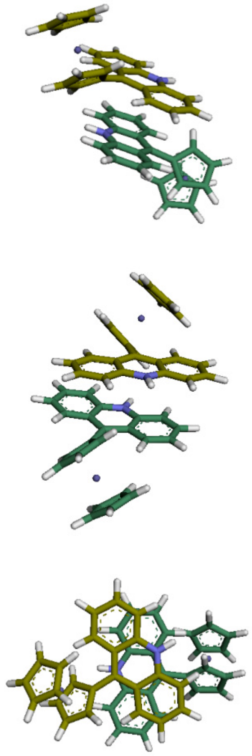

a b c

Figure S6.1. Dimers in water: compounds **1a**$_p$ (a), **1b**$_p$ (b), **1c**$_p$ (c) from different points of view. Carbon atoms of one molecule are green, carbon atoms of the other molecule are marsh green.

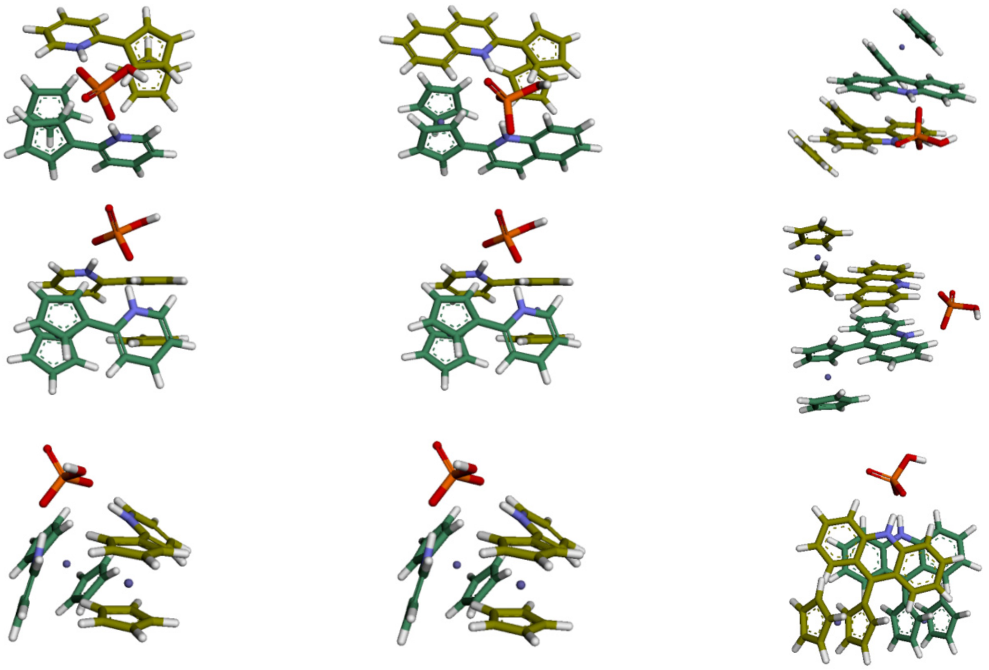

a c c

Figure S6.2. Dimers in water stabilized by HPO_4^{2-} anion: compounds **1a**$_p$ (a), **1b**$_p$ (b), **1c**$_p$ (c) from different points of view. Carbon atoms of one molecule are green, carbon atoms of the other molecule are marsh green.

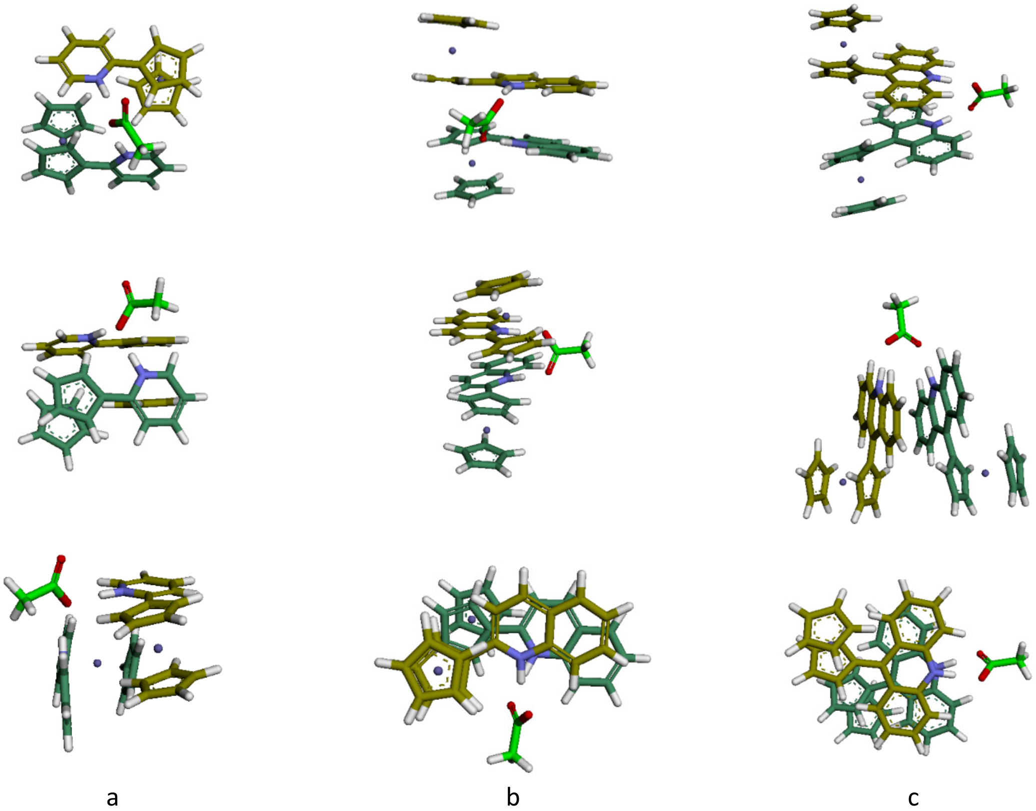

Figure S6.3 Dimers in water stabilized by CH_3COO^- anion: compounds **1a**$_p$ (a), **1b**$_p$ (b), **1c**$_p$ (c) from different points of view. Carbon atoms of one molecule are green, carbon atoms of the other molecule are marsh green. Carbon atoms of CH_3COO^- anion are bright green.

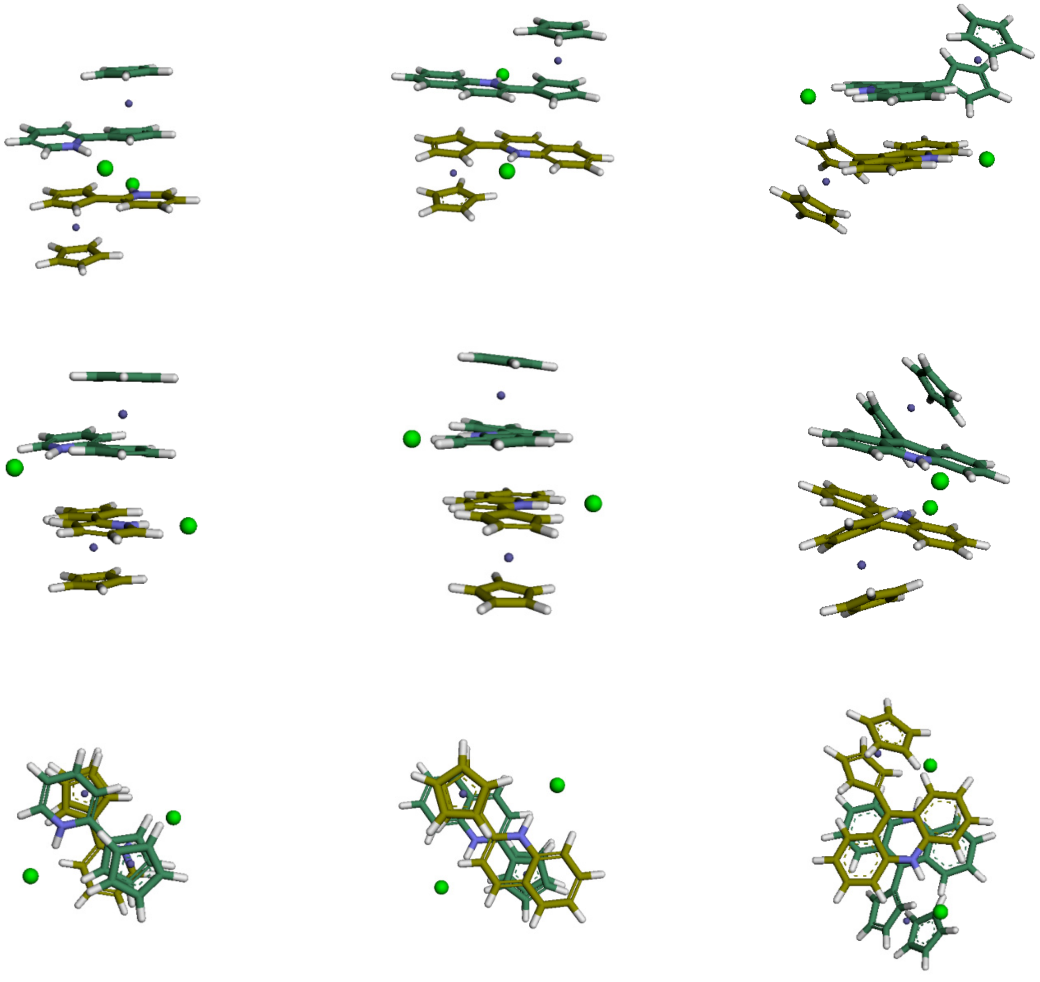

a c c

Figure S6.4 Dimers in water stabilized by two Cl^- anions (Tris-HCl buffer, pH=8.0): compounds $\mathbf{1a}_p$ (a), $\mathbf{1b}_p$ (b), $\mathbf{1c}_p$ (c) from different points of view. Carbon atoms of one molecule are green, carbon atoms of the other molecule are marsh green. Chloride anions are bright green.

Table S3. Enthalpy of dimers formation (kcal/mol)

	Dimer in water	Dimer in water stabilized by HPO_4^{2-} anion (phosphate buffer, pH=7.4)	Dimer in water stabilized by CH_3COO^- anion (acetate buffer, pH =3.6)	Dimer in water stabilized by Cl^- anion (Tris-HCl buffer, pH=8.0)	Dimer in ethanol stabilized by SO_4^{2-} anion
pyridine	-3.3	n/c	n/c	n/c	n/c
quinolone	-6.9	n/c	n/c	n/c	n/c
acridine	-10.5	n/c	n/c	n/c	n/c
pyridine H^+	-3.7	n/c	n/c	n/c	n/c
quinolone H^+	-7.8	n/c	n/c	n/c	n/c
acridine H^+	-11.9	n/c	n/c	n/c	n/c
**1a**$_p$	-10.4	-34.8	-29.0	-10.9	-36.4
**1b**$_p$	-12.6	-36.8	-30.2	-14.6	-38.1
**1c**$_p$	-17.1	-39.2	-33.2	-18.3	-40.3

n/c – not calculated

Apparently, the aggregation of the studied compounds should influence their inhibitory activity of cholinesterases and self-aggregation of β-amyloid as well as their AOA. It is known that acridine based derivatives reveal strong tendency for aggregation both in the crystalline state [145] and in water solution [83]. So, we investigated the aggregation ability of studied compound with heterocyclic substituents under experimental conditions (in water and ethanol).

Ferrocenes can dimerize via stacking formation between aromatic fragments. Aromatic fragments can form different stacking types: π-π stacking, parallel-slipped stacking, T-shape stacking [146-148]. It was shown that water molecules can form bridges between dimer molecules and thereby stabilize dimers [149,150]. Protonation changes the electron density distribution in the aromatic fragment and can thus affect dimer stability [151].

The calculated dimer structures of **1a**$_p$, **1b**$_p$ and **1c**$_p$ in pure water are shown Fig.S6.1. Dimers of **1a**$_p$, **1b**$_p$ are stabilized via T-shape stacking between aromatic heterocyclic fragment of one molecules and cyclopentadienyl of the other molecule. While the **1c**$_p$ dimer is stabilized via π-π stacking of the aromatic heterocyclic fragments of both molecules.

The enthalpies of dimers formation in water are given in Table S3. For comparison, the enthalpies of π-π stacked dimers formation for pyridine, quinoline and acridine in neutral and protonated forms are also presented. It can be seen that the advantage of dimer formation increases with increasing the aromatic system size. Also, protonation increases the advantage of dimer formation despite the Coulomb repulsion of similarly charged molecules.

It can be reasonably assumed that the dimerization of positively charged protonated compounds should be stabilized by buffer anions (HPO_4^{2-} and H_2PO4^- in phosphate buffer, pH = 7.4, or CH_3COO^- in acetate buffer, pH =3.6; Cl^- anions in Tris-HCl buffer, pH = 8.0) due to additional Coulomb interaction. The most stable dimers of protonated compounds **1a**$_p$, **1b**$_p$ and **1c**$_p$, calculated in water without (simple dimer) and with additional stabilizing anions HPO_4^{2-}, CH_3COO^- and Cl^- are shown in Figs. S6.1, S6.2, S6.3 and S6.4, respectively. The enthalpies of their formation are given in Table S3.

The increase of the dimerization advantage for compounds **1a**$_p$, **1b**$_p$, **1c**$_p$ compared to pyridine H^+, quinoline H^+, acridine H^+ is resulted from the increase in the aromatic system size due to the cyclopentadienyl of the ferrocene group. The second cyclopentadienyl allows the formation of T-shape stacking for compounds **1a**$_p$, **1b**$_p$, **1c**$_p$.

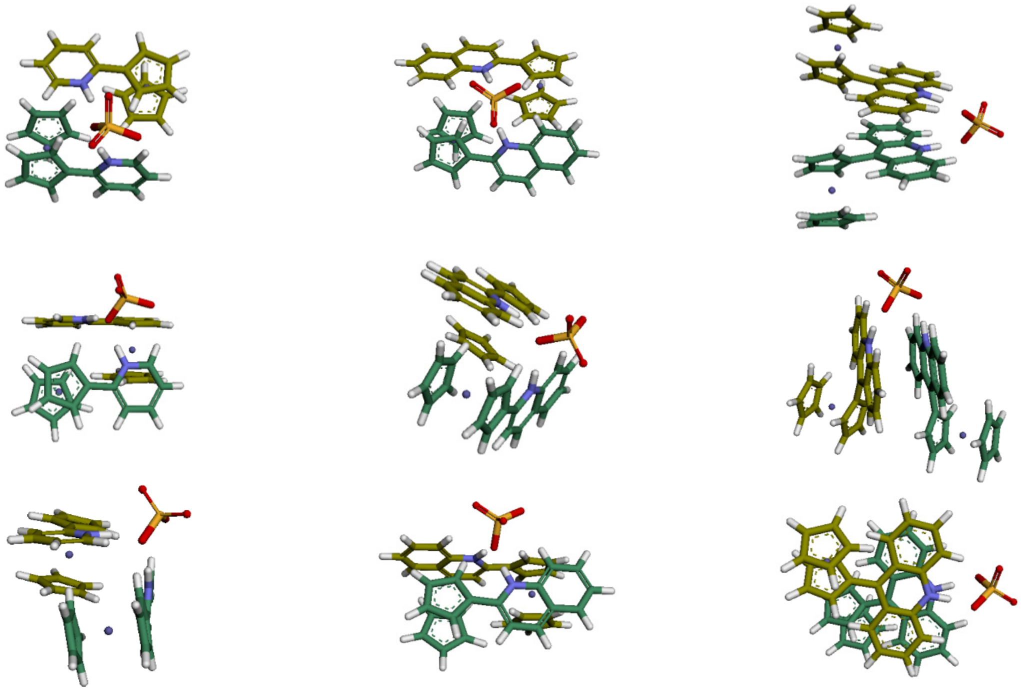

a c c

Figure S6.5. Dimers in ethanol stabilized by SO_4^{2-} anion: compounds **1a**$_p$ (a), **1b**$_p$ (b), **1c**$_p$ (c) from different points of view. Carbon atoms of one molecule are green, carbon atoms of the other molecule are marsh green.

Similar tendencies were observed for dimer formation in ethanol, where dimers were stabilized via sulfate anion SO_4^{2-} formed under $ABTS^{\bullet+}$ pre-generation [152]. The most stable dimers of protonated compounds **1a**$_p$, **1b**$_p$ and **1c**$_p$ stabilized by SO_4^{2-} in ethanol are shown in Figure S6.5. The corresponding enthalpies of dimer formation are given in Table S3.

Thus, quantum-chemical calculations have shown (see Table S3) that the advantage of dimer formation growths with increasing size of the aromatic heterocyclic ligand. At the same time, buffer anions increase the advantage of dimer formation for protonated compounds.

Section S7. Heatmaps of percent overlap of docked ligands in *Ec*BChE, *Ss*CES1, *Ee*AChE, and *Hs*Aβ_{42}

Monomers

ID	1a	1b	1c	1d	1e	1o	5a	5b	5c	5d	5e	5o	7a	7b	7c	7d	7o	don	myr	prm	tac
1a	100.0	59.6	42.4	23.5	75.4	40.1	56.4	28.4	38.2	33.7	41.7	75.6	70.0	37.2	36.6	26.7	73.6	24.4	29.8	28.2	40.6
1b	59.6	100.0	44.8	21.0	61.5	33.8	38.8	25.3	40.5	25.8	37.7	47.4	52.4	33.2	38.7	22.4	47.3	20.9	31.0	31.9	51.9
1c	42.4	44.8	100.0	21.6	43.0	25.8	47.4	27.8	80.2	37.0	36.8	35.9	45.7	39.2	65.5	27.8	33.1	42.3	40.6	37.9	44.9
1d	23.5	21.0	21.6	100.0	26.7	19.7	21.8	44.4	20.8	34.1	28.1	24.3	22.9	24.0	18.9	33.6	20.8	35.7	18.3	36.2	19.9
1e	75.4	61.5	43.0	26.7	100.0	37.7	47.3	27.8	38.2	31.8	43.4	71.1	66.1	36.7	35.4	26.1	66.3	22.3	31.7	29.3	44.2
1o	40.1	33.8	25.8	19.7	37.7	100.0	24.5	24.8	23.6	20.7	31.7	39.4	33.0	27.7	24.0	21.2	40.2	16.5	17.4	21.6	34.7
5a	56.4	38.8	47.4	21.8	47.3	24.5	100.0	25.7	42.3	47.6	35.1	49.8	63.3	45.8	37.9	25.8	43.4	29.4	30.4	34.4	27.9
5b	28.4	25.3	27.8	44.4	27.8	24.8	25.7	100.0	25.3	36.7	39.3	29.3	26.2	25.2	22.9	57.5	29.3	43.5	14.7	42.1	21.3
5c	38.2	40.5	80.2	20.8	38.2	23.6	42.3	25.3	100.0	33.8	32.6	32.1	40.8	35.8	73.1	26.5	29.3	43.3	38.3	35.5	41.7
5d	33.7	25.8	37.0	34.1	31.8	20.7	47.6	36.7	33.8	100.0	28.7	31.8	40.6	39.3	29.4	42.2	28.5	41.9	23.6	44.4	19.5
5e	41.7	37.7	36.8	28.1	43.4	31.7	35.1	39.3	32.6	28.7	100.0	40.8	37.5	31.6	31.0	35.8	40.9	22.2	22.3	31.7	31.9
5o	75.6	47.4	35.9	24.3	71.1	39.4	49.8	29.3	32.1	31.8	40.8	100.0	64.5	34.7	30.2	27.0	75.4	20.7	25.9	24.4	36.5
7a	70.0	52.4	45.7	22.9	66.1	33.0	63.3	26.2	40.8	40.6	37.5	64.5	100.0	43.6	36.0	27.6	54.9	24.7	31.0	33.1	34.0
7b	37.2	33.2	39.2	24.0	36.7	27.7	45.8	25.2	35.8	39.3	31.6	34.7	43.6	100.0	32.7	23.5	31.9	24.4	23.9	36.2	26.4
7c	36.6	38.7	65.5	18.9	35.4	24.0	37.9	22.9	73.1	29.4	31.0	30.2	36.0	32.7	100.0	21.8	26.9	39.8	43.9	32.6	42.7
7d	26.7	22.4	27.8	33.6	26.1	21.2	25.8	57.5	26.5	42.2	35.8	27.0	27.6	23.5	21.8	100.0	28.1	44.1	10.9	38.9	17.4
7o	73.6	47.3	33.1	20.8	66.3	40.2	43.4	29.3	29.3	28.5	40.9	75.4	54.9	31.9	26.9	28.1	100.0	18.9	20.2	21.8	32.3
don	24.4	20.9	42.3	35.7	22.3	16.5	29.4	43.5	43.3	41.9	22.2	20.7	24.7	24.4	39.8	44.1	18.9	100.0	29.3	38.2	26.8
myr	29.8	31.0	40.6	18.3	31.7	17.4	30.4	14.7	38.3	23.6	22.3	25.9	31.0	23.9	43.9	10.9	20.2	29.3	100.0	17.9	35.6
prm	28.2	31.9	37.9	36.2	29.3	21.6	34.4	42.1	35.5	44.4	31.7	24.4	33.1	36.2	32.6	38.9	21.8	38.2	17.9	100.0	23.6
tac	40.6	51.9	44.9	19.9	44.2	34.7	27.9	21.3	41.7	19.5	31.9	36.5	34.0	26.4	42.7	17.4	32.3	26.8	35.6	23.6	100.0

(Row axis label: Monomers)

Figure S7.1. Heatmap of percent overlap of docked monomer ligands in *Ec*BChE.

Monomers

ID	1a	1b	1c	1d	1e	Fc	5a	5b	5c	5d	5e	8	7a	7b	7c	7d	9	don	myr	prm	tac
1a.2	0.0	0.0	1.2	0.3	0.0	0.0	1.5	0.1	4.3	2.4	0.0	0.0	1.1	5.7	4.0	1.7	0.0	1.8	4.0	1.7	0.0
1b.2	0.0	0.0	0.8	0.0	0.0	0.0	1.1	0.0	1.2	0.8	0.0	0.0	0.4	4.5	0.6	0.5	0.0	0.4	0.3	1.2	0.0
1c.2	5.6	4.2	12.6	17.9	4.4	3.7	12.7	21.0	17.6	22.2	7.9	5.2	8.8	16.3	15.3	21.3	5.2	24.3	11.0	23.7	1.8
1d.1	24.1	21.5	21.5	96.5	27.0	20.8	22.1	45.0	21.0	34.0	29.0	25.0	23.2	24.5	19.4	34.2	21.5	35.6	18.5	35.5	20.2
1e.1	76.0	62.2	44.1	26.4	97.3	37.4	47.5	27.8	39.6	32.0	44.1	71.0	67.5	36.6	36.8	26.4	66.3	22.6	32.8	29.6	44.4
Fc.1	40.5	33.2	25.3	19.2	37.5	97.6	25.3	24.8	23.2	21.0	31.5	39.2	33.3	27.2	23.5	21.2	40.0	16.2	17.0	21.6	34.0
5a.2	0.7	0.0	7.3	1.7	0.3	0.3	5.6	2.5	11.4	5.9	2.0	0.7	2.5	8.9	10.4	5.2	0.6	7.6	9.1	4.1	0.3
5b.2	0.0	0.0	0.1	1.1	0.0	0.0	0.0	0.6	3.2	0.9	0.0	0.0	0.0	1.1	2.2	0.7	0.0	2.4	2.6	3.1	0.0
5c.2	0.0	0.0	0.0	0.0	0.0	0.0	0.0	0.0	0.0	0.0	0.0	0.0	0.0	0.0	0.5	0.0	0.0	0.0	0.2	0.0	0.0
5d.1	34.2	26.2	37.0	33.2	32.6	19.8	48.4	36.7	34.1	93.7	28.5	31.9	40.7	39.7	30.4	42.6	28.2	43.3	23.8	44.2	19.1
5e.1	41.2	37.3	36.8	28.1	42.9	31.7	35.1	39.3	32.6	28.7	99.1	40.3	37.1	31.6	31.0	35.4	40.4	22.2	22.3	31.7	31.9
8.1	75.1	47.0	35.1	24.0	70.6	38.9	49.4	29.0	31.4	31.5	39.9	98.2	63.4	34.9	29.6	26.7	74.9	20.5	25.2	24.1	36.1
7a.2	0.0	0.0	2.8	0.3	0.0	0.0	1.7	0.1	6.0	2.3	0.0	0.0	0.7	6.3	5.8	1.3	0.0	2.5	6.8	1.7	0.0
7b.2	0.0	0.0	0.0	0.4	0.0	0.0	0.0	0.4	2.2	0.6	0.0	0.0	0.0	1.1	1.4	0.6	0.0	1.3	2.7	2.5	0.0
7c.2	0.0	0.0	0.0	0.0	0.0	0.0	0.0	0.0	1.4	0.0	0.0	0.0	0.0	0.0	1.8	0.0	0.0	0.1	0.6	0.0	0.0
7d.1	27.2	24.0	23.3	62.2	29.5	22.5	21.0	53.8	24.0	31.8	36.4	28.7	25.8	25.4	21.1	46.7	27.5	37.1	13.4	37.2	21.7
9.1	75.5	45.3	32.9	19.6	64.9	39.8	44.2	28.7	29.8	28.7	40.1	76.5	57.7	32.6	27.5	28.7	89.8	18.1	20.1	20.9	31.5
don.1	23.8	20.4	41.5	36.0	21.7	16.0	28.8	42.0	42.0	39.9	22.0	20.2	23.6	22.9	38.8	43.6	18.4	93.7	29.0	37.3	25.1
myr.1	30.5	30.9	40.5	17.9	32.0	17.0	31.0	14.7	38.2	23.6	22.9	26.2	30.9	23.5	44.1	10.9	20.9	29.2	97.9	17.6	35.5
prm.1	27.4	31.3	37.1	35.4	28.7	20.8	34.2	41.5	34.7	43.6	30.9	23.8	32.6	35.7	31.6	38.0	21.3	37.4	17.2	97.5	22.8
tac.1	40.7	51.0	44.1	19.6	45.0	33.6	28.0	20.7	41.0	19.0	31.6	36.7	34.2	25.7	41.9	16.8	33.1	26.5	35.7	23.1	97.0

(Row axis label: Monomers and Dimers)

Figure S7.2. Mixed heatmap of percent overlap of docked monomer and dimer ligands in *Ec*BChE.

Monomers (columns) × Monomers (rows)

ID	1a	1b	1c	1d	1e	1o	5a	5b	5c	5d	5e	5o	7a	7b	7c	7d	7o	don	myr	prm	tac
1a	100.0	31.4	56.9	72.3	63.8	76.5	50.0	48.1	30.2	46.4	62.5	59.1	60.0	31.4	30.2	55.6	63.4	42.3	36.2	23.5	47.8
1b	31.4	100.0	25.4	25.0	18.8	25.5	26.2	33.9	83.8	28.6	35.1	21.1	29.1	100.0	83.8	31.7	22.2	62.2	20.8	66.0	21.8
1c	56.9	25.4	100.0	69.1	58.9	41.2	50.0	43.8	24.6	46.9	45.2	51.9	42.4	25.4	24.6	47.7	38.6	34.4	58.2	20.0	60.0
1d	72.3	25.0	69.1	100.0	87.5	55.3	65.5	57.6	24.2	61.0	68.5	69.4	60.4	25.0	24.2	70.2	63.3	33.8	55.1	19.8	72.3
1e	63.8	18.8	58.9	87.5	100.0	60.5	70.6	61.8	18.2	65.5	58.2	75.6	62.0	18.8	18.2	63.2	65.2	27.7	49.3	14.8	63.8
1o	76.5	25.5	41.2	55.3	60.5	100.0	48.9	46.9	24.5	45.1	59.1	59.0	60.0	25.5	24.5	52.0	78.8	37.5	24.6	18.8	30.4
5a	50.0	26.2	50.0	65.5	70.6	48.9	100.0	87.5	25.4	91.7	60.0	73.9	64.0	26.2	25.4	77.4	54.0	44.8	40.5	27.0	52.9
5b	48.1	33.9	43.8	57.6	61.8	46.9	87.5	100.0	32.8	80.8	52.5	70.8	61.5	33.9	32.8	68.4	51.9	53.6	35.9	33.3	45.5
5c	30.2	83.8	24.6	24.2	18.2	24.5	25.4	32.8	100.0	27.7	33.9	20.3	28.1	83.8	100.0	34.9	21.4	59.6	20.3	70.0	21.1
5d	46.4	28.6	46.9	61.0	65.5	45.1	91.7	80.8	27.7	100.0	55.9	68.0	59.3	28.6	27.7	78.2	50.0	51.7	38.5	32.4	49.1
5e	62.5	35.1	45.2	68.5	58.2	59.1	60.0	52.5	33.9	55.9	100.0	56.9	74.5	35.1	33.9	74.1	75.0	44.8	40.5	27.0	44.4
5o	59.1	21.1	51.9	69.4	75.6	59.0	73.9	70.8	20.3	68.0	56.9	100.0	68.2	21.1	20.3	56.4	72.5	31.0	45.5	16.2	55.6
7a	60.0	29.1	42.4	60.4	62.0	60.0	64.0	61.5	28.1	59.3	74.5	68.2	100.0	29.1	28.1	57.1	77.5	39.3	38.0	22.2	41.2
7b	31.4	100.0	25.4	25.0	18.8	25.5	26.2	33.9	83.8	28.6	35.1	21.1	29.1	100.0	83.8	31.7	22.2	62.2	20.8	66.0	21.8
7c	30.2	83.8	24.6	24.2	18.2	24.5	25.4	32.8	100.0	27.7	33.9	20.3	28.1	83.8	100.0	34.9	21.4	59.6	20.3	70.0	21.1
7d	55.6	31.7	47.7	70.2	63.2	52.0	77.4	68.4	34.9	78.2	74.1	56.4	57.1	31.7	34.9	100.0	56.6	55.2	39.2	35.1	50.0
7o	63.4	22.2	38.6	63.3	65.2	78.8	54.0	51.9	21.4	50.0	75.0	72.5	77.5	22.2	21.4	56.6	100.0	32.7	34.8	16.9	36.7
don	42.3	62.2	34.4	33.8	27.7	37.5	44.8	53.6	59.6	51.7	44.8	31.0	39.3	62.2	59.6	55.2	32.7	100.0	22.0	60.7	32.1
myr	36.2	20.8	58.2	55.1	49.3	24.6	40.5	35.9	20.3	38.5	40.5	45.5	38.0	20.8	20.3	39.2	34.8	22.0	100.0	17.0	56.7
prm	23.5	66.0	20.0	19.8	14.8	18.8	27.0	33.3	70.0	32.4	27.0	16.2	22.2	66.0	70.0	35.1	16.9	60.7	17.0	100.0	16.7
tac	47.8	21.8	60.0	72.3	63.8	30.4	52.9	45.5	21.1	49.1	44.4	55.6	41.2	21.8	21.1	50.0	36.7	32.1	56.7	16.7	100.0

Figure S7.3. Heatmap of percent overlap of docked monomer ligands in *Ee*AChE.

Monomers (columns) × Monomers and Dimers (rows)

ID	1a	1b	1c	1d	1e	Fc	5a	5b	5c	5d	5e	8	7a	7b	7c	7d	9	don	myr	prm	tac
1a.2	0.0	22.2	0.0	0.0	0.0	0.0	0.8	5.6	27.8	7.0	0.0	0.0	0.0	24.1	29.2	6.7	0.0	18.0	0.0	29.3	0.0
1b.2	0.0	8.8	0.0	0.0	0.0	0.0	0.0	0.5	19.7	0.1	0.0	0.0	0.0	10.7	19.3	0.1	0.0	7.1	0.0	14.7	0.0
1c.2	0.0	15.9	0.0	0.0	0.0	0.0	0.4	3.8	21.6	5.2	0.0	0.0	0.0	17.3	22.0	5.0	0.0	12.4	0.0	21.1	0.0
1d.1	54.6	1.7	58.3	97.7	63.2	38.8	34.5	27.7	1.1	30.3	39.1	40.4	32.1	1.6	1.1	31.6	40.6	18.2	46.3	0.2	50.7
1e.1	43.9	0.0	49.5	64.4	98.9	52.4	38.8	31.0	0.0	32.5	43.1	49.8	36.1	0.0	0.0	33.0	55.3	17.9	31.9	0.0	34.0
Fc.1	55.7	0.0	33.2	37.5	53.4	84.7	41.0	36.2	0.0	38.7	47.6	68.2	38.4	0.0	0.0	42.4	78.1	21.3	19.9	0.0	23.9
5a.2	0.0	23.3	0.0	0.0	0.0	0.0	3.6	7.3	28.9	9.1	0.4	0.0	1.7	24.5	29.6	8.3	0.0	19.0	0.0	28.0	0.0
5b.2	0.0	4.8	0.0	0.0	0.0	0.0	0.0	0.1	12.1	0.0	0.0	0.0	0.0	6.7	12.2	0.0	0.0	4.0	0.0	11.7	0.0
5c.2	0.0	17.6	0.0	0.0	0.0	0.0	3.1	6.6	22.3	7.4	0.2	0.0	1.4	19.1	22.4	6.9	0.0	14.1	0.0	21.0	0.0
5d.1	35.4	20.1	27.1	29.8	32.7	36.5	53.3	61.7	15.3	96.6	46.8	53.2	49.9	19.4	16.3	68.0	41.8	45.3	19.0	13.8	21.4
5e.1	43.3	6.6	38.2	37.9	42.3	47.6	45.4	44.2	5.3	46.2	97.7	48.1	54.4	6.4	4.8	49.2	53.6	31.8	22.2	2.7	22.5
8.1	52.6	0.0	34.3	40.1	49.4	60.8	45.7	42.9	0.0	54.1	49.2	98.2	44.6	0.0	0.0	41.8	71.6	21.1	25.9	0.0	28.6
7a.2	0.0	24.0	0.0	0.0	0.0	0.0	2.6	6.5	28.5	8.3	0.3	0.0	1.3	25.7	29.5	8.1	0.0	19.3	0.0	28.6	0.0
7b.2	0.0	4.7	0.0	0.0	0.0	0.0	0.0	0.0	12.3	0.0	0.0	0.0	0.0	6.5	12.5	0.0	0.0	4.1	0.0	12.8	0.0
7c.2	0.0	18.4	0.0	0.0	0.0	0.0	2.0	5.2	24.8	7.2	0.3	0.0	1.1	20.4	24.8	6.9	0.0	14.7	0.0	22.5	0.0
7d.1	0.0	30.1	0.0	0.0	0.0	0.0	2.5	8.0	56.1	8.8	0.0	0.0	0.6	26.5	49.6	10.5	0.0	16.6	0.0	33.2	0.0
9.1	52.7	0.0	36.0	41.3	56.9	74.4	37.7	38.0	0.0	40.8	55.5	70.4	43.3	0.0	0.0	39.3	97.4	21.7	25.2	0.0	22.5
don.1	21.4	35.6	19.9	18.4	18.4	19.1	30.3	40.0	23.6	44.8	32.4	21.2	29.5	34.6	24.6	45.1	21.1	96.2	16.2	27.1	19.1
myr.1	32.9	1.1	47.1	46.4	32.4	18.8	19.7	17.5	0.8	19.0	22.5	25.9	17.5	1.0	0.8	18.0	25.1	16.1	97.9	0.2	51.3
prm.1	0.0	33.0	0.2	0.0	0.0	0.0	4.1	11.4	35.0	13.8	2.7	0.0	4.1	33.1	35.3	13.6	0.0	26.9	0.0	94.0	0.2
tac.1	48.3	1.0	46.0	52.0	35.8	20.1	27.1	20.5	0.4	20.9	22.3	28.3	19.8	1.0	0.4	21.4	22.5	18.3	51.9	0.0	97.6

Figure S7.4. Mixed heatmap of percent overlap of docked monomer and dimer ligands in *Ee*AChE.

Monomers and Dimers

ID	1a.2	1b.2	1c.2	1d.1	1e.1	Fc.1	5a.2	5b.2	5c.2	5d.1	5e.1	8.1	7a.2	7b.2	7c.2	7d.1	9.1	don.1	myr.1	prm.1	tac.1
1a.2	100.0	31.4	0.0	29.4	25.1	18.3	0.0	27.7	0.0	30.2	29.7	21.4	27.2	32.9	0.0	27.8	21.4	17.4	20.0	24.6	0.0
1b.2	31.4	100.0	0.0	31.2	16.4	12.6	0.5	65.1	0.0	28.2	20.6	17.1	61.8	82.2	0.0	29.0	13.2	19.5	25.2	30.4	0.0
1c.2	0.0	0.0	100.0	0.0	0.0	0.0	0.0	0.0	87.8	0.0	0.0	0.0	0.0	0.0	90.7	0.0	0.0	0.0	0.0	0.0	0.0
1d.1	29.4	31.2	0.0	100.0	30.7	30.8	0.5	27.1	0.0	53.6	33.4	34.1	29.1	32.7	0.0	81.0	32.9	30.9	47.9	37.1	0.0
1e.1	25.1	16.4	0.0	30.7	100.0	38.4	0.0	20.1	0.0	36.8	73.2	49.6	21.9	19.2	0.0	31.9	45.5	26.3	24.0	21.7	0.0
Fc.1	18.3	12.6	0.0	30.8	38.4	100.0	0.0	12.1	0.0	27.2	36.6	51.8	16.0	13.4	0.0	29.6	81.6	21.2	20.5	21.0	0.0
5a.2	0.0	0.5	0.0	0.5	0.0	0.0	100.0	1.0	0.0	1.9	0.0	0.0	0.0	0.5	0.0	0.4	0.0	12.4	0.0	1.5	23.9
5b.2	27.7	65.1	0.0	27.1	20.1	12.1	1.0	100.0	0.0	28.4	23.0	17.9	58.4	64.3	0.0	25.7	14.2	20.1	24.1	28.3	0.0
5c.2	0.0	0.0	87.8	0.0	0.0	0.0	0.0	0.0	100.0	0.0	0.0	0.0	0.0	0.0	94.3	0.0	0.0	0.0	0.0	0.0	0.0
5d.1	30.2	28.2	0.0	53.6	36.8	27.2	1.9	28.4	0.0	100.0	37.6	28.5	26.9	31.0	0.0	55.4	32.7	33.3	39.4	34.5	0.0
5e.1	29.7	20.6	0.0	33.4	73.2	36.6	0.0	23.0	0.0	37.6	100.0	43.9	25.9	23.5	0.0	33.7	43.5	24.6	25.0	22.9	0.0
8.1	21.4	17.1	0.0	34.1	49.6	51.8	0.0	17.9	0.0	28.5	43.9	100.0	22.6	17.2	0.0	33.1	48.4	26.8	27.7	26.2	0.0
7a.2	27.2	61.8	0.0	29.1	21.9	16.0	0.0	58.4	0.0	26.9	25.9	22.6	100.0	63.5	0.0	27.2	16.7	18.5	25.8	24.8	0.0
7b.2	32.9	82.2	0.0	32.7	19.2	13.4	0.5	64.3	0.0	31.0	23.5	17.2	63.5	100.0	0.0	32.2	15.1	18.8	27.0	28.7	0.0
7c.2	0.0	0.0	90.7	0.0	0.0	0.0	0.0	0.0	94.3	0.0	0.0	0.0	0.0	0.0	100.0	0.0	0.0	0.0	0.0	0.0	0.0
7d.1	27.8	29.0	0.0	81.0	31.9	29.6	0.4	25.7	0.0	55.4	33.7	33.1	27.2	32.2	0.0	100.0	33.7	27.0	43.6	35.4	0.0
9.1	21.4	13.2	0.0	32.9	45.5	81.6	0.0	14.2	0.0	32.7	43.5	48.4	16.7	15.1	0.0	33.7	100.0	21.0	20.4	19.4	0.0
don.1	17.4	19.5	0.0	30.9	26.3	21.2	12.4	20.1	0.0	33.3	24.6	26.8	18.5	18.8	0.0	27.0	21.0	100.0	23.1	30.5	16.8
myr.1	20.0	25.2	0.0	47.9	24.0	20.5	0.0	24.1	0.0	39.4	25.0	27.7	25.8	27.0	0.0	43.6	20.4	23.1	100.0	31.4	0.0
prm.1	24.6	30.4	0.0	37.1	21.7	21.0	1.5	28.3	0.0	34.5	22.9	26.2	24.8	28.7	0.0	35.4	19.4	30.5	31.4	100.0	0.0
tac.1	0.0	0.0	0.0	0.0	0.0	0.0	23.9	0.0	0.0	0.0	0.0	0.0	0.0	0.0	0.0	0.0	0.0	16.8	0.0	0.0	100.0

Monomers and Dimers

Figure S7.5. Heatmap of percent overlap of docked monomer ligands in *Hs*Aβ$_{42}$.

Monomers

ID	1a	1b	1c	1d	1e	Fc	5a	5b	5c	5d	5e	8	7a	7b	7c	7d	9	don	myr	prm	tac
1a.2	23.2	21.7	0.0	32.4	24.0	18.2	0.0	26.5	0.0	30.4	29.6	21.5	25.3	0.0	0.0	28.8	21.3	16.1	27.4	24.7	0.0
1b.2	22.2	28.6	0.0	31.2	15.9	12.6	0.0	27.8	0.0	28.2	20.3	16.9	19.2	0.0	0.0	31.0	12.9	19.1	17.4	30.2	0.0
1c.2	0.0	0.0	0.0	0.0	0.0	0.0	0.0	0.0	0.0	0.0	0.0	0.0	0.0	0.0	0.0	0.0	0.0	0.0	0.0	0.0	0.0
1d.1	44.2	53.7	0.0	60.8	31.3	30.6	0.0	62.9	0.0	53.6	33.0	33.8	37.7	0.0	0.0	87.2	32.2	29.1	24.0	36.3	0.0
1e.1	44.6	37.5	0.0	34.9	87.8	38.1	0.0	33.8	0.0	36.8	71.9	49.8	38.0	0.0	0.0	32.4	45.2	23.5	34.6	21.6	0.0
Fc.1	58.9	48.1	0.0	30.5	40.0	95.4	0.0	25.9	0.0	26.8	36.0	52.1	37.7	0.0	0.0	28.0	81.5	20.0	31.8	20.9	0.0
5a.2	0.0	0.7	25.9	1.5	0.0	0.0	0.0	0.1	0.0	1.9	0.0	0.0	0.0	28.0	0.0	0.5	0.0	12.6	0.0	1.7	24.2
5b.2	20.4	25.6	0.0	29.5	19.3	12.0	0.0	24.1	0.0	28.4	22.7	17.7	17.6	0.0	0.0	27.3	13.7	17.9	18.9	27.8	0.0
5c.2	0.0	0.0	0.0	0.0	0.0	0.0	0.0	0.0	0.1	0.0	0.0	0.0	0.0	0.0	0.1	0.0	0.0	0.0	0.0	0.0	0.0
5d.1	38.9	47.9	0.0	74.6	36.2	27.0	0.0	48.0	0.0	99.3	37.1	28.6	36.4	0.0	0.0	57.1	32.9	31.7	30.8	33.8	0.0
5e.1	40.4	34.6	0.0	34.3	80.0	36.3	0.0	33.3	0.0	37.2	97.8	44.1	43.9	0.0	0.0	35.2	43.2	21.6	32.5	22.8	0.0
8.1	55.5	47.0	0.0	31.3	50.0	52.9	0.0	34.8	0.0	28.5	43.9	99.4	33.5	0.0	0.0	32.1	47.4	24.6	29.9	26.6	0.0
7a.2	25.4	27.4	0.0	27.7	21.5	15.9	0.0	28.1	0.0	26.9	25.6	22.4	20.9	0.0	0.0	28.8	16.3	17.7	20.7	24.7	0.0
7b.2	22.0	28.2	0.0	31.3	19.2	13.2	0.0	29.8	0.0	31.2	23.2	17.1	22.9	0.0	0.0	33.8	14.7	18.6	19.8	28.2	0.0
7c.2	0.0	0.0	0.0	0.0	0.0	0.0	0.0	0.0	0.0	0.0	0.0	0.0	0.0	0.0	0.0	0.0	0.0	0.0	0.0	0.0	0.0
7d.1	41.3	50.2	0.0	56.8	33.2	29.4	0.0	62.6	0.0	55.4	33.2	33.2	37.4	0.0	0.0	90.0	33.0	26.7	24.9	34.7	0.0
9.1	52.0	43.4	0.0	34.3	47.8	78.2	0.0	29.2	0.0	32.3	42.3	48.6	36.1	0.0	0.0	32.1	97.4	18.4	38.0	19.3	0.0
don.1	30.0	36.8	13.4	34.8	25.5	21.4	0.0	27.5	0.0	33.0	24.5	26.9	18.1	14.3	0.0	28.7	21.1	66.8	14.7	31.2	16.8
myr.1	38.9	46.7	0.0	41.7	25.7	20.4	0.0	45.7	0.0	39.4	24.9	27.4	24.7	0.0	0.0	44.5	20.1	22.9	16.2	31.3	0.0
prm.1	27.9	34.4	0.0	39.0	21.3	21.5	0.0	28.2	0.0	34.5	22.5	26.2	23.1	0.0	0.0	35.5	19.5	29.7	25.0	94.5	0.0
tac.1	0.0	0.0	57.1	0.0	0.0	0.0	0.0	0.0	0.0	0.0	0.0	0.0	0.0	51.7	0.0	0.0	0.0	18.1	0.0	0.0	98.8

Monomers and Dimers

Figure S7.6. Mixed heatmap of percent overlap of docked monomer and dimer ligands in *Hs*Aβ$_{42}$.

Monomers (columns) / Monomers (rows)

ID	1a	1b	1c	1d	1e	1o	5a	5b	5c	5d	5e	5o	7a	7b	7c	7d	7o	bnp	don	myr	prm	tac
1a	100.0	74.1	0.0	0.0	0.0	0.0	69.8	63.2	0.0	0.0	61.0	0.0	75.7	65.0	0.0	0.0	0.0	0.0	0.0	0.0	0.0	43.0
1b	74.1	100.0	0.0	0.0	0.0	0.0	59.6	81.6	0.0	0.0	53.9	0.0	64.4	82.9	0.0	0.0	0.0	0.0	0.0	0.0	0.0	53.3
1c	0.0	0.0	100.0	20.9	50.8	0.0	0.0	0.0	80.3	38.1	0.0	0.0	0.0	0.0	86.8	20.0	0.0	0.0	31.8	30.2	48.8	0.0
1d	0.0	0.0	20.9	100.0	29.2	0.0	0.0	0.0	27.8	37.0	0.0	0.0	0.0	0.0	25.6	84.3	0.0	0.0	12.7	36.5	26.8	0.0
1e	0.0	0.0	50.8	29.2	100.0	0.0	0.0	0.0	42.5	30.5	0.0	0.0	0.0	0.0	45.0	27.6	0.0	0.0	17.9	39.9	28.9	0.0
1o	0.0	0.0	0.0	0.0	0.0	100.0	0.0	0.0	0.0	0.0	0.0	0.0	0.0	0.0	0.0	0.0	63.7	29.6	0.0	0.0	0.0	0.0
5a	69.8	59.6	0.0	0.0	0.0	0.0	100.0	58.3	0.0	0.0	57.7	0.0	74.2	65.3	0.0	0.0	0.0	0.0	0.0	0.0	0.0	37.2
5b	63.2	81.6	0.0	0.0	0.0	0.0	58.3	100.0	0.0	0.0	48.9	0.0	56.3	75.9	0.0	0.0	0.0	0.0	0.0	0.0	0.0	45.7
5c	0.0	0.0	80.3	27.8	42.5	0.0	0.0	0.0	100.0	40.1	0.0	0.0	0.0	0.0	85.3	26.6	0.0	0.0	30.0	33.3	56.4	0.0
5d	0.0	0.0	38.1	37.0	30.5	0.0	0.0	0.0	40.1	100.0	0.0	0.0	0.0	0.0	42.4	34.9	0.0	0.0	19.2	26.8	36.5	0.0
5e	61.0	53.9	0.0	0.0	0.0	0.0	57.7	48.9	0.0	0.0	100.0	0.0	58.8	51.0	0.0	0.0	0.0	0.0	0.0	0.0	0.0	38.1
5o	0.0	0.0	0.0	0.0	0.0	0.0	0.0	0.0	0.0	0.0	0.0	100.0	0.0	0.0	0.0	0.0	0.0	0.0	0.0	0.0	0.0	0.0
7a	75.7	64.4	0.0	0.0	0.0	0.0	74.2	56.3	0.0	0.0	58.8	0.0	100.0	64.4	0.0	0.0	0.0	0.0	0.0	0.0	0.0	39.3
7b	65.0	82.9	0.0	0.0	0.0	0.0	65.3	75.9	0.0	0.0	51.0	0.0	64.4	100.0	0.0	0.0	0.0	0.0	0.0	0.0	0.0	48.9
7c	0.0	0.0	86.8	25.6	45.0	0.0	0.0	0.0	85.3	42.4	0.0	0.0	0.0	0.0	100.0	24.5	0.0	0.0	29.6	33.9	55.9	0.0
7d	0.0	0.0	20.0	84.3	27.6	0.0	0.0	0.0	26.6	34.9	0.0	0.0	0.0	0.0	24.5	100.0	0.0	0.0	12.9	33.4	25.5	0.0
7o	0.0	0.0	0.0	0.0	0.0	63.7	0.0	0.0	0.0	0.0	0.0	0.0	0.0	0.0	0.0	0.0	100.0	34.6	0.0	0.0	0.0	0.0
bnp	0.0	0.0	0.0	0.0	0.0	29.6	0.0	0.0	0.0	0.0	0.0	0.0	0.0	0.0	0.0	0.0	34.6	100.0	0.0	0.0	0.0	0.0
don	0.0	0.0	31.8	12.7	17.9	0.0	0.0	0.0	30.0	19.2	0.0	0.0	0.0	0.0	29.6	12.9	0.0	0.0	100.0	16.3	27.4	0.0
myr	0.0	0.0	30.2	36.5	39.9	0.0	0.0	0.0	33.3	26.8	0.0	0.0	0.0	0.0	33.9	33.4	0.0	0.0	16.3	100.0	27.8	0.0
prm	0.0	0.0	48.8	26.8	28.9	0.0	0.0	0.0	56.4	36.5	0.0	0.0	0.0	0.0	55.9	25.5	0.0	0.0	27.4	27.8	100.0	0.0
tac	43.0	53.3	0.0	0.0	0.0	0.0	37.2	45.7	0.0	0.0	38.1	0.0	39.3	48.9	0.0	0.0	0.0	0.0	0.0	0.0	0.0	100.0

Figure S7.7. Heatmap of percent overlap of a set of docked ligands in *Ss*CES1.

Monomers (columns) / Monomers and Dimers (rows)

ID	1a	1b	1c	1d	1e	Fc	5a	5b	5c	5d	5e	8	7a	7b	7c	7d	9	bnp	don	myr	prm	tac
1a.2	18.6	18.1	0.0	0.0	0.0	0.0	25.0	22.8	0.0	0.0	26.3	0.0	21.3	20.2	0.0	0.0	0.0	0.0	0.3	0.0	0.0	9.3
1b.2	0.0	0.0	15.5	15.4	16.8	0.0	0.0	0.0	17.8	18.2	0.0	0.0	0.0	0.0	18.8	14.1	0.0	0.0	4.4	18.4	19.7	0.0
1c.2	0.0	0.0	11.4	21.1	9.6	0.0	0.0	0.0	15.9	19.2	0.0	0.0	0.0	0.0	15.1	20.2	0.0	0.0	2.6	10.0	17.8	0.0
1d.1	0.0	0.0	20.8	98.8	29.1	0.0	0.0	0.0	27.7	37.3	0.0	0.0	0.0	0.0	25.9	84.0	0.0	0.0	12.7	36.8	27.0	0.0
1e.1	0.0	0.0	0.0	0.0	0.0	0.0	0.0	0.0	0.0	0.0	0.0	59.8	0.0	0.0	0.0	0.0	0.0	0.0	0.0	0.0	0.0	0.0
Fc.1	0.0	0.0	0.0	0.0	0.0	97.7	0.0	0.0	0.0	0.0	0.0	0.0	0.0	0.0	0.0	0.0	62.6	29.7	0.0	0.0	0.0	0.0
5a.2	0.0	0.0	15.3	24.0	16.5	0.0	0.0	0.0	18.9	21.0	0.0	0.0	0.0	0.0	18.9	25.2	0.0	0.0	4.6	16.7	22.0	0.0
5b.2	0.0	0.0	8.6	18.4	10.1	0.0	0.0	0.0	11.3	13.1	0.0	0.0	0.0	0.0	11.3	19.9	0.0	0.0	3.4	11.3	13.8	0.0
5c.2	0.0	0.0	21.2	9.5	13.5	0.0	0.0	0.0	23.3	17.4	0.0	0.0	0.0	0.0	24.3	9.5	0.0	0.0	14.9	8.1	26.4	0.0
5d.1	0.0	0.0	38.3	37.2	30.4	0.0	0.0	0.0	40.6	98.3	0.0	0.0	0.0	0.0	42.9	35.1	0.0	0.0	19.0	26.8	37.0	0.0
5e.1	0.0	0.0	41.3	20.8	47.0	0.0	0.0	0.0	37.1	30.3	0.0	0.0	0.0	0.0	38.9	20.6	0.0	0.0	21.4	25.4	30.0	0.0
8.1	0.0	0.0	0.0	0.0	0.0	0.0	0.0	0.0	0.0	0.0	0.0	93.6	0.0	0.0	0.0	0.0	0.0	0.0	0.0	0.0	0.0	0.0
7a.2	17.2	16.4	0.0	0.0	0.0	0.0	22.3	20.4	0.0	0.0	23.6	0.0	19.1	18.2	0.0	0.0	0.0	0.0	0.3	0.0	0.0	7.8
7b.2	0.0	0.0	9.1	18.9	10.7	0.0	0.0	0.0	11.7	13.1	0.0	0.0	0.0	0.0	11.9	20.7	0.0	0.0	3.4	11.8	14.2	0.0
7c.2	0.0	0.0	21.8	10.2	13.8	0.0	0.0	0.0	24.4	17.8	0.0	0.0	0.0	0.0	25.3	10.1	0.0	0.0	15.2	8.7	27.3	0.0
7d.1	0.0	0.0	19.0	76.3	25.7	0.0	0.0	0.0	25.1	37.2	0.0	0.0	0.0	0.0	23.3	88.3	0.0	0.0	12.7	33.3	23.6	0.0
9.1	0.0	0.0	0.0	0.0	0.0	63.3	0.0	0.0	0.0	0.0	0.0	0.0	0.0	0.0	0.0	0.0	96.7	34.5	0.0	0.0	0.0	0.0
bnp.1	0.0	0.0	0.0	0.0	0.0	29.6	0.0	0.0	0.0	0.0	0.0	0.0	0.0	0.0	0.0	0.0	34.6	99.9	0.0	0.0	0.0	0.0
don.1	0.0	0.0	32.3	12.5	18.3	0.0	0.0	0.0	30.2	19.8	0.0	0.0	0.0	0.0	30.1	12.6	0.0	0.0	92.7	16.5	27.9	0.0
myr.1	0.0	0.0	30.2	36.4	40.2	0.0	0.0	0.0	33.3	26.7	0.0	0.0	0.0	0.0	33.8	33.3	0.0	0.0	16.3	99.6	27.7	0.0
prm.1	0.0	0.0	51.1	15.3	30.8	0.0	0.0	0.0	48.6	29.1	0.0	0.0	0.0	0.0	52.8	15.2	0.0	0.0	27.2	27.7	67.5	0.0
tac.1	41.9	53.9	0.0	0.0	0.0	0.0	36.3	46.3	0.0	0.0	37.2	0.0	38.4	48.9	0.0	0.0	0.0	0.0	0.0	0.0	0.0	95.8

Figure S7.8. Mixed heatmap of percent overlap of docked monomer and dimer ligands in *Ss*CES1.

Section S8. Validation of molecular docking method

MSC-DP cells were obtained from the “Vertebrate Cell Culture Collection” supported by the Ministry of Education and Science of the Russian Federation (Agreement No. 075-15-2021-683). Reprogramming of MSC-DP into neuronal phenotype (MSC-Neu) was performed by culturing for 5 days in Neurobasal medium (BioinnLabs. Russia) with the addition of Neuromax component (PanEco. Russia). 3% fetal bovine serum, 100 units/ml penicillin and 0.1 mg/ml streptomycin (PanEco. Russia). Neuronal phenotype was verified by analyzing the expression of a panel of mature neuronal markers, including

β3-tubulin, NeuN, MAP2, synaptophysin (Syp), PSD95, and Neuro D1 using real-time polymerase chain reaction (RT-PCR).

Validation of the molecular docking method was done using the SHEBA procedure (Jung and Lee, 2000) in YASARA to conduct a 3D alignment of the *Hs*AChE protein containing the top-ranked docking pose of the reference compound, donepezil, with a crystal structure of *Hs*AChE in complex with donepezil (PDB ID 4EY7; 2.35 Å resolution). As shown in Figure S7.1, the ligands approached congruency with a heavy-atom RMSD of 0.541 Å as calculated by DockRMSD [153].

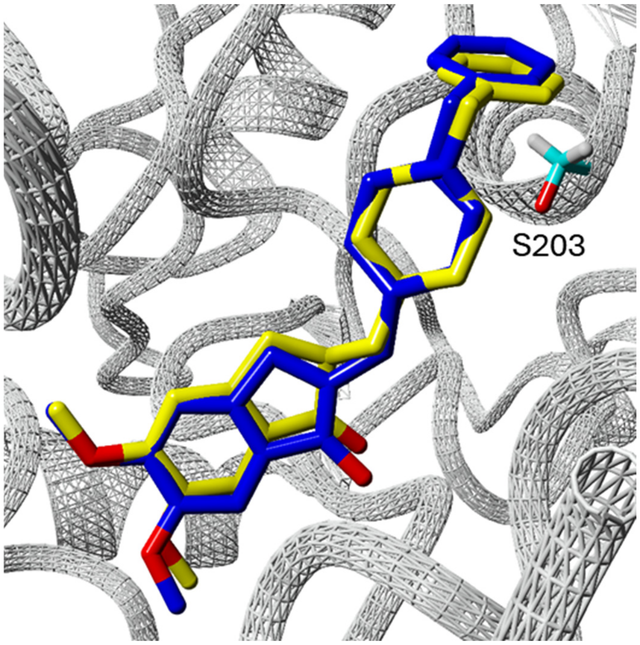

Figure S8.1. Structure of *Hs*AChE generated by the AlphaFold3 reproduction code, Protenix 0.5.0 [76] containing the top-scoring docking pose from the implementation of Autodock Vina 1.2.5 [116,117] in YASARA 25.07.15 for Linux [104] of the reference ligand, donepezil (carbon atoms colored blue), 3D-aligned with an X-ray crystal structure of *Hs*AChE in complex with donepezil (carbon atoms colored yellow) (PDB ID 4EY7; resolution 2.35 A). Ligands shown as sticks; protein secondary structure shown as gray mesh. Hydrogen atoms hidden for clarity. Active site S203 residue shown for orientation. Protein alignment done with SHEBA [154] in YASARA. Ligand RMSD = 0.541 Å calculated by DockRMSD [153]. Image rendered in YASARA.

Section S9. Reprogramming of mesenchymal stem cells obtained from human dental pulp (MSC-DP)

Reprogramming of MSC-DP into neuronal phenotype (MSC-Neu) was performed by culturing for 5 days in Neurobasal medium (BioinnLabs. Russia) with the addition of Neuromax component (PanEco. Russia). 3% fetal bovine serum, 100 units/ml penicillin and 0.1 mg/ml streptomycin (PanEco. Russia). Neuronal phenotype was verified by analyzing the expression of a panel of mature neuronal markers, including β3-tubulin, NeuN, MAP2, synaptophysin (Syp), PSD95, and Neuro D1 using real-time polymerase chain reaction (RT-PCR).

RNA was isolated using ExtractRNA (Eurogen, Moscow, Russia) and then reverse transcribed using the MMLV RT kit (Eurogen, Moscow, Russia) according to the manufacturer's instructions. RT-PCR was

performed using the CFX96 real-time PCR detection system (BioRad, Hercules, CA, USA) and qPCRmix-HS SYBR (Eurogen, Moscow, Russia) according to the manufacturer's protocol. The identity of the amplicons was confirmed by melting curve analysis. The primer sequences are presented in Table 2; all primers were synthesized by Evrogen (Moscow, Russia). PCR parameters were 5 min pre-denaturation at 95°C, followed by 40 cycles of 30 sec at 95°C, 30 sec at 65°C, and 30 sec at 70°C. Data were analyzed for fold change using BioRad CFX software. Typical results of MSC-Neu neuronal phenotype verification were published earlier [102].

Table S4. Sequences of primers used in the study.

Actin	Forward	5′-TCAATGTCCCAGCCATGTATGT-3′
	Reverse	5′-GTGACACCATCTCCAGAGTCC-3′
NeuN	Forward	5′-CAAGGACGGTCCAGAAGGAG-3′
	Reverse	5′-GGTAGTGGGAGGTGAGGTCT-3′
MAP2	Forward	5′-GGAGGGCGCTAAGTCCG-3′
	Reverse	5′-AAAATCTGGGCGCAGAAACTG-3′
Neuro D1	Forward	5′-TCTTCCACGTTAAGCCTCCG-3′
	Reverse	5′- CCATCAAAGGAAGGGCTGGT-3′
β3-tubulin	Forward	5′-CCATGAAGGAGGTGGACGAG-3′
	Reverse	5′-ACGTTGTTGGGGATCCACTC-3′
SYP	Forward	5′-CTTCGCCATCTTOGCCTTTG-3′
	Reverse	5′-TCACTCTCGGTCTTGTTGGC-3′
PSD95	Forward	5′-GGATATGTGAACGGGACCGA-3′
	Reverse	5′-AAGCCCAGACCTGAGTTACC-3′
